# Regiodivergent Functionalization of Protected and Unprotected Carbohydrates using Photoactive 4‐Tetrafluoropyridinylthio Fragment as an Adaptive Activating Group

**DOI:** 10.1002/anie.202412436

**Published:** 2024-10-24

**Authors:** Shen Cao, Haobo Zhang, Mingshuo Chen, Niming Zhu, Beibei Zhan, Peng Xu, Xiaoping Chen, Biao Yu, Xiaheng Zhang

**Affiliations:** ^1^ School of Chemistry and Materials Science, Hangzhou Institute for Advanced Study University of Chinese Academy of Sciences 1 Sub-lane Xiangshan Hangzhou 310024 P. R. China; ^2^ State Key Laboratory of Chemical Biology, Shanghai Institute of Organic Chemistry, University of Chinese Academy of Sciences Chinese Academy of Sciences 345 Lingling Road Shanghai 200032 China

**Keywords:** Carbohydrates, unprotected saccharides, glycosyl radicals, radical reactions

## Abstract

The selective functionalization of carbohydrates holds a central position in synthetic carbohydrate chemistry, driving the ongoing quest for ideal approaches to manipulate these compounds. In this study, we introduce a general strategy that enables the regiodivergent functionalization of saccharides. The use of electron‐deficient photoactive 4‐tetrafluoropyridinylthio (SPyf) fragment as an adaptable activating group, facilitated efficient functionalization across all saccharide sites. More importantly, this activating group can be directly installed at the C1, C5 and C6 positions of biomass‐derived carbohydrates in a single step and in a site‐selective manner, allowing for the efficient and precision‐oriented modification of unprotected saccharides and glycans.

## Graphical abstract

A general platform has been developed for the regiodivergent functionalization of both protected and unprotected carbohydrates by utilizing the electron‐deficient thiyl fragment as a versatile and adaptive activating group. This method facilitates the efficient generation of carbon radicals at different saccharide sites, providing a valuable tool for the expeditous synthesis of a diverse array of high‐value *C*‐glycosides and rare sugars.

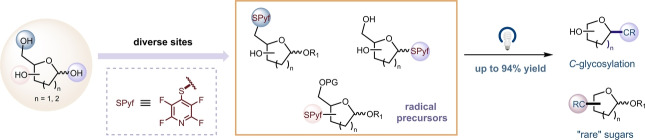

## Introduction

**Figure 1:**
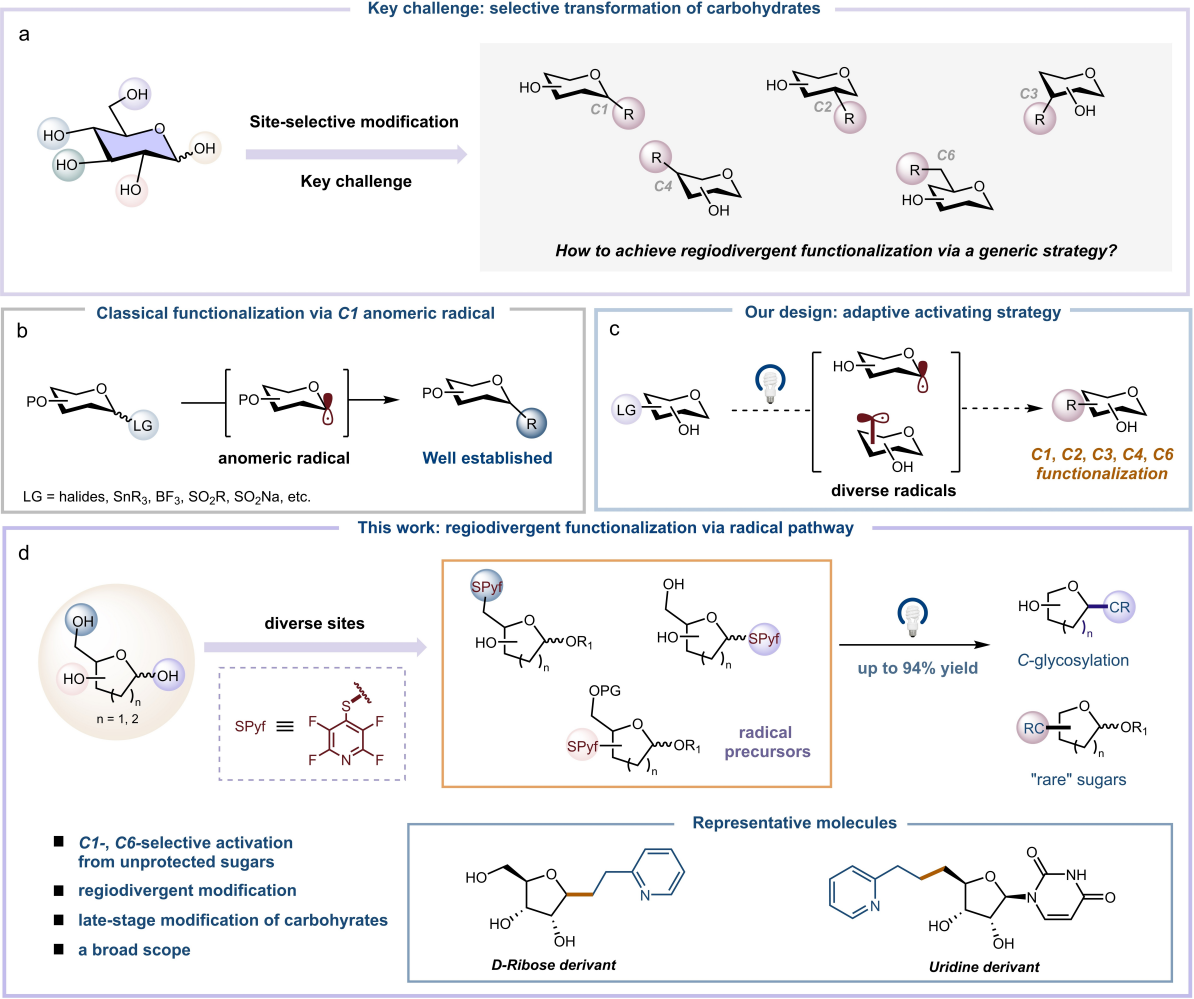
Regiodivergent functionalization of carbohydrates. **a**, Selective transformation of carbohydrates. **b**, Classical functionalization via C1 anomeric radical. **c**, Adaptive activating strategy. **d**, Regiodivergent functionalization of carbohydrates via radical pathway.

Carbohydrates are essential biomolecules, playing crucial roles in a plethora of biological processes.[^1^, ^2^] Consequently, it is important to devise efficient methodologies that facilitate the functionalization and precise manipulation of these compounds. Nonetheless, the chemical intricacy of carbohydrates, characterized by the abundance of chemically indistinguishable O−H, C−H and C−O bonds, presents a substantial challenge for selective skeletal modifications, especially in a generic approach (Figure 1a).[^3^, ^4^] In practice, the domain of carbohydrate chemistry still relies on the utilization of protecting groups or lengthy synthetic routes to access structurally defined glycosides, which significantly diminishes synthetic efficiency.[^5^, ^6^] As such, the identification of general and efficient methods for selectively transforming carbohydrates while minimizing the need for protecting groups would be incredibly valuable.

Over the past century, a myriad of chemical transformations has been developed, allowing the selective functionalization of unprotected or partially protected carbohydrates.[^7^, ^8^, ^9^] However, the majority of these transformations capitalize on the inherent reactivity of O−H groups. Emerging recently, photoredox catalysis has become a valuable technique for the selective transformation of carbohydrates at unconventional or complementary sites (through the use of C−H and C−O bonds), taking advantage of distinctive reaction pathways via radical‐mediated transformations.[^10^, ^11^, ^12^] In this context, the past few years have witnessed a series of impressive advancements by MacMillan,^[13]^ Minnaard,^[14]^ Taylor^[15]^ and Wendlandt^[16]^ group, employing dual hydrogen‐atom transfer (HAT)/photoredox catalysis to achieve selective manipulation of C−H bonds in carbohydrates. The utilization of C−O bond activation emerges as a complementary approach for sugar core modification. This technique entails the direct activation of C−O bonds using a suitable activating group, which is followed by the cleavage of these bonds to generate carbohydrate‐based C‐radicals that can subsequently engage in a range of reactions.[^17^, ^18^, ^19^] A notable example was demonstrated recently by Chi and co‐workers,^[20]^ who showed that the selective activation and modification of C−O bonds in unprotected carbohydrates could be achieved through a two‐step sequence involving NHC‐catalyzed site‐selective acylation followed by photocatalytic C−O bond cleavage. Nevertheless, most of the methods are currently limited by challenges in selectively activating analogous C−O bonds and the high bond dissociation energy of C(*sp* ^*3*^)−O bond.^[21]^

A promising alternative approach is to transform the C−O bond into a precursor more amenable to radical formation, mediated by an appropriate activating group. This precursor can then be subjected to a single electron transfer (SET) event to generate C‐radicals, namely glycosyl radicals, which can further undergo functionalization.[^22^, ^23^, ^24^, ^25^, ^26^, ^27^, ^28^, ^29^, ^30^, ^31^, ^32^, ^33^, ^34^, ^35^, ^36^, ^37^] Yet currently selective functionalization of carbohydrates has predominantly concentrated on the C1 position by generating anomeric radicals, while modifications of other sites on the carbohydrate skeletons for rare sugar synthesis have been relatively unexplored (Figure 1b).[^38^, ^39^] Given that rare sugars play a significant role in a variety of bioactive natural products and pharmaceuticals, we recognize the need and envision new activating group capable of effectively initiate the production of C‐radicals at both the anomeric position and other sites in a regiodivergent approach, thereby facilitating subsequent functionalization (Figure 1c).

While conventional alkyl sulfides are typically resistant to single electron reduction, the incorporation of an electron‐withdrawing group, such as perfluorinated pyridine ring, can render the corresponding sulfides susceptible to photoredox conditions, thereby facilitating the generation of alkyl radicals. Precedents set by the Dilman group have established the feasibility of producing alkyl radicals by employing the 4‐tetrafluoropyridinylthio (SPyf) fragment as a photoactive group.[^40^, ^41^, ^42^, ^43^] Building on these findings, we questioned whether it would be possible to leverage an electron‐deficient thiyl fragment as an appropriate activating group for generating C‐radicals at the carbohydrate scaffold, which could then be subjected to further manipulation. Herein, we report our successful endeavors in establishing a general strategy for the regiodivergent functionalization of carbohydrates under photoredox catalysis, utilizing the SPyf fragment as a versatile and adaptive activating group. This approach allows the manipulation of carbohydrates at all sites, thereby providing access to a diverse range of high‐value rare sugars. Remarkably, the direct, one‐step introduction of this activating group at the C1, C5 and C6 positions of biomass carbohydrates enables the site‐selective functionalization of unprotected saccharides and glycans, complementing existing techniques. We anticipate that this strategy will serve as a valuable tool for the efficient and precise manipulation of carbohydrates (Figure 1d). During the preparation of this manuscript, the Koh and Davis group disclosed a strategy that uses the SPyf fragment as an anomeric activation group for generating glycosyl radicals.[^44^, ^45^, ^46^]

## Results and Discussion

**Figure 2:**
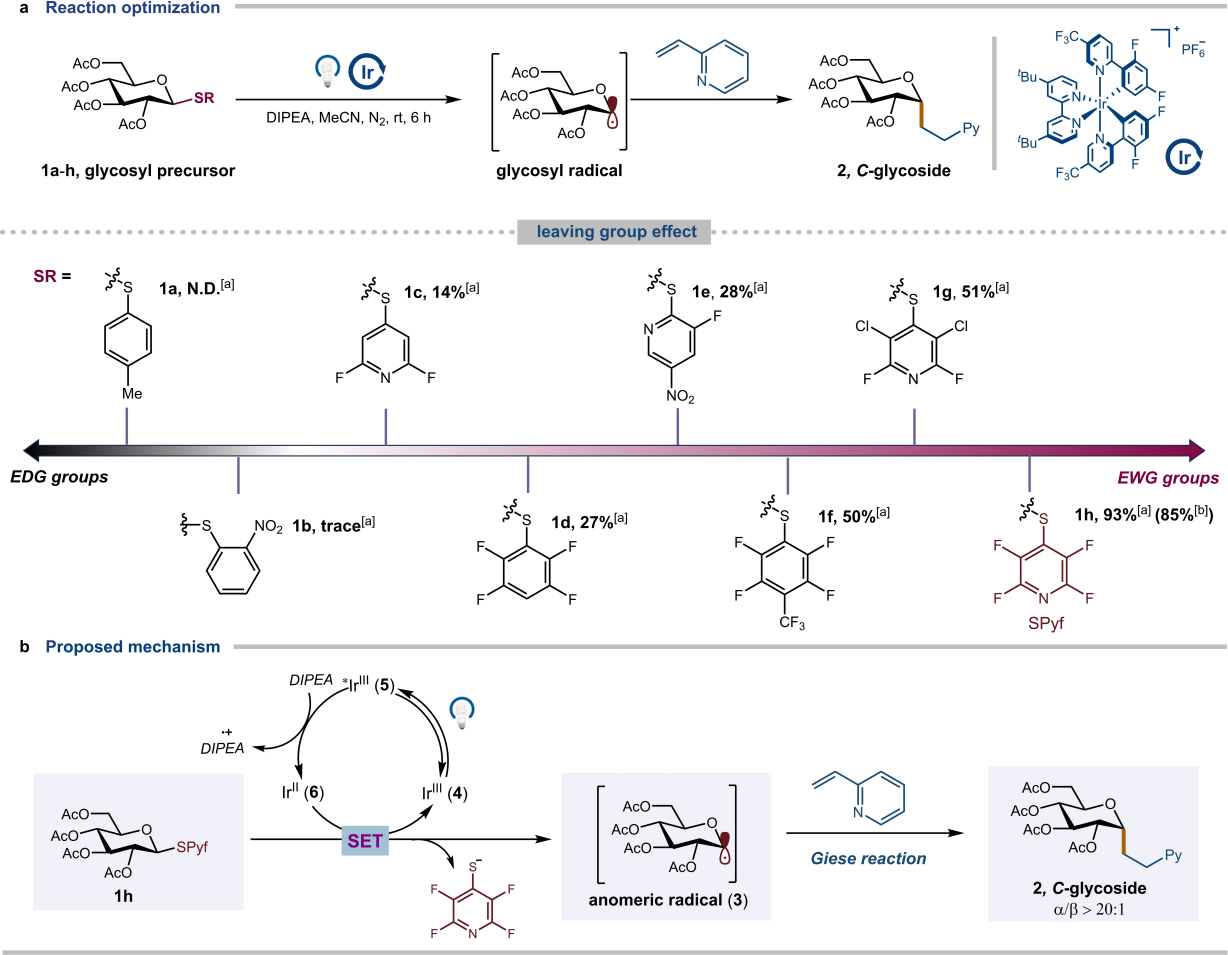
Optimization of different leaving groups and proposed mechanism. **a**, Optimization of leaving groups. **b**, Proposed mechanism. Performed with glycosyl precursors **1 a**–**h** (0.1 mmol, 1.0 equiv.), 2‐vinylpyridine (1.5 equiv.), DIPEA (2.0 equiv.), Ir[dF(CF_3_)ppy]_2_(dtbbpy)PF_6_ (2 mol %) in MeCN (0.1 M), Kessil LEDs 427 nm (40 W) for 6 h. [a] Yield by ^1^H NMR analysis of the crude reaction mixtures using mesitylene as internal standard. [b] Isolated yield. EDG, electron donating group; EWG, electron withdrawing group; SET, single electron transfer.

Our investigation commenced with the generation of glycosyl radicals from thioglycosides and subsequent Giese addition under mild photoirradiation conditions (see Supporting Information for details). Recognizing the significance of selecting an appropriate glycosyl precursor for this transformation, a range of aryl thioglycosides (**1 a**–**1 h**) bearing both electron‐donating groups and electron‐withdrawing groups were prepared. We then evaluated the efficiency of Giese addition with 2‐vinylpyridine in the presence of Ir[dF(CF_3_)ppy]_2_(dtbbpy)PF_6_ and DIPEA at room temperature with MeCN as solvent. As disclosed in Figure 2a, the presence of pyridine rings and fluorine‐containing substituents significantly improve the yield of *C*‐glycoside **2**, potentially due to the more easily reducible electron‐deficient aromatic rings, leading to faster glycosyl radical formation (**1 h**, 85 % isolated yield). The addition byproduct of thiolate onto 2‐vinylpyridine was detected by GC‐MS analysis, indicating the in situ formation of a departing thiolate. Control experiments revealed that light, photocatalyst and reductant are all essential for this transformation (see Supporting Information for details). A plausible pathway was proposed for this transformation (Figure 2b). Visible‐light photo‐excitation of **4** would generate excited state photocatalyst **5**, which underwent a SET event with DIPEA to give **6**. Subsequent SET reduction with aryl thioglycoside **1 h** generated a glycosyl radical **3** and 2,3,5,6‐tetrafluoropyridine‐4‐thiolate. The glycosyl radical then underwent Giese addition with the alkene, affording the desired *C*‐glycoside **2** in a stereoselective manner.

The power of our methodology is exemplified by its wide applicability. With 2‐vinylpyridine as the coupling partner (Table 1), tetrafluoropyridinyl thioglycosides derived from various monosaccharides, including glucose, galactose, mannose, rhamnose, and glucopyranuronic acid readily participated in this reaction, yielding the desired products in high yields with excellent stereoselectivity (**2**, **7**–**10**, 72–94 % yields). Additionally, ribose and mannofuranose precursors were viable under the reaction conditions, furnishing the corresponding products **11** and **12** in 77 % and 84 % yield, respectively. Furthermore, those prepared from disaccharides such as maltose and lactose were also competent substrates, affording the C‐linked glycoconjugates **13** and **14** in good yields. The efficient transformation of diverse sterically congested hydroxy groups on carbohydrate scaffolds is itself nontrivial, whether selective or not.^[20]^ Notably, our strategy allows for the direct tagging (C−S bond formation) and editing (C−C bond formation) of a specific hydroxy group at all saccharide sites, which dramatically expedite the preparation of uncommon sugars, especially deoxygenated sugars, that are typically challenging to access (**15**–**19**, 34–85 % yields). Remarkably, this protocol exhibits excellent stereoselectivity throughout the C−C bond formation stage, likely attributed to the steric shielding of the nearby substituents.[^15^, ^16^, ^20^] We next investigated the scope of the reaction with respect to the alkene coupling partner. A number of electron‐deficient alkenes could participate in this reaction. Vinyl‐substituted heterocyclic ring such as pyridines (**20**–**26**), isoquinolines (**27**, **28**), quinoline (**29**), and pyrimidine (**30**) were all compatible substrates, providing the desired products in acceptable yields. Moreover, styrene derivatives were also reactive, and the corresponding alkyl *C*‐glycosides **31**–**34** were obtained in 72–85 % yields. Importantly, other Michael acceptors bearing complex skeletons derived from natural products, such as epiandrosterone (**35**), dehydroabietylamine (**36**), diacetonefructose (**37**), and 5‐aminosalicylic acid (**38**), provided good yields, showcasing the potential for late‐stage functionalization.

**Table 1:**
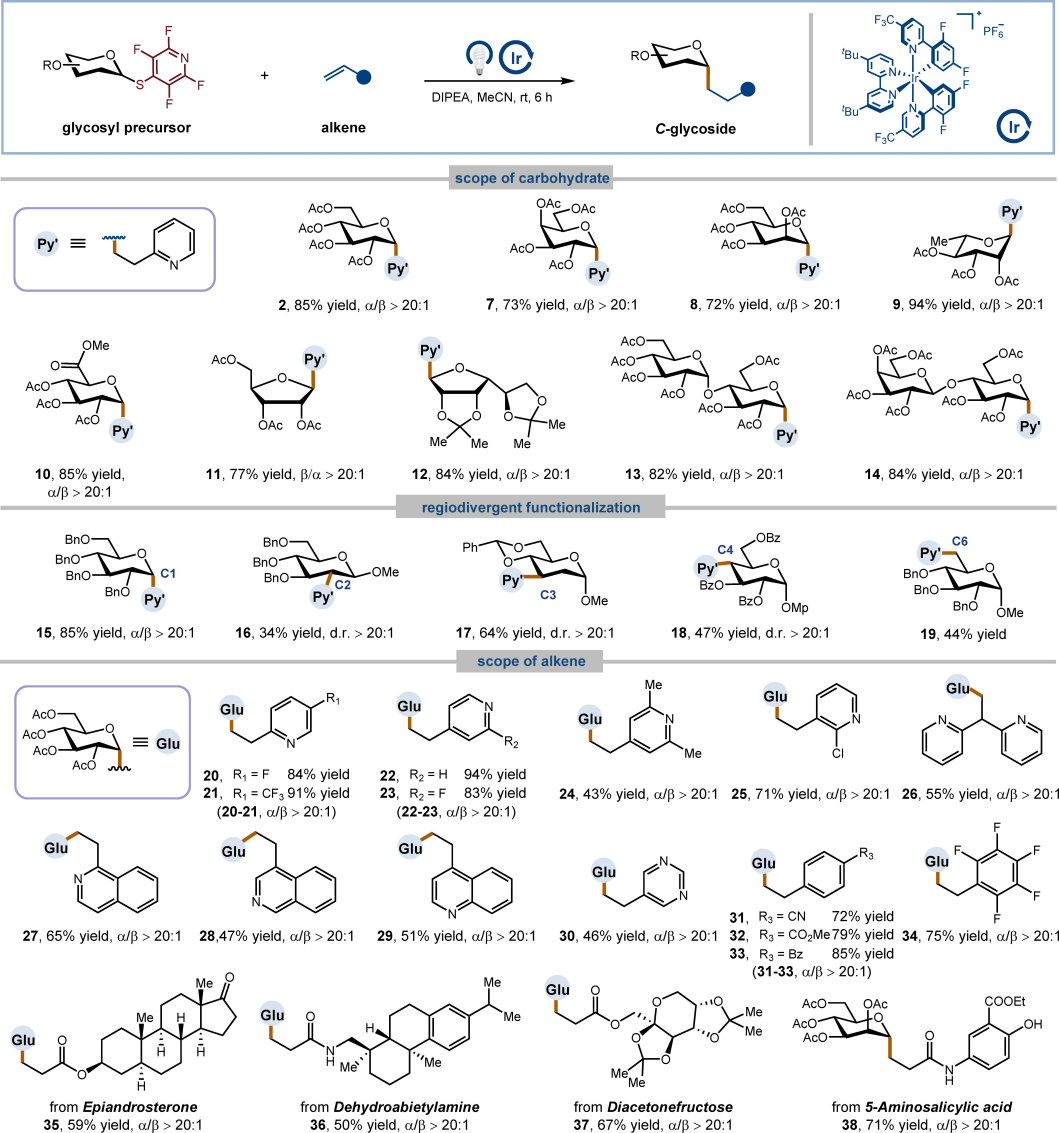
Scope of carbohydrates and alkenes.^[a]^

[a] Isolated yields. The anomeric ratios of the isolated and purified products were determined by ^1^H NMR spectroscopic analysis. General reaction conditions: glycosyl precursor (0.1 mmol, 1.0 equiv.), alkene (1.5 equiv.), DIPEA (2.0 equiv.), Ir[dF(CF_3_)ppy]_2_(dtbbpy)PF_6_ (2 mol %) in MeCN (0.1 M), Kessil LEDs 427 nm (40 W) for 6 h. See Supporting Information for full experimental details. MP=*p*‐methoxyphenyl.

After establishing proof‐of‐concept with the aforementioned outcomes, we became intrigued by the potential application of our reactions in the functionalization of unprotected saccharides. These compounds, characterized by multiple free hydroxyl groups and stereocenters, pose a longstanding challenge for site‐selective and stereoselective modifications. Our study began with the development of a synthetic route to C1‐, C5‐ or C6‐selective radical precursors in a single step (Table 2). To achieve this, dimethylimidazolium 4‐tetrafluoropyridinylthio (DMSPyf) conjugates were conveniently prepared on multigram scale by simply mixing Shoda's reagent, (2‐chloro‐1,3‐dimethylimidazolinium chloride, DMC), with tetrafluoropyridine‐4‐thiol.^[47]^ Encouragingly, this reagent exhibited sufficient electrophilic to react with the anomeric hydroxyl group of unprotected saccharides, yielding C1‐selective radical precursors in a single step. Furthermore, the other two precursors could be efficiently prepared via the Mitsunobu reaction by utilizing tetrafluoropyridine‐4‐thiol as nucleophile. Having successfully prepared large quantities of unprotected thioglycoside precursors, we next explored their reactivities and site‐selectivities with Ibrutinib or 2‐vinylpyridine (Table 2). First, a diverse range of glycosyl units smoothly underwent this reaction. Products substituted with glucosyl (**39**, **44**), mannosyl (**40**, **45**), galactosyl (**41**, **46**), and rhamnosyl (**42**, **47**) were all obtained in good to excellent yields. It was noteworthy that the formation of unprotected furanoside (**43**) also occurred with moderate yield but excellent stereoselectivity. Selective modification of carbohydrates is crucial for accessing uncommon sugars, which plays a vital role in numerous bioactive natural products and drugs and can serve as chemical tools to interrogate glycan function.^[48]^

**Table 2:**
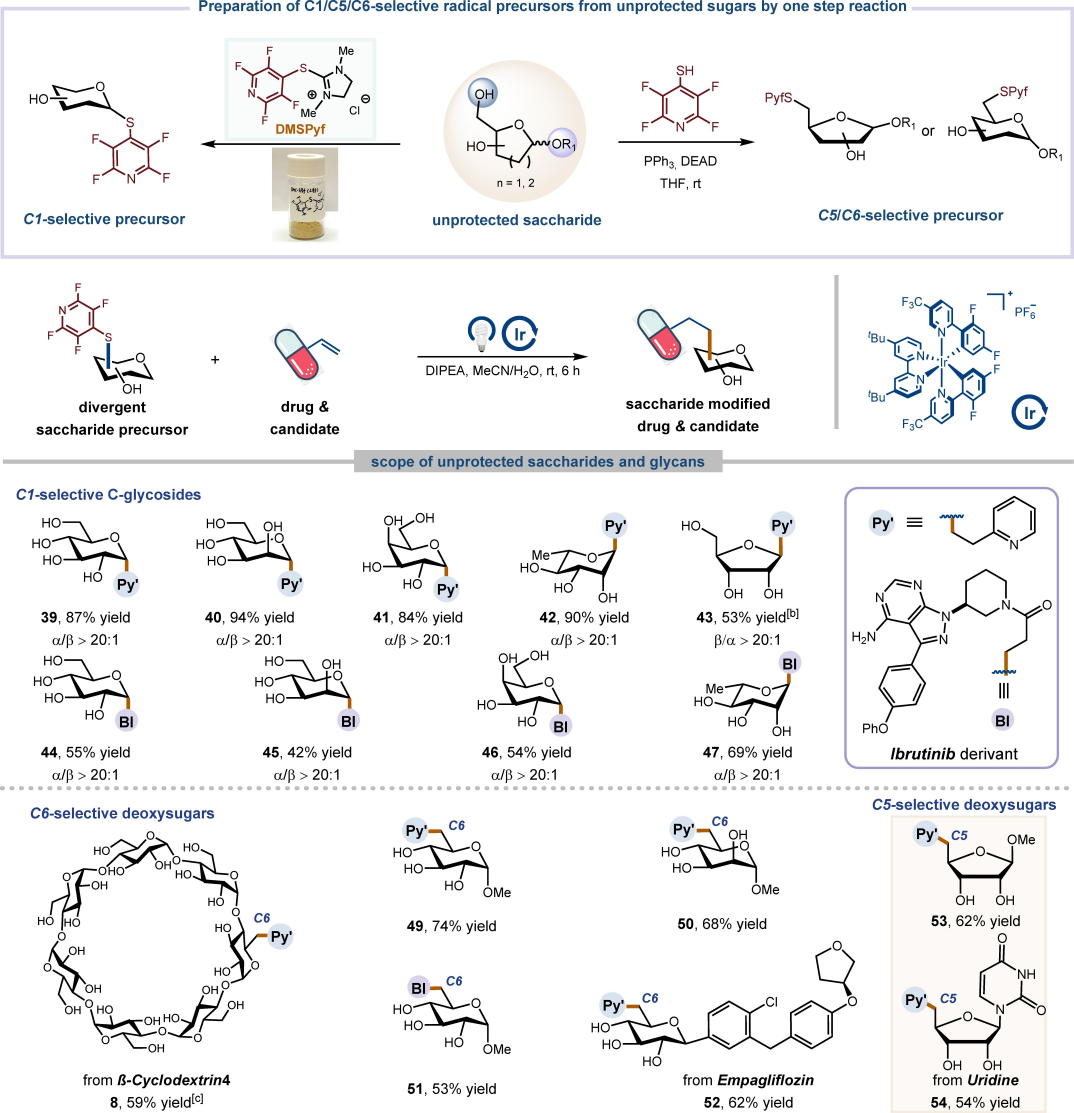
Scope of site‐selective functionalization of unprotected saccharides and glycans.^[a]^

[a] Isolated yields. The anomeric ratios of the isolated and purified products were determined by ^1^H NMR spectroscopic analysis. General reaction conditions: glycosyl precursor (0.1 mmol, 1.0 equiv.), alkene (1.5 equiv.), DIPEA (2.0 equiv.), Ir[dF(CF_3_)ppy]_2_(dtbbpy)PF_6_ (2 mol %) in MeCN/H_2_O=9 : 1 (0.1 M), Kessil LEDs 427 nm (40 W) for 6 h. [b] Synthesis of unprotected saccharide precursor from protecting group removal, see Supporting Information for its preparation. [c] DMF as reaction solvent. See Supporting Information for full experimental details. DEAD=diethyl azodicarboxylate.

Gratifyingly, our two‐step selective functionalization protocol for this photocatalyzed C−S bond cleavage can be extended to C5 and C6 alkylation, enabling the efficient synthesis of unprotected rare saccharides. Consequently, 6‐glucosyl (**49**, **51**, **52**), 6‐mannosyl (**50**) and 5‐furanosyl (**53**, **54**) precursors exhibited similar efficiencies, affording the desired products with moderated yields. Cyclodextrins are cyclic oligosaccharides composed of glucose units with over 20 hydroxyl groups. The selective modification of these compounds can significantly change their properties or introduce new functionalities.^[49]^ We were excited to discover that our protocol can selectively modify a single hydroxyl group in *β*‐cyclodextrin, resulting in the efficient synthesis of the *β*‐cyclodextrin alkylated product **48**.

**Figure 3:**
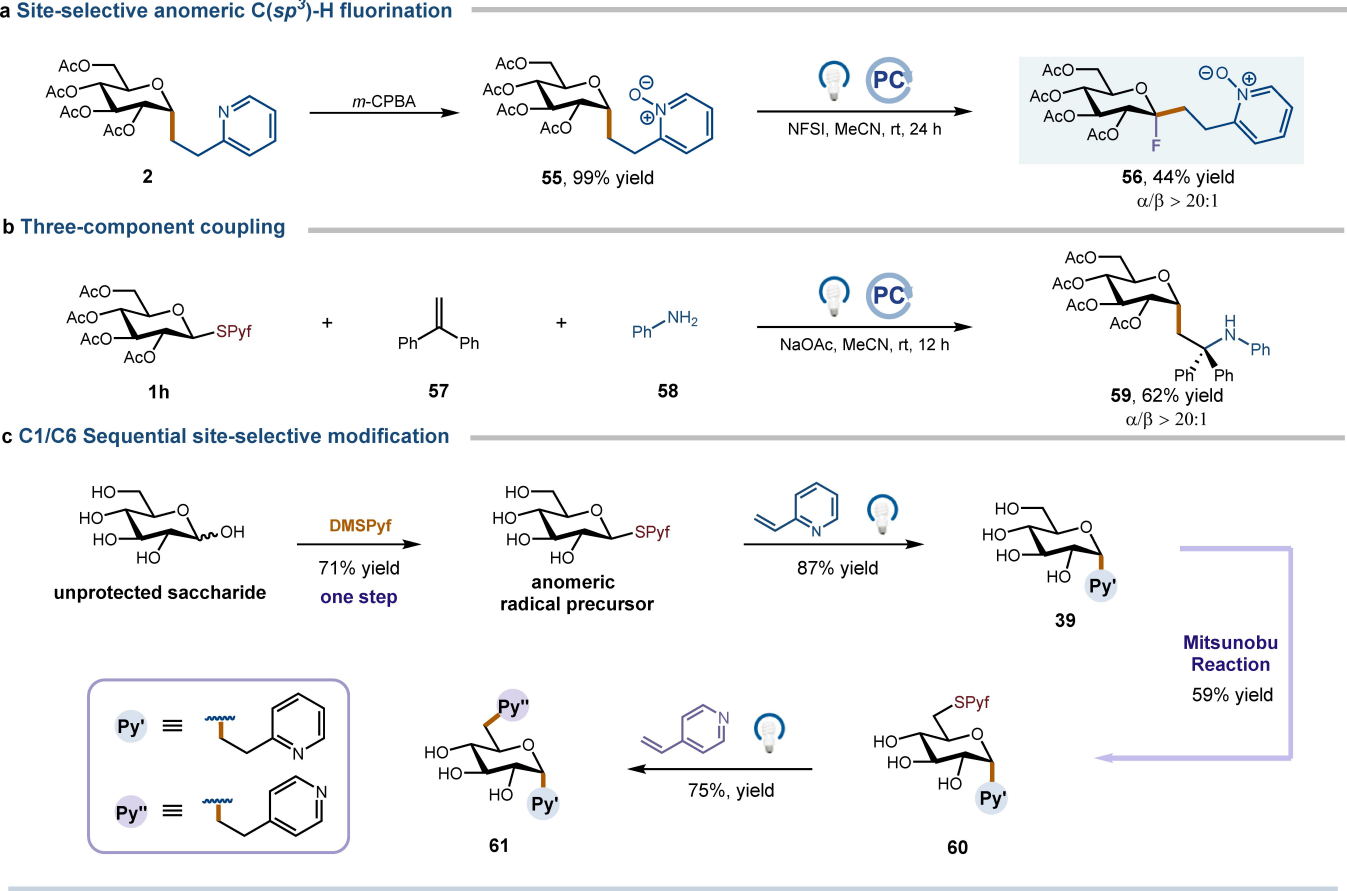
Synthetic applications. Isolated yields. See Supporting Information for full experimental details. *m*‐CPBA=3‐chloroperoxybenzoic acid. NFSI=*N*‐fluorobenzenesulfonimide.

To highlight the potential utility of our method, several synthetic applications were showcased. Glycosyl fluorides are an important class of donors and have been widely used in the chemical synthesis of oligosaccharides. To our delight, treatment of **2** with *m*‐CPBA afforded the pyridine‐*N*‐oxide derivative **55** in 99 % yield, which could undergo anomeric C−H fluorination through a dual HAT/photoredox catalysis event to give a rare quaternary fluorinated saccharide **56** with complete stereocontrol (Figure 3a, see Supporting Information for details). Compound **56** can function as an uncommon glycosyl fluoride capable of undergoing additional glycosylation, leading to a diverse range of quaternary saccharides. It is important to note that accessing this compound using existing synthetic methodologies would be challenging. Furthermore, a three‐component coupling among **1 h**, 1,1‐diphenylethylene **57**, and aniline **58** was conducted to provide the intriguing amination carbohydrate **59** in a stereoselective manner with a 62 % yield (Figure 3b). Lastly, a sequential site‐selective functionalization of unprotected saccharide was demonstrated. As illustrated in Figure 3c, treatment of glucose with our newly developed reagent DMSPyf afforded an anomeric radical precursor, which was then subjected to photocatalysis conditions, resulting in the formation of *C*‐glycoside **39**. Subsequent exposure of **39** to a Mitsunobu reaction led to the generation of a C6‐selective radical precursor **60**, which, upon treatment with a second Giese addition yielded the C1‐ and C6‐functional *C*‐glycoside **61** in just four steps. We anticipate that the ability to conduct regio‐selective and sequential coupling steps with unprotected saccharides will offer significant advantages in terms of the step economy in building complex carbohydrate molecules, by eliminating the need to install and remove related protecting groups.

## Conclusion

In summary, we have established a general platform for the regiodivergent functionalization of carbohydrates, leveraging the electron‐deficient thiyl fragment as a versatile activating group. Our approach enables the efficient generation of carbon radicals across all saccharide sites, permitting the precise carbohydrate manipulation. The site‐selective functionalization of unprotected saccharides and glycans at the C1, C5, and C6 position is accomplished through the direct, single‐step installation of this activating group. We demonstrate that this adaptive activating strategy can be successfully employed in the selective modification of *β*‐cyclodextrin and the sequential site‐selective functionalization of unprotected saccharides, showcasing its applicability to the complex settings.

## Supporting Information

The authors have cited additional references within the Supporting Information.

## Conflict of Interests

The Authors declare no competing interests.

1

## Supporting information

As a service to our authors and readers, this journal provides supporting information supplied by the authors. Such materials are peer reviewed and may be re‐organized for online delivery, but are not copy‐edited or typeset. Technical support issues arising from supporting information (other than missing files) should be addressed to the authors.

Supporting Information

## Data Availability

The data that support the findings of this study are available in the supplementary material of this article.
