# Supplementary material for: Regiodivergent Functionalization of Protected and Unprotected Carbohydrates using Photoactive 4‐Tetrafluoropyridinylthio Fragment as an Adaptive Activating Group
Source: Angew Chem Int Ed Engl. 2024 Oct 24;63(52):e202412436. doi: 10.1002/anie.202412436 (PMC11656145; doi:10.1002/anie.202412436)
Supplement: Supplementary file 1 — Supporting Information [file ANIE-63-e202412436-s001.pdf]

Angewandte Chemie
Eine Zeitschrift der Gesellschaft Deutscher Chemiker

Supporting Information

Regiodivergent Functionalization of Protected and Unprotected Carbohydrates using Photoactive 4-Tetrafluoropyridinylthio Fragment as an Adaptive Activating Group

S. Cao, H. Zhang, M. Chen, N. Zhu, B. Zhan, P. Xu, X. Chen, B. Yu*, X. Zhang**

Regiodivergent Functionalization of Protected and Unprotected Carbohydrates using Photoactive 4-Tetrafluoropyridinylthio Fragment as an Adaptive Activating Group

Shen Cao[†1], Haobo Zhang[†1], Mingshuo Chen[1], Niming Zhu[1], Beibei Zhan[1], Peng Xu[1,2], Xiaoping Chen[1*], Biao Yu[1,2*] and Xiaheng Zhang[1*]

[1]*School of Chemistry and Materials Science, Hangzhou Institute for Advanced Study, University of Chinese Academy of Sciences, 1 Sub-lane Xiangshan, Hangzhou 310024, P. R. China.*

[2]*State Key Laboratory of Chemical Biology, Shanghai Institute of Organic Chemistry, University of Chinese Academy of Sciences, Chinese Academy of Sciences, 345 Lingling Road, Shanghai 200032, China*

[†]*These authors contributed equally to this work.*

*Corresponding author.

Email: xiaopingchen@ucas.ac.cn; byu@sioc.ac.cn; xiahengz@ucas.ac.cn

Supporting Information

Table of Contents

1) General Information...3
2) Synthesis of Radical Precursors 4
3) Reaction Optomization..30
4) Procedures of C-Alkyl Modification with Carbohydrates.......................................33
5) Experimental Data...34
6) Further Application...76
7) References..81
8) Spectral Data...82

1) General Information

Commercial reagents were purchased from Bide Pharmatech CO.,Ltd, Honghu Pharmatech Ltd., Adamas-beta, J&K, TCI, Yuanye Bio-Technology, Panjin Yanfeng Technology. Organic solutions were concentrated under reduced pressure on a Büchi rotary evaporator using a water bath. All reactions were carried out in oven-dried tubes, prepared under nitrogen atmosphere. Chromatographic purification of products was accomplished using forced-flow chromatography on silica gel (Silicycle, 230–400 mesh) according to the method of Still. Thin-layer chromatography (TLC) was performed on Huanghai 0.25 mm silica gel F-254 plates. Visualization of the developed chromatogram was performed by fluorescence quenching, ceric ammonium molybdate (CAM) stain, Phosphomolybdic Acid (PMA) stain or $KMnO_4$ stain. ^1H NMR spectra were recorded on a Bruker UltraShield Plus Avance III 600 MHz or a Bruker UltraShield Plus Avance III 400 MHz and are internally referenced to residual protic $CDCl_3$ (7.26 ppm), CD_3OD (3.31 ppm), DMSO-*d*6 (2.50 ppm). Data for ^1H NMR are reported as follows: chemical shift (δ ppm), multiplicity (s = singlet, d = doublet, t = triplet, q = quartet, m = multiplet, dd = doublet of doublets, dt = doublet of triplets, dq = doublet of quartets), coupling constant (Hz), and integration. ^{13}C NMR spectra were recorded on a Bruker UltraShield Plus Avance III 150 MHz or a Bruker UltraShield Plus Avance III 100 MHz and are internally referenced to residual protic $CDCl_3$ (77.16 ppm), CD_3OD (49.00 ppm), DMSO-*d*6 (39.52 ppm). ^{19}F NMR spectra were recorded on Bruker UltraShield Plus Avance III 400 MHz (376 MHz). High Resolution Mass Spectra were obtained on Thermo Fisher Exactive Plus Orbitrap Mass Spectrum (ESI).

2) Synthesis of Radical Precursors

Most radical precursors are synthesized in one step and can be stored at room temperature over months under dark conditions.

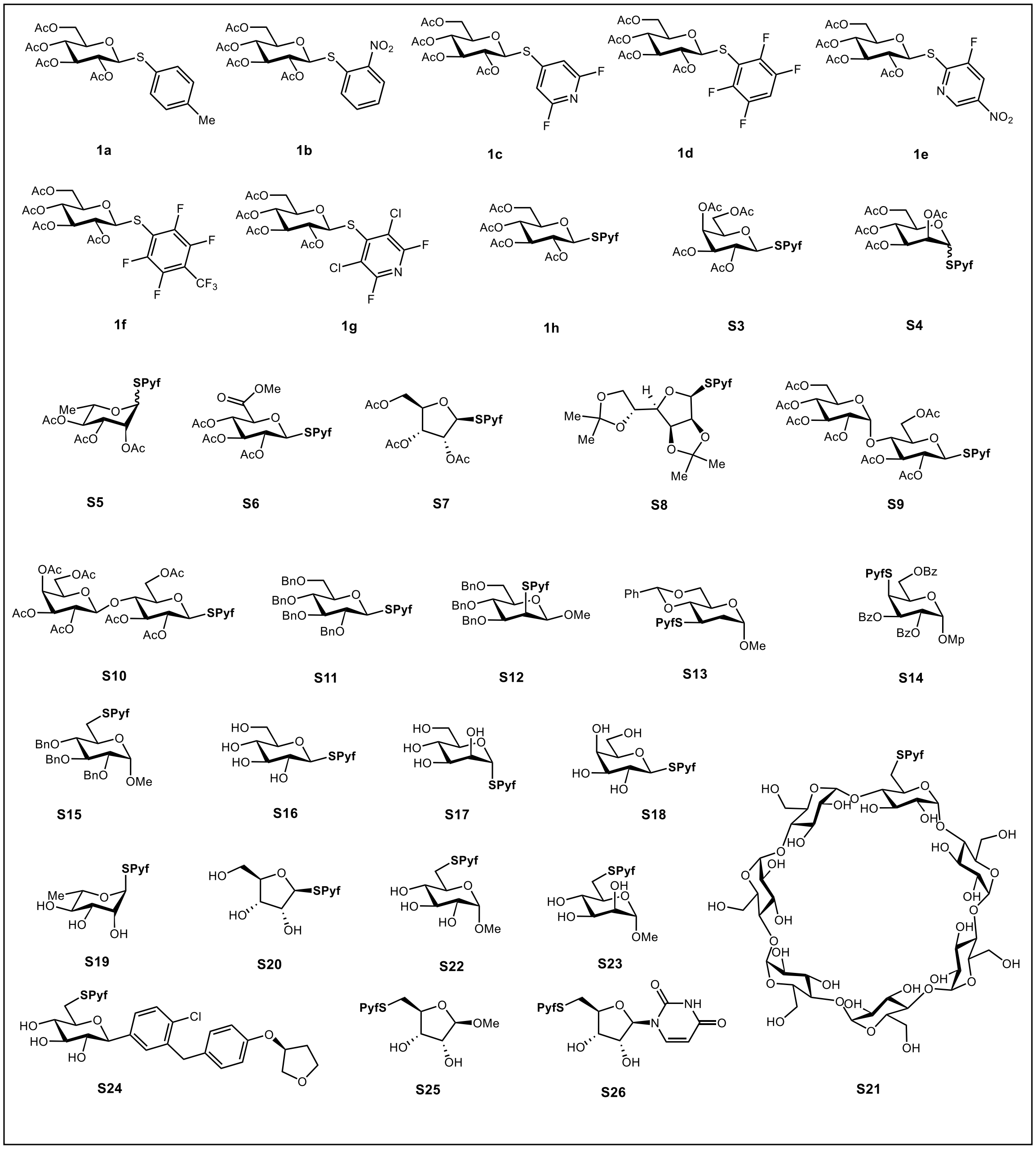

Figure S1. Synthesis of different radical precursors.

1a[1] and **1b**[2] was synthesized according to previous reports.

Synthesis of 2,3,5,6-tetrafluoropyridine-4-thiol (S1)

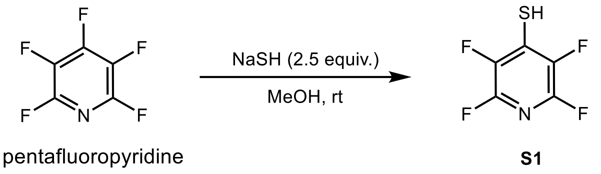

According to literature[3], a round flask equipped with stir bar was added sodium hydrosulfide hydrate (20.7 g, 370 mmol, 2.5 equiv.) in methanol (50 mL), which was then immersed into an ice water bath. The mixture was stirred for 10 mins before pentafluoropyridine (16.2 mL, 147.9 mmol, 1.0 equiv.) was added dropwise. The cold bath was removed and stirred at room temperature for 30 mins. Volatile components were evaporated under reduced pressure, giving a gray yellow solid. Concentrated hydrochloric acid (40 mL) was added to quench the solid residue. The mixture was extracted with petroleum ether (1×40 mL and 2×30 mL). The combined organic layers were washed with brine, dried over Na_2SO_4, filtered, and concentrated in vacuo giving yellow oil in flask. Finally, the residue was distilled under vacuum collecting the fraction boiling at 62-64 °C (32 mbar) to afford 2,3,5,6-tetraflupropyridine-4-thiol (**S1,** 24.1 g, 90%) as a colorless liquid.

Synthesis of dimethylimidazolium 4-tetrafluoropyridinylthio chloride (DMSPyf, S2)

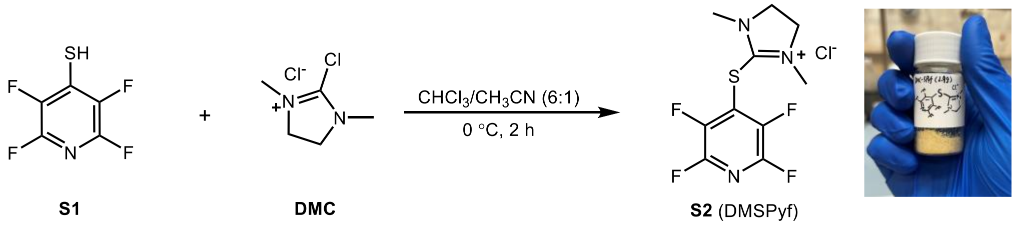

The colorless liquid **S1** (1.9 g, 10.5 mmol, 1.05 equiv.) was dissolved in $CHCl_3$ (30 ml) and acetonitrile (5 ml), then 2-chloro-1,3-dimethylimidazolium chloride (DMC, 1.7 g, 10 mmol, 1.0 equiv.) was added to reaction mixture at 0 °C. The reaction mixture was stirred at 0 °C for 1 h and evaporated in vacuo. The solid was triturated with Et_2O for several hours to give dimethylimidazolium 4-tetrafluoropyridinylthio (DMSPyf) (**S2,** 2.9 g, 92%) as a yellow solid.

^{1}H NMR (400 MHz, $CDCl_3$) δ 4.15 (s, 4H), 3.38 (s, 6H).

^{13}C NMR (100 MHz, $CDCl_3$) δ 145.74 – 144.53 (m), 142.75 – 142.38 (m), 142.36 – 142.25 (m), 140.91 – 139.02 (m), 123.31 – 122.01 (m), 51.08, 36.34.

^{19}F NMR (376 MHz, $CDCl_3$) δ -86.36 – -86.61 (m, 2F), -134.76 – -135.00 (m, 2F).

HRMS (ESI) m/z calcd. for $C_{10}F_4N_3S^+$ ($[M]^+$) 280.0526, found: 280.0522.

General Procedure A:

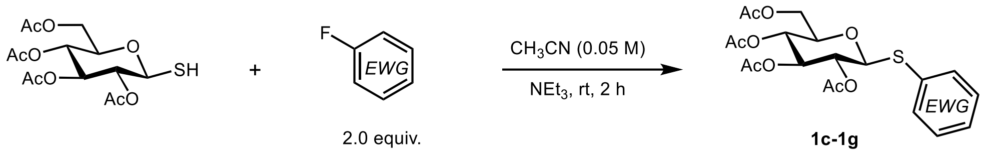

To a Schlenk tube equipped with stir bar was added 2,3,4,6-tetra-O-acetyl-β-D-thiogalactopyranose (728 mg, 2.0 mmol), triethylamine (2.0 equiv.), and dry acetonitrile (30 mL). Then the fluoro-substituted benzene (2.0 equiv.) was added dropwise and stirred for 2 h. The reaction was quenched by water and diluted with DCM, the organic layer was combined, washed by brine, dried over Na_2SO_4, filtered, concentrated *in vacuo* without further purification to give a white solid.

General Procedure B:

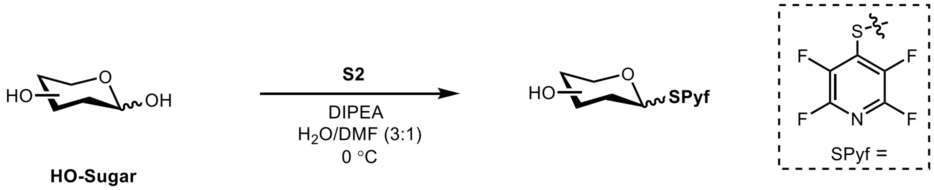

To a solution of unprotected saccharides **HO-sugar** (0.2 mmol, 1.0 equiv.) in H_2O: DMF = 3:1 (0.5 M), DIPEA (2.5 mmol, 5.0 equiv.) and DMSPyf **S2** (1.5 mmol, 3.0 equiv.) were added, then the reaction solution was stirred at 0 ºC for 2 h. The mixture was concentrated in vacuo and the resulting residue was purified by reverse phase column chromatography to afford products.

General Procedure C:

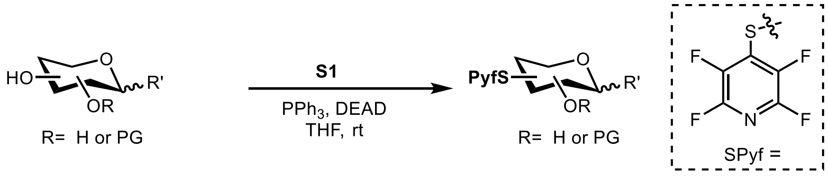

To a solution of unprotected saccharides (1.0 equiv.), 2,3,5,6-tetraflupropyridine-4-thiol (1.5 equiv.) and PPh_3 (2.0 equiv.) in dry THF or DMF (0.1 M), DEAD (1.3 equiv.) was added dropwise at 0 °C under a nitrogen atmosphere over a period of 5 mins. After consumption of the starting material was confirmed by TLC analysis (about 4 h), the

solvent was evaporated under reduced pressure and the resulting oil purified by flash column chromatography to give products.

General Procedure D:

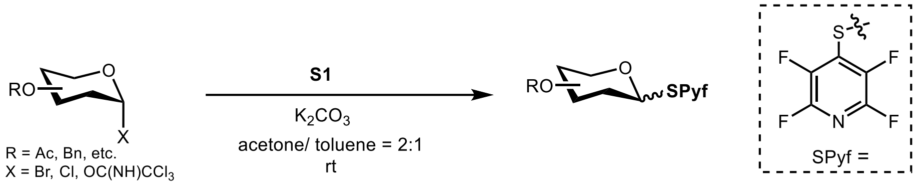

To a solution of glycosyl precursors (5.0 mmol, 1.0 equiv.) in dry acetone:toluene = 2:1 (0.2 M), 2,3,5,6-tetraflupropyridine-4-thiol (10.0 mmol, 2 equiv.) and K_2CO_3 (2.0 equiv.) were added, the mixture was stirred for 10 mins. After the complete consumption of glycosyl acetate monitored by TLC analysis, the reaction mixture was filtered and concentrated in vacuo. The resulting residue was purified by silica gel flash chromatography to give the thioglycoside donors as colorless oil.

(3,5-Difluoropyridin-4-yl)-2,3,4,6-tetra-O-acetyl-β-D-thioglucopyranoside (1c)

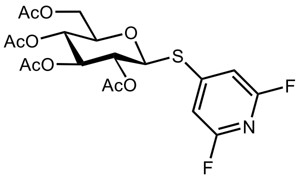

Prepared following the **general procedure A** outlined above starting from 2,3,4,6-Tetra-O-acetyl-β-D-thiogalactopyranose on 2.0 mmol scale to give **1c** (915.8 mg, 96%, β only) as a white solid.

^{1}H NMR (400 MHz, CDCl$_3$) δ 6.82 (s, 2H), 5.29 (t, *J* = 9.3 Hz, 1H), 5.14 – 5.09 (m, 1H), 5.09 – 5.03 (m, 1H), 4.98 (d, *J* = 10.1 Hz, 1H), 4.21 – 4.17 (m, 2H), 3.89 (ddd, *J* = 2.5, 6.3, 10.2 Hz, 1H), 2.10 (s, 3H), 2.05 (s, 3H), 2.03 (s, 3H), 2.00 (s, 3H).

^{13}C NMR (100 MHz, CDCl$_3$) δ 170.64, 170.08, 169.47, 169.34, 162.95 (d, *J* = 16.6 Hz), 160.50 (d, *J* = 16.6 Hz), 154.90 (t, *J* = 8.4 Hz), 105.02, 104.87, 104.73, 104.59, 83.29, 76.38, 73.43, 69.44, 68.10, 62.36, 20.65, 20.62, 20.61.

^{19}F NMR (376 MHz, $CDCl_3$) δ -67.89 (s, 2F).

HRMS (ESI) m/z calcd. for $C_{19}H_{21}F_2NO_9S$ ($[M+H]^+$) 478.0983, found: 478.0977.

(2,3,5,6-Tetrafluoro)phenyl-2,3,4,6-tetra-O-acetyl-β-D-thioglucopyranoside (1d)

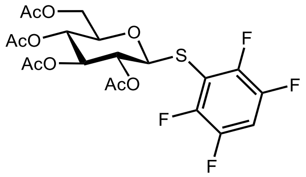

Prepared following the **general procedure A** outlined above starting from 2,3,4,6-tetra-O-acetyl-β-D-thiogalactopyranose on 2.0 mmol scale to give **1d** (911.4 mg, 89%, β only) as a white solid.

^{1}H NMR (400 MHz, $CDCl_3$) δ 7.15 (dd, *J* = 7.2, 9.5 Hz, 1H), 5.19 (t, *J* = 9.3 Hz, 1H), 5.05 (t, *J* = 9.8 Hz, 1H), 4.97 (t, *J* = 9.6 Hz, 1H), 4.75 (d, *J* = 10.1 Hz, 1H), 4.17 (dd, *J* = 5.3, 12.4 Hz, 1H), 4.08 (dd, *J* = 2.5, 12.4 Hz, 1H), 3.65 (ddd, *J* = 2.5, 5.3, 10.0 Hz, 1H), 2.08 (s, 3H), 2.02 (s, 3H), 2.00 (s, 3H), 1.98 (s, 3H).

^{13}C NMR (100 MHz, $CDCl_3$) δ 170.63, 170.21, 169.49, 169.41, 148.74 – 148.48 (m), 147.52 – 147.15 (m), 146.30 – 146.00 (m), 145.03 – 144.64 (m), 111.02 -111.34 (m), 107.65 -107.99 (m), 85.10, 76.20, 73.84, 70.70, 68.15, 62.00, 20.65, 20.64, 20.62, 20.60.

^{19}F NMR (376 MHz, $CDCl_3$) δ -131.41 – -131.61 (m, 2F), -137.31 – -137.50 (m, 2F).

HRMS (ESI) m/z calcd. for $C_{21}H_{20}F_4O_9S$ ($[M+Na]^+$) 535.0662, found: 535.0657.

(6-Fluoro-4-nitropyridin-2-yl)-2,3,4,6-tetra-O-acetyl-β-D-thioglucopyranoside (1e)

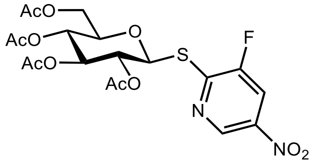

Prepared following the **general procedure A** outlined above starting from 2,3,4,6-tetra-O-acetyl-β-D-thiogalactopyranose on 2.0 mmol scale to give **1e** (646.4 mg, 64%, β only) as a white solid.

^{1}H NMR (400 MHz, CDCl$_3$) δ 9.14 (d, *J* = 2.1 Hz, 1H), 8.08 (dd, *J* = 2.2, 8.4 Hz, 1H), 5.94 (d, *J* = 10.3 Hz, 1H), 5.37 (t, *J* = 9.2 Hz, 1H), 5.29 (dd, *J* = 9.2, 10.4 Hz, 1H), 5.17 (dd, *J* = 9.2, 10.1 Hz, 1H), 4.25 (dd, *J* = 4.5, 12.5 Hz, 1H), 4.09 (dd, *J* = 2.3, 12.5 Hz, 1H), 3.91-3.87 (m, 1H), 2.04 (s, 3H), 2.03 (s, 3H), 2.02 (s, 3H), 2.02 (s, 3H).

^{13}C NMR (100 MHz, CDCl$_3$) δ 170.66, 170.21, 169.49, 155.95, 153.29 (d, *J* = 7.4 Hz), 142.23, 140.36 (d, *J* = 4.8 Hz), 116.96 (d, *J* = 21.9 Hz), 79.76, 76.30, 73.99, 69.06, 68.00, 61.75, 20.77, 20.66.

^{19}F NMR (376 MHz, CDCl$_3$) δ -117.43 (d, *J* = 8.6 Hz, 1F).

HRMS (ESI) m/z calcd. for $C_{19}H_{21}FN_2O_{11}S$ ($[M+Na]^+$) 527.0748, found: 527.0743.

((2,3,5,6-Tetrafluoro-4-(trifluoromethyl))phenyl 2,3,4,6-tetra-O-acetyl-β-D-thioglucopyranoside (1f)

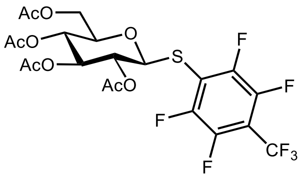

Prepared following the **general procedure A** outlined above starting from 2,3,4,6-tetra-O-acetyl-β-D-thiogalactopyranose on 2.0 mmol scale to give **1f** (1.0 g, 90%, β only) as a white solid.

^{1}H NMR (400 MHz, CDCl$_3$) δ 5.22 (t, *J* = 9.3 Hz, 1H), 5.11 – 5.04 (m, 1H), 5.01 (d, *J* = 9.3 Hz, 1H), 4.90 (d, *J* = 10.0 Hz, 1H), 4.13 – 4.09 (m, 2H), 3.68 (ddd, *J* = 2.5, 5.3, 10.1 Hz, 1H), 2.10 (s, 3H), 2.03 (s, 3H), 2.02 (s, 3H), 2.01 (s, 3H).

^{13}C NMR (100 MHz, $CDCl_3$) δ 170.52, 170.13, 169.42, 169.38, 148.82 – 148.48 (m), 146.30 – 145.97 (m), 145.65 – 145.28 (m), 143.05 – 142.64 (m), 122.41 – 118.88 (m), 116.44 – 115.46 (m), 110.93 – 110.32 (m), 83.98, 76.31, 73.63, 70.71, 67.97, 61.88, 20.58, 20.57, 20.55, 20.48.

^{19}F NMR (376 MHz, $CDCl_3$) δ -56.41 (t, *J* = 21.8 Hz, 3F), -129.93 (q, *J* = 9.6 Hz, 2F), -139.38 (dq, *J* = 10.6, 21.7 Hz, 2F).

HRMS (ESI) m/z calcd. for $C_{21}H_{19}F_7O_9S$ ($[M+Na]^+$) 603.0536, found: 603.0532.

(2,6-Dichloro-3,5-difluoropyridin-4-yl)-2,3,4,6-tetra-O-acetyl-β-D-thioglu-copyranoside (1g)

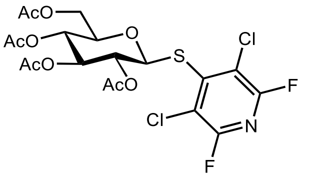

Prepared following the **general procedure A** outlined above starting from 2,3,4,6-tetra-O-acetyl-β-D-thiogalactopyranose on 2.0 mmol scale to give **1g** (991.9 mg, 91%, β only) as a white solid.

^{1}H NMR (400 MHz, $CDCl_3$) δ 5.29 – 5.20 (m, 1H), 5.20 – 5.14 (m, 1H), 5.07 (t, *J* = 9.5 Hz, 1H), 4.98 (d, *J* = 9.5 Hz, 1H), 4.20 – 4.12 (m, 1H), 4.04 (dd, *J* = 2.4, 12.4 Hz, 1H), 3.63 (ddd, *J* = 2.4, 5.8, 10.0 Hz, 1H), 2.09 (s, 3H), 2.02 (s, 3H), 2.02 (s, 6H).

^{13}C NMR (100 MHz, $CDCl_3$) δ 170.53, 170.21, 169.44, 156.17 (d, *J* = 15.3 Hz), 153.70 (d, *J* = 14.9 Hz), 148.37, 84.69, 76.36, 73.58, 70.99, 68.06, 62.02, 20.75, 20.66, 20.64, 20.64.

^{19}F NMR (376 MHz, $CDCl_3$) δ -68.39. (s, 2F).

HRMS (ESI) m/z calcd. for $C_{19}H_{19}Cl_2F_2NO_9S$ ($[M+H]^+$) 546.0204, found: 546.0199.

(Perfluoropyridin-4-yl)-2,3,4,6-tetra-O-acetyl-β-D-thioglucopyranoside (1h)

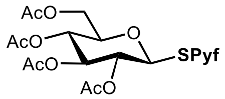

Prepared following the **general procedure D** outlined above starting from (2R,3R,4S,5R,6R)-2-(acetoxymethyl)-6-bromotetrahydro-2H-pyran-3,4,5-triyl triacetate on 5.0 mmol scale to give **1h** (2.3 g, 91%, β/α > 20:1) as a white foam.

^{1}H NMR (400 MHz, CDCl3) δ 5.26 (t, *J* = 9.2 Hz, 1H), 5.19 (d, *J* = 10.0 Hz, 1H), 5.08 (t, *J* = 9.7 Hz, 2H), 4.19 – 4.05 (m, 2H), 3.74 (ddd, *J* = 10.1, 5.2, 2.6 Hz, 1H), 2.09 (s, 3H), 2.03 (s, 3H), 2.03 (s, 3H), 2.01 (s, 3H).

^{13}C NMR (100 MHz, $CDCl_3$) δ 170.59, 170.16, 169.44, 169.41, 145.22 – 144.05 (m), 142.90 – 142.52 (m), 142.54 – 142.30 (m), 140.88 – 139.63 (m), 127.42 – 126.67 (m), 82.40, 73.60, 70.82, 67.97, 61.89, 20.65, 20.62, 1.14.

^{19}F NMR (376 MHz, $CDCl_3$) δ -89.62 – -90.14 (m, 2F), -136.41 – -136.71 (m, 2F).

HRMS (ESI) m/z calcd. for $C_{19}H_{19}F_4NO_9S$ ($[M+Na]^+$) 536.0615, found: 536.0605.

(Perfluoropyridin-4-yl)-2,3,4,6-tetra-O-acetyl-β-D-thiogalactopyranoside (S3)

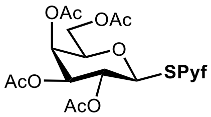

Prepared following the **general procedure D** outlined above starting from (2R,3R,4S,5R,6R)-2-(acetoxymethyl)-6-bromotetrahydro-2H-pyran-3,4,5-triyl triacetate on 5.0 mmol scale to give **S3** (1.52 g, 91%, β/α > 20:1) as a white foam.

^{1}H NMR (400 MHz, $CDCl_3$) δ 5.42 (d, *J* = 3.1 Hz, 1H), 5.29 (dd, *J* = 9.9, 9.9 Hz, 1H), 5.15 (d, *J* = 9.9 Hz, 1H), 5.08 (dd, *J* = 9.8, 3.4 Hz, 1H), 4.12 – 4.00 (m, 2H), 3.95 (dd, 1H), 2.15 (s, 3H), 2.09 (s, 3H), 1.98 (s, 3H), 1.98 (s, 3H).

^{13}C NMR (100 MHz, CDCl$_3$) δ 170.40, 170.17, 169.93, 169.58, 145.19 – 144.51 (m), 142.62 – 142.45 (m), 140.46 – 140.15 (m), 140.10 – 139.81 (m), 127.34 – 127.07 (m), 83.24, 83.20, 83.16, 75.33, 71.64, 68.04, 67.14, 61.57, 20.67, 20.65, 20.57, 20.50.

^{19}F NMR (376 MHz, CDCl$_3$) δ -89.96 – -90.22 (m, 2F), -136.39 – -136.69 (m, 2F).

HRMS (ESI) m/z calcd. for $C_{19}H_{19}F_4NO_9S$ ($[M+Na]^+$) 536.0615, found: 536.0613.

(Perfluoropyridin-4-yl)-2,3,4,6-tetra-O-acetyl-α/β-D-thiomannopyranoside (S4)

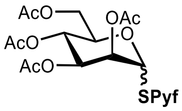

Prepared following the **general procedure D** outlined above starting from ((2R,3R,4S,5R,6R)-2-(acetoxymethyl)-6-bromotetrahydro-2H-pyran-3,4,5-triyl triacetate on 5.0 mmol scale to give the mixture of α/β isomer **S4** (1.52 g, 91%, α/β = 1.37:1) as a white foam.

^{1}H NMR (400 MHz, CDCl$_3$) δ 5.75 (d, *J* = 1.5 Hz, 0.57H, ***α*-H1**), 5.59 (d, *J* = 3.4 Hz, 0.44 H), 5.50 (dd, *J* = 1.5, 3.4 Hz, 0.60H), 5.44 (d, *J* = 1.2 Hz, 0.42H, ***β*-H1**), 5.35 (dd, *J* = 3.4, 9.9 Hz, 0.60H), 5.27 (d, *J* = 9.9 Hz, 0.46H), 5.25 – 5.19 (m, 0.54H), 5.09 (dd, *J* = 3.4, 10.1 Hz, 0.43H), 4.31 (ddd, *J* = 2.2, 6.4, 9.5 Hz, 0.60H), 4.20 – 4.06 (m, 1.49H), 4.00 (dd, *J* = 2.3, 12.4 Hz, 0.58H), 3.72 (ddd, *J* = 2.5, 6.4, 9.6 Hz, 0.40H), 2.23 (s, 1.20H), 2.16 (s, 1.77H), 2.07 (s, 1.73H), 2.03 (s, 1.21H), 2.01 (s, 1.72H), 2.00 (s, 1.24H), 1.98 (s, 1.28H), 1.95 (s, 1.65H).

^{13}C NMR (100 MHz, CDCl$_3$) δ 170.52, 170.35, 170.11, 169.94, 169.82, 169.66, 169.63, 169.60, 145.11 – 144.73 (m), 145.02 – 144.53 (m), 143.94 – 143.63 (m), 142.86 – 142.08 (m), 142.44 – 141.86 (m), 141.36 – 141.21 (m), 141.38 – 141.03 (m), 139.79 – 139.58 (m), 127.32 – 127.10 (m), 125.38 – 124.98 (m), 83.33, 83.30, 83.27, 80.74, 80.70, 71.54, 70.92, 69.60, 69.48, 68.87, 65.76, 65.37, 62.42, 62.23, 20.73, 20.65, 20.64, 20.55, 20.53, 20.51, 20.49, 20.37.

^{19}F NMR (376 MHz, $CDCl_3$) δ -88.96 – -89.29 (m, 1.24F), -89.95 – -90.21 (m, 0.77F), -134.26 – -134.69 (m, 1.31F), -137.00 – -137.26 (m, 0.71F).

HRMS (ESI) m/z calcd. for $C_{19}H_{19}F_4NO_9S$ ($[M+Na]^+$) 536.0615, found: 536.0613.

(Perfluoropyridin-4-yl)-2,3,4-tri-O-acetyl-α/β-L-thiorhamnopyranoside (S5)

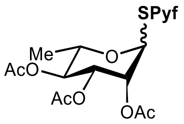

Prepared following the **general procedure D** outlined above starting from (3R,4R,5S,6S)-2-bromo-6-methyltetrahydro-2H-pyran-3,4,5-triyl triacetate on 5.0 mmol scale to give the mixture of α/β isomer **S5** (1.52 g, 91%, α/β = 1.79:1) as a white foam.

^{1}H NMR (400 MHz, $CDCl_3$) δ 5.70 (d, *J* = 1.5 Hz, 0.65H, ***α*-H1**), 5.60 (dd, *J* = 1.2, 3.2 Hz, 0.36H), 5.50 (dd, *J* = 1.6, 3.5 Hz, 0.66H), 5.42 (d, *J* = 1.2 Hz, 0.36H,), 5.31 (dd, *J* = 3.5, 10.1 Hz, 0.67H), 5.14 (d, *J* = 9.9 Hz, 0.67H), 5.11 – 5.06 (m, 0.37H), 5.06 – 5.01 (m, 0.38H), 4.29 – 4.17 (m, 0.68H), 3.65 – 3.51 (m, 0.37H), 2.24 (s, 1.11H), 2.16 (s, 1.94H), 2.07 (s, 2.00H), 2.04 (s, 0.97H), 2.01 (s, 1.94H), 1.98 (s, 1.04H), 1.24 (d, *J* = 6.1 Hz, 1.08H), 1.15 (d, *J* = 6.2 Hz, 1.96H).

^{13}C NMR (100 MHz, $CDCl_3$) δ 170.17, 170.09, 169.94, 169.85, 169.78, 145.09 – 144.50 (m), 144.83 – 144.42 (m), 144.16 – 143.84 (m), 143.84 – 143.48 (m), 142.33 – 142.01 (m), 141.58 – 141.28 (m), 141.28 – 140.87 (m), 140.31 – 139.90 (m), 126.13 – 125.92 (m), 125.89 – 125.37 (m), 83.71, 83.68, 83.65, 80.82, 80.78, 75.59, 71.54, 70.38, 70.08, 69.96, 69.77, 69.02, 68.91, 20.79, 20.76, 20.74, 20.61, 20.57, 20.56, 17.55, 17.20.

^{19}F NMR (376 MHz, $CDCl_3$) δ -89.15 – -89.40 (m, 1.30F), -89.85 – -90.12 (m, 0.70F), -134.82 – -135.18 (m, 1.28F), -136.77 – -137.12 (m, 0.69F).

HRMS (ESI) m/z calcd. for $C_{17}H_{17}F_4NO_7S$ ($[M+Na]^+$) 478.0560, found: 478.0559.

Methyl (perfluoropyridin-4-yl)-2,3,4-tri-O-acetyl-β-D-thioglucuronate (S6)

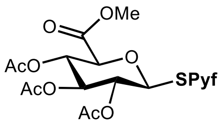

Prepared following the **general procedure D** outlined above starting from (2S,3R,4S,5S,6S)-2-bromo-6-(methoxycarbonyl)tetrahydro-2H-pyran-3,4,5-triyl triacetate on 5.0 mmol scale to give **S6** (1.52 g, 91%, β/α > 20:1) as a white foam.

^{1}H NMR (400 MHz, CDCl$_3$) δ 5.33 – 5.28 (m, 1H), 5.27 – 5.21 (m, 1H), 5.20 (d, *J* = 7.8 Hz, 1H), 5.09 (t, *J* = 9.4 Hz, 1H), 4.04 (d, *J* = 9.7 Hz, 1H), 3.73 (s, 3H), 2.09 (s, 3H), 2.03 (s, 3H), 2.02 (s, 3H).

^{13}C NMR (100 MHz, CDCl$_3$) δ 170.04, 169.35, 169.32, 166.31, 145.39 – 144.53 (m), 143.07 – 142.88 (m), 142.74 – 142.42 (m), 140.65 – 139.86 (m), 126.81 – 126.00 (m), 82.93, 76.32, 72.83, 70.46, 69.00, 53.20, 20.65, 20.60, 20.55.

^{19}F NMR (376 MHz, CDCl$_3$) δ -89.50 – -89.71 (m, 2F), -135.93 – -136.15 (m, 2F).

HRMS (ESI) m/z calcd. for $C_{18}H_{17}F_4NO_9S$ ($[M+Na]^+$) 522.0458, found: 522.0453.

(Perfluoropyridin-4-yl)-2,3,5-tri-O-acetyl-β-D-thioribofuranoside (S7)

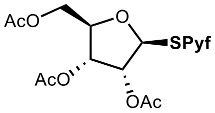

Prepared following the **general procedure D** outlined above starting from (2R,3R,4R)-2-(acetoxymethyl)-5-bromotetrahydrofuran-3,4-diyl diacetate on 5.0 mmol scale to give **S7** (1.52 g, 91%, β/α > 20:1) as a white foam.

^{1}H NMR (400 MHz, CDCl$_3$) δ 5.83 (d, *J* = 3.0 Hz, 1H), 5.49 – 5.46 (m, 1H), 5.44 (dd, *J* = 5.1, 3.0 Hz, 1H), 4.38 (dd, *J* = 5.9, 3.7 Hz, 1H), 4.33 (dd, *J* = 12.3, 3.3 Hz, 1H), 4.18 – 4.13 (m, 1H), 2.15 (s, 3H), 2.12 (s, 6H).

^{13}C NMR (100 MHz, CDCl$_3$) δ 170.53, 169.74, 169.50, 144.96 – 144.58 (m), 143.22 – 143.04 (m), 142.58 – 142.41 (m), 142.60 – 142.12 (m), 126.96 – 126.80 (m), 86.33, 86.29, 80.91, 75.08, 70.97, 62.80, 20.67, 20.49.

^{19}F NMR (376 MHz, CDCl$_3$) δ -89.85 – -90.10 (m, 2F), -135.49 – -135.70 (m, 2F).

HRMS (ESI) m/z calcd. for $C_{16}H_{15}F_4NO_7S$ ([M+Na]$^+$) 464.0403, found: 464.0398.

4-(((3aS,4S,6R,6aS)-6-((R)-2,2-dimethyl-1,3-dioxolan-4-yl)-2,2-dimethyltetrahydrofuro[3,4-d][1,3]dioxol-4-yl)thio)-2,3,5,6-tetrafluoropyridine (S8)

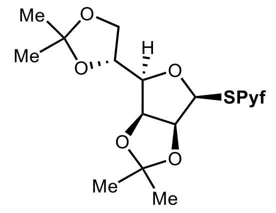

Prepared following the **general procedure D** outlined above starting from (3aS,4R,6R,6aS)-6-((R)-2,2-dimethyl-1,3-dioxolan-4-yl)-2,2-dimethyltetrahydrofuro[3,4-d][1,3]dioxol-4-yl 2,2,2-trichloroacetimidate on 5.0 mmol scale to give **S8** (1.52 g, 91%, β/α $>$ 20:1) as a white foam.

^{1}H NMR (500 MHz, CDCl$_3$) δ 5.87 (s, 1H), 4.88 (dd, *J* = 6.1, 3.4 Hz, 1H), 4.76 (d, *J* = 5.9 Hz, 1H), 4.39 (dd, *J* = 6.8, 4.7 Hz, 1H), 4.17 (dd, *J* = 7.3, 3.4 Hz, 1H), 4.00 (dd, *J* = 8.8, 6.4 Hz, 1H), 3.75 (dd, *J* = 8.8, 4.5 Hz, 1H), 1.47 (s, 3H), 1.37 (s, 3H), 1.35 (s, 6H).

^{13}C NMR (100 MHz, CDCl$_3$) δ 144.78 – 144.40 (m), 144.03 – 143.74 (m), 142.62 – 142.35 (m), 141.45 – 141.19 (m), 127.13 – 126.74 (m), 113.79, 109.51, 90.44, 90.41, 85.56, 81.66, 79.71, 72.47, 66.64, 26.69, 25.99, 25.24, 24.76.

^{19}F NMR (471 MHz, CDCl$_3$) δ -89.92 – -90.13 (m, 2F), -135.12 – -135.33 (m, 2F).

HRMS (ESI) m/z calcd. for $C_{17}H_{19}F_4NO_5S$ ([M+H]$^+$) 426.0998, found: 426.0989.

(Perfluoropyridin-4-yl)-hepta-O-acetyl-β-D-thiomaltopyranoside (S9)

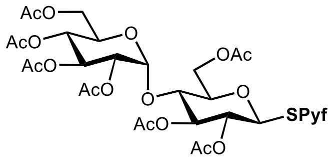

Prepared following the **general procedure D** outlined above starting from (2R,3R,4S,5R,6R)-2-(acetoxymethyl)-6-(((2R,3R,4S,5R,6R)-4,5-diacetoxy-2-(acetoxymethyl)-6-bromotetrahydro-2H-pyran-3-yl)oxy)tetrahydro-2H-pyran-3,4,5-triyl triacetate on 5.0 mmol scale to give **S9** (1.52 g, 91%, β/α > 20:1) as a white foam.

^{1}H NMR (400 MHz, CDCl$_3$) δ 5.39 (d, *J* = 4.0 Hz, 1H), 5.36 – 5.24 (m, 2H), 5.20 (d, *J* = 10.0 Hz, 1H), 5.04 (t, *J* = 9.9 Hz, 1H), 4.92 (t, *J* = 9.5 Hz, 1H), 4.85 (dd, *J* = 10.6, 4.0 Hz, 1H), 4.45 (d, *J* = 12.2 Hz, 1H), 4.24 (dd, *J* = 12.5, 4.1 Hz, 1H), 4.13 (dd, *J* = 12.4, 5.2 Hz, 1H), 4.05 (d, *J* = 12.4 Hz, 1H), 3.97 – 3.88 (m, 2H), 3.73 (d, *J* = 9.0 Hz, 1H), 2.09 (s, 3H), 2.08 (s, 3H), 2.07 (s, 3H), 2.04 (s, 3H), 2.02 (s, 6H), 2.00 (s, 3H).

^{13}C NMR (100 MHz, CDCl$_3$) δ 170.57, 170.54, 170.29, 170.14, 169.94, 169.57, 169.47, 145.05 – 144.44 (m), 143.10 – 142.81 (m), 142.73 – 142.47 (m), 140.16 – 139.90 (m), 126.65 – 126.23 (m), 95.85, 81.89, 76.00, 72.55, 71.53, 70.04, 69.27, 68.74, 67.98, 62.52, 61.55, 20.89, 20.69, 20.61, 20.58, 20.50.

^{19}F NMR (376 MHz, CDCl$_3$) δ -89.63 – -90.01 (m, 2F), -135.92 – -136.36 (m, 2F).

HRMS (ESI) m/z calcd. for $C_{31}H_{35}F_4NO_{17}S$ ($[M+Na]^+$) 824.1460, found: 824.1464.

(Perfluoropyridin-4-yl)-hepta-O-acetyl-β-D-thiolactopyranoside (S10)

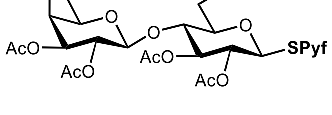

Prepared following the **general procedure D** outlined above starting from (2R,3S,4S,5R,6S)-2-(acetoxymethyl)-6-(((2R,3R,4S,5R,6R)-4,5-diacetoxy-2-

(acetoxymethyl)-6-bromotetrahydro-2H-pyran-3-yl)oxy)tetrahydro-2H-pyran-3,4,5-triyl triacetate on 5.0 mmol scale to give **S10** (1.52 g, 91%, β/α = 18.2:1) as white foam.

^{1}H NMR (400 MHz, CDCl$_3$) δ 5.34 (dd, *J* = 3.5, 1.2 Hz, 1H), 5.24 (t, *J* = 9.1 Hz, 1H), 5.15 (d, *J* = 10.0 Hz, 1H), 5.10 (dd, *J* = 10.4, 7.8 Hz, 1H), 5.04 – 4.97 (m, 1H), 4.95 (dd, *J* = 10.4, 3.4 Hz, 1H), 4.53 – 4.45 (m, 1H), 4.49 – 4.42 (m, 1H), 4.10 (dd, *J* = 11.1, 6.8 Hz, 2H), 4.02 (dd, *J* = 12.1, 6.0 Hz, 1H), 3.87 (dd, *J* = 6.8, 1.3 Hz, 1H), 3.79 (dd, *J* = 10.0, 9.0 Hz, 1H), 3.65 (ddd, *J* = 10.0, 5.9, 2.1 Hz, 1H), 2.15 (s, 3H), 2.09 (s, 3H), 2.06 (s, 3H), 2.05 (s, 3H), 2.05 (s, 3H), 2.04 (s, 3H), 1.96 (s, 3H).

^{13}C NMR (100 MHz, CDCl$_3$) δ 170.46, 170.26, 170.22, 170.15, 169.71, 169.63, 169.17, 142.99 – 142.67 (m), 142.65 – 142.31 (m), 140.44 – 140.12 (m), 140.12 – 139.69 (m), 126.98 – 126.62 (m), 101.16, 82.03, 81.98, 75.90, 73.48, 71.08, 70.96, 70.86, 69.07, 66.66, 61.83, 60.92, 20.79, 20.72, 20.68, 20.62, 20.58.

^{19}F NMR (376 MHz, CDCl$_3$) δ -89.73 – -90.00 (m, 2F), -136.25 – -136.53 (m, 2F).

HRMS (ESI) m/z calcd. for $C_{31}H_{35}F_4NO_{17}S$ ($[M+Na]^+$) 824.1460, found: 824.1462.

(Perfluoropyridin-4-yl)-2,3,4,6-tetra-O-benzyl-β-D-thioglucopyranoside (S11)

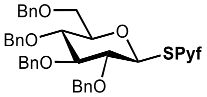

Prepared following the **general procedure D** outlined above starting from (2R,3R,4S,5R,6S)-3,4,5-tris(benzyloxy)-2-((benzyloxy)methyl)-6-chlorotetrahydro-2H-pyran on 5.0 mmol scale to give **S11** (1.52 g, 91%, β/α ＞ 20:1) as a white foam.

^{1}H NMR (400 MHz, CDCl$_3$) δ 7.24 (dd, *J* = 18.0, 2.0 Hz, 15H), 7.19 – 7.15 (m, 3H), 7.13 – 7.08 (m, 2H), 5.11 (d, *J* = 9.6 Hz, 1H), 4.83 (d, *J* = 9.6 Hz, 3H), 4.79 – 4.73 (m, 2H), 4.52 (d, *J* = 10.8 Hz, 1H), 4.45 (d, *J* = 12.2 Hz, 1H), 4.37 (d, *J* = 12.2 Hz, 1H), 3.71 – 3.62 (m, 2H), 3.54 (ddd, *J* = 13.9, 5.3, 3.5 Hz, 3H), 3.43 – 3.35 (m, 1H).

^{13}C NMR (100 MHz, CDCl$_3$) δ 144.92 – 144.21 (m), 142.47 – 142.12 (m), 142.48 – 141.76 (m), 140.23 – 139.93 (m), 139.80 – 139.64 (m), 138.15, 137.87, 137.80, 137.30, 128.55, 128.49, 128.39, 128.18, 128.13, 127.92, 127.87, 127.76, 127.65, 86.57, 83.06, 82.02, 79.53, 75.85, 75.74, 75.10, 73.30, 68.22.

^{19}F NMR (376 MHz, CDCl$_3$) δ -90.90 – -91.19 (m, 2F), -136.99 – -137.23 (m, 2F).

HRMS (ESI) m/z calcd. for $C_{39}H_{35}F_4NO_5S$ ($[M+Na]^+$) 728.2070, found: 728.2068.

4-(((2R,3S,4S,5R,6R)-4,5-Bis(benzyloxy)-6-((benzyloxy)methyl)-2-methoxytetrahydro-2H-pyran-3-yl)thio)-2,3,5,6-tetrafluoropyridine (S12)

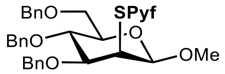

Prepared following the **general procedure C** outlined above starting from (2R,3R,4R,5R,6R)-4,5-bis(benzyloxy)-6-((benzyloxy)methyl)-2-methoxytetrahydro-2H-pyran-3-ol on 2.0 mmol scale to give **S12** (0.35 g, 28%) as a white foam.

^{1}H NMR (400 MHz, CDCl$_3$) δ 7.33 – 7.29 (m, 2H), 7.29 – 7.24 (m, 2H), 7.20 – 7.09 (m, 7H), 7.13 – 7.09 (m, 2H), 7.05 – 7.01 (m, 2H), 4.76 (d, J = 10.8 Hz, 1H), 4.63 (d, J = 12.1 Hz, 1H), 4.53 – 4.47 (m, 4H), 4.41 – 4.36 (m, 2H), 3.99 (t, J = 9.2 Hz, 1H), 3.78 (dd, J = 9.0, 4.1 Hz, 1H), 3.71 (d, J = 3.5 Hz, 2H), 3.44 – 3.40 (m, 1H), 3.39 (s, 3H).

^{13}C NMR (100 MHz, CDCl$_3$) δ 143.62 – 143.42 (m), 142.02 – 141.78 (m), 140.80 – 140.49 (m), 138.38, 137.96, 136.96, 128.61, 128.56, 128.50, 128.22, 128.20, 128.02, 127.74, 127.18, 100.92, 81.41, 75.34, 74.34, 73.67, 72.14, 68.97, 57.15, 51.77.

^{19}F NMR (376 MHz, CDCl$_3$) δ -91.61 – -92.29 (m, 2F), -136.03 – -136.69 (m, 2F).

HRMS (ESI) m/z calcd. for $C_{33}H_{31}F_4NO_5S$ ($[M+Na]^+$) 652.1757, found: 652.1758.

2,3,5,6-Tetrafluoro-4-(((2R,4aR,6S,8R,8aR)-6-methoxy-2-phenylhexahydropyrano[3,2-d][1,3]dioxin-8-yl)thio)pyridine (S13)

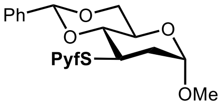

Prepared following the **general procedure C** outlined above starting from (2R,4aR,6S,8S,8aS)-6-methoxy-2-phenylhexahydropyrano[3,2-d][1,3]dioxin-8-ol on 2.0 mmol scale to give **S13** (0.60 g, 69%) as a white foam.

^{1}H NMR (400 MHz, CDCl$_3$) δ 7.35 – 7.25 (m, 3H), 7.12 (dd, *J* = 1.8, 7.9 Hz, 2H), 5.45 (s, 1H), 4.79 (dd, *J* = 1.2, 3.7 Hz, 1H), 4.22 (dd, *J* = 4.9, 10.4 Hz, 1H), 4.19 – 4.12 (m, 1H), 3.88 – 3.79 (m, 1H), 3.71 (d, *J* = 10.3 Hz, 1H), 3.66 – 3.60 (m, 1H), 3.39 (s, 3H), 2.30 -2.24 (m, 1H), 1.90 -1.83 (m, 1H).

^{13}C NMR (100 MHz, CDCl$_3$) δ 144.49 – 144.16 (m), 143.47 – 143.21 (m), 143.13 – 142.87 (m), 141.99 – 141.78 (m), 140.57 – 140.33 (m), 136.66, 129.90, 129.40, 128.33, 125.58, 102.00, 97.84, 84.65, 69.17, 64.39, 54.97, 42.52, 35.70.

^{19}F NMR (376 MHz, CDCl$_3$) δ -91.31 – -91.55 (m, 2F), -135.37 – -135.65 (m, 2F).

HRMS (ESI) m/z calcd. for $C_{19}H_{17}F_4NO_4S$ ($[M+H]^+$) 432.0892, found: 432.0885.

(2R,3R,4R,5S,6R)-6-((Benzoyloxy)methyl)-2-(4-methoxyphenoxy)-5-((perfluoropyridin-4-yl)thio)tetrahydro-2H-pyran-3,4-diyl dibenzoate (S14)

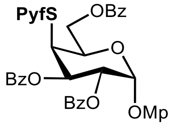

Prepared following the **general procedure C** outlined above starting from (2R,3R,4S,5R,6R)-6-((benzoyloxy)methyl)-5-hydroxy-2-(4-methoxyphenoxy)tetrahydro-2H-pyran-3,4-diyl dibenzoate on 2.0 mmol scale to give **S14** (0.44 g, 34%) as a white foam.

^{1}H NMR (400 MHz, CDCl$_3$) δ 8.12 – 8.02 (m, 1H), 7.96 – 7.90 (m, 2H), 7.60 (s, 1H), 7.57 – 7.45 (m, 3H), 7.49 – 7.36 (m, 5H), 6.92 (d, *J* = 9.1 Hz, 1H), 6.68 (d, *J* = 9.1 Hz, 1H), 5.79 (t, *J* = 9.5 Hz, 1H), 5.69 (dd, *J* = 7.7, 9.7 Hz, 1H), 5.30 (s, 1H), 5.20 (d, *J* = 7.7 Hz,

1H), 4.67 (dd, J = 2.9, 12.1 Hz, 1H), 4.56 (dd, J = 5.9, 12.1 Hz, 1H), 4.16 – 4.09 (m, 3H), 4.06 – 3.96 (m, 1H).

^{13}C NMR (100 MHz, CDCl$_3$) δ 166.19, 165.77, 165.26, 156.00 – 155.89 (m), 154.18 – 154.07 (m), 153.63 – 153.50 (m), 151.12 – 151.01 (m), 151.10 – 150.80 (m), 133.56, 133.47, 133.42, 132.20, 130.05, 129.97, 128.55, 128.28, 119.23, 114.59, 73.11, 72.73, 72.12, 71.78, 65.03, 64.21, 64.11, 62.97, 62.87, 62.43, 60.54, 55.70, 14.49, 14.36, 14.32, 14.29.

^{19}F NMR (376 MHz, CDCl$_3$) δ -82.71 – -93.57 (m, 2F), -134.29 – -134.65 (m, 2F).

HRMS (ESI) m/z calcd. for $C_{39}H_{29}F_4NO_9S$ ([M+Na]$^+$) 786.1397, found: 786.1392.

2,3,5,6-Tetrafluoro-4-((((2S,3S,4S,5R,6S)-3,4,5-tris(benzyloxy)-6-methoxytetra--hydro-2H-pyran-2-yl)methyl)thio)pyridine (S15)

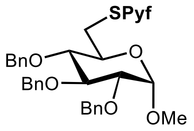

Prepared following the **general procedure C** outlined above starting from ((2R,3R,4S,5R,6S)-3,4,5-tris(benzyloxy)-6-methoxytetrahydro-2H-pyran-2-yl)methanol on 5.0 mmol scale to give **S15** (2.89 g, 92%) as a white solid.

^{1}H NMR (400 MHz, CDCl$_3$) δ 7.38 – 7.21 (m, 15H), 4.96 (d, J = 11.0, 2H), 4.82 – 4.75 (m, 2H), 4.61 (d, J = 11.7, 2H), 4.43 (d, J = 3.5 Hz, 1H), 3.97 (t, J = 9.2 Hz, 1H), 3.81 (dd, J = 2.8, 8.0, 9.0 Hz, 1H), 3.48 (dd, J = 3.6, 9.7 Hz, 1H), 3.46 – 3.40 (m, 1H), 3.38 (d, J = 9.4 Hz, 1H), 3.35 (s, 3H), 3.10 – 3.01 (m, 1H).

^{13}C NMR (100 MHz, CDCl$_3$) δ 145.01 – 144.44 (m), 142.52 – 142.13 (m), 142.33 – 141.90 (m), 139.96 – 139.60 (m), 138.51, 137.96, 137.83, 131.97 – 131.57 (m), 128.56, 128.53, 128.51, 128.14, 128.07, 127.79, 98.03, 81.80, 79.92, 79.80, 75.86, 75.16, 73.48, 70.22, 55.38, 35.22.

^{19}F NMR (376 MHz, $CDCl_3$) δ -91.27 – -91.56 (m, 2F), -136.78 – -137.23 (m, 2F).

HRMS (ESI) m/z calcd. for $C_{33}H_{31}F_4NO_5S$ ($[M+Na]^+$) 652.1757, found: 652.1752.

(Perfluoropyridin-4-yl)-β-D-thioglucopyranoside (S16)

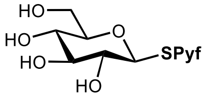

Prepared following the **general procedure B** outlined above starting from D-Glucose on 0.2 mmol scale to give **S16** (48.5 mg, 71%, β/α ＞ 20:1) as a white solid.

^{1}H NMR (400 MHz, CD_3OD) δ 5.20 (d, *J* = 9.5 Hz, 1H), 3.79 -3.73 (m, 1H), 3.59 – 3.52 (m, 1H), 3.47 – 3.39 (m, 1H), 3.33 (dd, *J* = 3.5, 1.8 Hz, 3H).

^{13}C NMR (150 MHz, CD_3OD) δ 144.30 – 143.90 (m), 142.66 – 142.34 (m), 142.34 – 141.99 (m), 140.84 – 139.85 (m), 129.13 – 128.23 (m), 83.52, 81.20, 78.09, 74.20, 69.83, 61.12.

^{19}F NMR (376 MHz, CD_3OD) δ -94.77 – -95.02 (m, 2F), -139.36 – -139.60 (m, 2F).

HRMS (ESI) m/z calcd. for $C_{11}H_{11}F_4NO_5S$ ($[M+Na]^+$) 368.0192, found: 368.0185.

(Perfluoropyridin-4-yl)-α-D-thiomannopyranoside (S17)

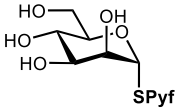

Prepared following the **general procedure B** outlined above starting from D-Mannose on 0.2 mmol scale to give **S17** (42.8 mg, 62%, α/β ＞ 20:1) as a white solid.

^{1}H NMR (400 MHz, CD_3OD) δ 5.89 (d, *J* = 1.4 Hz, 1H), 4.12 (dd, *J* = 1.4, 3.5 Hz, 1H), 3.87 – 3.83 (m, 1H), 3.83 – 3.81 (m, 1H), 3.74 (dd, *J* = 2.3, 12.1 Hz, 1H), 3.69 (d, *J* = 9.6 Hz, 1H), 3.64 – 3.59 (m, 1H).

^{13}C NMR (150 MHz, CD_3OD) δ 145.14 – 144.48 (m), 144.23 – 143.71 (m), 142.61 – 141.60 (m), 141.65 – 141.18 (m), 134.75 – 124.04 (m), 86.60, 75.66, 71.36, 71.28, 67.01, 60.91.

^{19}F NMR (376 MHz, CD_3OD) δ -90.17 – -96.77 (m, 2F), -132.62 – -147.03 (m, 2F).

HRMS (ESI) m/z calcd. for $C_{11}H_{11}F_4NO_5S$ ($[M+Na]^+$) 368.0192, found: 368.0186.

(Perfluoropyridin-4-yl)-β-D-thiogalactopyranoside (S18)

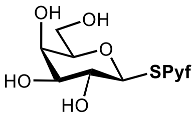

Prepared following the **general procedure B** outlined above starting from D-Galactose on 0.2 mmol scale to give **S18** (26.2 mg, 38%, β/α > 20:1) as a white solid.

^{1}H NMR (400 MHz, CD_3OD) δ 5.15 (d, *J* = 9.6 Hz, 1H), 3.91 (d, *J* = 3.4 Hz, 1H), 3.68 (d, *J* = 9.4 Hz, 1H), 3.65 (d, *J* = 2.0 Hz, 1H), 3.60 (d, *J* = 1.1 Hz, 1H), 3.55 (dd, *J* = 9.1, 3.4 Hz, 1H), 3.33 – 3.25 (m, 1H).

^{13}C NMR (150 MHz, CD_3OD) δ 144.22 – 143.93 (m), 142.47 – 142.26 (m), 142.39 – 142.23 (m), 140.81 – 140.58 (m), 128.71 – 128.46 (m), 84.18, 79.93, 74.68, 71.30, 69.11, 61.06.

^{19}F NMR (376 MHz, CD_3OD) δ -94.79 – -95.03 (m, 2F), -139.11 – -139.34 (m, 2F).

HRMS (ESI) m/z calcd. for $C_{11}H_{11}F_4NO_5S$ ($[M+Na]^+$) 368.0192, found: 368.0183.

(Perfluoropyridin-4-yl)-α-L-thiorhamnopyranoside (S19)

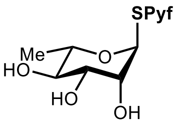

Prepared following the **general procedure B** outlined above starting from L-Rhamnose on 0.2 mmol scale to give **S19** (30.3 mg, 46%, α/β > 20:1) as a white solid.

^{1}H NMR (400 MHz, CD_3OD) δ 5.73 (d, *J* = 1.4 Hz, 1H), 4.15 – 4.08 (m, 1H), 3.91 (dd, *J* = 6.2, 9.4 Hz, 1H), 3.76 (dd, *J* = 3.5, 9.5 Hz, 1H), 3.48 (d, *J* = 9.4 Hz, 1H), 1.19 (d, *J* = 6.2 Hz, 3H).

^{13}C NMR (150 MHz, CD_3OD) δ 145.14 – 144.48 (m), 144.23 – 143.71 (m), 142.61 – 141.60 (m), 141.65 – 141.18 (m), 127.65 – 126.05 (m), 86.60, 75.66, 71.36, 71.28, 67.01, 60.91.

^{19}F NMR (376 MHz, CD_3OD) δ -93.33 – -93.71 (m, 2F), -137.93 -138.37 (m, 2F).

HRMS (ESI) m/z calculated for $C_{11}H_{11}F_4NO_4S$ ($[M-H]^-$) 328.0272, found: 328.0272.

(Perfluoropyridin-4-yl)-β-D-thioribofuranoside (S20)

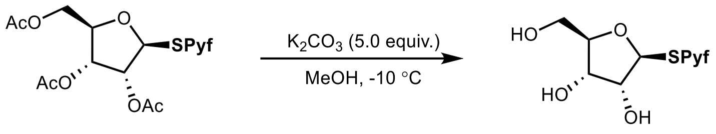

S7 **S20**

To a solution of glycosyl precursors **S7** (2.0 mmol, 1.0 equiv.) in MeOH (0.1 M), K_2CO_3 (0.2 equiv.) was added at -10 °C. After the complete consumption of glycosyl acetate as monitored by TLC analysis, the reaction mixture was filtered and concentrated in vacuo. The resulting residue was purified by silica gel flash chromatography to give the thioglycosides donor **S20** as a colorless oil.

^{1}H NMR (400 MHz, CD_3OD) δ 5.75 (d, 1H), 4.32 (dd, *J* = 6.6, 4.8 Hz, 1H), 4.28 (dd, *J* = 11.6, 2.7 Hz, 1H), 4.20 (dd, *J* = 4.8, 2.3 Hz, 1H), 4.15 (ddd, *J* = 6.4, 6.0, 2.7 Hz, 1H), 4.09 (dd, *J* = 11.7, 5.4 Hz, 1H).

^{13}C NMR (100 MHz, CD_3OD) δ 144.56 – 144.37 (m), 144.20 – 144.04 (m), 143.64 – 143.43 (m), 142.02 – 141.84 (m), 141.67 – 141.50 (m), 90.18, 83.88, 77.41, 72.58, 64.88.

^{19}F NMR (376 MHz, CD_3OD) δ -94.03 – -94.27 (m, 2F), -138.30 - -138.54 (m, 2F).

HRMS (ESI) m/z calculated for $C_{10}H_9F_4NO_4S$ ($[M-H]^-$) 314.0116, found: 314.0126.

Tetrafluoropyridinyl thio-β-cyclodextrin (S21)

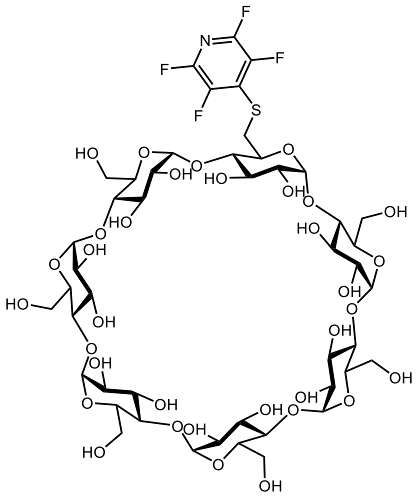

Prepared following the **general procedure C** outlined above (DMF as reaction solvent, *c* = 0.044 M) starting from β-Cyclodextrin on 0.3 mmol scale to give **S21** (39.0 mg, 10%) as a white solid.

^{1}H NMR (400 MHz, D_2O) δ 5.07 (s, 1H), 4.99 - 4.92 (m, 5H), 4.89 (s, 1H), 3.95 – 3.85 (m, 8H), 3.79 - 3.55 (m, 16H), 3.55 – 3.47 (m, 18H).

^{13}C NMR (100 MHz, D_2O) δ 144.31 – 143.36 (m), 142.73 – 140.33 (m), 142.34 – 142.05 (m), 141.00 – 140.62 (m), 131.03 – 130.73 (m), 102.24, 102.04, 101.86, 101.75, 100.83, 84.26, 81.23, 81.15, 81.06, 80.67, 80.34, 73.25, 73.12, 73.00, 72.83, 72.61, 72.21, 72.03, 71.89, 71.79, 71.66, 71.51, 70.60, 60.04, 59.91, 35.27.

^{19}F NMR (376 MHz, D_2O) δ -91.79 – -92.31 (m, 2F), -136.21 – -136.74 (m, 2F).

HRMS (ESI) m/z calcd. for $C_{47}H_{69}F_4NO_{34}S$ ($[M+H]^+$) 1300.3436, found: 1300.3434.

(2S,3R,4S,5S,6S)-2-Methoxy-6-(((perfluoropyridin-4-yl)thio)methyl)tetrahydro-2H-pyran-3,4,5-triol (S22)

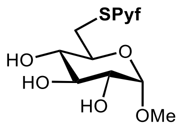

Prepared following the **general procedure C** outlined above starting from alpha-D-Methylglucoside on 5.0 mmol scale to give **S22** (1.59 g, 89%) as a white foam.

^{1}H NMR (400 MHz, CD_3OD) δ 4.55 (d, *J* = 3.8 Hz, 1H), 3.71 (d, *J* = 8.9 Hz, 1H), 3.65 (dd, *J* = 2.5, 13.9 Hz, 1H), 3.58 (t, *J* = 9.2 Hz, 1H), 3.38 (s, 3H), 3.37 – 3.35 (m, 1H), 3.26 (t, *J* = 9.2 Hz, 1H).

^{13}C NMR (100 MHz, CD_3OD) δ 146.04 – 145.63 (m), 144.12 – 143.89 (m), 143.60 – 143.23 (m), 141.92 – 141.72 (m), 133.50 – 133.24 (m), 101.31, 74.68, 74.28, 73.36, 73.11, 55.64, 36.39.

^{19}F NMR (376 MHz, CD_3OD) δ -90.66 – -101.24 (m, 2F), -131.84 – -148.31 (m, 2F).

HRMS (ESI) m/z calcd. for $C_{12}H_{13}F_4NO_5S$ ($[M-H]^-$) 358.0378, found: 358.0372.

(2S,3S,4S,5S,6S)-2-Methoxy-6-(((perfluoropyridin-4-yl)thio)methyl)tetrahydro-2H-pyran-3,4,5-triol (S23)

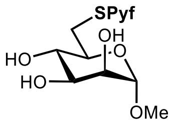

Prepared following the **general procedure C** outlined above starting from alpha-D-Methylmannoside on 5.0 mmol scale to give **S23** (1.38 g, 77%) as a white foam.

^{1}H NMR (400 MHz, CD_3OD) δ 4.52 (d, *J* = 1.7 Hz, 1H), 3.79 (dd, *J* = 1.7, 3.1 Hz, 1H), 3.72 – 3.64 (m, 2H), 3.64 – 3.58 (m, 2H), 3.45 – 3.38 (m, 1H), 3.36 (s, 3H).

^{13}C NMR (100 MHz, CD_3OD) δ 146.00 – 145.65 (m), 144.10 – 143.90 (m), 143.54 – 143.28 (m), 141.57 – 141.38 (m), 133.50 – 133.25 (m), 102.81, 74.17, 72.18, 71.84, 71.17, 55.28, 36.45.

^{19}F NMR (376 MHz, CD_3OD) δ -89.95 – -99.25 (m, 2F), -132.48 – -148.31 (m, 2F).

HRMS (ESI) m/z calcd. for $C_{12}H_{13}F_4NO_5S$ ($[M+Na]^+$) 382.0349, found: 382.0340.

(2S,3R,4R,5S,6S)-2-(4-Chloro-3-(4-(((S)-tetrahydrofuran-3-yl)oxy)benzyl)phenyl)-6-(((perfluoropyridin-4-yl)thio)methyl)tetrahydro-2H-pyran-3,4,5-triol (S24)

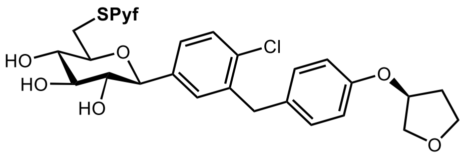

Prepared following the **general procedure C** outlined above starting from Empagliflozin on 2.0 mmol scale to give **S24** (0.61g, 77%) as a white foam.

^{1}H NMR (400 MHz, CD_3OD) δ 7.27 (d, J = 8.2 Hz, 1H), 7.10 – 7.05 (m, 2H), 6.97 (d, J = 2.1 Hz, 1H), 6.87 (dd, J = 8.2, 2.1 Hz, 1H), 6.82 – 6.76 (m, 2H), 4.95 (ddd, J = 6.2, 4.2, 2.0 Hz, 1H), 4.05 – 4.01 (m, 1H), 3.97 – 3.87 (m, 5H), 3.84 (dd, J = 8.3, 4.2 Hz, 1H), 3.58 (dd, J = 8.4, 2.4 Hz, 1H), 3.49 – 3.43 (m, 1H), 3.41 – 3.36 (m, 2H), 3.22 – 3.18 (m, 1H), 2.20 -1.84 (m, 1H), 2.12 – 2.04 (m, 1H).

^{13}C NMR (100 MHz, CD_3OD) δ 157.22, 145.49 – 145.17 (m), 144.77 – 144.56 (m), 144.47 – 144.19 (m), 143.09 – 142.76 (m), 141.95 – 141.66 (m), 139.98, 139.08, 134.78, 133.14, 131.31, 131.04, 130.24, 127.16, 116.42, 82.85, 82.72, 79.28, 78.51, 76.00, 74.00, 68.10, 39.16, 35.86, 33.84.

^{19}F NMR (376 MHz, CD_3OD) δ -94.64 – -94.99 (m, 2F), -138.70 – -138.96 (m, 2F).

HRMS (ESI) m/z calcd. for $C_{28}H_{26}ClF_4NO_6S$ ($[M+H]^+$) 616.1183, found: 616.1177.

(2R,3R,4S,5S)-2-Methoxy-5-(((perfluoropyridin-4-yl)thio)methyl)tetrahydrofuran-3,4-diol (S25)

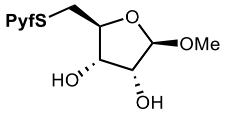

Prepared following the **general procedure C** outlined above starting from beta-methyl-D-ribofuranoside on 5.0 mmol scale to give **S25** (1.02 g, 62%) as a white foam.

^{1}H NMR (400 MHz, CD_3OD) δ 4.70 (s, 1H), 4.16 – 4.06 (m, 2H), 3.94 – 3.79 (m, 1H), 3.60 – 3.50 (m, 1H), 3.43 – 3.38 (m, 1H), 3.26 (s, 3H).

^{13}C NMR (100 MHz, CD_3OD) δ 146.04 – 145.41 (m), 144.84 – 143.97 (m), 143.21 – 143.45 (m), 142.23 – 141.13 (m), 133.19 – 131.94 (m), 110.12, 83.24, 76.08, 75.09, 55.51, 38.13.

^{19}F NMR (376 MHz, CD_3OD) δ -84.27 – -102.87 (m, 2F), -128.86 – -145.61 (m, 2F).

HRMS (ESI) m/z calculated for $C_{11}H_{11}F_4NO_4S$ ($[M-H]^-$) 328.0272, found: 328.0265.

1-((2R,3R,4S,5S)-3,4-Dihydroxy-5-(((perfluoropyridin-4-yl)thio)methyl)tetrahydrofuran-2-yl)pyrimidine-2,4(1H,3H)-dione (S26)

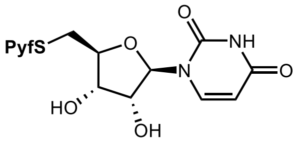

Prepared following the **general procedure C** outlined above starting from uridine on 5.0 mmol scale to give **S26** (1.12 g, 55%) as a white foam.

^{1}H NMR (400 MHz, CD3OD) δ 7.54 (d, *J* = 8.1 Hz, 1H), 5.70 (d, *J* = 8.0 Hz, 1H), 5.68 (d, *J* = 3.9 Hz, 1H), 4.28 (dd, 1H), 4.13 (d, *J* = 5.9 Hz, 1H), 4.10 – 4.03 (m, 1H), 3.72 – 3.62 (m, 1H), 3.53 (dd, *J* = 14.1, 7.3 Hz, 1H), 3.31 (d, *J* = 1.6 Hz, 1H).

^{13}C NMR (100 MHz, CD_3OD) δ 165.95, 151.98, 146.02 – 145.33 (m), 144.47 – 143.94 (m), 143.89 – 143.42 (m), 142.17 – 141.26 (m), 132.96 – 131.97 (m), 103.01, 93.03, 84.35, 74.28, 73.55.

^{19}F NMR (376 MHz, CD_3OD) δ -93.43 – -95.49 (m, 2F), -138.23 – -139.72 (m, 2F).

HRMS (ESI) m/z calcd. for $C_{14}H_{11}F_4N_3O_5S$ ($[M+H]^+$) 410.0434, found: 410.0428.

3) Reaction Optomization

To an 8 mL vial equipped with a stir bar was added glycosyl donors (0.10 mmol), benzyl acrylate (1.5 equiv.), DIPEA (2.0 equiv.), Ir[(dtbbpy)(ppy)$_2$]PF$_6$ (2 mmol%.), and MeCN (2.0 mL), the vial was sealed and the solution was degassed by sparging with nitrogen for 10 minutes before sealing with parafilm. Then the reaction was stirred and irradiated using 40 W 427 nm blue LED lamps (5 cm away, with cooling fan & air-condition at 25 ℃ to maintain the reaction at room temperature) for 6 h. The reaction was quenched by exposure to air. The solution of mesitylene (1.0 equiv.) as internal standard was added then the reaction mixture was analyzed by ^{1}H NMR.

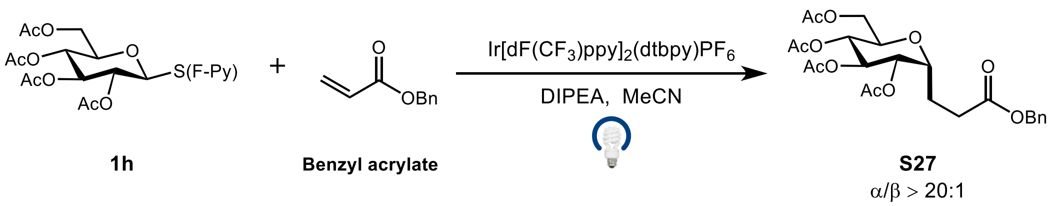

Entry	Variation of standard condition	Yield/%
1	none	87
2	[Ir(dtbpy)(ppy)$_2$]PF$_6$ instead of PC	40
3	4CZIPN instead of PC	trace
4	N,N-Dicyclohexylmethylamine instead of DIPEA	63
5	NEt$_3$ instead of DIPEA	48
6	DMSO instead of MeCN	84
7	THF instead of MeCN	27
8	without light	N.D.
9	without PC	trace
10	without reductant	trace

Table S1. Evaluation of Reaction Conditions. Yields Determined by ^{1}H NMR.

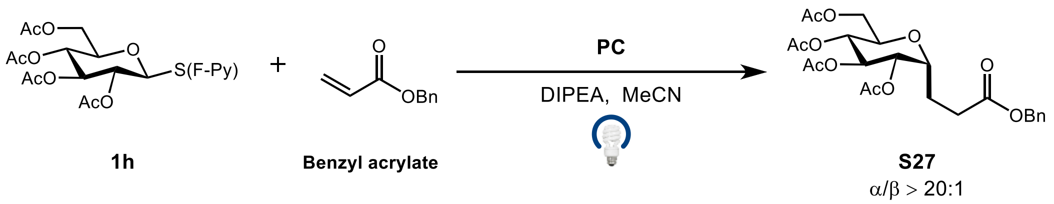

Entry	Screening of Photocatalysts	Yield/%
1	$Ir[dF(CF_3)ppy]_2(dtbpy)PF_6$	87
2	*fac*-$Ir(ppy)_3$	trace
3	$[Ir(dtbpy)(ppy)_2]PF_6$	40
4	4DPAIPN	trace
5	4CZIPN	trace
6	*Eosin* Y	21

Table S2. Optimization of Different Photocatalysts. Yields Determined by ^{1}H NMR.

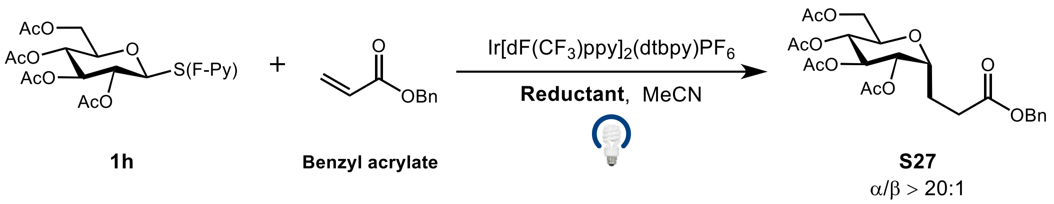

Entry	Screening of Reductant	Yield/%
1	DIPEA	87
2	Quinuclidine	15
3	N,N-Dicyclohexylmethylamine	63
4	Ph_3N	N.D.
5	NEt_3	48

Table S3. Optimization of Different Reductants. Yields Determined by ^{1}H NMR.

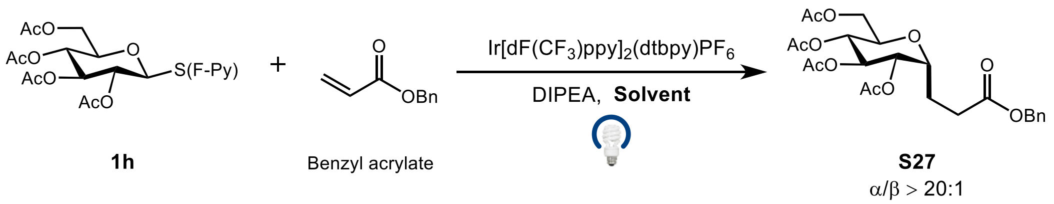

Entry	Screening of Solvent	Yield/%
1	MeCN	87
2	DMSO	84
3	DMF	63
4	Acetone	27
5	DCM	12
6	THF	27
7	1,4-dioxane	36
8	$PhCF_3$	4
9	EA	15
10	DMA	45

Table S4. Optimization of Different Solvents. Yields Determined by ^{1}H NMR.

4) Procedures of C-Alkyl Modification with Carbohydrates

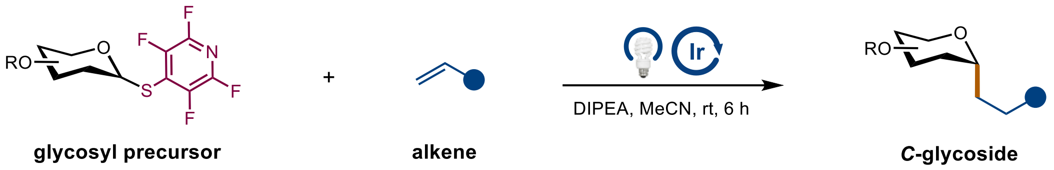

General procedure E: To an 8 mL vial equipped with a stir bar was added glycosyl thioglycoside (0.1 mmol, 1.0 equiv.), Ir[dF(CF_3)ppy]$_2$(dtbbpy)PF_6 (2.2 mg, 2 mmol%), alkene (0.15 mmol, 1.5 equiv.), DIPEA (19.2 μL, 0.2 mmol, 2.0 equiv.), and MeCN (1.0 mL). The vial was sealed and the solution was degassed by sparging with nitrogen for 5 minutes before sealing with parafilm. Then the reaction was stirred and irradiated using 40 W 427 nm blue LED lamps (5 cm away, with cooling fan & air-condition at 25 °C to maintain the reaction at room temperature) for 6 h. Considering the self-polymerization or hydrogenation of vinyl pyridine, another portion of vinyl pyridine (0.5 equiv.) was added after one hour. The reaction was quenched by exposure to air and concentrated. The residue was purified by flash chromatography on silica gel to afford the desired product.

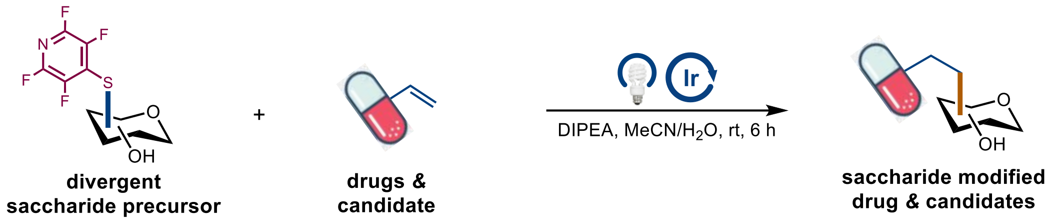

General procedure F: To an 8 mL via equipped with a stir bar was added glycosyl thioglycoside (0.1 mmol, 1.0 equiv.), Ir[dF(CF_3)ppy]$_2$(dtbbpy)PF_6 (2.2 mg, 2 mmol%), alkene (16.2 μL, 0.15 mmol, 1.5 equiv.), DIPEA (19.2 μL, 0.2 mmol, 2.0 equiv.), MeCN/H_2O = 9:1 (1.0 mL). The vial was sealed and the solution was degassed by sparging with nitrogen for 5 minutes before sealing with parafilm. Then the reaction was stirred and irradiated using 40 W 427 nm blue LED lamps (5 cm away, with cooling fan & air-condition at 25 °C to maintain the reaction at room temperature) for 6 h. Considering the self-polymerization or hydrogenation of vinyl pyridine, another portion of vinyl pyridine (0.5 equiv.) was added after one hour. The reaction was quenched by exposure to air and concentrated. The residue was purified by flash chromatography on silica gel to afford the desired product.

5) Experimental Data

(2R,3R,4R,5S,6R)-2-(Acetoxymethyl)-6-(2-(pyridin-2-yl)ethyl)tetrahydro-2H-pyran-3,4,5-triyl triacetate (2)

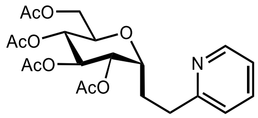

Prepared following the **general procedure E** outlined above using Ir[dF(CF_3)ppy]$_2$(dtbbpy)PF_6 (2.2 mg, 2 mmol%), glycosyl donor **S3** (0.1 mmol), 2-vinylpyridine (16.2 μL, 0.15 mmol, 1.5 equiv.), DIPEA (19.2 μL, 0.2 mmol, 2.0 equiv.), and MeCN (1 mL). Purification by flash chromatography (PE:EA = 3:1) provided the title compound (37.1 mg, 85% yield) as a colorless oil.

^{1}H NMR (400 MHz, $CDCl_3$) δ 8.53 (d, *J* = 4.8 Hz, 1H), 7.61 (dd, *J* = 1.8, 7.6 Hz, 1H), 7.16 (d, *J* = 8.2 Hz, 2H), 7.13 (d, *J* = 5.3 Hz, 1H), 5.35 (t, *J* = 9.3 Hz, 1H), 5.09 (dd, *J* = 5.9, 9.7 Hz, 1H), 5.00 (t, *J* = 9.3 Hz, 1H), 4.23 (d, *J* = 5.0 Hz, 1H), 4.19 (s, 1H), 4.01 (dd, *J* = 2.5, 12.2 Hz, 1H), 3.94 – 3.87 (m, 1H), 2.99 – 2.86 (m, 1H), 2.83 – 2.74 (m, 1H), 2.33 – 2.20 (m, 1H), 2.09 (s, 3H), 2.03 (s, 3H), 2.02 (s, 3H), 2.00 (s, 3H).

^{13}C NMR (100 MHz, $CDCl_3$) δ 170.78, 170.24, 169.81, 169.68, 160.71, 149.40, 136.79, 123.31, 121.57, 72.65, 70.72, 70.57, 69.12, 68.77, 62.51, 33.68, 25.24, 20.88, 20.85, 20.80, 20.76.

HRMS (ESI) m/z calcd. for $C_{21}H_{27}NO_9$ ($[M+H]^+$) 438.1764, found: 438.1754.

(2R,3S,4R,5S,6R)-2-(Acetoxymethyl)-6-(2-(pyridin-2-yl)ethyl)tetrahydro-2H-pyran-3,4,5-triyl triacetate (7)

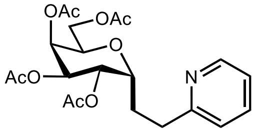

Prepared following the **general procedure E** outlined above using Ir[dF(CF_3)ppy]$_2$(dtbbpy)PF_6 (2.2 mg, 2 mmol%), glycosyl donor **S3** (0.1 mmol), 2-

vinylpyridine (16.2 μL, 0.15 mmol, 1.5 equiv.), DIPEA (19.2 μL, 0.2 mmol, 2.0 equiv.), and MeCN (1 mL). Purification by flash chromatography (PE:EA = 3:1) provided the title compound (31.9 mg, 73% yield) as a colorless oil.

^{1}H NMR (400 MHz, CDCl$_3$) δ 8.51 (d, *J* = 3.3 Hz, 1H), 7.61 – 7.55 (m, 1H), 7.16 – 7.08 (m, 2H), 5.39 (d, *J* = 3.4 Hz, 1H), 5.28 (dd, *J* = 9.8, 5.4 Hz, 1H), 5.20 (dd, *J* = 9.9, 3.4 Hz, 1H), 4.23 (ddd, *J* = 11.6, 5.4, 3.4 Hz, 1H), 4.13 – 4.03 (m, 3H), 2.95 – 2.85 (m, 1H), 2.76 (dd, *J* = 9.4, 7.0 Hz, 1H), 2.21 – 2.12 (m, 1H), 2.10 (s, 3H), 2.03 (d, *J* = 3.5 Hz, 6H), 1.98 (s, 3H), 1.96 – 1.86 (m, 1H).

^{13}C NMR (100 MHz, CDCl$_3$) δ 170.60, 170.23, 170.06, 169.98, 160.70, 149.27, 136.80, 123.26, 121.49, 72.09, 68.26, 68.12, 67.90, 67.82, 61.69, 33.73, 25.42, 20.88, 20.82, 20.76, 20.73.

HRMS (ESI) m/z calcd. for $C_{21}H_{27}NO_9$ ([M+H]$^+$) 438.1764, found: 438.1754.

(2R,3R,4R,5R,6R)-2-(Acetoxymethyl)-6-(2-(pyridin-2-yl)ethyl)tetrahydro-2H-pyran-3,4,5-triyl triacetate (8)

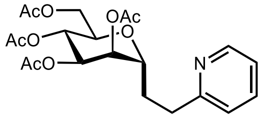

Prepared following the **general procedure E** outlined above using Ir[dF(CF$_3$)ppy]$_2$(dtbbpy)PF$_6$ (2.2 mg, 2 mmol%), glycosyl donor **S4** (0.1 mmol), 2-vinylpyridine (16.2 μL, 0.15 mmol, 1.5 equiv.), DIPEA (19.2 μL, 0.2 mmol, 2.0 equiv.), and MeCN (1 mL). Purification by flash chromatography (PE:EA = 3:1) provided the title compound (31.4 mg, 72% yield) as a colorless oil.

^{1}H NMR (400 MHz, CDCl$_3$) δ 8.54 (d, *J* = 4.8 Hz, 1H), 7.65 – 7.55 (m, 1H), 7.20 – 7.09 (m, 2H), 5.30 – 5.15 (m, 3H), 4.31 (dd, *J* = 12.1, 5.8 Hz, 1H), 4.01 (dd, *J* = 12.1, 2.9 Hz, 2H), 3.92 (ddd, *J* = 8.3, 5.8, 2.8 Hz, 1H), 2.95 – 2.91 (m, 1H), 2.89 – 2.79 (m, 1H), 2.37 – 2.24 (m, 2H), 2.11 (d, *J* = 6.1 Hz, 6H), 2.05 (s, 3H), 2.01 (s, 3H).

^{13}C NMR (100 MHz, CDCl$_3$) δ 170.86, 170.42, 170.10, 169.83, 160.26, 137.12, 123.52, 121.71, 74.89, 70.91, 70.38, 69.20, 67.01, 62.59, 33.82, 28.34, 21.12, 20.94, 20.90, 20.84.

HRMS (ESI) m/z calcd. for $C_{21}H_{27}NO_9$ ($[M+H]^+$) 438.1764, found: 438.1754.

(2S,3S,4R,5S,6S)-2-Methyl-6-(2-(pyridin-2-yl)ethyl)tetrahydro-2H-pyran-3,4,5-triyl triacetate (9)

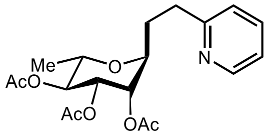

Prepared following the **general procedure E** outlined above using Ir[dF(CF$_3$)ppy]$_2$(dtbbpy)PF$_6$ (2.2 mg, 2 mmol%), glycosyl donor **S5** (0.1 mmol), 2-vinylpyridine (16.2 μL, 0.15 mmol, 1.5 equiv.), DIPEA (19.2 μL, 0.2 mmol, 2.0 equiv.), and MeCN (1 mL). Purification by flash chromatography (PE:EA = 3:1) provided the title compound (35.6 mg, 73% yield) as a colorless oil.

^{1}H NMR (400 MHz, CDCl$_3$) δ 8.52 (d, *J* = 4.0 Hz, 1H), 7.58 (dd, *J* = 7.6, 1.9 Hz, 1H), 7.19 – 7.07 (m, 2H), 5.24 – 5.15 (m, 2H), 5.02 (t, *J* = 8.9 Hz, 1H), 3.91 (ddd, *J* = 10.9, 4.4, 2.3 Hz, 1H), 3.78 (dd, *J* = 8.8, 6.2 Hz, 1H), 2.99 – 2.90 (m, 1H), 2.82 – 2.77 (m, 2H), 2.37 – 2.24 (m, 1H), 2.10 (s, 3H), 2.03 (s, 3H), 1.98 (s, 3H).

^{13}C NMR (100 MHz, CDCl$_3$) δ 170.50, 170.22, 170.06, 160.53, 149.32, 136.75, 123.32, 121.50, 74.96, 71.68, 71.36, 69.43, 67.94, 34.25, 28.36, 21.12, 20.96, 20.84, 17.81.

HRMS (ESI) m/z calcd. for $C_{19}H_{25}NO_7$ ($[M+H]^+$) 380.1709, found: 380.1699.

(2S,3S,4R,5S,6R)-2-(Methoxycarbonyl)-6-(2-(pyridin-2-yl)ethyl)tetrahydro-2H-pyran-3,4,5-triyl triacetate (10)

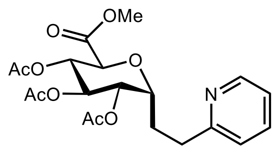

Prepared following the **general procedure E** outlined above using Ir[dF(CF_3)ppy]$_2$(dtbbpy)PF_6 (2.2 mg, 2 mmol%), glycosyl donor **S6** (0.1 mmol), 2-vinylpyridine (16.2 μL, 0.15 mmol, 1.5 equiv.), DIPEA (19.2 μL, 0.2 mmol, 2.0 equiv.), and MeCN (1 mL). Purification by flash chromatography (PE:EA = 3:1) provided the title compound (36.0 mg, 85% yield) as a colorless oil.

^{1}H NMR (400 MHz, $CDCl_3$) δ 8.50 (d, *J* = 6.5 Hz, 1H), 7.57 (d, *J* = 8.6 Hz, 1H), 7.17 (d, *J* = 7.9 Hz, 1H), 7.10 (dd, *J* = 8.6, 4.9 Hz, 1H), 5.26 (t, *J* = 3.8 Hz, 1H), 5.11 (t, *J* = 4.4 Hz, 1H), 4.79 (dd, *J* = 4.7, 2.7 Hz, 1H), 4.51 (d, *J* = 3.3 Hz, 1H), 4.32 (dd, *J* = 10.1, 3.3 Hz, 1H), 3.73 (s, 3H), 3.11 – 3.01 (m, 2H), 2.95 – 2.85 (m, 2H), 2.09 (s, 3H), 2.08 (s, 3H), 2.00 (s, 3H).

^{13}C NMR (100 MHz, $CDCl_3$) δ 169.92, 169.56, 168.92, 168.60, 161.11, 149.28, 136.58, 123.19, 121.33, 73.00, 70.41, 68.25, 67.41, 66.52, 52.46, 33.92, 29.37, 20.95, 20.82, 20.67.

HRMS (ESI) m/z calcd. for $C_{21}H_{25}NO_9$ ($[M+H]^+$) 424.1607, found: 424.1599.

(2R,3R,4S,5S)-2-(Acetoxymethyl)-5-(2-(pyridin-2-yl)ethyl)tetrahydrofuran-3,4-diyl diacetate (11)

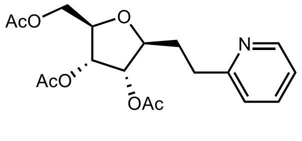

Prepared following the **general procedure E** outlined above using Ir[dF(CF_3)ppy]$_2$(dtbbpy)PF_6 (2.2 mg, 2 mmol%), glycosyl donor **S7** (0.1 mmol), 2-vinylpyridine (16.2 μL, 0.15 mmol, 1.5 equiv.), DIPEA (19.2 μL, 0.2 mmol, 2.0 equiv.), and MeCN (1 mL). Purification by flash chromatography (PE:EA = 3:1) provided the title compound (28.1 mg, 77% yield) as a colorless oil.

^{1}H NMR (400 MHz, $CDCl_3$) δ 8.51 (d, *J* = 7.9 Hz, 1H), 7.58 (dd, *J* = 7.7, 1.9 Hz, 1H), 7.19 – 7.06 (m, 2H), 5.17 (d, *J* = 5.6 Hz, 1H), 4.97 (t, *J* = 5.9 Hz, 1H), 4.31 (d, *J* = 10.0 Hz, 1H), 4.17 – 4.08 (m, 2H), 4.01 (ddd, *J* = 8.4, 6.1, 4.4 Hz, 1H), 2.96 (s, 1H), 2.89 – 2.81 (m, 1H), 2.20 – 2.10 (m, 1H), 2.08 (s, 3H), 2.05 (d, *J* = 2.8 Hz, 6H), 2.02 – 1.88 (m, 1H).

^{13}C NMR (100 MHz, $CDCl_3$) δ 170.80, 169.96, 169.95, 160.95, 149.21, 136.75, 123.13, 121.42, 80.51, 79.29, 74.35, 71.89, 63.79, 33.94, 33.10, 20.97, 20.74, 20.72.

HRMS (ESI) m/z calcd. for $C_{18}H_{23}NO_7$ ($[M+H]^+$) 366.1553, found: 366.1546.

2-(2-((3aR,4R,6R,6aS)-6-((R)-2,2-Dimethyl-1,3-dioxolan-4-yl)-2,2-dimethyltetrahydrofuro[3,4-d][1,3]dioxol-4-yl)ethyl)pyridine (12)

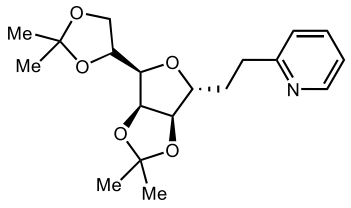

Prepared following the **general procedure E** outlined above using $Ir[dF(CF_3)ppy]_2(dtbbpy)PF_6$ (2.2 mg, 2 mmol%), glycosyl donor **S8** (0.1 mmol), 2-vinylpyridine (16.2 μL, 0.15 mmol, 1.5 equiv.), DIPEA (19.2 μL, 0.2 mmol, 2.0 equiv.), and MeCN (1 mL). Purification by flash chromatography (PE:EA = 3:1) provided the title compound (29.3 mg, 84% yield) as a colorless oil.

^{1}H NMR (400 MHz, $CDCl_3$) δ 8.52 (d, *J* = 4.9 Hz, 1H), 7.59 (dd, *J* = 1.9, 7.7 Hz, 1H), 7.16 (d, *J* = 7.8 Hz, 1H), 7.12 (dd, *J* = 5.1, 6.6 Hz, 1H), 4.77 (dd, *J* = 3.8, 6.0 Hz, 1H), 4.55 (d, *J* = 6.0 Hz, 1H), 4.38 (ddd, *J* = 4.3, 6.3, 7.8 Hz, 1H), 4.12 – 4.01 (m, 2H), 3.92 (dd, *J* = 4.3, 8.7 Hz, 1H), 3.74 (dd, *J* = 3.7, 7.8 Hz, 1H), 2.98 – 2.77 (m, 2H), 2.00 – 1.77 (m, 2H), 1.48 (s, 3H), 1.44 (s, 3H), 1.36 (s, 3H), 1.32 (s, 3H).

^{13}C NMR (100 MHz, $CDCl_3$) δ 160.83, 149.12, 136.90, 123.47, 121.48, 112.66, 109.32, 85.42, 83.77, 80.85, 73.52, 67.13, 34.33, 30.10, 27.10, 26.18, 25.32, 24.69.

HRMS (ESI) m/z calcd. for $C_{19}H_{27}NO_5$ ([M+H]$^+$) 350.1967, found: 350.1959.

(2R,3R,4S,5R,6R)-2-(Acetoxymethyl)-6-(((2R,3R,4S,5S,6R)-4,5-diacetoxy-2-(acetoxymethyl)-6-(2-(pyridin-2-yl)ethyl)tetrahydro-2H-pyran-3-yl)oxy)tetrahydro-2H-pyran-3,4,5-triyl triacetate (13)

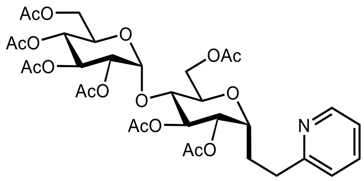

Prepared following the **general procedure E** outlined above using Ir[dF(CF_3)ppy]$_2$(dtbbpy)PF_6 (2.2 mg, 2 mmol%), glycosyl donor **S9** (0.1 mmol), 2-vinylpyridine (16.2 μL, 0.15 mmol, 1.5 equiv.), DIPEA (19.2 μL, 0.2 mmol, 2.0 equiv.), and MeCN (1 mL). Purification by flash chromatography (PE:EA = 3:1) provided the title compound (59.4 mg, 82% yield) as a colorless oil.

^{1}H NMR (400 MHz, $CDCl_3$) δ 8.52 (d, *J* = 4.1 Hz, 1H), 7.60 (dd, *J* = 1.8, 7.6 Hz, 1H), 7.20 – 7.08 (m, 2H), 5.40 – 5.30 (m, 2H), 5.24 (t, *J* = 7.7 Hz, 1H), 5.02 (d, *J* = 9.8 Hz, 1H), 4.93 (dd, *J* = 5.2, 8.1 Hz, 1H), 4.85 (dd, *J* = 3.9, 10.5 Hz, 1H), 4.23 (dd, *J* = 5.6, 10.7 Hz, 3H), 4.12 – 3.99 (m, 3H), 3.97 – 3.90 (m, 1H), 3.83 – 3.75 (m, 1H), 2.92 – 3.87 (m, 1H), 2.84 – 2.71 (m, 1H), 2.18 (s, 2H), 2.11 (s, 3H), 2.08 (s, 3H), 2.03 (s, 6H), 2.00 (s, 6H), 1.97 (s, 3H).

^{13}C NMR (100 MHz, $CDCl_3$) δ 170.68, 170.60, 170.19, 170.05, 169.92, 169.57, 160.80, 149.40, 136.74, 123.26, 121.50, 96.36, 72.09, 71.64, 70.41, 70.11, 69.82, 69.57, 68.51, 68.20, 63.07, 61.68, 33.82, 26.43, 21.03, 20.96, 20.85, 20.79, 20.71, 20.69, 20.68.

HRMS (ESI) m/z calcd. for $C_{33}H_{43}NO_{17}$ ([M+H]$^+$) 726.2609, found: 726.2601.

(2R,3S,4S,5R,6S)-2-(Acetoxymethyl)-6-(((2R,3R,4S,5S,6R)-4,5-diacetoxy-2-(acetoxymethyl)-6-(2-(pyridin-2-yl)ethyl)tetrahydro-2H-pyran-3-yl)oxy)tetrahydro-2H-pyran-3,4,5-triyl triacetate (14)

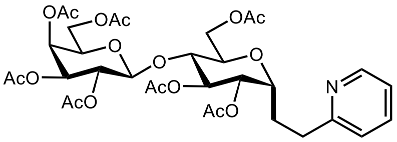

Prepared following the **general procedure E** outlined above using Ir[dF(CF_3)ppy]$_2$(dtbbpy)PF_6 (2.2 mg, 2 mmol%), glycosyl donor **S10** (0.1 mmol), 2-vinylpyridine (16.2 μL, 0.15 mmol, 1.5 equiv.), DIPEA (19.2 μL, 0.2 mmol, 2.0 equiv.), and MeCN (1 mL). Purification by flash chromatography (PE:EA = 3:1) provided the title compound (60.9 mg, 84% yield) as a colorless oil.

^{1}H NMR (400 MHz, $CDCl_3$) δ 8.49 (d, *J* = 4.6 Hz, 1H), 7.57 (d, *J* = 7.7 Hz, 1H), 7.11 (dd, *J* = 7.6, 12.7 Hz, 2H), 5.39 – 5.25 (m, 2H), 5.12 – 5.04 (m, 1H), 4.99 – 4.90 (m, 2H), 4.48 (d, *J* = 7.9 Hz, 1H), 4.30 (dd, *J* = 2.1, 11.8 Hz, 1H), 4.09 -4.00 (m, 5H), 3.89 – 3.78 (m, 2H), 3.65 (t, *J* = 8.4 Hz, 1H), 2.87 -2.82 (m, 1H), 2.73 -2.69 (m, 1H), 2.10 (d, *J* = 8.8 Hz, 6H), 2.02 (t, *J* = 5.6 Hz, 12H), 1.93 (s, 3H).

^{13}C NMR (100 MHz, $CDCl_3$) δ 170.45, 170.34, 170.13, 170.04, 169.95, 169.60, 169.13, 160.62, 149.26, 136.56, 123.04, 121.35, 101.21, 71.93, 70.99, 70.64, 70.29, 70.07, 69.10, 66.69, 62.37, 60.84, 33.51, 25.69, 20.83, 20.75, 20.68, 20.63, 20.59, 20.47.

HRMS (ESI) m/z calcd. for $C_{33}H_{43}NO_{17}$ ($[M+H]^+$) 726.2609, found: 726.2605.

(2R,3R,4R,5S,6R)-2-(Benzyloxy)methyl)-3,4,5-tris(benzyloxy)-6-(2-(pyridin-2-yl)ethyl)tetrahydro-2H-pyran (15)

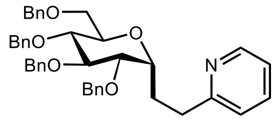

Prepared following the **general procedure E** outlined above using Ir[dF(CF_3)ppy]$_2$(dtbbpy)PF_6 (2.2 mg, 2 mmol%), glycosyl donor **S11** (0.1 mmol), 2-

vinylpyridine (16.2 μL, 0.15 mmol, 1.5 equiv.), DIPEA (19.2 μL, 0.2 mmol, 2.0 equiv.), and MeCN (1 mL). Purification by flash chromatography (PE:EA = 3:1) provided the title compound (53.5 mg, 85% yield) as a colorless oil.

^{1}H NMR (400 MHz, CDCl$_3$) δ 8.52 – 8.46 (m, 1H), 7.56 – 7.49 (m, 1H), 7.34 – 7.18 (m, 18H), 7.12 – 7.01 (m, 4H), 4.88 (t, *J* = 10.8 Hz, 1H), 4.78 (dd, *J* = 16.3, 10.9 Hz, 2H), 4.65 – 4.53 (m, 2H), 4.46 (dd, *J* = 11.5, 9.3 Hz, 2H), 4.05 – 4.00 (m, 1H), 3.83 – 3.75 (m, 1H), 3.74 – 3.54 (m, 6H), 2.97 – 2.86 (m, 1H), 2.82 – 2.72 (m, 1H).

^{13}C NMR (100 MHz, CDCl$_3$) δ 161.62, 149.26, 138.86, 138.36, 138.16, 136.47, 128.48, 128.45, 128.03, 128.00, 127.95, 127.80, 127.74, 127.68, 123.26, 121.17, 82.66, 80.18, 78.31, 75.62, 75.15, 73.68, 73.61, 73.08, 71.18, 69.14, 34.08, 24.90.

HRMS (ESI) m/z calcd. for $C_{41}H_{43}NO_5$ ($[M+H]^+$) 630.3219, found: 630.3210.

((2R,3S,4R,5R,6R))-3,4-Bis(benzyloxy)-2-((benzyloxy)methyl)-6-methoxy-5-(2-(pyridin-2-yl)ethyl)tetrahydro-2H-pyran (16)

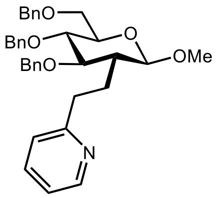

Prepared following the **general procedure E** outlined above using Ir[dF(CF$_3$)ppy]$_2$(dtbbpy)PF$_6$ (2.2 mg, 2 mmol%), glycosyl donor **S12** (0.1 mmol), 2-vinylpyridine (16.2 μL, 0.15 mmol, 1.5 equiv.), DIPEA (19.2 μL, 0.2 mmol, 2.0 equiv.), and MeCN (1 mL). Purification by flash chromatography (PE:EA = 3:1) provided the title compound (18.8 mg, 34% yield) as a colorless oil.

^{1}H NMR (400 MHz, CDCl$_3$) δ 8.47 – 8.41 (m, 1H), 7.50 (dd, *J* = 1.9, 7.7 Hz, 1H), 7.32 – 7.24 (m, 5H), 7.24 – 7.20 (m, 9H), 7.14 – 7.09 (m, 2H), 7.02 (d, *J* = 7.7 Hz, 1H), 4.83 (d, *J* = 11.0 Hz, 1H), 4.71 (d, *J* = 10.8 Hz, 1H), 4.62 (d, *J* = 11.0 Hz, 1H), 4.57 (d, *J* = 12.2 Hz, 1H), 4.50 (d, *J* = 11.6 Hz, 2H), 4.12 (d, *J* = 8.5 Hz, 1H), 3.68 (dd, *J* = 2.7, 4.3 Hz, 2H),

3.57 – 3.50 (m, 1H), 3.42 (s, 3H), 2.89 (dd, *J* = 5.3, 10.1 Hz, 1H), 2.87 – 2.76 (m, 1H), 1.98 -1.94 (m, 1H), 1.88 – 1.81 (m, 1H), 1.77 – 1.71 (m, 1H).

^{13}C NMR (100 MHz, $CDCl_3$) δ 138.38, 138.23, 138.10, 128.46, 128.42, 128.37, 127.89, 127.83, 127.79, 127.67, 127.61, 123.12, 121.18, 104.78, 83.04, 75.10, 75.05, 74.72, 73.53, 69.12, 56.81, 46.64, 35.29, 29.72, 27.15, 18.45.

HRMS (ESI) m/z calcd. for $C_{35}H_{39}NO_5$ ($[M+H]^+$) 554.2906, found: 554.2897.

2-(2-((2R,4aR,6S,8R,8aS)-6-Methoxy-2-phenylhexahydropyrano[3,2-d][1,3]dioxin-8-yl)ethyl)pyridine (17)

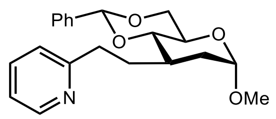

Prepared following the **general procedure E** outlined above using $Ir[dF(CF_3)ppy]_2(dtbbpy)PF_6$ (2.2 mg, 2 mmol%), glycosyl donor **S13** (0.1 mmol), 2-vinylpyridine (16.2 μL, 0.15 mmol, 1.5 equiv.), DIPEA (19.2 μL, 0.2 mmol, 2.0 equiv.), and MeCN (1 mL). Purification by flash chromatography (PE:EA = 3:1) provided the title compound (22.7 mg, 64% yield) as a colorless oil.

^{1}H NMR (400 MHz, $CDCl_3$) δ 8.43 (dd, *J* = 1.9, 5.0 Hz, 1H), 7.49 (dd, *J* = 1.9, 7.7 Hz, 1H), 7.42 (d, *J* = 2.1 Hz, 1H), 7.40 (d, *J* = 1.8 Hz, 1H), 7.31 – 7.28 (m, 1H), 7.27 (p, *J* = 1.1 Hz, 2H), 7.06 (dd, *J* = 1.1, 7.9 Hz, 1H), 7.01 – 6.95 (m, 1H), 5.45 (s, 1H), 4.66 (dd, *J* = 1.2, 3.7 Hz, 1H), 4.16 (dd, *J* = 4.6, 10.0 Hz, 1H), 3.77 – 3.70 (m, 1H), 3.63 (t, *J* = 10.3 Hz, 1H), 3.29 (s, 3H), 2.81 (s, 1H), 2.71 (s, 1H), 2.22 – 2.07 (m, 3H), 2.06 – 1.98 (m, 1H), 1.56 – 1.44 (m, 2H).

^{13}C NMR (100 MHz, $CDCl_3$) δ 161.97, 148.92, 137.81, 136.67, 128.89, 128.23, 126.15, 122.99, 121.13, 101.74, 98.24, 82.79, 69.47, 64.09, 54.68, 35.34, 35.17, 34.21, 31.89.

HRMS (ESI) m/z calcd. for $C_{21}H_{25}NO_4$ ($[M+H]^+$) 356.1862, found: 356.1853.

(2R,3R,4S,5R,6S)-6-((Benzoyloxy)methyl)-2-(4-methoxyphenoxy)-5-(2-(pyridin-2-yl)ethyl)tetrahydro-2H-pyran-3,4-diyl dibenzoate (18)

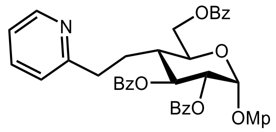

Prepared following the **general procedure E** outlined above using Ir[dF(CF_3)ppy]$_2$(dtbbpy)PF_6 (2.2 mg, 2 mmol%), glycosyl donor **S14** (0.1 mmol), 2-vinylpyridine (16.2 μL, 0.15 mmol, 1.5 equiv.), DIPEA (19.2 μL, 0.2 mmol, 2.0 equiv.), and MeCN (1 mL). Purification by flash chromatography (PE:EA = 3:1) provided the title compound (32.2 mg, 47% yield) as a colorless oil.

^{1}H NMR (400 MHz, $CDCl_3$) δ 8.39 (dd, *J* = 1.8, 5.1 Hz, 1H), 8.11 – 8.03 (m, 2H), 7.99 – 7.88 (m, 4H), 7.59 (d, *J* = 7.4 Hz, 1H), 7.55 – 7.42 (m, 5H), 7.36 (dd, *J* = 7.7, 12.6 Hz, 4H), 7.03 (t, *J* = 7.9 Hz, 2H), 6.98 – 6.86 (m, 2H), 6.64 – 6.57 (m, 2H), 5.74 (dd, *J* = 9.4, 10.8 Hz, 1H), 5.60 (dd, *J* = 7.8, 9.4 Hz, 1H), 5.12 (d, *J* = 7.8 Hz, 1H), 4.83 (dd, *J* = 2.4, 12.0 Hz, 1H), 4.50 (dd, *J* = 7.0, 11.9 Hz, 1H), 4.05 (s, 1H), 3.68 (s, 3H), 3.07 – 2.95 (m, 1H), 2.86 (s, 1H), 2.43 – 2.31 (m, 1H), 2.09 – 2.01 (m, 1H)

^{13}C NMR (100 MHz, $CDCl_3$) δ 166.27, 166.21, 165.48, 160.63, 155.49, 151.22, 149.22, 136.59, 133.36, 133.23, 133.15, 129.88, 129.85, 129.74, 128.45, 128.36, 122.97, 121.36, 118.85, 114.35, 100.68, 73.18, 64.45, 62.27, 55.55, 41.52, 33.94, 26.77, 14.43, 14.25, 14.16

HRMS (ESI) m/z calcd. for $C_{34}H_{30}NO_7$ ($[M+H]^+$) 688.2541, found: 688.2537.

(2R,3R,4S,5R,6S)-6-((Benzoyloxy)methyl)-2-(4-methoxyphenoxy)-5-(2-(pyridin-2-yl)ethyl)tetrahydro-2H-pyran-3,4-diyl dibenzoate (19)

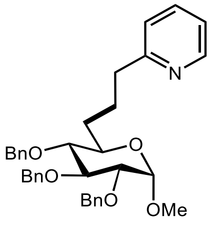

Prepared following the **general procedure E** outlined above using Ir[dF(CF_3)ppy]$_2$(dtbbpy)PF_6 (2.2 mg, 2 mmol%), glycosyl donor **S17** (0.1 mmol), 2-vinylpyridine (16.2 μL, 0.15 mmol, 1.5 equiv.), DIPEA (19.2 μL, 0.2 mmol, 2.0 equiv.), and MeCN (1 mL). Purification by flash chromatography (PE:EA = 3:1) provided the title compound (24.3 mg, 44% yield) as a colorless oil.

^{1}H NMR (400 MHz, CDCl$_3$) δ 8.47 – 8.56 (m, 1H), 7.56 (d, *J* = 1.9 Hz, 1H), 7.53 (dd, *J* = 1.9, 10.8 Hz, 1H), 7.46 – 7.34 (m, 2H), 7.27 – 7.18 (m, 4H), 7.17 – 7.09 (m, 2H), 7.02 (dd, *J* = 1.1, 7.9 Hz, 1H), 6.95 (d, *J* = 7.9 Hz, 1H), 5.09 (d, *J* = 11.2 Hz, 1H), 4.91 – 4.85 (m, 2H), 4.86 – 4.80 (m, 1H), 4.75 – 4.69 (m, 1H), 4.60 (dd, *J* = 9.9, 12.1 Hz, 2H), 4.50 (d, *J* = 3.6 Hz, 1H), 4.47 – 4.39 (m, 2H), 4.00 – 3.93 (m, 1H), 3.83 (dd, *J* = 8.8, 9.7 Hz, 1H), 3.81 – 3.76 (m, 1H), 3.63 (dd, *J* = 6.3, 9.5 Hz, 1H), 3.47 (dd, *J* = 3.6, 9.6 Hz, 2H), 3.35 (s, 2H), 3.26 (t, *J* = 9.1 Hz, 1H), 3.05 (t, *J* = 9.1 Hz, 1H), 2.69 – 2.77 (m, 1H), 2.62 (dd, *J* = 6.9, 9.2 Hz, 2H), 2.44 – 2.29 (m, 2H).

^{13}C NMR (100 MHz, CDCl$_3$) δ 161.53, 149.02, 142.05, 141.22, 139.10, 138.18, 136.44, 128.42, 128.40, 128.37, 128.24, 128.17, 128.15, 128.08, 127.88, 127.84, 127.60, 127.41, 127.39, 127.30, 127.12, 126.91, 122.77, 121.04, 97.86, 97.65, 82.46, 82.42, 81.78, 81.70, 80.66, 80.36, 75.04, 73.26, 73.23, 66.69, 66.42, 60.42, 55.06, 54.99, 36.82, 36.45, 33.59, 21.07, 18.69, 17.95, 14.22.

HRMS (ESI) m/z calcd. for $C_{35}H_{39}NO_5$ ($[M+H]^+$) 554.2906, found: 554.2898.

(2R,3R,4R,5S,6R)-2-(Acetoxymethyl)-6-(2-(5-fluoropyridin-2-yl)ethyl)tetrahydro-2H-pyran-3,4,5-triyl triacetate (20)

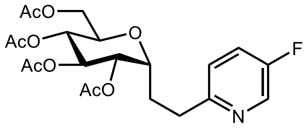

Prepared following the **general procedure E** outlined above using Ir[dF(CF_3)ppy]$_2$(dtbbpy)PF_6 (2.2 mg, 2 mmol%), glycosyl donor **1h** (0.1 mmol), alkene (0.15 mmol, 1.5 equiv.), DIPEA (19.2 μL, 0.2 mmol, 2.0 equiv.), and MeCN (1 mL).

Purification by flash chromatography (PE:EA = 3:1) provided the title compound (38.2 mg, 84% yield) as a colorless oil.

^{1}H NMR (400 MHz, CDCl$_3$) δ 8.32 (d, *J* = 3.2 Hz, 1H), 7.26 (dd, *J* = 8.5, 3.0 Hz, 1H), 7.13 – 7.04 (m, 1H), 5.28 (dd, *J* = 9.4, 9.4 Hz, 1H), 5.02 (dd, *J* = 9.6, 5.9 Hz, 1H), 4.93 (dd, *J* = 9.3, 9.3 Hz, 1H), 4.21 – 4.08 (m, 2H), 3.98 (dd, *J* = 12.0, 2.7 Hz, 1H), 3.83 – 3.78 (m, 1H), 2.85 (s, 1H), 2.71 (s, 1H), 2.27 – 2.10 (m, 1H), 2.02 (s, 3H), 1.97 (s, 3H), 1.96 (s, 3H), 1.94 (s, 3H), 1.89 – 1.84 (m, 1H).

^{13}C NMR (100 MHz, CDCl$_3$) δ 169.75, 169.24, 168.78, 168.67, 155.60, 155.56, 136.63, 136.40, 122.91 (d, *J* = 3.9 Hz), 122.53, 122.35, 71.37, 69.63, 69.52, 68.11, 67.83, 61.52, 31.71, 24.29, 19.86, 19.84, 19.79, 19.75.

^{19}F NMR (376 MHz, CDCl$_3$) δ -130.45 (s, 1F).

HRMS (ESI) m/z calcd. for $C_{21}H_{26}FNO_9$ ($[M+H]^+$) 456.1670, found: 456.1663.

(2R,3R,4R,5S,6R)-2-(Acetoxymethyl)-6-(2-(5-(trifluoromethyl)pyridin-2-yl)ethyl)tetrahydro-2H-pyran-3,4,5-triyl triacetate (21)

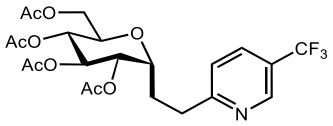

Prepared following the **general procedure E** outlined above using Ir[dF(CF$_3$)ppy]$_2$(dtbbpy)PF$_6$ (2.2 mg, 2 mmol%), glycosyl donor **1h** (0.1 mmol), alkene (0.15 mmol, 1.5 equiv.), DIPEA (19.2 μL, 0.2 mmol, 2.0 equiv.), and MeCN (1 mL). Purification by flash chromatography (PE:EA = 3:1) provided the title compound (46.0 mg, 91% yield) as a colorless oil.

^{1}H NMR (400 MHz, CDCl$_3$) δ 8.78 – 8.74 (m, 1H), 7.84 – 7.78 (m, 1H), 7.27 (d, *J* = 8.1 Hz, 1H), 5.30 (t, *J* = 9.3 Hz, 1H), 5.06 (ddd, *J* = 9.6, 5.9, 2.6 Hz, 1H), 4.96 (t, *J* = 9.2 Hz,

1H), 4.23 – 4.15 (m, 2H), 4.01 (dd, *J* = 12.1, 2.6 Hz, 1H), 3.95 – 3.84 (m, 1H), 2.97 (s, 1H), 2.82 (s, 1H), 2.31 – 2.12 (m, 2H), 2.05 (s, 3H), 2.00 (s, 3H), 1.99 (s, 3H), 1.98 (s, 3H).

^{13}C NMR (100 MHz, $CDCl_3$) δ 170.72, 170.23, 169.77, 169.66, 164.73, 146.41 (q, *J* = 4.0 Hz), 133.72, 124.80 (q, *J* = 214.6 Hz), 122.98, 72.23, 70.46, 70.37, 68.96, 68.82, 62.41, 33.51, 24.94, 20.83, 20.81, 20.77, 20.73.

^{19}F NMR (376 MHz, $CDCl_3$) δ -62.29 (s, 3F).

HRMS (ESI) m/z calcd. for $C_{22}H_{26}F_3NO_9$ ($[M+H]^+$) 506.1638, found: 506.1634.

(2R,3R,4R,5S,6R)-2-(Acetoxymethyl)-6-(2-(pyridin-4-yl)ethyl)tetrahydro-2H-pyran-3,4,5-triyl triacetate (22)

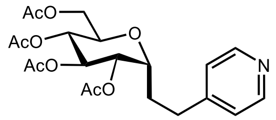

Prepared following the **general procedure E** outlined above using Ir[dF(CF_3)ppy]$_2$(dtbbpy)PF_6 (2.2 mg, 2 mmol%), glycosyl donor **1h** (0.1 mmol), alkene (0.15 mmol, 1.5 equiv.), DIPEA (19.2 μL, 0.2 mmol, 2.0 equiv.), and MeCN (1 mL). Purification by flash chromatography (PE:EA = 3:1) provided the title compound (41.0 mg, 94% yield) as a colorless oil.

^{1}H NMR (400 MHz, $CDCl_3$) δ 8.54 – 8.46 (m, 2H), 7.15 – 7.09 (m, 2H), 5.29 (t, *J* = 9.0 Hz, 1H), 5.07 (dd, *J* = 9.3, 5.7 Hz, 1H), 4.97 (t, *J* = 9.0 Hz, 1H), 4.24 (dd, *J* = 12.1, 5.6 Hz, 1H), 4.15 (ddd, *J* = 11.7, 5.7, 3.5 Hz, 1H), 4.08 (dd, *J* = 12.1, 2.7 Hz, 1H), 3.81 – 3.93 (m, 1H), 2.83 – 2.74 (m, 1H), 2.63 – 2.57 (m, 1H), 2.19 – 2.11 (m, 1H), 2.08 (s, 3H), 2.02 (s, 6H), 2.01 (s, 3H).

^{13}C NMR (100 MHz, $CDCl_3$) δ 170.65, 170.15, 169.63, 169.59, 149.99, 123.93, 71.80, 70.41, 70.28, 69.15, 68.97, 62.50, 30.62, 26.45, 20.83, 20.77, 20.76, 20.73.

HRMS (ESI) m/z calcd. for $C_{21}H_{27}NO_9$ ([M+H]$^+$) 438.1764, found: 438.1757.

(2R,3R,4R,5S,6R)-2-(Acetoxymethyl)-6-(2-(2-fluoropyridin-4-yl)ethyl)tetrahydro-2H-pyran-3,4,5-triyl triacetate (23)

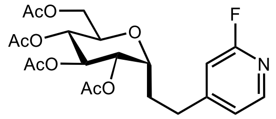

Prepared following the **general procedure E** outlined above using Ir[dF(CF$_3$)ppy]$_2$(dtbbpy)PF$_6$ (2.2 mg, 2 mmol%), glycosyl donor **1h** (0.1 mmol), alkene (0.15 mmol, 1.5 equiv.), DIPEA (19.2 μL, 0.2 mmol, 2.0 equiv.), and MeCN (1 mL). Purification by flash chromatography (PE:EA = 3:1) provided the title compound (37.8 mg, 83% yield) as a colorless oil.

^{1}H NMR (400 MHz, CDCl$_3$) δ 8.13 (d, *J* = 5.1 Hz, 1H), 7.06 – 6.96 (m, 1H), 6.77 (s, 1H), 5.29 (t, *J* = 8.9 Hz, 1H), 5.08 (dd, *J* = 9.2, 5.6 Hz, 1H), 4.97 (t, *J* = 8.9 Hz, 1H), 4.26 (dd, *J* = 12.1, 5.7 Hz, 1H), 4.15 (ddd, *J* = 11.6, 5.6, 3.5 Hz, 1H), 4.10 (dd, *J* = 12.1, 2.8 Hz, 1H), 3.91 – 3.80 (m, 1H), 2.83 – 2.77 (m, 1H), 2.69 – 2.61 (m, 1H), 2.21 – 2.10 (m, 1H), 2.10 (s, 3H), 2.04 (s, 3H), 2.04 (s, 3H), 2.02 (s, 3H).

^{13}C NMR (100 MHz, CDCl$_3$) δ 169.67, 169.15, 168.65, 168.61, 163.32 (d, *J* = 238.9 Hz), 154.98, 146.93, 120.70, 108.51, 70.62, 69.34, 69.23, 68.29, 67.94, 61.52, 29.51, 25.39, 19.86, 19.80, 19.79, 19.76.

^{19}F NMR (376 MHz, CDCl$_3$) δ -68.47 (s, 1F).

HRMS (ESI) m/z calcd. for $C_{21}H_{26}FNO_9$ ([M+H]$^+$) 456.1670, found: 456.1667.

(2R,3R,4R,5S,6R)-2-(Acetoxymethyl)-6-(2-(2,6-dimethylpyridin-4-yl)ethyl)tetrahydro-2H-pyran-3,4,5-triyl triacetate (24)

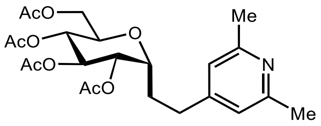

Prepared following the **general procedure E** outlined above using Ir[dF(CF_3)ppy]$_2$(dtbbpy)PF_6 (2.2 mg, 2 mmol%), glycosyl donor **1h** (0.1 mmol), alkene (0.15 mmol, 1.5 equiv.), DIPEA (19.2 µL, 0.2 mmol, 2.0 equiv.), and MeCN (1 mL). Purification by flash chromatography (PE:EA = 3:1) provided the title compound (20.0 mg, 43% yield) as a colorless oil.

^{1}H NMR (400 MHz, $CDCl_3$) δ 6.79 (s, 2H), 5.29 (t, *J* = 9.1 Hz, 1H), 5.07 (dd, *J* = 9.5, 5.7 Hz, 1H), 4.97 (t, *J* = 9.0 Hz, 1H), 4.24 (dd, *J* = 12.1, 5.5 Hz, 1H), 4.19 – 4.10 (m, 1H), 4.08 (dd, *J* = 12.2, 2.7 Hz, 1H), 3.84 (ddd, *J* = 8.7, 5.5, 2.7 Hz, 1H), 2.68 (dd, *J* = 10.1, 5.1 Hz, 1H), 2.52 – 2.49 (m, 7H), 2.08 (s, 3H), 2.06 – 2.04 (m, 1H), 2.02 (s, 6H), 2.01 (s, 3H), 1.79 – 1.71 (m, 1H).

^{13}C NMR (100 MHz, $CDCl_3$) δ 170.72, 170.24, 169.69, 169.62, 157.76, 150.92, 120.64, 71.93, 70.38, 70.24, 68.98, 68.90, 62.43, 30.48, 26.39, 24.17, 20.86, 20.81, 20.78, 20.75.

HRMS (ESI) m/z calcd. for $C_{23}H_{31}NO_9$ ($[M+H]^+$) 466.2077, found: 466.2068.

(2R,3R,4R,5S,6R)-2-(Acetoxymethyl)-6-(2-(2-chloropyridin-3-yl)ethyl)tetrahydro-2H-pyran-3,4,5-triyl triacetate (25)

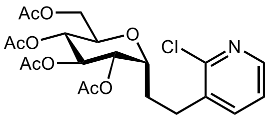

Prepared following the **general procedure E** outlined above using Ir[dF(CF_3)ppy]$_2$(dtbbpy)PF_6 (2.2 mg, 2 mmol%), glycosyl donor **1h** (0.1 mmol), alkene (0.15 mmol, 1.5 equiv.), DIPEA (19.2 µL, 0.2 mmol, 2.0 equiv.), and MeCN (1 mL). Purification by flash chromatography (PE:EA = 3:1) provided the title compound (33.4 mg, 71% yield) as a colorless oil.

^{1}H NMR (400 MHz, CDCl$_3$) δ 8.28 (dd, *J* = 4.8, 1.9 Hz, 1H), 7.58 (dd, *J* = 7.4, 2.0 Hz, 1H), 7.20 (dd, *J* = 7.5, 4.7 Hz, 1H), 5.30 (dd, 1H), 5.10 (dd, *J* = 9.7, 5.9 Hz, 1H), 4.99 (t, *J* = 9.2 Hz, 1H), 4.27 – 4.17 (m, 2H), 4.11 (dd, *J* = 12.1, 2.6 Hz, 1H), 3.92 (ddd, *J* = 8.6, 5.6, 2.5 Hz, 1H), 2.96 – 2.90 (m, 1H), 2.65 – 2.58 (m, 1H), 2.22 – 2.07 (m, 4H), 2.04 (s, 3H), 2.03 (s, 3H), 2.01 (s, 3H), 1.82 -1.77 (m, 1H).

^{13}C NMR (100 MHz, CDCl$_3$) δ 170.74, 170.20, 169.72, 169.67, 151.29, 147.93, 139.25, 135.19, 122.90, 72.25, 70.53, 70.41, 69.10, 69.04, 62.60, 29.17, 25.01, 20.88, 20.80, 20.79, 20.77.

HRMS (ESI) m/z calcd. for $C_{21}H_{26}ClNO_9$ ($[M+H]^+$) 472.1374, found: 472.1368.

(2R,3R,4R,5S,6R)-2-(Acetoxymethyl)-6-(2,2-di(pyridin-2-yl)ethyl)tetrahydro-2H-pyran-3,4,5-triyl triacetate (26)

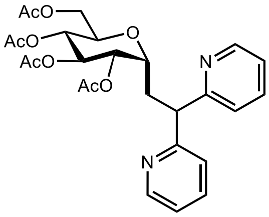

Prepared following the **general procedure E** outlined above using Ir[dF(CF$_3$)ppy]$_2$(dtbbpy)PF$_6$ (2.2 mg, 2 mmol%), glycosyl donor **1h** (0.1 mmol), alkene (0.15 mmol, 1.5 equiv.), DIPEA (19.2 μL, 0.2 mmol, 2.0 equiv.), and MeCN (1 mL). Purification by flash chromatography (PE:EA = 3:1) provided the title compound (28.2 mg, 55% yield) as a colorless oil.

^{1}H NMR (400 MHz, CDCl$_3$) δ 8.57 – 8.54 (m, 2H), 7.62 (m, 2H), 7.34 (t, *J* = 7.6 Hz, 2H), 7.15 (m, 2H), 5.37 (dd, 1H), 5.05 (dd, *J* = 9.9, 6.0 Hz, 1H), 5.00 (t, *J* = 9.4 Hz, 1H), 4.45 (dd, *J* = 8.9, 5.8 Hz, 1H), 4.18 – 4.14 (m, 1H), 4.11 (m, 1H), 3.92 (m, 1H), 3.80 (dd, *J* = 12.2, 2.5 Hz, 1H), 2.72 – 2.62 (m, 1H), 2.10 (s, 3H), 2.07 (s, 3H), 2.05 – 2.03 (m, 1H), 2.01 (s, 3H), 2.00 (s, 3H).

^{13}C NMR (100 MHz, CDCl$_3$) δ 170.74, 170.23, 169.71, 169.57, 149.30, 124.14, 123.18, 122.10, 122.00, 71.66, 70.65, 70.21, 68.76, 68.69, 62.06, 29.26, 20.82, 20.81, 20.70, 20.65.

HRMS (ESI) m/z calcd. for $C_{26}H_{30}N_2O_9$ ($[M+H]^+$) 515.2029, found: 515.2024.

(2R,3R,4R,5S,6R)-2-(Acetoxymethyl)-6-(2-(isoquinolin-1-yl)ethyl)tetrahydro-2H-pyran-3,4,5-triyl triacetate (27)

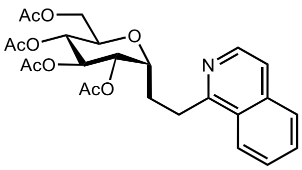

Prepared following the **general procedure E** outlined above using Ir[dF(CF$_3$)ppy]$_2$(dtbbpy)PF$_6$ (2.2 mg, 2 mmol%), glycosyl donor **1h** (0.1 mmol), alkene (0.15 mmol, 1.5 equiv.), DIPEA (19.2 μL, 0.2 mmol, 2.0 equiv.), and MeCN (1 mL). Purification by flash chromatography (PE:EA = 3:1) provided the title compound (31.7 mg, 65% yield) as a colorless oil.

^{1}H NMR (400 MHz, CDCl$_3$) δ 8.44 (d, *J* = 5.7 Hz, 1H), 8.17 – 8.12 (m, 1H), 7.84 (d, *J* = 8.0 Hz, 1H), 7.70 – 7.66 (m, 1H), 7.61 – 7.57 (tm, 1H), 7.54 (d, *J* = 5.8 Hz, 1H), 5.38 (t, *J* = 9.3 Hz, 1H), 5.15 (dd, *J* = 9.7, 5.9 Hz, 1H), 5.02 (t, *J* = 9.3 Hz, 1H), 4.39 (m, 1H), 4.25 (dd, *J* = 12.1, 5.4 Hz, 1H), 4.06 (dd, *J* = 12.1, 2.5 Hz, 1H), 3.98 (m, 1H), 3.47 (dd, *J* = 10.3, 5.3 Hz, 1H), 3.29 (m 1H), 2.46 – 2.36 (m, 1H), 2.24 – 2.17 (m, 1H), 2.08 (s, 3H), 2.03 (s, 3H), 2.03 (s, 3H), 2.01 (s, 3H).

^{13}C NMR (100 MHz, CDCl$_3$) δ 170.76, 170.21, 169.86, 169.69, 160.38, 141.88, 136.36, 130.15, 127.71, 127.46, 127.13, 124.81, 119.69, 73.05, 70.69, 70.62, 69.18, 68.96, 62.71, 30.15, 24.36, 20.90, 20.86, 20.80, 20.76.

HRMS (ESI) m/z calcd. for $C_{25}H_{29}NO_9$ ($[M+H]^+$) 488.1920, found: 488.1914.

(2R,3R,4R,5S,6R)-2-(Acetoxymethyl)-6-(2-(isoquinolin-4-yl)ethyl)tetrahydro-2H-pyran-3,4,5-triyl triacetate (28)

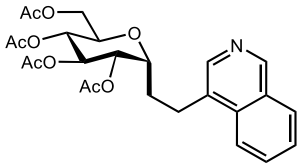

Prepared following the **general procedure E** outlined above using Ir[dF(CF_3)ppy]$_2$(dtbbpy)PF_6 (2.2 mg, 2 mmol%), glycosyl donor **1h** (0.1 mmol), alkene (0.15 mmol, 1.5 equiv.), DIPEA (19.2 μL, 0.2 mmol, 2.0 equiv.), and MeCN (1 mL). Purification by flash chromatography (PE:EA = 3:1) provided the title compound (22.9 mg, 47% yield) as a colorless oil.

^{1}H NMR (400 MHz, CDCl$_3$) 9.16 (s, 1H), 8.42 (s, 1H), 8.06 – 7.99 (m, 1H), 7.98 (d, J = 8.5 Hz, 1H), 7.77 (m, 1H), 7.64 (m, 1H), 5.27 (t, J = 9.0 Hz, 1H), 5.10 (dd, J = 5.6, 9.4 Hz, 1H), 4.99 (t, J = 9.0 Hz, 1H), 4.29 (dd, J = 5.9, 12.0 Hz, 1H), 4.14 (dd, J = 2.6, 12.2 Hz, 1H), 3.94 -3.88 (m, 1H), 3.22 – 3.15 (m, 1H), 3.09 – 2.94 (m, 1H), 2.12 (s, 3H), 2.04 (s, 3H), 2.01 (s, 3H), 1.99 (s,3H).

^{13}C NMR (100 MHz, CDCl$_3$) δ 170.74, 170.12, 169.67, 169.64, 151.78, 142.70, 134.70, 130.81, 128.75, 127.25, 122.53, 72.31, 70.45, 70.34, 69.33, 69.00, 62.67, 20.89, 20.76, 20.73.

HRMS (ESI) m/z calcd. for $C_{25}H_{29}NO_9$ ([M+H]$^+$) 488.1920, found: 488.1912.

(2R,3R,4R,5S,6R)-2-(Acetoxymethyl)-6-(2-(quinolin-4-yl)ethyl)tetrahydro-2H-pyran-3,4,5-triyl triacetate (29)

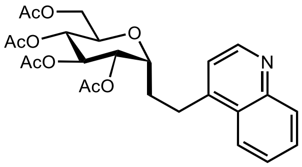

Prepared following the **general procedure E** outlined above using $Ir[dF(CF_3)ppy]_2(dtbbpy)PF_6$ (2.2 mg, 2 mmol%), glycosyl donor **1h** (0.1 mmol), alkene (0.15 mmol, 1.5 equiv.), DIPEA (19.2 μL, 0.2 mmol, 2.0 equiv.), and MeCN (1 mL). Purification by flash chromatography (PE:EA = 3:1) provided the title compound (24.8 mg, 51% yield) as a colorless oil.

^{1}H NMR (400 MHz, CDCl$_3$) δ 8.84 (d, *J* = 4.6 Hz, 1H), 8.16 (d, *J* = 8.4 Hz, 1H), 8.03 (d, *J* = 8.4 Hz, 1H), 7.75 – 7.71 (m, 1H), 7.60 – 7.53 (m, 1H), 7.29 (d, *J* = 4.4 Hz, 1H), 5.30 (t, *J* = 9.0 Hz, 1H), 5.12 (dd, *J* = 9.3, 5.7 Hz, 1H), 5.00 (t, *J* = 8.9 Hz, 1H), 4.32 – 4.27 (m, 2H), 4.14 (dd, *J* = 12.0, 2.8 Hz, 1H), 3.94 – 3.87 (m, 1H), 3.30 – 3.21 (m, 1H), 3.07 – 2.99 (m, 1H), 2.32 – 2.20 (m, 1H), 2.11 (s, 3H), 2.04 (s, 3H), 2.02 (s, 3H), 2.00 (s, 3H), 1.96 – 1.93 (m, 1H).

^{13}C NMR (100 MHz, CDCl$_3$) δ 170.71, 170.14, 169.66, 169.63, 150.26, 148.44, 147.08, 130.57, 129.45, 127.46, 126.84, 123.16, 120.97, 72.27, 70.42, 70.33, 69.36, 69.00, 62.65, 27.59, 26.10, 21.21, 20.89, 20.77, 20.68.

HRMS (ESI) m/z calcd. for $C_{25}H_{29}NO_9$ ($[M+H]^+$) 488.1920, found: 488.1913.

(2R,3R,4R,5S,6R)-2-(Acetoxymethyl)-6-(2-(pyrimidin-5-yl)ethyl)tetrahydro-2H-pyran-3,4,5-triyl triacetate (30)

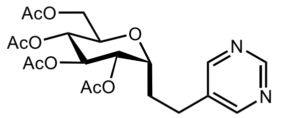

Prepared following the **general procedure E** outlined above using $Ir[dF(CF_3)ppy]_2(dtbbpy)PF_6$ (2.2 mg, 2 mmol%), glycosyl donor **1h** (0.1 mmol), alkene (0.15 mmol, 1.5 equiv.), DIPEA (19.2 μL, 0.2 mmol, 2.0 equiv.), and MeCN (1 mL). Purification by flash chromatography (PE:EA = 3:1) provided the title compound (20.1 mg, 46% yield) as a colorless oil.

^{1}H NMR (400 MHz, CDCl$_3$) δ 9.12 (s, 1H), 8.63 (s, 2H), 5.30 (t, *J* = 8.8 Hz, 1H), 5.10 (dd, *J* = 9.1, 5.6 Hz, 1H), 4.99 (t, *J* = 8.8 Hz, 1H), 4.28 (dd, *J* = 12.1, 5.8 Hz, 1H), 4.17 (dd, *J* = 11.8, 5.5, 1H), 4.11 (dd, *J* = 12.1, 2.8 Hz, 1H), 3.89 (ddd, *J* = 8.8, 5.7, 2.8 Hz, 1H), 2.80 (dd, *J* = 9.8, 4.9 Hz, 1H), 2.65 – 2.60 (m, 1H), 2.12 (s, 3H), 2.06 (s, 6H), 2.04 (s, 3H), 1.91 – 1.76 (m, 1H).

^{13}C NMR (100 MHz, CDCl$_3$) δ 170.65, 170.09, 169.63, 169.59, 157.27, 156.90, 134.03, 71.44, 70.28, 70.13, 69.39, 68.85, 62.47, 26.97, 25.96, 20.86, 20.79, 20.77, 20.75.

HRMS (ESI) m/z calcd. for $C_{21}H_{26}N_2O_9$ ([M+H]$^+$) 439.1716, found: 439.1716.

(2R,3R,4R,5S,6R)-2-(Acetoxymethyl)-6-(4-cyanophenethyl)tetrahydro-2H-pyran-3,4,5-triyl triacetate (31)

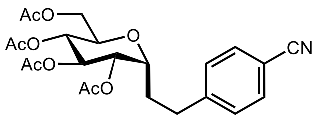

Prepared following the **general procedure E** outlined above using Ir[dF(CF$_3$)ppy]$_2$(dtbbpy)PF$_6$ (2.2 mg, 2 mmol%), glycosyl donor **1h** (0.1 mmol), alkene (0.15 mmol, 1.5 equiv.), DIPEA (19.2 μL, 0.2 mmol, 2.0 equiv.), and MeCN (1 mL). Purification by flash chromatography (PE:EA = 3:1) provided the title compound (33.2 mg, 72% yield) as a colorless oil.

^{1}H NMR (400 MHz, CDCl$_3$) δ 7.60 – 7.57 (m, 2H), 7.32 – 7.28 (m, 2H), 5.29 (t, *J* = 9.0 Hz, 1H), 5.07 (dd, *J* = 9.4, 5.7 Hz, 1H), 4.97 (t, *J* = 9.0 Hz, 1H), 4.25 (dd, *J* = 12.1, 5.5 Hz, 1H), 4.15 – 4.09 (m, 1H), 4.08 (dd, *J* = 12.2, 2.7 Hz, 1H), 3.86 – 3.83 (m, 1H), 2.82 – 2.77 (m, 1H), 2.66 -2.59 (m, 1H), 2.20 – 2.10 (m, 1H), 2.09 (s, 3H), 2.03 (s, 3H), 2.02 (s, 3H), 2.01 (s, 3H), 1.80 -1.73 (m, 1H).

^{13}C NMR (100 MHz, CDCl$_3$) δ 170.62, 170.11, 169.60, 169.56, 146.59, 132.46, 129.35, 118.91, 110.34, 71.64, 70.33, 70.23, 69.06, 68.93, 62.47, 31.32, 26.98, 20.80, 20.74, 20.72, 20.69.

HRMS (ESI) m/z calcd. for $C_{23}H_{27}NO_9$ ($[M+Na]^+$) 484.1584, found: 484.1580.

(2R,3R,4R,5S,6R)-2-(Acetoxymethyl)-6-(4-(methoxycarbonyl)phenethyl)tetrahydro-2H-pyran-3,4,5-triyl triacetate (32)

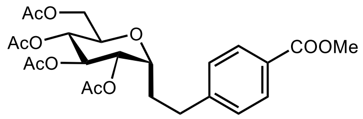

Prepared following the **general procedure E** outlined above using Ir[dF(CF$_3$)ppy]$_2$(dtbbpy)PF$_6$ (2.2 mg, 2 mmol%), glycosyl donor **1h** (0.1 mmol), alkene (0.15 mmol, 1.5 equiv.), DIPEA (19.2 μL, 0.2 mmol, 2.0 equiv.), and MeCN (1 mL). Purification by flash chromatography (PE:EA = 3:1) provided the title compound (39.0 mg, 79% yield) as a colorless oil.

^{1}H NMR (400 MHz, CDCl$_3$) δ 7.98 (d, J = 8.3 Hz, 2H), 7.29 – 7.25 (m, 2H), 5.31 (t, J = 9.1 Hz, 1H), 5.09 (dd, J = 9.4, 5.7 Hz, 1H), 4.99 (t, J = 9.1 Hz, 1H), 4.25 (dd, J = 12.2, 5.5 Hz, 1H), 4.18 (ddd, J = 11.6, 5.7, 3.4 Hz, 1H), 4.08 (dd, J = 12.1, 2.7 Hz, 1H), 3.91 (s, 3H), 3.87 – 3.79 (m, 1H), 2.83 – 2.77 (m, 1H), 2.67 – 2.59 (m, 1H), 2.21 – 2.12 (m, 1H), 2.11 (s, 3H), 2.04 (s, 3H), 2.03 (s, 3H), 2.02 (s, 3H), 1.88 – 1.81 (m, 1H).

^{13}C NMR (100 MHz, CDCl$_3$) δ 170.73, 170.22, 169.68, 169.63, 167.05, 146.43, 129.99, 128.54, 128.36, 71.93, 70.45, 70.28, 68.93, 62.45, 52.14, 31.24, 27.01, 20.85, 20.79, 20.77, 20.74.

HRMS (ESI) m/z calcd. for $C_{24}H_{30}O_{11}$ ($[M+Na]^+$) 517.1686, found: 517.1677.

(2R,3R,4R,5S,6R)-2-(Acetoxymethyl)-6-(4-benzoylphenethyl)tetrahydro-2H-pyran-3,4,5-triyl triacetate (33)

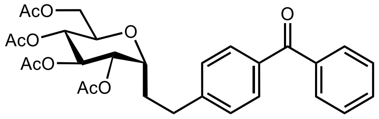

Prepared following the **general procedure E** outlined above using $Ir[dF(CF_3)ppy]_2(dtbbpy)PF_6$ (2.2 mg, 2 mmol%), glycosyl donor **1h** (0.1 mmol), alkene (0.15 mmol, 1.5 equiv.), DIPEA (19.2 μL, 0.2 mmol, 2.0 equiv.), and MeCN (1 mL). Purification by flash chromatography (PE:EA = 3:1) provided the title compound (45.9 mg, 85% yield) as a colorless oil.

^{1}H NMR (400 MHz, CDCl$_3$) δ 7.81 – 7.72 (m, 4H), 7.61 – 7.53 (m, 1H), 7.47 (t, *J* = 7.4 Hz, 2H), 7.30 (d, *J* = 7.8 Hz, 2H), 5.32 (t, *J* = 9.1 Hz, 1H), 5.10 (dd, *J* = 5.8, 9.4 Hz, 1H), 4.99 (t, *J* = 9.0 Hz, 1H), 4.30 – 4.22 (m, 1H), 4.21 – 4.16 (m, 1H), 4.10 (dd, *J* = 2.7, 12.2 Hz, 1H), 3.89 (ddd, *J* = 2.7, 5.6, 8.7 Hz, 1H), 2.93 – 2.80 (m, 1H), 2.77 – 2.67 (m, 1H), 2.20 – 2.12 (m, 1H), 2.10 (s, 3H), 2.03 (s, 6H), 2.01 (s, 3H), 1.91 – 1.77 (m, 1H).

^{13}C NMR (100 MHz, CDCl$_3$) δ 196.38, 170.70, 170.20, 169.67, 169.62, 146.06, 137.91, 135.89, 132.41, 130.64, 130.07, 128.48, 128.38, 71.94, 70.54, 70.38, 69.06, 69.05, 62.58, 31.28, 27.20, 20.85, 20.80, 20.78, 20.74.

HRMS (ESI) m/z calcd. for $C_{29}H_{32}O_{10}$ ($[M+H]^+$) 541.2073, found: 541.2076.

(2R,3R,4R,5S,6R)-2-(Acetoxymethyl)-6-(2-(perfluorophenyl)ethyl)tetrahydro-2H-pyran-3,4,5-triyl triacetate (34)

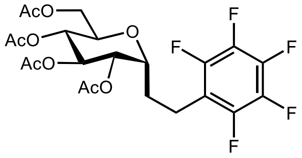

Prepared following the **general procedure E** outlined above using $Ir[dF(CF_3)ppy]_2(dtbbpy)PF_6$ (2.2 mg, 2 mmol%), glycosyl donor **1h** (0.1 mmol), alkene (0.15 mmol, 1.5 equiv.), DIPEA (19.2 μL, 0.2 mmol, 2.0 equiv.), and MeCN (1 mL). Purification by flash chromatography (PE:EA = 3:1) provided the title compound (39.5 mg, 75% yield) as a colorless oil.

^{1}H NMR (400 MHz, CDCl$_3$) δ 5.29 (t, *J* = 9.1 Hz, 1H), 5.14 (dd, *J* = 9.4, 5.7 Hz, 1H), 5.01 (t, *J* = 9.1 Hz, 1H), 4.30 – 4.21 (m, 2H), 4.15 (dd, *J* = 12.2, 2.5 Hz, 1H), 3.89 (dd, *J* = 6.0, 2.9 Hz, 1H), 2.91 – 2.85 (m, 1H), 2.71 – 2.64 (m, 1H), 2.12 (s, 3H), 2.07 (s, 6H), 2.06 – 2.04 (m, 4H), 1.84 -1.77 (m, 1H).

^{13}C NMR (150 MHz, CDCl$_3$) δ 170.79, 170.24, 169.74, 169.69, 146.70 – 145.58 (m), 144.76 – 143.30 (m), 138.63 – 138.39 (m), 137.22 – 136.25 (m), 114.30 – 113.84 (m), 72.14, 70.36, 70.15, 69.18, 68.80, 62.44, 25.29, 20.81, 20.79, 18.20.

^{19}F NMR (376 MHz, CDCl$_3$) δ -144.36 (dd, *J* = 22.3, 8.4 Hz), -156.82 (t, *J* = 20.8 Hz), -162.15 (td, *J* = 21.9, 8.3 Hz).

HRMS (ESI) m/z calcd. for $C_{22}H_{23}F_5O_9$ ($[M+Na]^+$) 549.1160, found: 549.1157.

(2R,3R,4R,5S,6R)-2-(Acetoxymethyl)-6-(3-(((3S,5S,8R,9S,10S,13S,14S)-10,13-dimethyl-17-oxohexadecahydro-1H-cyclopenta[a]phenanthren-3-yl)oxy)-3-oxopropyl)tetrahydro-2H-pyran-3,4,5-triyl triacetate (35)

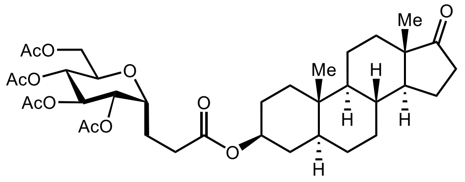

Prepared following the **general procedure E** outlined above using Ir[dF(CF$_3$)ppy]$_2$(dtbbpy)PF$_6$ (2.2 mg, 2 mmol%), glycosyl donor **1h** (0.1 mmol), alkene (0.15 mmol, 1.5 equiv.), DIPEA (19.2 μL, 0.2 mmol, 2.0 equiv.), and MeCN (1 mL). Purification by flash chromatography (PE:EA = 3:1) provided the title compound (39.9 mg, 59% yield) as a colorless oil.

^{1}H NMR (400 MHz, CDCl$_3$) δ 5.26 (t, *J* = 9.2 Hz, 1H), 5.02 (dd, *J* = 9.6, 5.8 Hz, 1H), 4.93 (t, *J* = 9.1 Hz, 1H), 4.64 -4.60 (m, 1H), 4.18 (dd, *J* = 12.2, 5.0 Hz, 1H), 4.11 (dd, *J* = 5.9, 3.3 Hz, 1H), 3.99 (dd, *J* = 12.2, 2.6 Hz, 1H), 3.78 (ddd, *J* = 9.4, 5.0, 2.6 Hz, 1H), 2.41 – 2.21 (m, 4H), 2.03 (s, 4H), 2.00 (s, 3H), 1.97 – 1.95 (m, 6H), 1.90 – 1.80 (m, 2H), 1.78

– 1.70 (m, 4H), 1.70 – 1.64 (m, 1H), 1.63 – 1.57 (m, 1H), 1.55 – 1.52 (m, 1H), 1.50 – 1.40 (m, 3H), 1.33 – 1.23 (m, 4H), 1.17 – 1.08 (m, 2H), 1.02 – 0.90 (m, 2H), 0.79 (s, 6H).

^{13}C NMR (100 MHz, CDCl$_3$) δ 172.36, 170.72, 170.20, 169.72, 169.62, 74.02, 72.22, 70.43, 70.31, 68.97, 68.86, 62.31, 54.46, 51.52, 47.89, 44.80, 36.82, 35.95, 35.79, 35.18, 34.09, 31.67, 30.93, 30.21, 28.41, 27.55, 21.90, 20.85, 20.84, 20.80, 20.74, 20.60, 13.94, 12.34.

HRMS (ESI) m/z calcd. for $C_{36}H_{52}O_{12}$ ($[M+H]^+$) 677.3537, found: 677.3533.

(2R,3R,4R,5S,6R)-2-(Acetoxymethyl)-6-(3-((((1R,4aS,10aR)-7-isopropyl-1,4a-dimethyl-1,2,3,4,4a,9,10,10a-octahydrophenanthren-1-yl)methyl)amino)-3-oxopropyl)tetrahydro-2H-pyran-3,4,5-triyl triacetate (36)

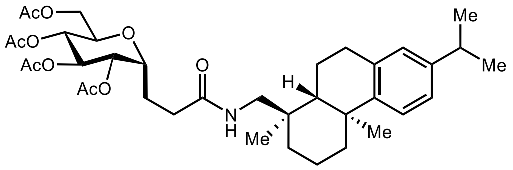

Prepared following the **general procedure E** outlined above using Ir[dF(CF$_3$)ppy]$_2$(dtbbpy)PF$_6$ (2.2 mg, 2 mmol%), glycosyl donor **1h** (0.1 mmol), alkene (0.15 mmol, 1.5 equiv.), DIPEA (19.2 μL, 0.2 mmol, 2.0 equiv.), and MeCN (1 mL). Purification by flash chromatography (PE:EA = 3:1) provided the title compound (33.6 mg, 50% yield) as a colorless oil.

^{1}H NMR (400 MHz, CDCl$_3$) δ 7.16 (d, *J* = 8.2 Hz, 1H), 6.99 (dd, *J* = 2.1, 8.1 Hz, 1H), 6.89 (d, *J* = 2.0 Hz, 1H), 5.56 (t, *J* = 6.4 Hz, 1H), 5.31 (t, *J* = 9.2 Hz, 1H), 5.06 (dd, *J* = 5.8, 9.6 Hz, 1H), 4.97 (t, *J* = 9.2 Hz, 1H), 4.18 (dd, *J* = 4.7, 12.3 Hz, 1H), 4.15 – 4.09 (m, 1H), 4.03 (dd, *J* = 2.7, 12.2 Hz, 1H), 3.85 (ddd, *J* = 2.8, 4.7, 9.5 Hz, 1H), 3.16 (d, *J* = 6.4 Hz, 2H), 2.98 – 2.86 (m, 1H), 2.81-2.79 (m, 2H), 2.36 – 2.23 (m, 2H), 2.21 – 2.12 (m, 1H), 2.04 (s, 3H), 2.03 (s, 3H), 2.02 (s, 3H), 2.01 (s, 3H), 1.87 (dd, *J* = 7.7, 13.4 Hz, 2H), 1.81 – 1.65 (m, 2H), 1.39 -1.34 (m, 3H), 1.22 (s, 3H), 1.21 (s, 7H), 0.93 (s, 3H).

^{13}C NMR (100 MHz, CDCl$_3$) δ 171.91, 170.68, 170.09, 169.76, 169.64, 147.26, 145.83, 134.80, 127.02, 124.23, 124.01, 72.28, 70.49, 70.28, 69.00, 68.91, 62.49, 45.37, 38.47, 37.54, 37.48, 36.30, 33.54, 32.25, 30.20, 25.30, 24.06, 21.43, 20.78, 20.75, 20.71, 19.05, 18.84, 18.68.

HRMS (ESI) m/z calcd. for $C_{37}H_{53}NO_{10}$ ([M+H]$^+$) 672.3747, found: 672.3743.

(2R,3R,4R,5S,6R)-2-(Acetoxymethyl)-6-(3-oxo-3-(((3aR,5aS,8aS,8bR)-2,2,7,7-tetramethyltetrahydro-3aH-bis([1,3]dioxolo)[4,5-b:4',5'-d]pyran-3a-yl)methoxy)propyl)tetrahydro-2H-pyran-3,4,5-triyl triacetate (37)

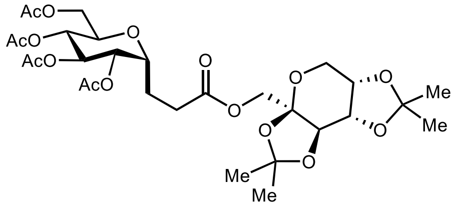

Prepared following the **general procedure E** outlined above using Ir[dF(CF$_3$)ppy]$_2$(dtbbpy)PF$_6$ (2.2 mg, 2 mmol%), glycosyl donor **1h** (0.1 mmol), alkene (0.15 mmol, 1.5 equiv.), DIPEA (19.2 μL, 0.2 mmol, 2.0 equiv.), and MeCN (1 mL). Purification by flash chromatography (PE:EA = 3:1) provided the title compound (43.3 mg, 67% yield) as a colorless oil.

^{1}H NMR (400 MHz, CDCl$_3$) δ 5.24 (t, J = 9.2 Hz, 1H), 5.02 (dd, J = 9.6, 5.8 Hz, 1H), 4.93 (t, J = 9.1 Hz, 1H), 4.53 (dd, J = 7.9, 2.6 Hz, 1H), 4.33 (d, J = 11.6 Hz, 1H), 4.24 – 4.15 (m, 3H), 4.11 (ddd, J = 11.9, 5.7, 3.1 Hz, 1H), 3.99 (d, J = 11.7 Hz, 1H), 3.94 (dd, J = 12.2, 2.6 Hz, 1H), 3.87 – 3.76 (m, 2H), 3.69 (d, J = 13.0 Hz, 1H), 2.59 – 2.24 (m, 2H), 2.14 – 2.03 (m, 1H), 2.01 (s, 3H), 1.99 (s, 3H), 1.96 (s, 3H), 1.95 (s, 3H), 1.83-1.79 (m, 1H), 1.47 (s, 3H), 1.41 (s, 3H), 1.33 (s, 3H), 1.27 (s, 3H).

^{13}C NMR (100 MHz, CDCl$_3$) δ 172.12, 170.57, 170.04, 169.62, 169.53, 116.40, 109.18, 108.79, 101.49, 72.30, 70.79, 70.60, 70.29, 70.14, 70.09, 68.98, 68.64, 65.49, 62.16, 61.30, 29.86, 26.49, 25.90, 25.27, 24.07, 20.71, 20.69, 20.64, 1.86.

HRMS (ESI) m/z calcd. for $C_{29}H_{42}O_{16}$ ([M+Na]$^+$) 669.2371, found: 669.2366.

(2R,3R,4R,5R,6R)-2-(Acetoxymethyl)-6-(3-((3-(ethoxycarbonyl)-4-hydroxyphenyl)amino)-3-oxopropyl)tetrahydro-2H-pyran-3,4,5-triyl triacetate (38)

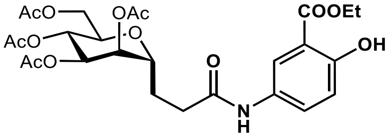

Prepared following the **general procedure E** outlined above using Ir[dF(CF_3)ppy]$_2$(dtbbpy)PF_6 (2.2 mg, 2 mmol%), glycosyl donor **S21** (0.1 mmol), alkene (0.15 mmol, 1.5 equiv.), DIPEA (19.2 μL, 0.2 mmol, 2.0 equiv.), and MeCN (1 mL). Purification by flash chromatography (PE:EA = 3:1) provided the title compound (40.3 mg, 71% yield) as a colorless oil.

^{1}H NMR (400 MHz, $CDCl_3$) δ 10.70 (s, 1H), 8.04 (d, *J* = 2.5 Hz, 1H), 7.56 – 7.48 (m, 2H), 6.94 (d, *J* = 8.9 Hz, 1H), 5.26 (dd, *J* = 3.3, 8.4 Hz, 1H), 5.22 – 5.13 (m, 2H), 4.46 – 4.43 (m, 1H), 4.43 – 4.40 (m, 2H), 4.40 – 4.38 (m, 1H), 4.09 – 3.98 (m, 2H), 4.00 – 3.89 (m, 1H), 2.44 (t, *J* = 7.0 Hz, 2H), 2.24 – 2.16 (m, 1H), 2.11 (s, 3H), 2.08 (s, 3H), 2.07 (s, 3H), 2.03 (s, 3H), 1.41 (t, *J* = 7.1 Hz, 3H).

^{13}C NMR (100 MHz, $CDCl_3$) δ 170.93, 170.37, 170.19, 170.00, 169.90, 169.80, 158.62, 129.52, 128.55, 121.50, 118.04, 112.45, 73.77, 71.15, 70.30, 68.77, 67.13, 62.32, 61.77, 33.14, 24.73, 21.02, 20.91, 20.85, 20.79, 14.28.

HRMS (ESI) m/z calcd. for $C_{26}H_{33}NO_{13}$ ([M+H]$^+$) 568.2030, found: 568.20125.

(2R,3S,4R,5R,6R)-2-(Hydroxymethyl)-6-(2-(pyridin-2-yl)ethyl)tetrahydro-2H-pyran-3,4,5-triol (39)

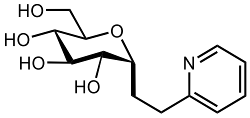

Prepared following the **general procedure F** outlined above using Ir[dF(CF_3)ppy]$_2$(dtbbpy)PF_6 (2.2 mg, 2 mmol%), glycosyl donor **S16** (0.1 mmol), alkene (0.15 mmol, 1.5 equiv.), DIPEA (19.2 μL, 0.2 mmol, 2.0 equiv.), and MeCN/H_2O = 9:1 (1 mL). Purification by flash chromatography (DCM:MeOH = 10:1) provided the title compound (23.3 mg, 87% yield) as a colorless oil.

^{1}H NMR (400 MHz, CD_3OD) δ 8.41 (d, *J* = 5.0 Hz, 1H), 7.78 – 7.66 (m, 1H), 7.35 (d, *J* = 7.9 Hz, 1H), 7.25 – 7.17 (m, 1H), 3.86 (dd, *J* = 10.5, 4.8 Hz, 1H), 3.77 (dd, *J* = 11.8, 2.5 Hz, 1H), 3.63 (dd, *J* = 11.9, 6.0 Hz, 1H), 3.59 – 3.51 (m, 2H), 3.51 – 3.44 (m, 1H), 3.25 – 3.20 (m, 1H), 2.94 – 2.87 (m, 1H), 2.78 – 2.73(m, 1H), 2.13 – 1.93 (m, 2H).

^{13}C NMR (100 MHz, CD_3OD) δ 162.99, 149.43, 138.75, 124.91, 122.77, 76.20, 75.21, 74.61, 72.97, 72.37, 63.17, 34.53, 26.36.

HRMS (ESI) m/z calcd. for $C_{13}H_{19}NO_5$ ($[M+H]^+$) 270.1341, found: 270.1336.

(2R,3S,4R,5S,6R)-2-(Hydroxymethyl)-6-(2-(pyridin-2-yl)ethyl)tetrahydro-2H-pyran-3,4,5-triol (40)

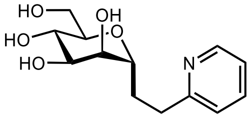

Prepared following the **general procedure F** outlined above using Ir[dF(CF_3)ppy]$_2$(dtbbpy)PF_6 (2.2 mg, 2 mmol%), glycosyl donor **S17** (0.1 mmol), alkene (0.15 mmol, 1.5 equiv.), DIPEA (19.2 μL, 0.2 mmol, 2.0 equiv.), and MeCN/H_2O = 9:1 (1 mL). Purification by flash chromatography (DCM:MeOH = 10:1) provided the title compound (25.3 mg, 94% yield) as a colorless oil.

^{1}H NMR (400 MHz, CD_3OD) δ 8.33 (d, *J* = 5.0 Hz, 1H), 7.65 (dd, *J* = 7.8, 1.9 Hz, 1H), 7.27 (d, *J* = 9.0 Hz, 1H), 7.14 (dd, *J* = 8.2, 5.7 Hz, 1H), 3.86 – 3.65 (m, 5H), 3.59 (d, *J* =

3.1 Hz, 1H), 3.57 (d, J = 3.4 Hz, 1H), 3.21 (p, J = 1.7 Hz, 2H), 2.86 – 2.81 (m, 1H), 2.73 – 2.68 (m, 1H).

^{13}C NMR (100 MHz, CD_3OD) δ 161.75, 148.05, 137.40, 123.57, 121.48, 72.97, 70.63, 69.08, 68.74, 60.75, 33.36, 25.73.

HRMS (ESI) m/z calcd. for $C_{13}H_{19}NO_5$ ($[M+H]^+$) 270.1341, found: 270.1337.

(2R,3R,4R,5R,6R)-2-(Hydroxymethyl)-6-(2-(pyridin-2-yl)ethyl)tetrahydro-2H-pyran-3,4,5-triol (41)

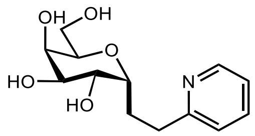

Prepared following the **general procedure F** outlined above using Ir[dF(CF_3)ppy]$_2$(dtbbpy)PF_6 (2.2 mg, 2 mmol%), glycosyl donor **S18** (0.1 mmol), alkene (0.15 mmol, 1.5 equiv.), DIPEA (19.2 μL, 0.2 mmol, 2.0 equiv.), and MeCN/H_2O = 9:1 (1 mL). Purification by flash chromatography (DCM:MeOH = 10:1) provided the title compound (22.6 mg, 84% yield) as a colorless oil.

^{1}H NMR (400 MHz, CD_3OD) δ 8.30 – 8.22 (m, 1H), 7.63 (dd, J = 1.8, 7.7 Hz, 1H), 7.25 (dd, J = 1.1, 7.9 Hz, 1H), 7.12 – 7.03 (m, 1H), 3.86 – 3.77 (m, 2H), 3.74 (dd, J = 5.2, 8.7 Hz, 1H), 3.70 (d, J = 3.5 Hz, 1H), 3.60 – 3.52 (m, 2H), 2.83 – 2.76 (m, 1H), 2.70 -2.65 (m, 1H), 2.08 – 1.89 (m, 1H), 1.88 – 1.78 (m, 1H).

^{13}C NMR (100 MHz, CD_3OD) δ 163.14, 149.44, 138.79, 124.95, 122.77, 74.35, 72.01, 70.50, 70.19, 62.18, 45.15, 34.77, 27.13.

HRMS (ESI) m/z calcd. for $C_{13}H_{19}NO_5$ ($[M+H]^+$) 270.1341, found: 270.1333.

(2S,3R,4R,5R,6S)-2-Methyl-6-(2-(pyridin-2-yl)ethyl)tetrahydro-2H-pyran-3,4,5-triol (42)

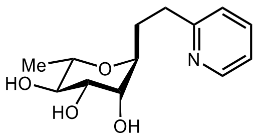

Prepared following the **general procedure F** outlined above using Ir[dF(CF_3)ppy]$_2$(dtbbpy)PF_6 (2.2 mg, 2 mmol%), glycosyl donor **S19** (0.1 mmol), alkene (0.15 mmol, 1.5 equiv.), DIPEA (19.2 µL, 0.2 mmol, 2.0 equiv.), and MeCN/H_2O = 9:1 (1 mL). Purification by flash chromatography (DCM:MeOH = 14:1) provided the title compound (22.7 mg, 90% yield) as a colorless oil.

^{1}H NMR (400 MHz, CD_3OD) δ 8.44 (d, *J* = 4.4 Hz, 1H), 7.78 (dd, *J* = 1.7, 7.7 Hz, 1H), 7.37 (d, *J* = 7.8 Hz, 1H), 7.31 – 7.21 (m, 1H), 3.80 (dd, *J* = 1.9, 11.1 Hz, 1H), 3.76 – 3.71 (m, 1H), 3.64 (dd, *J* = 3.2, 8.9 Hz, 1H), 3.49 -3.46 (m, 1H), 3.39 (t, *J* = 8.7 Hz, 1H), 2.96 – 2.75 (m, 2H), 2.23 – 2.09 (m, 1H), 1.91 – 1.77 (m, 1H), 1.23 (d, *J* = 6.1 Hz, 3H).

^{13}C NMR (100 MHz, CD_3OD) δ 162.40, 149.37, 139.02, 125.07, 122.97, 74.43, 73.17, 72.75, 70.97, 35.11, 29.87, 18.35.

HRMS (ESI) m/z calcd. for $C_{13}H_{19}NO_4$ ($[M+H]^+$) 254.1392, found: 254.1386.

(2R,3S,4R,5S)-2-(Hydroxymethyl)-5-(2-(pyridin-2-yl)ethyl)tetrahydrofuran-3,4-diol (43)

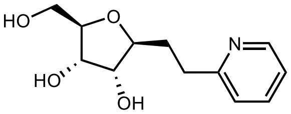

Prepared following the **general procedure F** outlined above using Ir[dF(CF_3)ppy]$_2$(dtbbpy)PF_6 (2.2 mg, 2 mmol%), glycosyl donor **S20** (0.1 mmol), alkene (0.15 mmol, 1.5 equiv.), DIPEA (19.2 µL, 0.2 mmol, 2.0 equiv.), and MeCN/H_2O = 9:1 (1 mL). Purification by flash chromatography (DCM:MeOH = 14:1) provided the title compound (12.7 mg, 53% yield) as a colorless oil.

^{1}H NMR (400 MHz, CD$_3$OD) δ 8.37 – 8.28 (m, 1H), 7.67 (dd, *J* = 1.8, 7.7 Hz, 1H), 7.27 (dd, *J* = 1.1, 7.9 Hz, 1H), 7.16 – 7.09 (m, 1H), 3.86 (t, *J* = 5.1 Hz, 1H), 3.75 – 3.68 (m, 1H), 3.68 – 3.63 (m, 2H), 3.60 (d, *J* = 3.5 Hz, 1H), 3.55 – 3.46 (m, 1H), 2.90 – 2.84 (m, 1H), 2.80 – 2.74 (m, 1H), 1.94 (s, 1H), 1.81 (s, 1H).

^{13}C NMR (100 MHz, CD$_3$OD) δ 162.93, 149.53, 138.78, 124.77, 122.79, 85.81, 83.46, 76.38, 72.97, 63.68, 35.15, 35.02.

HRMS (ESI) m/z calcd. for $C_{12}H_{17}NO_4$ ($[M+H]^+$) 240.1236, found: 240.1228.

1-((S)-3-(4-Amino-3-(4-phenoxyphenyl)-1H-pyrazolo[3,4-d]pyrimidin-1-yl)piperidin-1-yl)-3-((2R,3S,4R,6R)-3,4,5-trihydroxy-6-(hydroxymethyl)tetrahydro-2H-pyran-2-yl)propan-1-one (44)

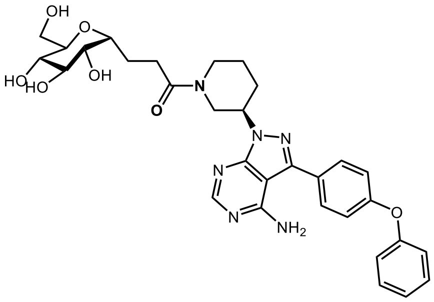

Prepared following the **general procedure F** outlined above using Ir[dF(CF$_3$)ppy]$_2$(dtbbpy)PF$_6$ (2.2 mg, 2 mmol%), glycosyl donor **S16** (0.1 mmol), alkene (0.15 mmol, 1.5 equiv.), DIPEA (19.2 μL, 0.2 mmol, 2.0 equiv.), and MeCN/H_2O = 9:1 (1 mL). Purification by flash chromatography (DCM:MeOH:Et$_3$N = 9:1:0.02) provided the title compound (33.2 mg, 55% yield) as a colorless oil.

^{1}H NMR (400 MHz, CD$_3$OD) δ 8.21 (d, *J* = 18.2 Hz, 1H), 7.62 (dd, *J* = 3.3, 8.7 Hz, 2H), 7.35 (t, *J* = 7.8 Hz, 2H), 7.17 – 7.08 (m, 2H), 7.08 – 7.01 (m, 3H), 4.72 (dd, *J* = 5.0, 10.4 Hz, 1H), 4.60 (d, *J* = 3.8 Hz, 1H), 4.58 (s, 1H), 4.33 (d, *J* = 13.1 Hz, 1H), 4.13 (dd, *J* = 4.0, 13.7 Hz, 1H), 3.99 (d, *J* = 13.7 Hz, 1H), 3.91 – 3.85 (m, 1H), 3.77 – 3.66 (m, 1H), 3.59 (d, *J* = 6.1 Hz, 1H), 3.52 (dd, *J* = 4.9, 9.5 Hz, 1H), 3.41 (dd, *J* = 3.2, 6.8 Hz, 1H), 3.36 – 3.26 (m, 2H), 3.19 - 3.15 (m, 2H), 2.91 (t, *J* = 11.5 Hz, 1H), 2.63 -2.58 (m, 1H),

2.45 – 2.40 (m, 2H), 2.35 – 2.20 (m, 1H), 2.13 (d, *J* = 12.6 Hz, 1H), 2.03 – 1.91 (m, 3H), 1.72 (t, *J* = 12.7 Hz, 1H), 1.65 (s, 1H), 1.24 -1.18 (m, 1H).

^{13}C NMR (100 MHz, CD_3OD) δ 174.06, 173.99, 159.86, 157.86, 157.84, 156.77, 156.63, 154.91, 145.83, 131.23, 125.06, 120.52, 119.93, 99.16, 76.45, 76.38, 74.81, 72.98, 72.20, 63.27, 54.36, 50.96, 47.02, 46.92, 43.08, 31.13, 30.17, 24.81, 21.87.

HRMS (ESI) m/z calcd. for $C_{31}H_{36}N_6O_7$ ($[M+H]^+$) 605.2723, found: 605.2717.

1-((S)-3-(4-Amino-3-(4-phenoxyphenyl)-1H-pyrazolo[3,4-d]pyrimidin-1-yl)piperidin-1-yl)-3-((2R,3R,4R,6R)-3,4,5-trihydroxy-6-(hydroxymethyl)tetrahydro-2H-pyran-2-yl)propan-1-one (45)

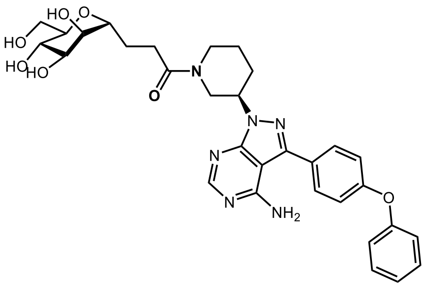

Prepared following the **general procedure F** outlined above using Ir[dF(CF_3)ppy]$_2$(dtbbpy)PF_6 (2.2 mg, 2 mmol%), glycosyl donor **S17** (0.1 mmol), alkene (0.15 mmol, 1.5 equiv.), DIPEA (19.2 μL, 0.2 mmol, 2.0 equiv.), and MeCN/H_2O = 9:1 (1 mL). Purification by flash chromatography (DCM:MeOH:Et_3N = 9:1:0.02) provided the title compound (25.4 mg, 42% yield) as a colorless oil.

^{1}H NMR (400 MHz, CD_3OD) δ 8.22 (d, *J* = 20.9 Hz, 1H), 7.63 (dd, *J* = 2.8, 8.7 Hz, 2H), 7.36 (d, *J* = 7.9 Hz, 2H), 7.15 (d, *J* = 7.4 Hz, 1H), 7.10 (d, *J* = 8.4 Hz, 2H), 7.07 – 7.03 (m, 2H), 4.78 – 4.66 (m, 1H), 4.59 (d, *J* = 12.0 Hz, 1H), 4.34 (d, *J* = 13.2 Hz, 1H), 4.13 (d, *J* = 13.7 Hz, 1H), 4.00 (d, *J* = 13.8 Hz, 1H), 3.89 – 3.78 (m, 1H), 3.80 – 3.75 (m, 1H), 3.75 – 3.69 (m, 2H), 3.70 – 3.64 (m, 2H), 3.58 (dd, *J* = 5.6, 8.3 Hz, 1H), 3.45 (ddd, *J* = 3.0, 6.3, 8.7 Hz, 1H), 3.37 (dd, *J* = 4.1, 8.3 Hz, 1H), 3.35 – 3.30 (m, 1H), 3.29 – 3.22 (m, 2H), 3.20 (t, *J* = 12.6 Hz, 1H), 2.92 (t, *J* = 12.2 Hz, 1H), 2.63- 2.57 (m, 1H), 2.49 – 2.40

(m, 1H), 2.36 – 2.20 (m, 1H), 2.15 (s, 1H), 2.06 – 1.90 (m, 2H), 1.88 – 1.67 (m, 2H), 1.63 – 1.58 (m, 1H).

^{13}C NMR (100 MHz, CD_3OD) δ 173.84, 159.89, 157.87, 156.80, 155.10, 154.96, 145.85, 131.23, 128.86, 125.08, 120.54, 99.19, 77.31, 76.42, 72.82, 69.59, 62.91, 54.38, 50.99, 49.85, 46.94, 43.13, 31.12, 30.82, 30.17, 25.90, 25.51, 24.82.

HRMS (ESI) m/z calcd. for $C_{31}H_{36}N_6O_7$ ($[M+H]^+$) 605.2723, found: 605.2715.

1-((S)-3-(4-Amino-3-(4-phenoxyphenyl)-1H-pyrazolo[3,4-d]pyrimidin-1-yl)piperidin-1-yl)-3-((2R,3S,4R,6R)-3,4,5-trihydroxy-6-(hydroxymethyl)tetrahydro-2H-pyran-2-yl)propan-1-one (46)

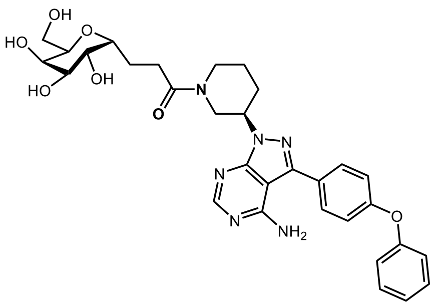

Prepared following the **general procedure F** outlined above using Ir[dF(CF_3)ppy]$_2$(dtbbpy)PF_6 (2.2 mg, 2 mmol%), glycosyl donor **S18** (0.1 mmol), alkene (0.15 mmol, 1.5 equiv.), DIPEA (19.2 μL, 0.2 mmol, 2.0 equiv.), and MeCN/H_2O = 9:1 (1 mL). Purification by flash chromatography (DCM:MeOH:Et_3N = 9:1:0.02) provided the title compound (32.6 mg, 54% yield) as a colorless oil.

^{1}H NMR (400 MHz, CD_3OD) δ 8.20 (d, *J* = 18.6 Hz, 1H), 7.61 (dd, *J* = 3.6, 8.7 Hz, 2H), 7.35 (t, *J* = 7.7 Hz, 2H), 7.12 (t, *J* = 7.4 Hz, 1H), 7.07 (d, *J* = 8.7 Hz, 2H), 7.03 (d, *J* = 8.0 Hz, 2H), 4.71 (dd, *J* = 5.0, 10.4 Hz, 1H), 4.67 – 4.58 (m, 1H), 4.56 (d, *J* = 4.1 Hz, 1H), 4.30 (d, *J* = 13.1 Hz, 1H), 4.20 – 4.07 (m, 1H), 4.03 – 3.88 (m, 1H), 3.83 (dd, *J* = 4.5, 7.0, 7.5 Hz, 1H), 3.67 – 3.60 (m, 2H), 3.55 – 3.48 (m, 1H), 3.36 – 3.26 (m, 2H), 3.19 (t, *J* = 12.6 Hz, 1H), 2.93 (t, *J* = 12.0 Hz, 1H), 2.62 (dd, *J* = 7.4, 15.2 Hz, 1H), 2.53 – 2.41 (m, 1H), 2.36 – 2.20 (m, 1H), 2.18 – 2.08 (m, 1H), 2.02 – 1.83 (m, 3H), 1.66 – 1.62 (m, 1H).

^{13}C NMR (100 MHz, CD_3OD) δ 174.27, 159.87, 59.77, 157.85, 156.76, 155.03, 154.92, 145.81, 131.22, 128.85, 125.06, 120.54, 120.52, 119.92, 99.16, 75.06, 71.91, 70.52, 62.47, 62.09, 54.31, 53.81, 50.98, 47.02, 46.89, 43.11, 31.10, 30.87, 25.91, 22.62,

HRMS (ESI) m/z calcd. for $C_{31}H_{36}N_6O_7$ ($[M+H]^+$) 605.2723, found: 605.2720.

1-((S)-3-(4-Amino-3-(4-phenoxyphenyl)-1H-pyrazolo[3,4-d]pyrimidin-1-yl)piperidin-1-yl)-3-((2S,4S,6R)-3,4,5-trihydroxy-6-methyltetrahydro-2H-pyran-2-yl)propan-1-one (47)

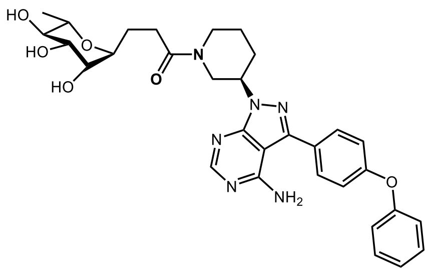

Prepared following the **general procedure F** outlined above using Ir[dF(CF_3)ppy]$_2$(dtbbpy)PF_6 (2.2 mg, 2 mmol%), glycosyl donor **S19** (0.1 mmol), alkene (0.15 mmol, 1.5 equiv.), DIPEA (19.2 μL, 0.2 mmol, 2.0 equiv.), and MeCN/H_2O = 9:1 (1 mL). Purification by flash chromatography (DCM:MeOH:Et_3N = 9:1:0.02) provided the title compound (40.6 mg, 69% yield) as a colorless oil.

^{1}H NMR (400 MHz, CD_3OD) δ 8.21 (d, *J* = 24.7 Hz, 1H), 7.62 (dd, *J* = 2.8, 8.7 Hz, 2H), 7.35 (t, *J* = 7.8 Hz, 2H), 7.11 (dd, *J* = 7.9, 21.3 Hz, 3H), 7.06 – 7.00 (m, 2H), 4.80 (dd, *J* = 4.3, 9.4 Hz, 1H), 4.77 – 4.68 (m, 1H), 4.59 (d, *J* = 11.8 Hz, 1H), 4.29 (d, *J* = 13.1 Hz, 1H), 4.16 – 4.04 (m, 1H), 3.96 (d, *J* = 13.7 Hz, 1H), 3.83 (dd, *J* = 3.3, 10.9 Hz, 1H), 3.78 – 3.68 (m, 2H), 3.67 – 3.57 (m, 1H), 3.48 – 3.39 (m, 1H), 3.38 (d, *J* = 2.6 Hz, 1H), 3.29 - 3.26 (m, 1H), 3.25 – 3.14 (m, 1H), 3.02 – 2.92 (m, 1H), 2.50 – 2.45 (m, 2H), 2.39 – 2.23 (m, 2H), 2.15 – 2.09 (m, 1H), 2.09 – 1.92 (m, 2H), 1.84 – 1.56 (m, 2H), 1.24 (d, *J* = 5.9 Hz, 2H), 1.15 (d, *J* = 6.0 Hz, 2H).

^{13}C NMR (100 MHz, CD_3OD) δ 173.61, 159.86, 159.79, 156.84, 155.11, 145.80, 131.21, 131.07, 128.87, 128.83, 125.07, 120.53, 119.92, 99.17, 78.10, 74.36, 73.14, 72.70, 71.04, 54.34, 51.09, 49.85, 46.96, 43.16, 31.07, 30.35, 25.87, 25.50, 24.74, 18.43,

HRMS (ESI) m/z calcd. for $C_{31}H_{36}N_6O_6$ ($[M+H]^+$) 589.2774, found: 589.2769.

β-Cyclodextrin derivant (48)

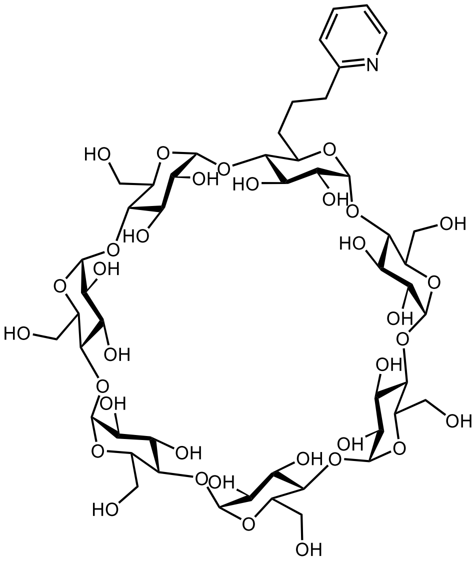

Prepared following the **general procedure F** outlined above using Ir[dF(CF_3)ppy]$_2$(dtbbpy)PF_6 (0.2 mg, 2 mmol%), glycosyl donor **S21** (0.01 mmol), alkene (0.02 mmol, 2.0 equiv.), DIPEA (2.0 μL, 0.02 mmol, 2.0 equiv.), and DMF (0.2 mL). Purification by flash chromatography (DCM:MeOH = 5:1) provided the title compound (10.2 mg, 59% yield) as a colorless oil.

^{1}H NMR (400 MHz, D_2O) δ 8.37 (t, J = 8.8 Hz, 1H), 7.87 (d, J = 27.4 Hz, 1H), 7.36 (s, 2H), 4.95 – 4.92 (m, 5H), 4.90 – 4.85 (m, 1H), 3.88 – 3.81 (m, 5H), 3.77 – 3.59 (m, 11H), 3.59 – 3.47 (m, 8H), 3.45 – 3.40 (m, 1H), 3.22 – 3.10 (m, 1H), 2.78 (s, 1H), 1.94 (s, 1H), 1.89 (s, 1H), 1.76 – 1.65 (m, 2H), 1.41 (d, J = 9.7 Hz, 1H).

^{13}C NMR (100 MHz, DMSO-d_6) δ 125.69, 123.91, 103.54, 103.51, 103.44, 103.40, 103.32, 102.83, 87.21, 82.73, 82.69, 82.57, 82.55, 82.47, 82.40, 74.85, 74.69, 74.61, 74.47, 73.63, 73.53, 73.41, 73.33, 72.28, 62.96, 61.56, 61.42, 37.44, 31.34, 26.15, 22.15, 15.01.

HRMS (ESI) m/z calcd. for $C_{49}H_{77}NO_{34}$ ($[M+H]^+$) 1224.4405, found: 1224.4409.

(2S,3R,4S,5S,6R)-2-Methoxy-6-(3-(pyridin-2-yl)propyl)tetrahydro-2H-pyran-3,4,5-triol (49)

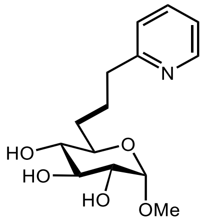

Prepared following the **general procedure F** outlined above using Ir[dF(CF_3)ppy]$_2$(dtbbpy)PF_6 (2.2 mg, 2 mmol%), glycosyl donor **S15** (0.1 mmol), alkene (0.15 mmol, 1.5 equiv.), DIPEA (19.2 μL, 0.2 mmol, 2.0 equiv.), and MeCN/H_2O = 9:1 (1 mL). Purification by flash chromatography (DCM:MeOH = 10:1) provided the title compound (20.9 mg, 74% yield) as a colorless oil.

^{1}H NMR (400 MHz, CD_3OD) δ 8.47 – 8.40 (m, 1H), 7.78 (dd, *J* = 1.8, 7.7 Hz, 1H), 7.34 (dd, *J* = 1.1, 7.9 Hz, 1H), 7.26 – 7.19 (m, 1H), 4.63 (d, *J* = 3.8 Hz, 1H), 3.57 (t, *J* = 9.3 Hz, 1H), 3.50 (dd, *J* = 2.3, 9.4 Hz, 1H), 3.05 (t, *J* = 9.3 Hz, 1H), 2.84 – 2.79 (m, 2H), 2.01 – 1.94 (m, 1H), 1.95 – 1.86 (m, 1H), 1.77 -1.72 (m, 1H), 1.47 – 1.43 (m, 1H), 1.42 – 1.33 (m, 1H), 1.31 (s, 1H).

^{13}C NMR (100 MHz, CD_3OD) δ 163.12, 149.45, 138.75, 124.65, 122.76, 101.15, 75.63, 75.10, 73.72, 72.13, 55.49, 38.72, 32.22, 27.23.

HRMS (ESI) m/z calcd. for $C_{14}H_{21}NO_5$ ($[M+H]^+$) 284.1498, found: 284.1488.

(2S,3S,4S,5S,6R)-2-Methoxy-6-(3-(pyridin-2-yl)propyl)tetrahydro-2H-pyran-3,4,5-triol (50)

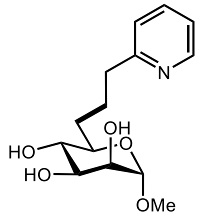

Prepared following the **general procedure F** outlined above using Ir[dF(CF_3)ppy]$_2$(dtbbpy)PF_6 (2.2 mg, 2 mmol%), glycosyl donor **S22** (0.1 mmol), alkene (0.15 mmol, 1.5 equiv.), DIPEA (19.2 μL, 0.2 mmol, 2.0 equiv.), and MeCN/H_2O = 9:1 (1 mL). Purification by flash chromatography (DCM:MeOH = 10:1) provided the title compound (19.2 mg, 68% yield) as a colorless oil.

^{1}H NMR (400 MHz, $CDCl_3$) δ 8.41 (dd, *J* = 1.8, 5.1 Hz, 1H), 7.75 (dd, *J* = 1.8, 7.7 Hz, 1H), 7.33 – 7.30 (m, 1H), 7.24 (dd, *J* = 5.0, 7.5 Hz, 1H), 4.57 (d, *J* = 1.8 Hz, 1H), 3.77 (dd, *J* = 1.7, 3.4 Hz, 1H), 3.61 (dd, *J* = 2.2, 3.6 Hz, 1H), 3.45 – 3.39 (m, 2H), 3.34 (s, 3H), 2.85 – 2.75 (m, 2H), 2.00 – 1.96 (m, 1H), 1.95 – 1.83 (m, 1H), 1.83 – 1.70 (m, 1H), 1.56 – 1.44 (m, 1H).

^{13}C NMR (100 MHz, $CDCl_3$) δ 159.86, 148.30, 137.78, 123.52, 121.97, 101.47, 72.82, 70.83, 70.50, 69.81, 53.90, 35.09, 32.91, 32.57.

HRMS (ESI) m/z calcd. for $C_{14}H_{21}NO_5$ ($[M+H]^+$) 284.1498, found: 284.1490.

1-((S)-3-(4-Amino-3-(4-phenoxyphenyl)-1H-pyrazolo[3,4-d]pyrimidin-1-yl)piperidin-1-yl)-4-((2R,4S,5S,6S)-3,4,5-trihydroxy-6-methoxytetrahydro-2H-pyran-2-yl)butan-1-one (51)

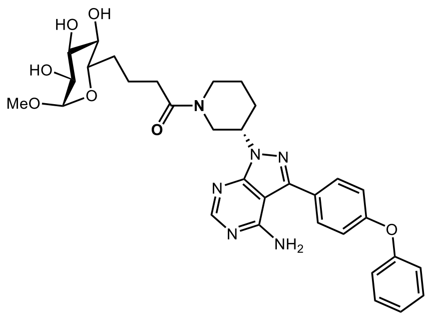

Prepared following the **general procedure F** outlined above using Ir[dF(CF_3)ppy]$_2$(dtbbpy)PF_6 (2.2 mg, 2 mmol%), glycosyl donor **S22** (0.1 mmol), alkene (0.15 mmol, 1.5 equiv.), DIPEA (19.2 μL, 0.2 mmol, 2.0 equiv.), and MeCN/H_2O = 9:1 (1 mL). Purification by flash chromatography (DCM:MeOH:Et_3N = 9:1:0.02) provided the title compound (32.8 mg, 53% yield) as a colorless oil.

^{1}H NMR (400 MHz, CDCl$_3$) δ 8.25 (d, *J* = 18.3 Hz, 1H), 7.67 (dd, *J* = 1.8, 8.6 Hz, 2H), 7.40 (dd, *J* = 7.3, 8.6 Hz, 2H), 7.17 (s, 1H), 7.14 (d, *J* = 8.7 Hz, 2H), 7.11 – 7.07 (m, 2H), 4.66 – 4.53 (m, 2H), 4.29 (d, *J* = 13.3 Hz, 1H), 4.13 (dd, *J* = 9.6, 16.5 Hz, 1H), 3.97 (d, *J* = 13.7 Hz, 1H), 3.77 (dd, *J* = 9.7, 13.3 Hz, 1H), 3.63 – 3.50 (m, 1H), 3.48 (d, *J* = 9.0 Hz, 1H), 3.43 – 3.36 (m, 3H), 3.34 (s, 1H), 3.30 – 3.23 (m, 6H), 3.05 (dd, *J* = 8.9, 12.3 Hz, 1H), 2.47 – 2.41 (m, 1H), 2.39 – 2.27 (m, 1H), 2.19 (dd, *J* = 5.8, 9.6 Hz, 1H), 1.95 (s, 1H), 1.85 (dd, *J* = 10.0, 21.0 Hz, 1H), 1.63 (t, *J* = 13.5 Hz, 2H), 1.54 – 1.38 (m, 1H).

^{13}C NMR (100 MHz, CDCl$_3$) δ 174.21, 159.94, 157.90, 156.64, 155.15, 131.24, 128.87, 128.83, 120.54, 101.15, 75.54, 75.05, 73.67, 72.18, 55.66, 55.54, 54.43, 53.80, 51.03, 47.05, 46.84, 43.09, 34.26, 32.17, 31.03, 25.86, 22.68, 19.31, 17.80.

HRMS (ESI) m/z calcd. for $C_{32}H_{38}N_6O_7$ ([M+H]$^+$) 619.2880, found: 619.2876.

(2S,3R,4S,5S,6R)-2-(4-Chloro-3-(4-(((S)-tetrahydrofuran-3-yl)oxy)benzyl)phenyl)-6-(3-(pyridin-2-yl)propyl)tetrahydro-2H-pyran-3,4,5-triol (52)

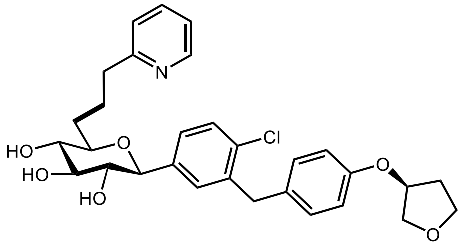

Prepared following the **general procedure F** outlined above using Ir[dF(CF_3)ppy]$_2$(dtbbpy)PF_6 (2.2 mg, 2 mmol%), glycosyl donor **S24** (0.1 mmol), alkene (0.15 mmol, 1.5 equiv.), DIPEA (19.2 μL, 0.2 mmol, 2.0 equiv.), and MeCN/H_2O = 9:1 (1 mL). Purification by flash chromatography (DCM:MeOH = 12:1) provided the title compound (33.4 mg, 62% yield) as a colorless oil.

^{1}H NMR (400 MHz, CD_3OD) δ 8.45 – 8.31 (m, 1H), 7.70 – 7.62 (m, 1H), 7.34 (d, *J* = 8.2 Hz, 1H), 7.28 – 7.19 (m, 3H), 7.11 (d, *J* = 8.5 Hz, 2H), 6.80 – 6.73 (m, 2H), 4.07 – 4.00 (m, 3H), 3.92 (dd, *J* = 3.1, 6.3 Hz, 2H), 3.88 (dd, *J* = 4.1, 8.4 Hz, 1H), 3.42 (t, *J* = 8.8 Hz, 1H), 2.79 (t, *J* = 7.3 Hz, 2H), 2.28 – 2.16 (m, 1H), 2.17 – 2.06 (m, 1H), 1.94 (dd, *J* = 3.9, 8.7 Hz, 2H), 1.82 – 1.72 (m, 1H), 1.54 – 1.48 (m, 1H), 1.35 – 1.24 (m, 1H).

^{13}C NMR (100 MHz, CD_3OD) δ 161.84, 155.85, 147.98, 138.92, 138.41, 137.30, 132.01, 130.23, 129.62, 128.67, 126.63, 123.29, 121.30, 115.05, 81.22, 79.57, 78.30, 77.14, 75.26, 74.03, 72.62, 66.72, 37.79, 37.27, 32.47, 30.99, 25.51.

HRMS (ESI) m/z calcd. for $C_{31}H_{34}ClNO_6$ ([M+H]$^+$) 540.2153, found: 540.2149.

(2R,3R,4S,5R)-2-Methoxy-5-(3-(pyridin-2-yl)propyl)tetrahydrofuran-3,4-diol (53)

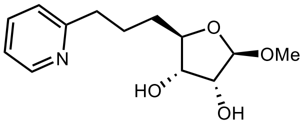

Prepared following the **general procedure F** outlined above using Ir[dF(CF_3)ppy]$_2$(dtbbpy)PF_6 (2.2 mg, 2 mmol%), glycosyl donor **S25** (0.1 mmol), alkene (0.15 mmol, 1.5 equiv.), DIPEA (19.2 μL, 0.2 mmol, 2.0 equiv.), and MeCN/H_2O = 9:1 (1

mL). Purification by flash chromatography (DCM:MeOH = 12:1) provided the title compound (15.7 mg, 62% yield) as a colorless oil.

^{1}H NMR (400 MHz, CD$_3$OD) δ 8.44 – 8.38 (m, 1H), 7.78 (dd, *J* = 1.8, 7.7 Hz, 1H), 7.35 (dd, *J* = 1.1, 7.9 Hz, 1H), 7.26 – 7.17 (m, 1H), 3.91 (dd, *J* = 2.6, 4.9 Hz, 2H), 3.89 – 3.83 (m, 1H), 3.37 – 3.31 (m, 1H), 2.86 – 2.81 (m, 2H), 1.93 – 1.89 (m, 1H), 1.89 – 1.79 (m, 1H), 1.72 – 1.66(m, 1H), 1.64 – 1.55 (m, 1H).

^{13}C NMR (100 MHz, CD$_3$OD) δ 163.11, 149.47, 138.74, 124.67, 122.78, 109.60, 83.45, 76.41, 76.33, 55.15, 38.56, 35.69, 27.67.

HRMS (ESI) m/z calcd. for $C_{13}H_{19}NO_4$ ($[M+H]^+$) 254.1392, found: 254.1387.

1-((2R,3R,4S,5R)-3,4-Dihydroxy-5-(3-(pyridin-2-yl)propyl)tetrahydrofuran-2-yl)pyrimidine-2,4(1H,3H)-dione (54)

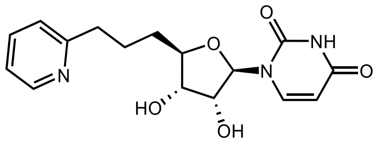

Prepared following the **general procedure F** outlined above using Ir[dF(CF$_3$)ppy]$_2$(dtbbpy)PF$_6$ (2.2 mg, 2 mmol%), glycosyl donor **S26** (0.1 mmol), alkene (0.15 mmol, 1.5 equiv.), DIPEA (19.2 μL, 0.2 mmol, 2.0 equiv.), and MeCN/H_2O = 9:1 (1 mL). Purification by flash chromatography (DCM:MeOH:Et$_3$N = 9:1:0.02) provided the title compound (18.0 mg, 54% yield) as a colorless oil.

^{1}H NMR (400 MHz, CD$_3$OD) δ 8.51 – 8.32 (m, 1H), 7.77 (dd, *J* = 1.8, 7.7 Hz, 1H), 7.56 (d, *J* = 8.1 Hz, 1H), 7.39 – 7.30 (m, 1H), 7.26 – 7.19 (m, 1H), 5.80 (d, *J* = 4.2 Hz, 1H), 5.72 (d, *J* = 8.1 Hz, 1H), 5.50 (s, 1H), 4.16 (dd, *J* = 4.2, 5.5 Hz, 1H), 3.95 – 3.91 (m, 1H), 3.88 – 3.85 (m, 1H), 2.87 – 2.81 (m, 2H), 1.97 – 1.89 (m, 1H), 1.88 – 1.81 (m, 1H), 1.77 – 1.68 (m, 1H), 1.76 – 1.64 (m, 1H).

^{13}C NMR (100 MHz, CD_3OD) δ 166.11, 162.87, 152.23, 149.60, 149.36, 142.54, 138.81, 125.09, 123.26, 102.94, 91.59, 84.60, 75.05, 74.73, 38.35, 33.90.

HRMS (ESI) m/z calcd. for $C_{16}H_{19}N_3O_5$ ($[M+H]^+$) 334.1403, found: 334.1399.

Additional examples:

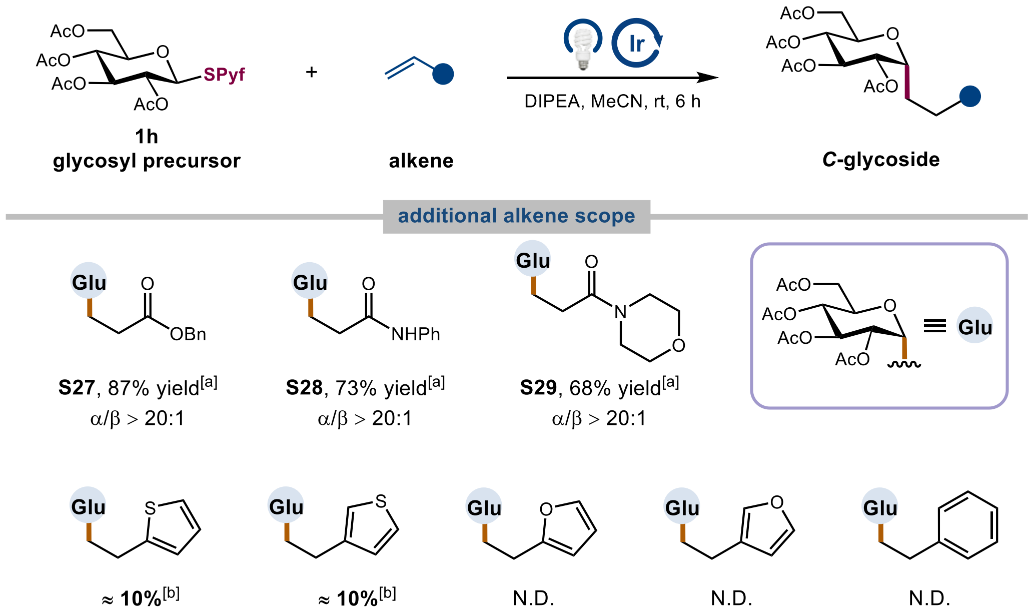

Table S5. Additional alkene scope. [a] Isolated yields. [b] Yields Determined by ^{1}H NMR. The anomeric ratios of isolated and purified products were determined by ^{1}H NMR spectroscopic analysis. N.D. = not detected.

(2R,3R,4R,5S,6R)-2-(Acetoxymethyl)-6-(3-(benzyloxy)-3-oxopropyl)tetrahydro-2H-pyran-3,4,5-triyl triacetate (S27)

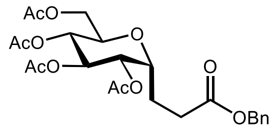

Prepared following the **general procedure E** outlined above using Ir[dF(CF_3)ppy]$_2$(dtbbpy)PF_6 (2.2 mg, 2 mmol%), glycosyl donor **1h** (0.1 mmol), alkene (0.15 mmol, 1.5 equiv.), DIPEA (19.2 µL, 0.2 mmol, 2.0 equiv.), and MeCN (1 mL).

Purification by flash chromatography (PE:EA = 3:1) provided the title compound (43.0 mg, 87% yield) as a colorless oil.

^{1}H NMR (400 MHz, $CDCl_3$) δ 7.37 – 7.31 (m, 5H), 5.30 (t, J = 9.1 Hz, 1H), 5.08 (dd, J = 9.6, 5.8 Hz, 1H), 4.98 (t, J = 9.1 Hz, 1H), 4.26 – 4.21 (m, 1H), 4.19 – 4.15 (m, 1H), 4.00 (dd, J = 12.2, 2.6 Hz, 1H), 3.84 (ddd, J = 9.4, 5.1, 2.6 Hz, 1H), 2.51 – 2.39 (m, 2H), 2.18 – 2.08 (m, 1H), 2.05 (s, 3H), 2.04 (s, 3H), 2.02 (s, 3H), 2.02 (s, 3H), 1.94 – 1.84 (m, 1H).

^{13}C NMR (100 MHz, $CDCl_3$) δ 172.73, 170.77, 170.21, 169.76, 169.66, 135.84, 128.74, 128.73, 128.49, 128.45, 128.44, 72.20, 70.36, 70.20, 68.99, 68.73, 66.69, 66.67, 62.21, 29.93, 20.93, 20.82, 20.77, 20.70.

(2R,3R,4R,5S,6R)-2-(Acetoxymethyl)-6-(3-oxo-3-(phenylamino)propyl)tetrahydro-2H-pyran-3,4,5-triyl triacetate (S28)

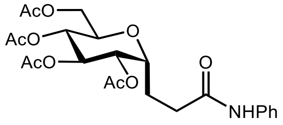

Prepared following the **general procedure E** outlined above using Ir[dF(CF_3)ppy]$_2$(dtbbpy)PF_6 (2.2 mg, 2 mmol%), glycosyl donor **1h** (0.1 mmol), alkene (0.15 mmol, 1.5 equiv.), DIPEA (19.2 μL, 0.2 mmol, 2.0 equiv.), and MeCN (1 mL). Purification by flash chromatography (PE:EA = 3:1) provided the title compound (35.0 mg, 73% yield) as a colorless oil.

^{1}H NMR (400 MHz, $CDCl_3$) δ 7.59 (s, 1H), 7.53 – 7.50 (m, 1H), 7.50 – 7.47 (m, 2H), 7.35 – 7.27 (m, 2H), 5.38 – 5.29 (m, 1H), 5.08 (dd, J = 9.4, 5.7 Hz, 1H), 4.99 (t, J = 9.1 Hz, 1H), 4.28 – 4.23 (m, 2H), 4.23 – 4.17 (m, 1H), 4.04 (dd, J = 12.3, 2.9 Hz, 1H), 2.46 – 2.36 (m, 2H), 2.29 – 2.16 (m, 1H), 2.06 (s, 3H), 2.05 (s, 3H), 2.02 (s, 3H), 2.01 (s, 3H), 1.98 – 1.92 (m, 1H).

^{13}C NMR (100 MHz, $CDCl_3$) δ 170.84, 170.28, 170.07, 169.79, 169.66, 137.98, 129.11, 128.88, 124.43, 119.84, 119.77, 72.28, 70.39, 70.30, 69.15, 68.87, 62.41, 33.13, 21.42, 20.82, 20.77, 20.74, 20.70.

(2R,3R,4R,5S,6R)-2-(acetoxymethyl)-6-(3-morpholino-3-oxopropyl)tetrahydro-2H-pyran-3,4,5-triyl triacetate (S29)

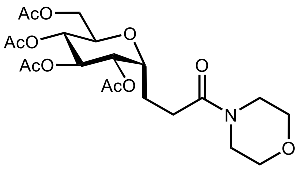

Prepared following the **general procedure E** outlined above using Ir[dF(CF_3)ppy]$_2$(dtbbpy)PF_6 (2.2 mg, 2 mmol%), glycosyl donor **1h** (0.1 mmol), alkene (0.15 mmol, 1.5 equiv.), DIPEA (19.2 μL, 0.2 mmol, 2.0 equiv.), and MeCN (1 mL). Purification by flash chromatography (PE:EA = 3:1) provided the title compound (32.1 mg, 68% yield) as a colorless oil.

^{1}H NMR (400 MHz, $CDCl_3$) δ 5.33 (t, J = 9.2 Hz, 1H), 5.07 (dd, J = 9.7, 5.8 Hz, 1H), 4.98 (t, J = 9.2 Hz, 1H), 4.24 – 4.13 (m, 2H), 4.04 (dd, J = 12.2, 2.7 Hz, 1H), 3.84 (ddd, J = 9.5, 4.9, 2.7 Hz, 1H), 3.69 – 3.57 (m, 6H), 3.48 – 3.42 (m, 2H), 2.48 – 2.25 (m, 2H), 2.22 – 2.10 (m, 2H), 2.06 (s, 3H), 2.04 (s, 3H), 2.01 (s, 3H), 2.00 (s, 3H).
^{13}C NMR (100 MHz, $CDCl_3$) δ 170.56, 170.47, 170.07, 169.80, 169.61, 72.47, 70.42, 70.36, 68.97, 68.89, 66.94, 66.62, 62.52, 45.92, 42.17, 29.01, 28.26, 20.82, 20.79, 20.73, 20.69.

6) Further Application

Synthetic application a:

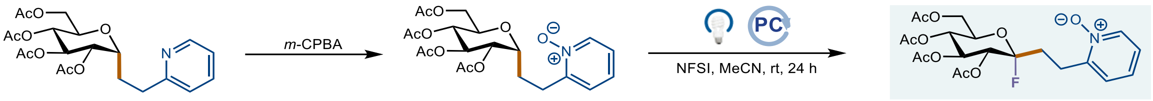

2 **55**, 99% yield **56**, 44% yield α/β > 20:1

To a solution of compound **2** (0.1 mmol, 1.0 equiv.) in DCM (0.1 M), 3-chloroperoxybenzoic acid (*m*-CPBA, 0.2 mmol, 2 equiv.) was added and the mixture was stirred for 2 h. After the complete consumption of compound **2** as monitored by TLC analysis, the reaction mixture was filtered and concentrated in vacuo. The resulting residue was purified by silica gel flash chromatography (DCM:MeOH = 10:1) to give the compound **55** (45.1 mg, 99% yield) as a colorless oil.

^{1}H NMR (400 MHz, $CDCl_3$) δ 8.26 – 8.23 (m, 1H), 7.28 (dd, *J* = 7.6, 2.3 Hz, 1H), 7.24 – 7.16 (m, 2H), 5.33 (t, *J* = 9.3 Hz, 1H), 5.10 (dd, *J* = 9.7, 5.9 Hz, 1H), 5.02 (t, *J* = 9.1 Hz, 1H), 4.28 (dd, *J* = 12.9, 5.5 Hz, 1H), 4.22 (ddd, *J* = 9.2, 5.7, 2.9 Hz, 1H), 4.10 (d, *J* = 2.6 Hz, 1H), 4.07 (t, *J* = 2.7 Hz, 1H), 3.21 – 3.17 (m, 1H), 2.79 – 2.69 (m, 1H), 2.44 – 2.30 (m, 1H), 2.10 (s, 3H), 2.03 (s, 3H), 2.02 (s, 3H), 2.00 (s, 3H).

^{13}C NMR (100 MHz, $CDCl_3$) δ 170.89, 170.26, 169.90, 169.79, 151.26, 139.92, 126.54, 126.18, 124.28, 72.37, 70.60, 70.52, 68.82, 68.75, 62.35, 27.28, 21.75, 20.93, 20.89, 20.82, 20.79.

HRMS (ESI) m/z calcd. for $C_{21}H_{27}NO_{10}$ ($[M+H]^+$) 454.1713, found: 454.1712.

To an 8 mL vial equipped with a stir bar was added compound **55** (0.1 mmol, 1.0 equiv.), $[Mes\text{-}Acr\text{-}Me^+]BF_4^-$ (3.9 mg, 10 mmol%), *N*-fluorobenzenesulfonimide (NFSI, 70.8 mg, 0.2 mmol, 2.0 equiv.), and MeCN (1.0 mL). The vial was sealed and the solution was degassed by sparging with nitrogen for 10 minutes before sealing with parafilm. Subsequently, the reaction was stirred and irradiated using 40 W 440 nm blue LED lamps (5 cm away, with cooling fan & air-condition at 25 °C to maintain the reaction at room temperature) for 24 h. The reaction was quenched by exposure to air and concentrated. The

residue was purified by flash chromatography on silica gel (DCM:MeOH = 10:1) to afford the desired product **56** (20.6 mg, 44% yield) as a colorless oil.

^{1}H NMR (400 MHz, $CDCl_3$) δ 8.33 (d, *J* = 6.5 Hz, 1H), 7.32 – 7.27 (m, 2H), 7.23 (d, *J* = 6.3 Hz, 1H), 5.43 (t, *J* = 9.7 Hz, 1H), 5.18 – 5.14 (m, 1H), 5.13 – 5.06 (m, 1H), 4.24 (dd, *J* = 12.4, 4.1 Hz, 1H), 4.14 (dd, *J* = 10.0, 3.9 Hz, 1H), 4.08 (dd, *J* = 12.4, 2.3 Hz, 1H), 3.10 (d, *J* = 7.4 Hz, 2H), 2.33 – 2.26 (m, 1H), 2.22 – 2.13 (m, 1H), 2.11 (s, 3H), 2.09 (s, 4H), 2.03 (s, 3H), 1.99 (s, 3H).

^{13}C NMR (100 MHz, $CDCl_3$) δ 170.73, 170.13, 169.95, 169.55, 140.00, 126.57, 124.25, 113.52, 111.23, 71.17, 70.99, 70.90, 70.38, 70.33, 67.66, 61.47, 46.00, 30.76 (d, *J* = 23.7 Hz), 20.87, 20.81, 20.71, 20.69.

^{19}F NMR (376 MHz, $CDCl_3$) δ -127.88 (td, *J* = 23.9, 7.1 Hz, 1F).

HRMS (ESI) m/z calcd. for $C_{21}H_{26}FNO_{10}$ ($[M+H]^+$) 472.1619, found: 472.1615.

Synthetic application b:

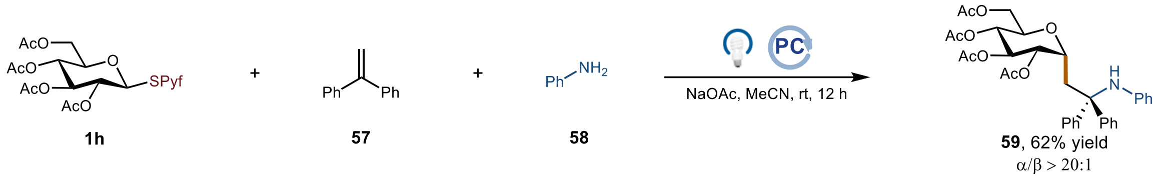

To an 8 mL vial equipped with a stir bar was added compound **1h** (0.2 mmol, 1.0 equiv.), DPAIPN (1.6 mg, 1 mmol%), 1,1-Diphenylethylene (42 μL, 0.24 mmol, 1.2 equiv.), aniline(42 μL, 0.24 mmol, 1.2 equiv.), sodium acetate (24.6 mg, 0.30 mmol, 1.5 equiv.), and MeCN (4.0 mL). The vial was sealed and the solution was degassed by sparging with nitrogen for 5 minutes before sealing with parafilm. Then the reaction was stirred and irradiated using 40 W 440 nm blue LED lamps (5 cm away, with cooling fan & air-condition at 25 °C to maintain the reaction at room temperature) for 12 h. The reaction was quenched by exposure to air and concentrated. The residue was purified by flash chromatography on silica gel (PE: EA = 4:1) to afford the desired product **59** (74.8 mg, 62% yield) as a colorless oil.

^{1}H NMR (400 MHz, CDCl$_3$) δ 7.44 (dd, *J* = 8.8, 2.0 Hz, 2H), 7.41 – 7.34 (m, 2H), 7.27 – 7.09 (m, 7H), 6.90 – 6.80 (m, 2H), 6.49 (t, *J* = 7.3 Hz, 1H), 6.25 (d, *J* = 8.0 Hz, 2H), 5.40 (d, *J* = 13.8 Hz, 1H), 5.10 (t, *J* = 8.8 Hz, 1H), 4.81 (t, *J* = 8.5 Hz, 1H), 4.73 (dd, *J* = 9.3, 5.4 Hz, 1H), 4.01 (dd, *J* = 11.6, 4.6 Hz, 1H), 3.90 (dd, *J* = 12.4, 4.6 Hz, 1H), 3.71 (ddd, *J* = 8.0, 4.6, 2.7 Hz, 1H), 3.46 (dd, *J* = 12.4, 2.5 Hz, 1H), 2.87 (dd, *J* = 15.3, 9.8 Hz, 1H), 2.58 (d, *J* = 15.2 Hz, 1H), 1.97 (s, 3H), 1.94 (s, 3H), 1.88 (s, 3H), 1.86 (s, 3H).

^{13}C NMR (100 MHz, CDCl$_3$) δ 170.70, 169.96, 169.45, 169.05, 145.73, 145.07, 143.40, 128.73, 128.64, 128.60, 128.54, 127.59, 127.15, 127.04, 126.98, 117.48, 115.53, 70.45, 70.23, 69.80, 69.58, 68.09, 65.07, 61.52, 35.30, 20.86, 20.76, 20.69, 20.66, 20.64.

HRMS (ESI) m/z calcd. for $C_{34}H_{37}NO_9$ ($[M+H]^+$) 604.2546, found: 604.2542.

Synthetic application c:

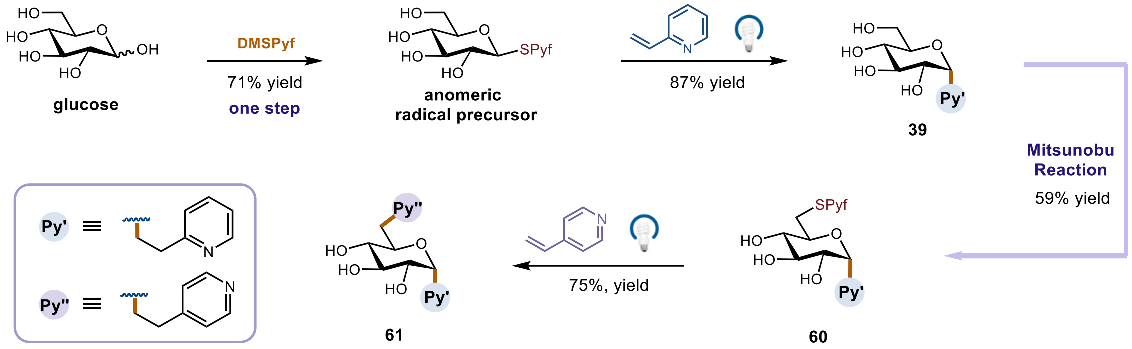

Prepared following the **general procedure B** and **general procedure F** outlined above starting on 1.0 mmol scale to give the compound **39** (157.2 mg, 59% yield for two steps) as a white foam.

To a solution of the above **39** (0.38 mmol, 1.0 equiv.), 2,3,5,6-tetraflupropyridine-4-thiol (104 mg, 0.57 mmol, 1.5 equiv.) and PPh_3 (199.0 mg, 0.76 mmol, 2.0 equiv.) in dry THF (40 mL) at 0 °C under a nitrogen atmosphere. DEAD (79 μL, 0.50 mmol, 1.3 equiv.) was added dropwise over a period of 5 mins. After consumption of the starting material was confirmed by TLC analysis (about 4 h), the solvent was evaporated under reduced pressure and the resulting oil purified by flash column chromatography to give product **60** (97.3 mg, 59% yield) as a colorless oil.

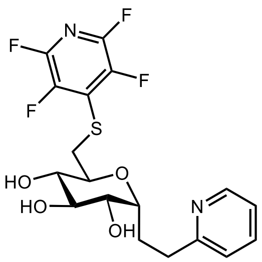

^{1}H NMR (400 MHz, CD_3OD) δ 8.32 – 8.29 (m, 1H), 7.66 – 7.61 (m, 1H), 7.17 – 7.13 (m, 2H), 3.66 – 3.63 (m, 1H), 3.60 – 3.56 (m, 1H), 3.52 (dd, J = 13.8, 2.7 Hz, 1H), 3.49 – 3.38 (m, 2H), 3.28 – 3.23 (m, 1H), 3.18 (d, J = 8.9 Hz, 1H), 2.81 (dd, J = 14.6, 7.6 Hz, 1H), 2.59 – 2.55 (m, 1H), 1.91 – 1.87 (m, 2H).

^{13}C NMR (100 MHz, CD_3OD) δ 161.33, 148.26, 143.06 – 142.87 (m), 142.77 – 142.46 (m), 142.31 – 141.77 (m), 140.17 – 140.01 (m), 137.24, 132.21 – 132.00 (m), 123.32, 121.40, 75.48, 73.40, 73.13, 72.51, 71.45, 35.31, 33.26, 24.64.

^{19}F NMR (376 MHz, CD_3OD) δ -94.70 – -95.19 (m, 2F), -138.79 – -139.30 (m, 2F).

HRMS (ESI) m/z calcd. for $C_{18}H_{18}F_4N_2O_4S$ ($[M+H]^+$) 435.1001, found: 435.0997.

To an 8 mL via equipped with a stir bar was added the above **60** (0.1 mmol, 1.0 equiv.), $Ir[dF(CF_3)ppy]_2(dtbbpy)PF_6$ (2.2 mg, 2 mmol%), 4-vinylpyridine (16.2 μL, 0.15 mmol, 1.5 equiv.), DIPEA (19.2 μL, 0.2 mmol, 2.0 equiv.), and MeCN/H_2O = 9:1 (1.0 mL). The vial was sealed and the solution was degassed by sparging with nitrogen for 10 minutes before sealing with parafilm. Then the reaction was stirred and irradiated using 40 W 427 nm blue LED lamps (5 cm away, with cooling fan & air-condition at 25 °C to maintain the reaction at room temperature) for 6 h. The reaction was quenched by exposure to air and concentrated. The residue was purified by flash chromatography on silica gel to afford the desired product **61**. (26.8 mg, 75% yield) as a colorless oil.

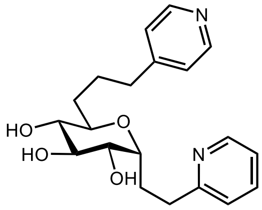

^{1}H NMR (400 MHz, CD_3OD) δ 8.43 (dd, J = 2.1, 4.7 Hz, 2H), 7.76 – 7.71 (m, 2H), 7.41 – 7.31 (m, 2H), 7.25 – 7.21 (m, 2H), 3.88 (dd, J = 5.3, 10.4 Hz, 1H), 3.59 (dd, J = 5.8, 9.5 Hz, 1H), 3.52 (d, J = 8.8 Hz, 1H), 3.45 (d, J = 2.4 Hz, 1H), 3.05 (t, J = 8.9 Hz, 1H), 2.85 – 2.80 (m, 2H), 2.79 (s, 2H), 2.14 – 2.00 (m, 4H), 1.97 – 1.87 (m, 2H).

^{13}C NMR (100 MHz, CD_3OD) δ 163.17, 162.88, 149.61, 149.47, 138.71, 124.75, 124.67, 122.79, 122.75, 76.60, 76.20, 75.21, 73.29, 73.20, 38.81, 34.99, 32.81, 27.36, 26.18.

HRMS (ESI) m/z calcd. for $C_{21H26}N_2O_4$ ($[M+H]^+$) 359.1971, found: 359.1967.

7) References

1. K. M. Koeller, C.-H. Wong, *Chem. Rev*. **2000**, *100*, 4465-4494.

2.H. Driguez, W. Szeja, *Synthesis* **1994**, *12*, 1413-1414.

3. M. O. Zubkov, M. D. Kosobokov, V. V. Levin, A. D. Dilman, *Org. Lett*. **2022**, *24*, 2354–2358.

8) Spectral Data

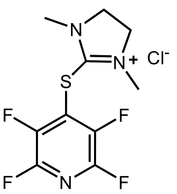

S2 - ^{1}H NMR (400 MHz, $CDCl_3$)

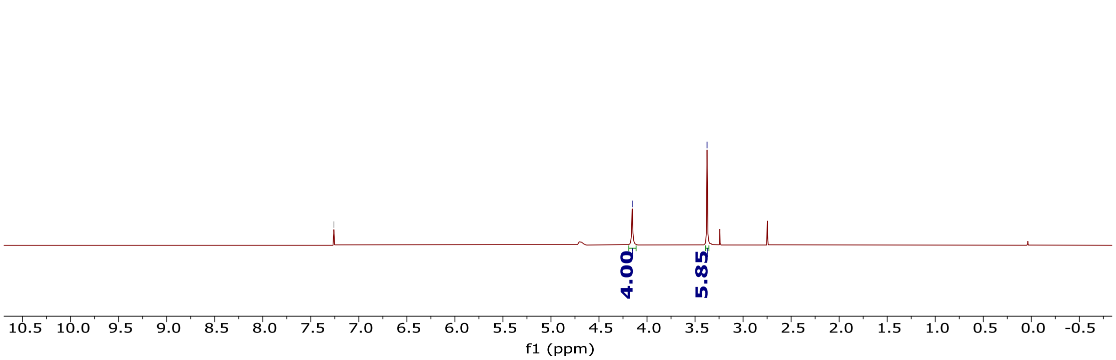

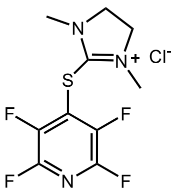

S2 - ^{13}C NMR (100 MHz, $CDCl_3$)

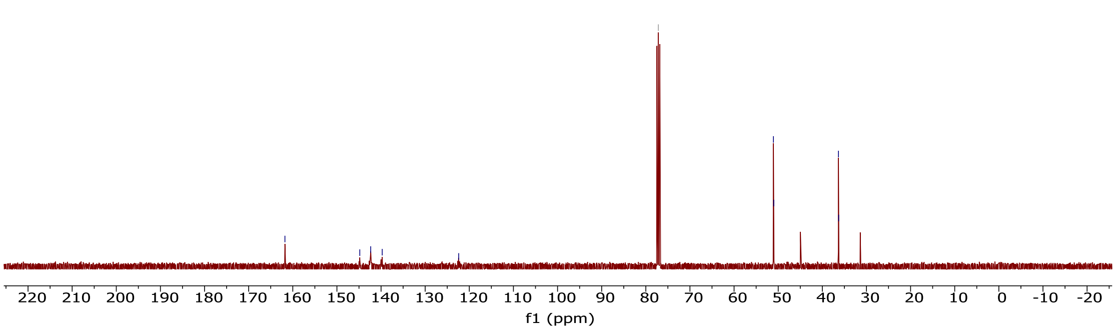

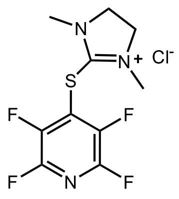

S2 - ^{19}F NMR (376 MHz, $CDCl_3$)

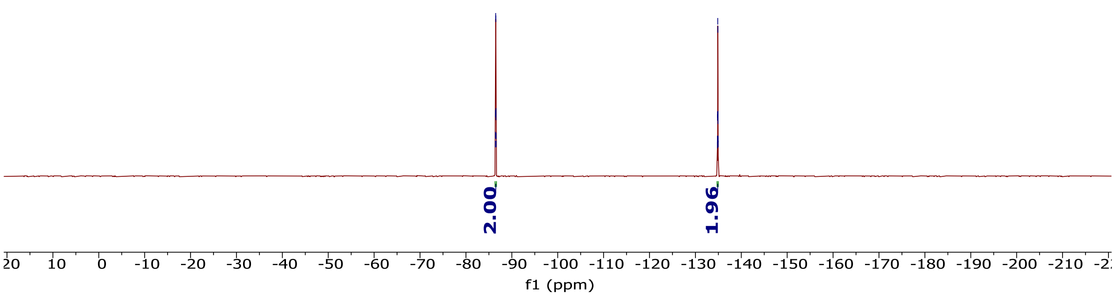

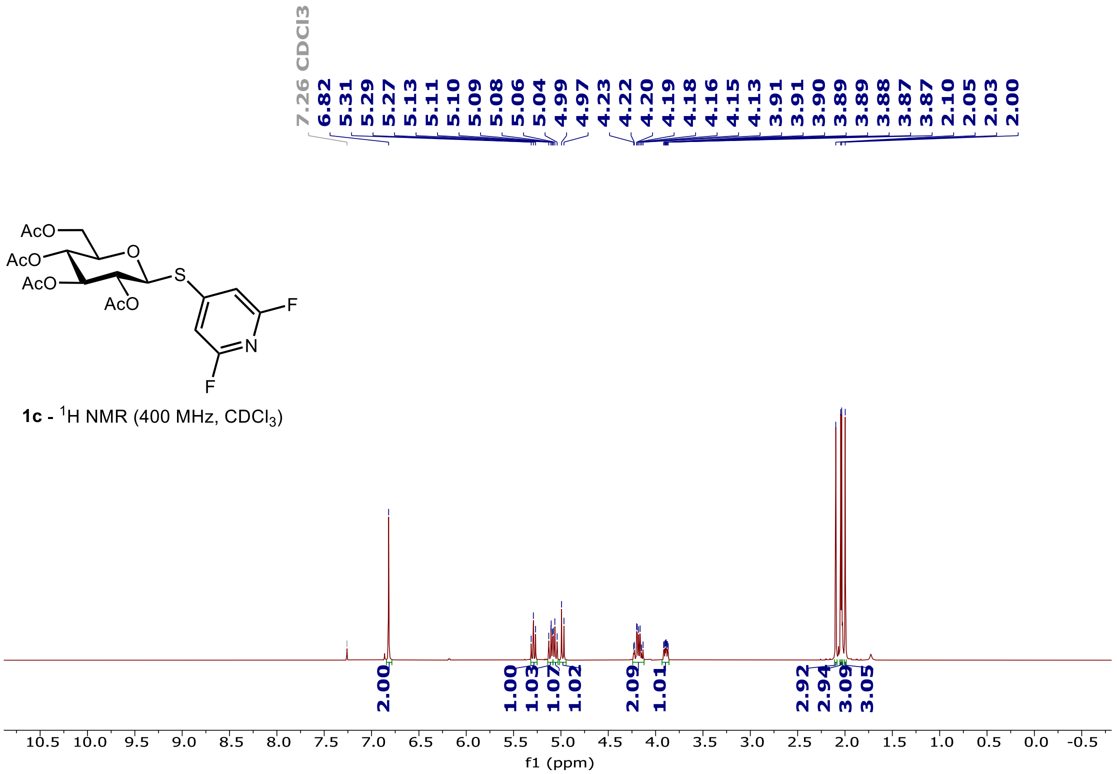
7.26 CDCl3
6.82
5.31
5.29
5.27
5.13
5.11
5.10
5.09
5.08
5.06
5.04
4.99
4.97
4.23
4.22
4.20
4.19
4.18
4.16
4.15
4.13
3.91
3.91
3.90
3.89
3.89
3.88
3.87
3.87
2.10
2.05
2.03
2.00
AcO
AcO
AcO
AcO
O
S
F
N
F
1c - ^{1}H NMR (400 MHz, $CDCl_3$)
2.00
1.00
1.03
1.07
1.02
2.09
1.01
2.92
2.94
3.09
3.05
10.5 10.0 9.5 9.0 8.5 8.0 7.5 7.0 6.5 6.0 5.5 5.0 4.5 4.0 3.5 3.0 2.5 2.0 1.5 1.0 0.5 0.0 -0.5
f1 (ppm)

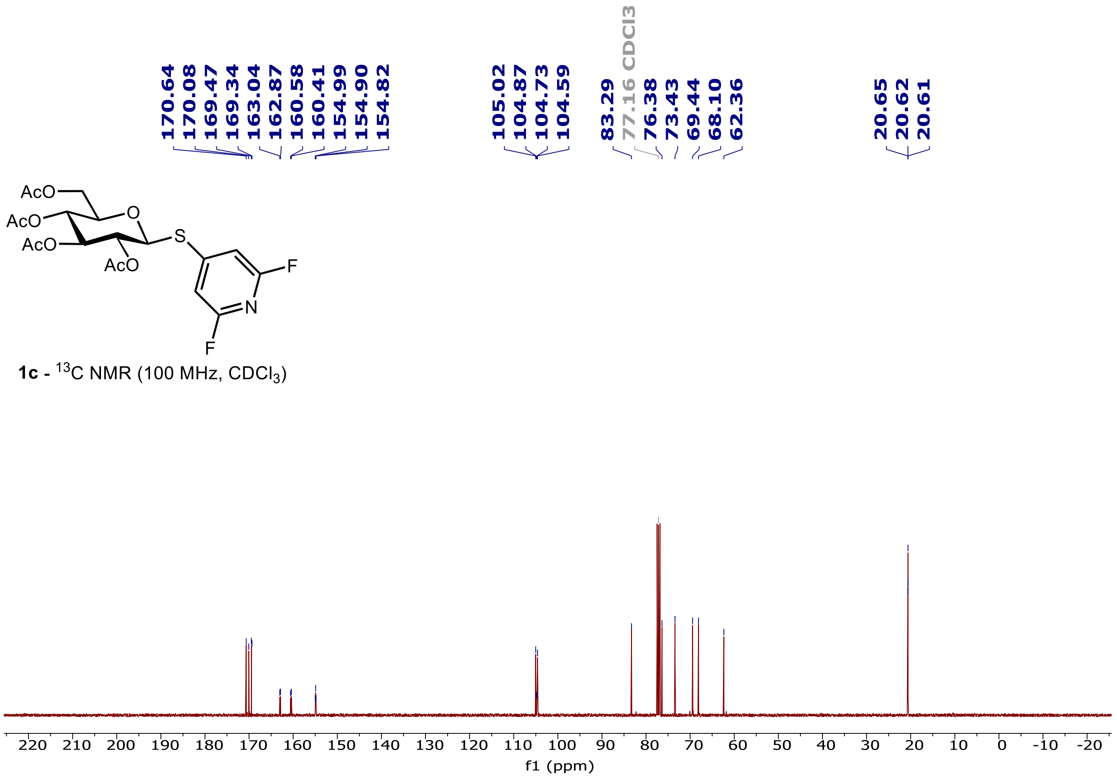
170.64
170.08
169.47
169.34
163.04
162.87
160.58
160.41
154.99
154.90
154.82
105.02
104.87
104.73
104.59
83.29
77.16 CDCl3
76.38
73.43
69.44
68.10
62.36
20.65
20.62
20.61
AcO
AcO
AcO
AcO
O
S
F
N
F
1c - ^{13}C NMR (100 MHz, $CDCl_3$)
220 210 200 190 180 170 160 150 140 130 120 110 100 90 80 70 60 50 40 30 20 10 0 -10 -20
f1 (ppm)

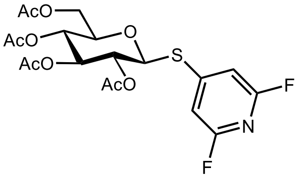

1c - ^{19}F NMR (376 MHz, $CDCl_3$)

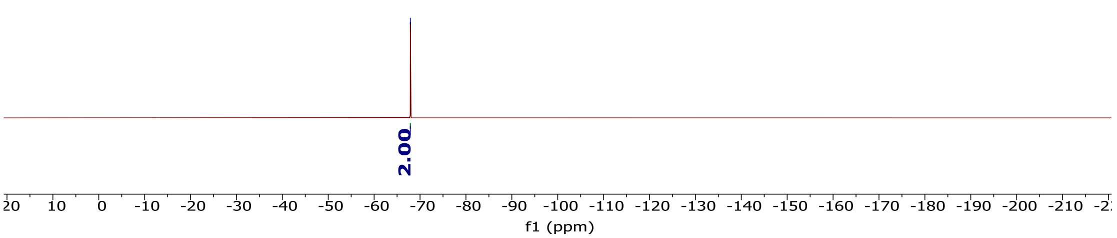

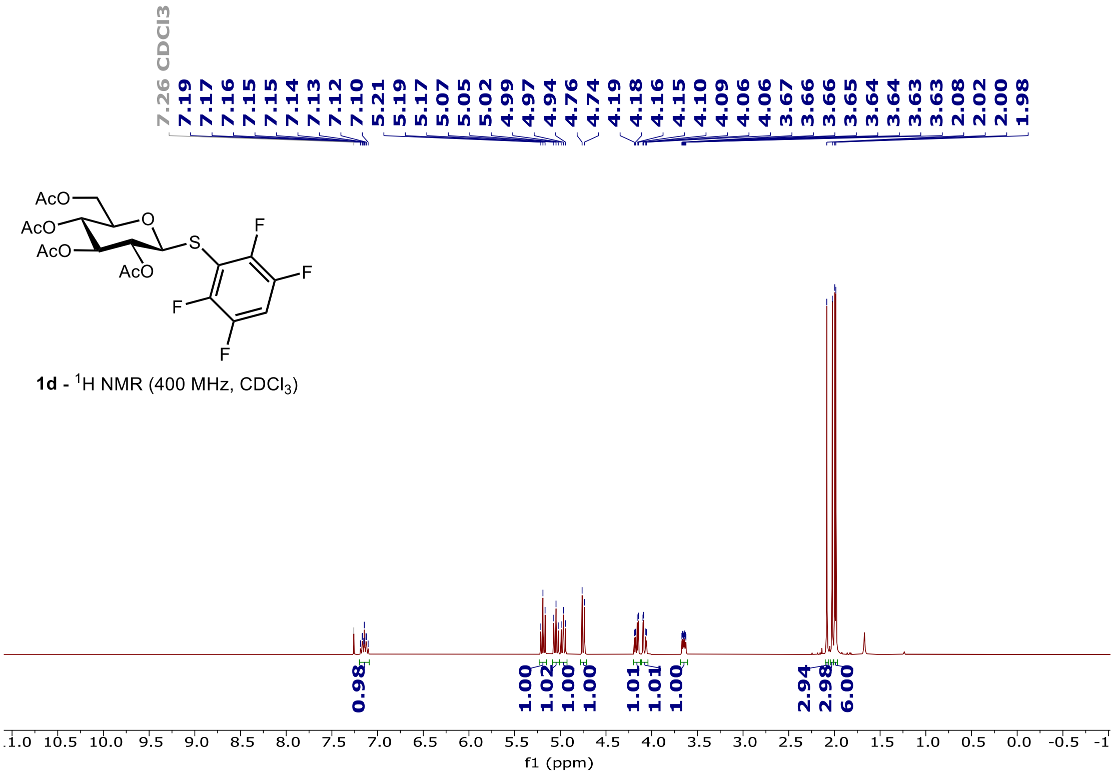

1d - ^{1}H NMR (400 MHz, $CDCl_3$)

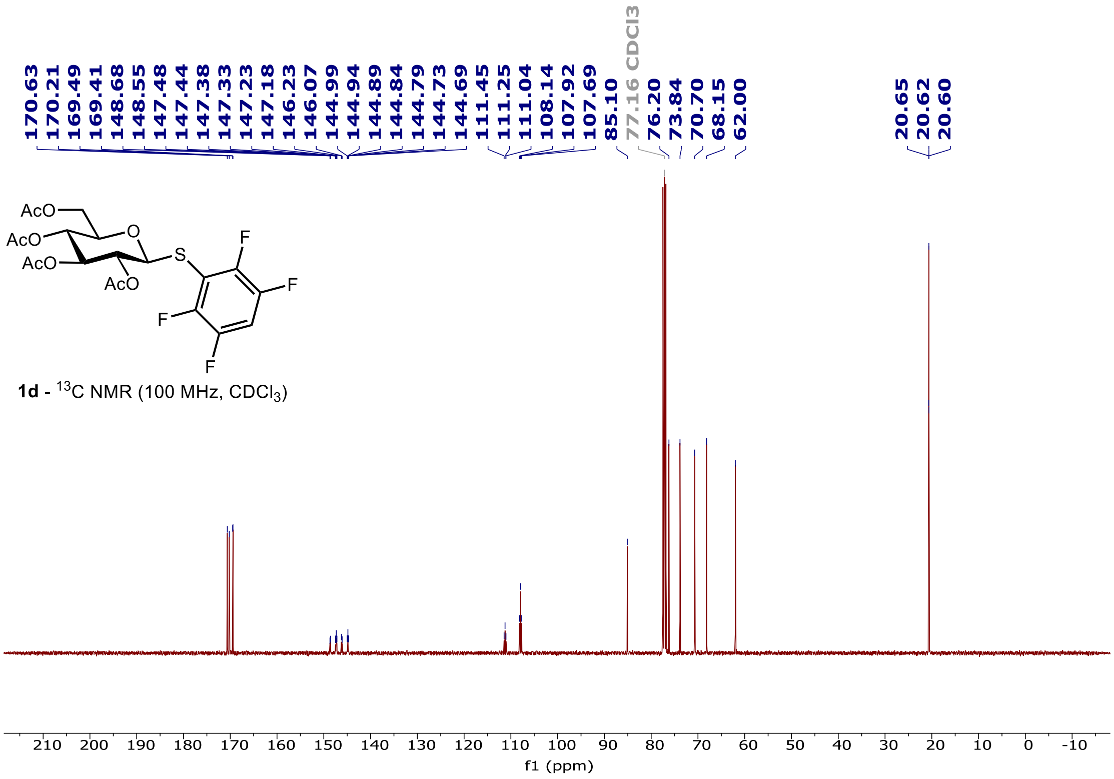

1d - ^{13}C NMR (100 MHz, $CDCl_3$)

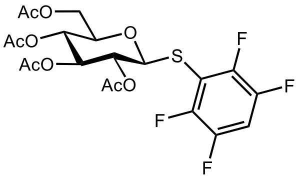

1d - ^{19}F NMR (376 MHz, $CDCl_3$)

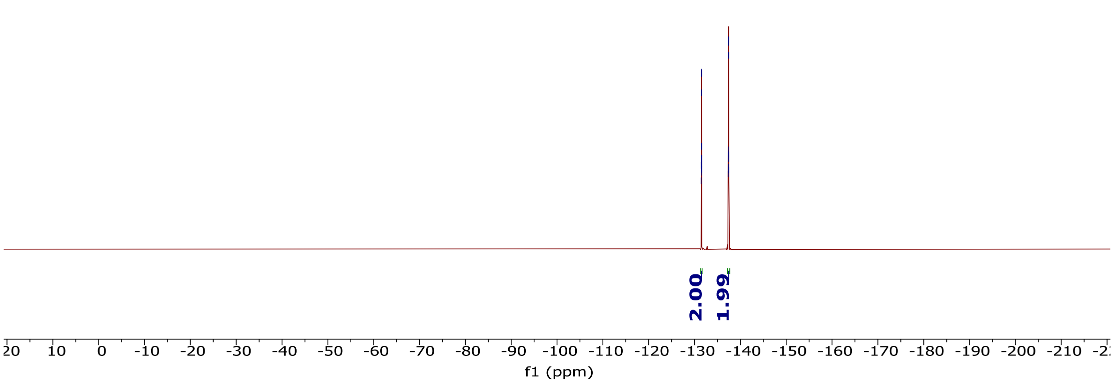

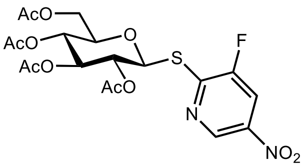

1e - ^{1}H NMR (400 MHz, $CDCl_3$)

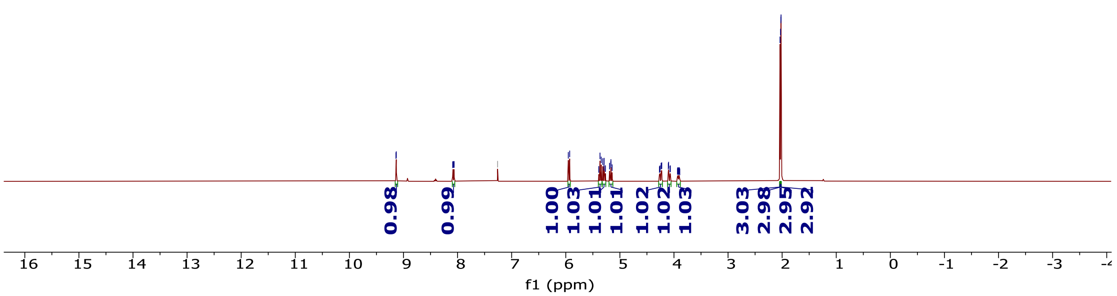

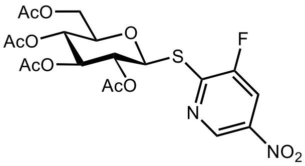

1e - ^{13}C NMR (100 MHz, $CDCl_3$)

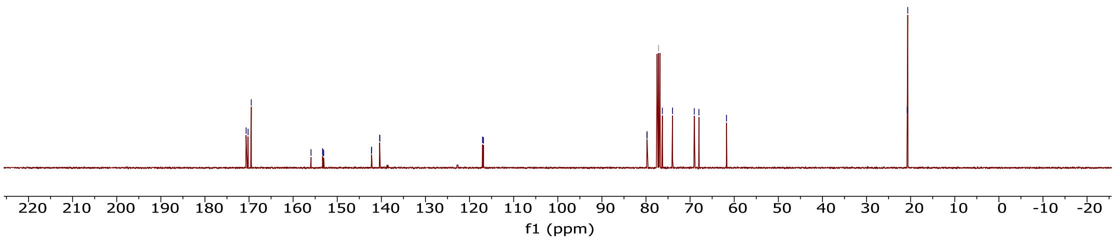

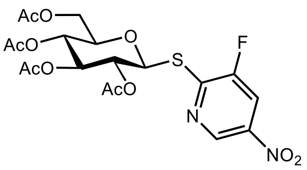

1e - ^{19}F NMR (376 MHz, $CDCl_3$)

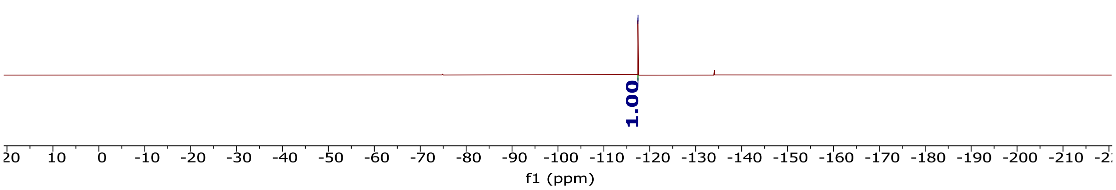

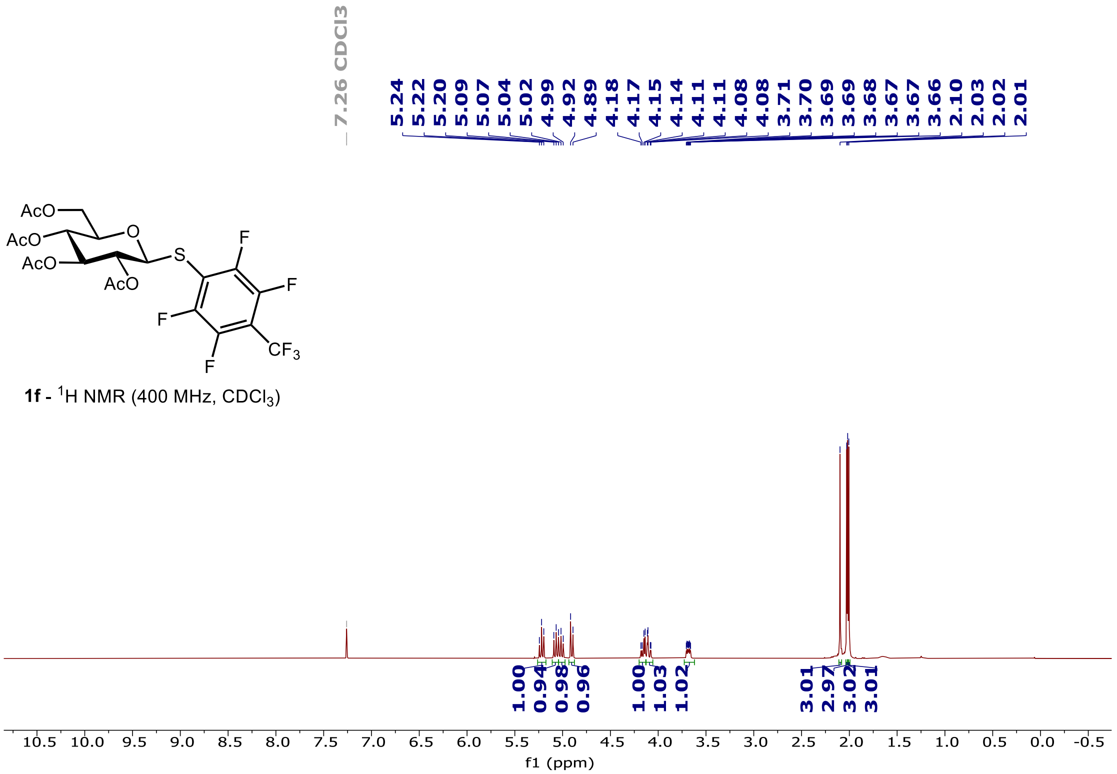
7.26 CDCl3
5.24 5.22 5.20 5.09 5.07 5.04 5.02 4.99 4.92 4.89 4.18 4.17 4.15 4.14 4.11 4.11 4.08 3.71 3.70 3.69 3.69 3.68 3.67 3.67 3.66 2.10 2.03 2.02 2.01
AcO
O
S
F
F
F
F
CF3
1f - 1H NMR (400 MHz, CDCl3)
1.00 0.94 0.98 0.96 1.00 1.03 1.02 3.01 2.97 3.02 3.01
10.5 10.0 9.5 9.0 8.5 8.0 7.5 7.0 6.5 6.0 5.5 5.0 4.5 4.0 3.5 3.0 2.5 2.0 1.5 1.0 0.5 0.0 -0.5
f1 (ppm)

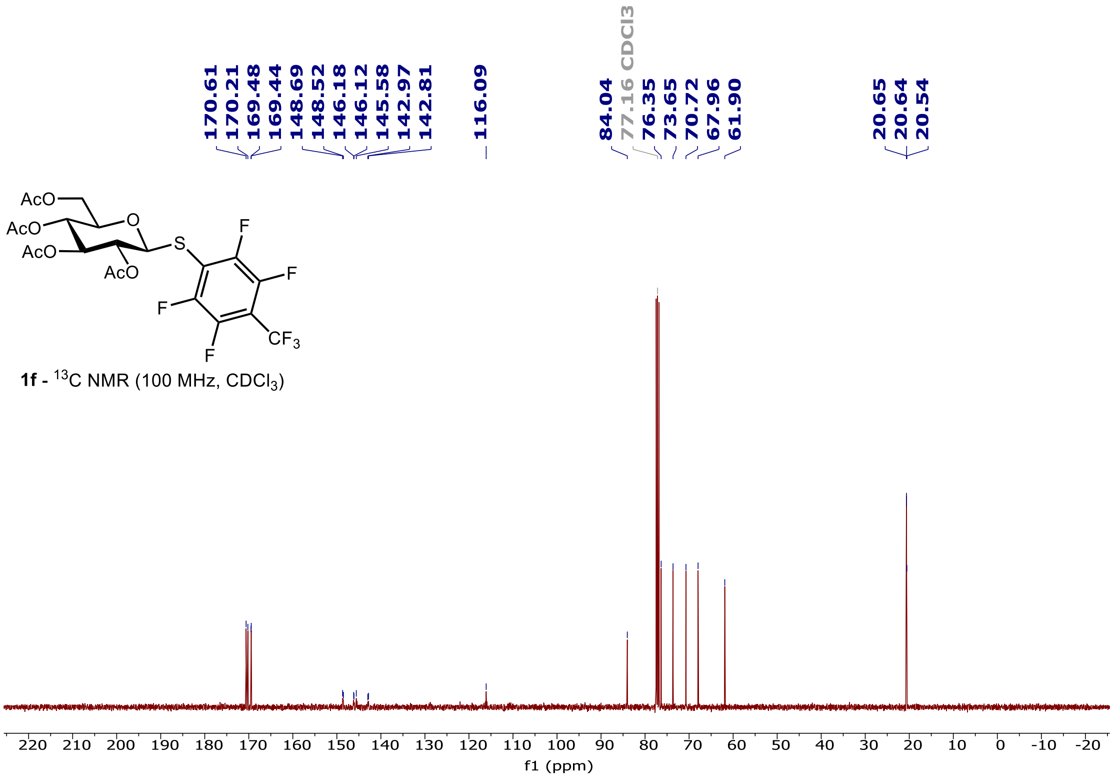
170.61 170.21 169.48 169.44 148.69 148.52 146.18 146.12 145.58 142.97 142.81 116.09
84.04 77.16 CDCl3 76.35 73.65 70.72 67.96 61.90
20.65 20.64 20.54
AcO
O
S
F
F
F
F
CF3
1f - 13C NMR (100 MHz, CDCl3)
220 210 200 190 180 170 160 150 140 130 120 110 100 90 80 70 60 50 40 30 20 10 0 -10 -20
f1 (ppm)

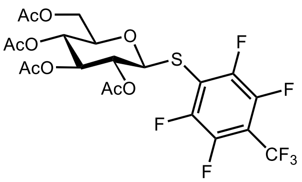

1f - ^{19}F NMR (376 MHz, $CDCl_3$)

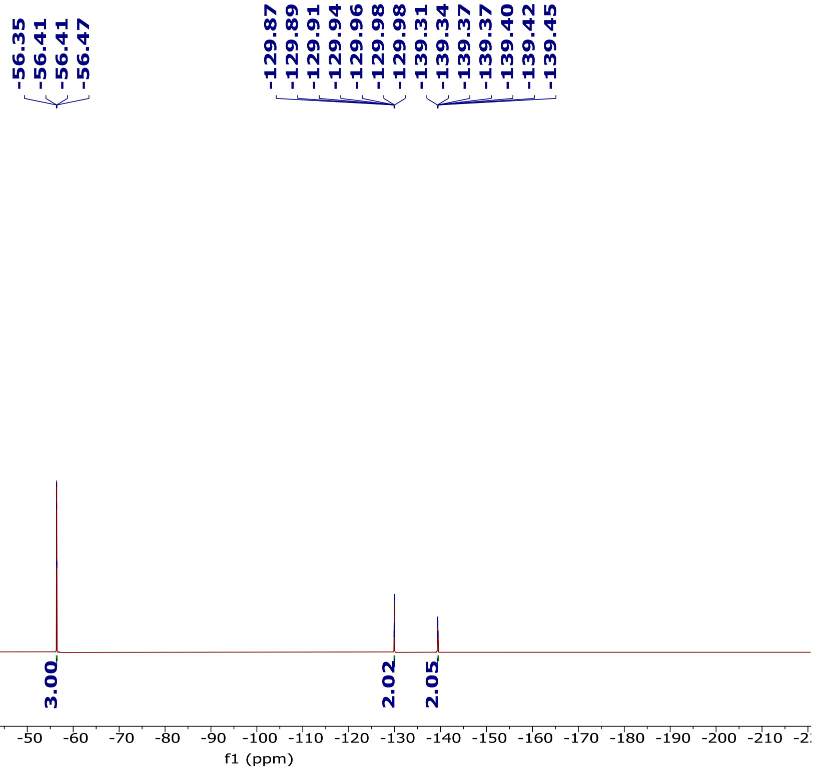

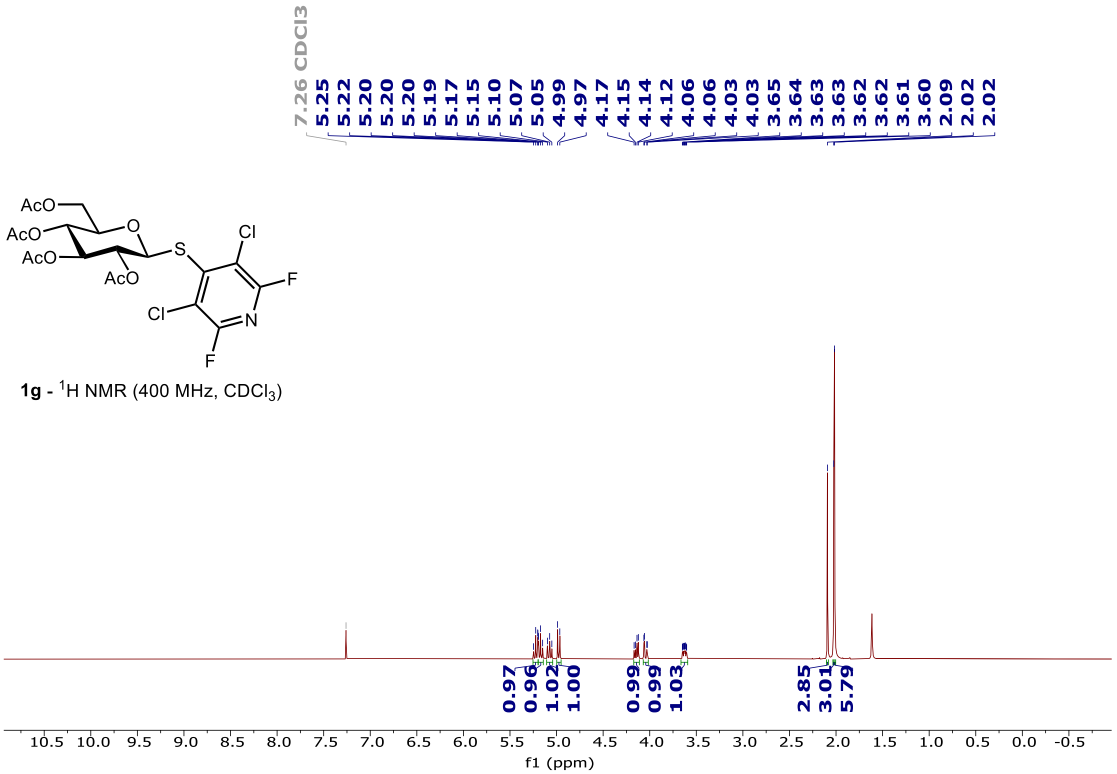

1g - ^{1}H NMR (400 MHz, $CDCl_3$)

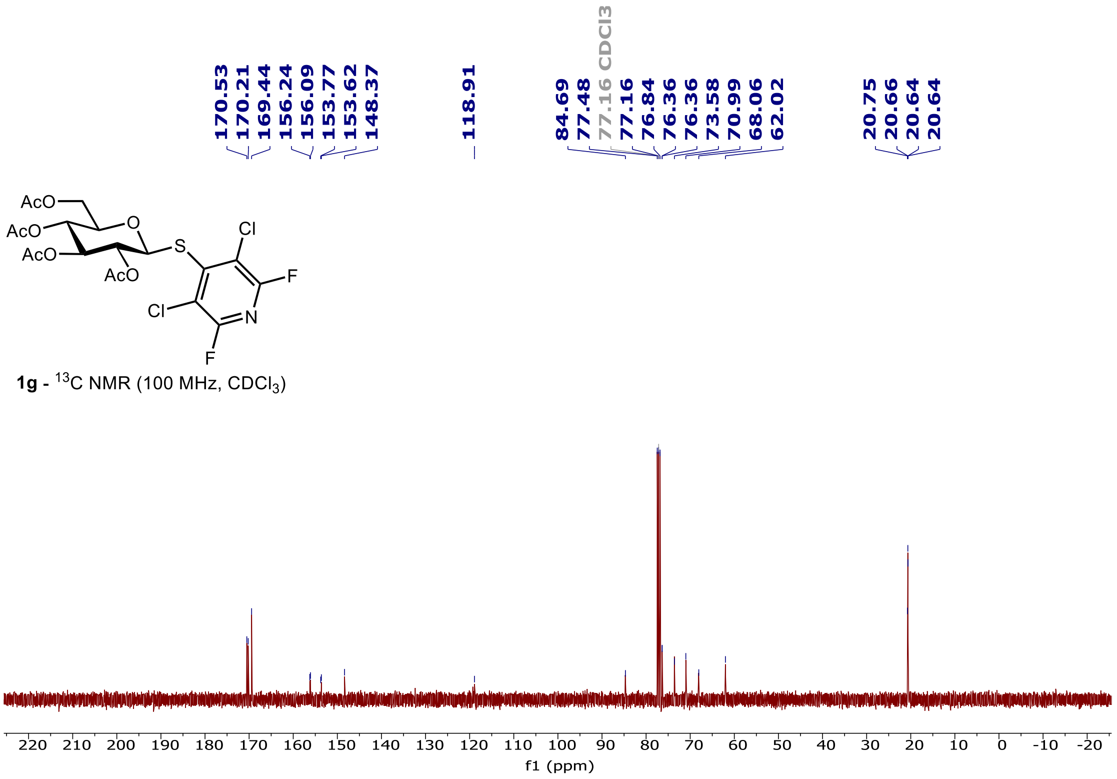

1g - ^{13}C NMR (100 MHz, $CDCl_3$)

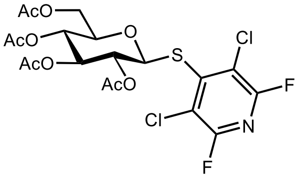

1g - ^{19}F NMR (376 MHz, $CDCl_3$)

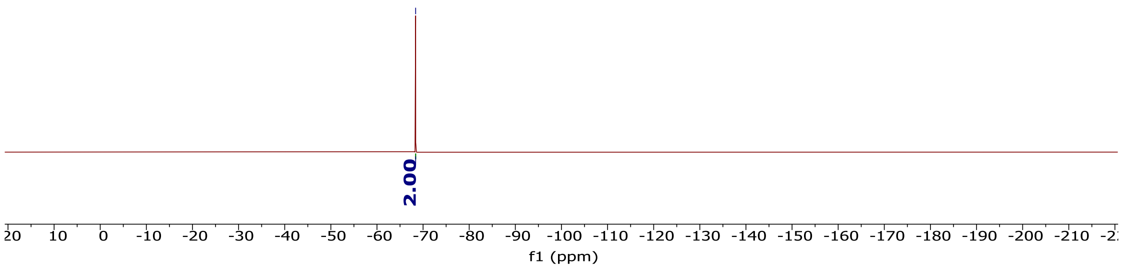

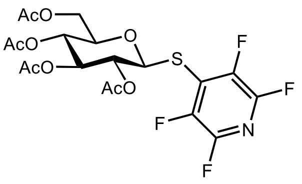

1h - ^{1}H NMR (400 MHz, $CDCl_3$)

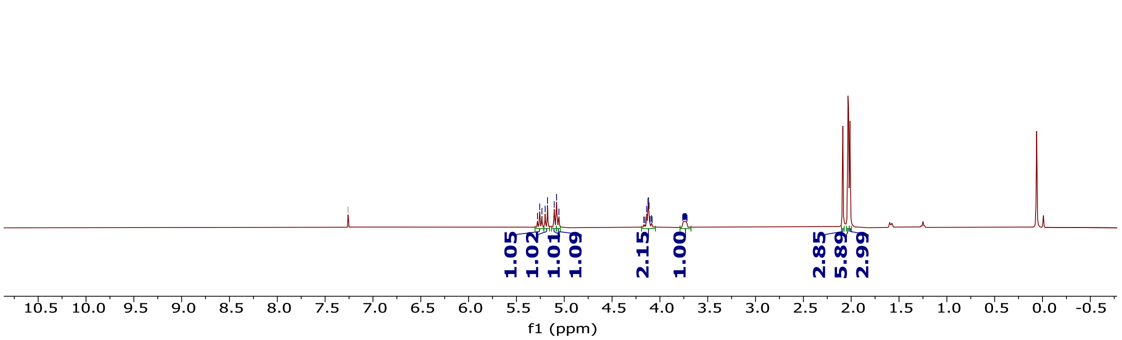

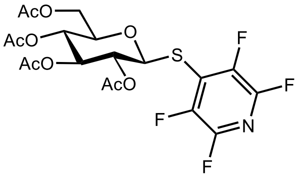

1h - ^{13}C NMR (100 MHz, $CDCl_3$)

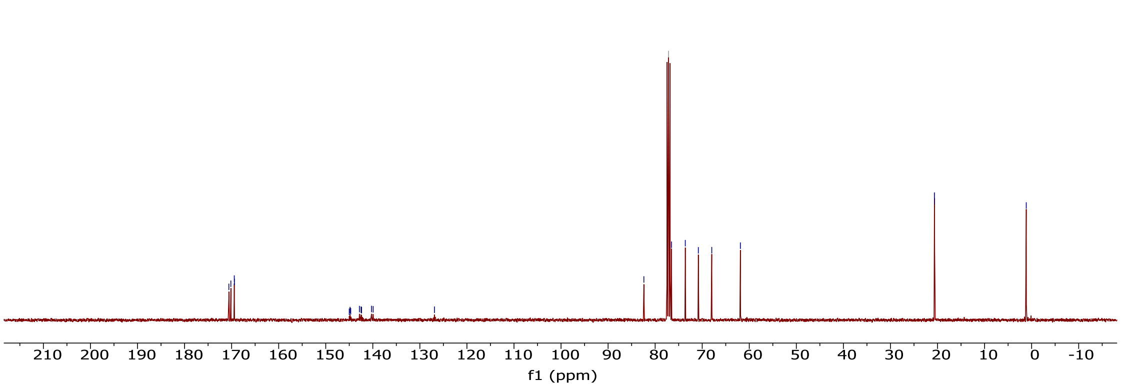

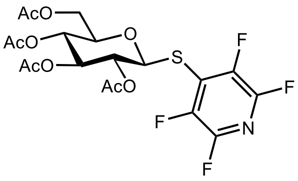

1h - ^{19}F NMR (376 MHz, $CDCl_3$)

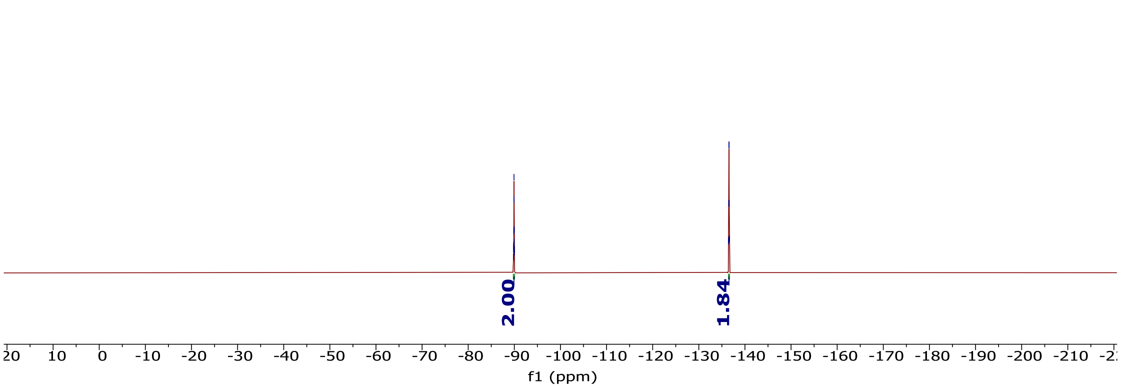

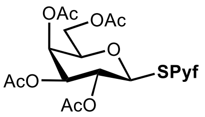

S3 - ^{1}H NMR (400 MHz, $CDCl_3$)

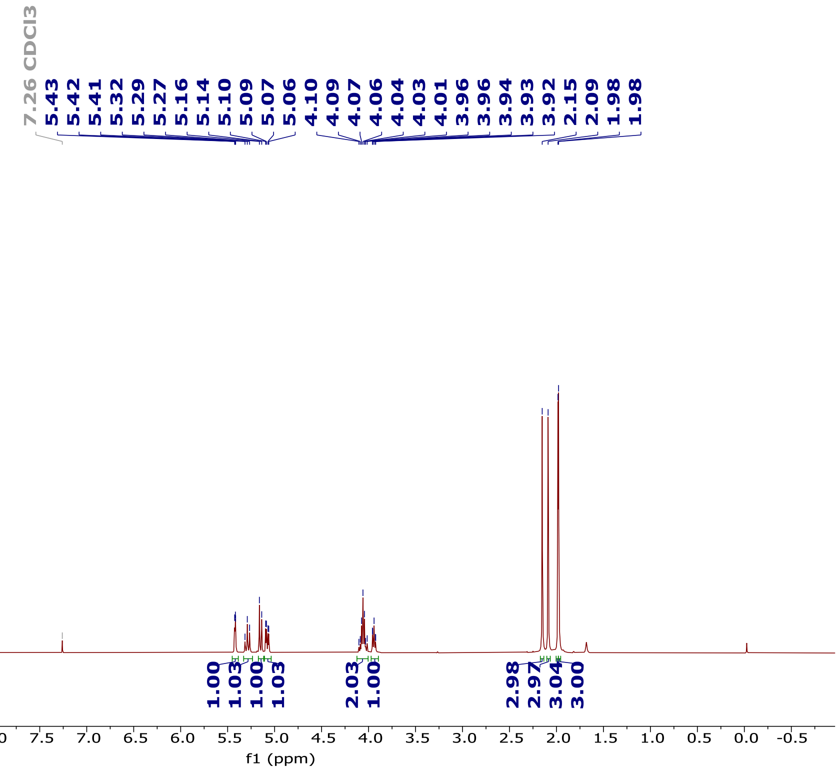

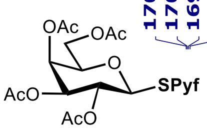

S3- ^{13}C NMR (100 MHz, $CDCl_3$)

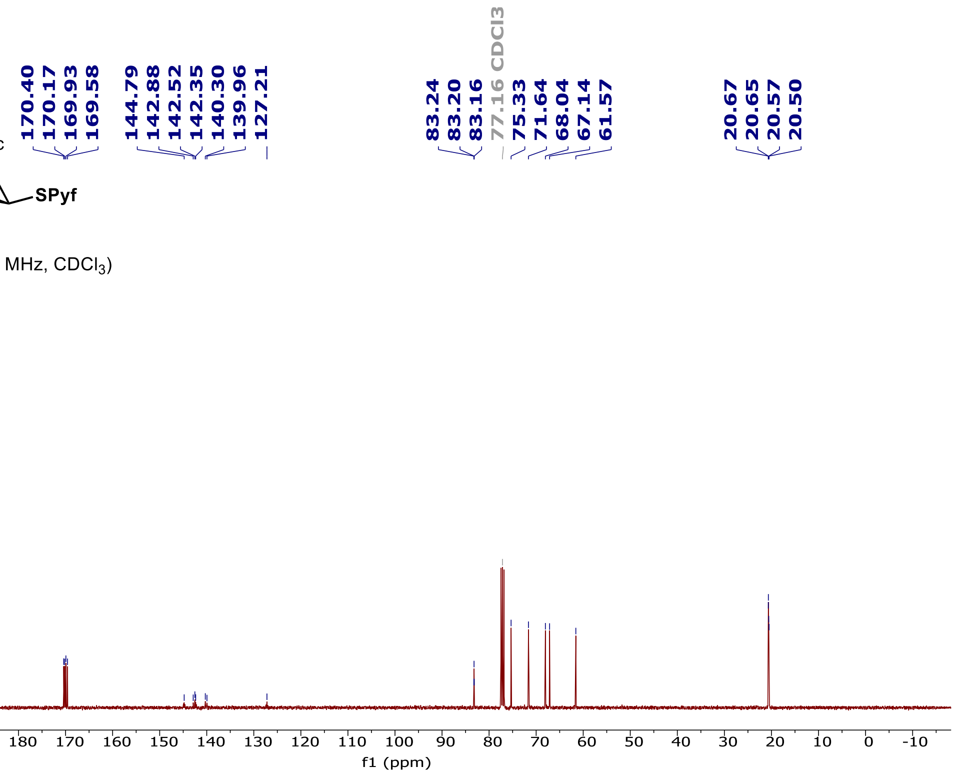

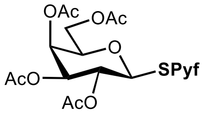

S3 - ^{19}F NMR (376 MHz, $CDCl_3$)

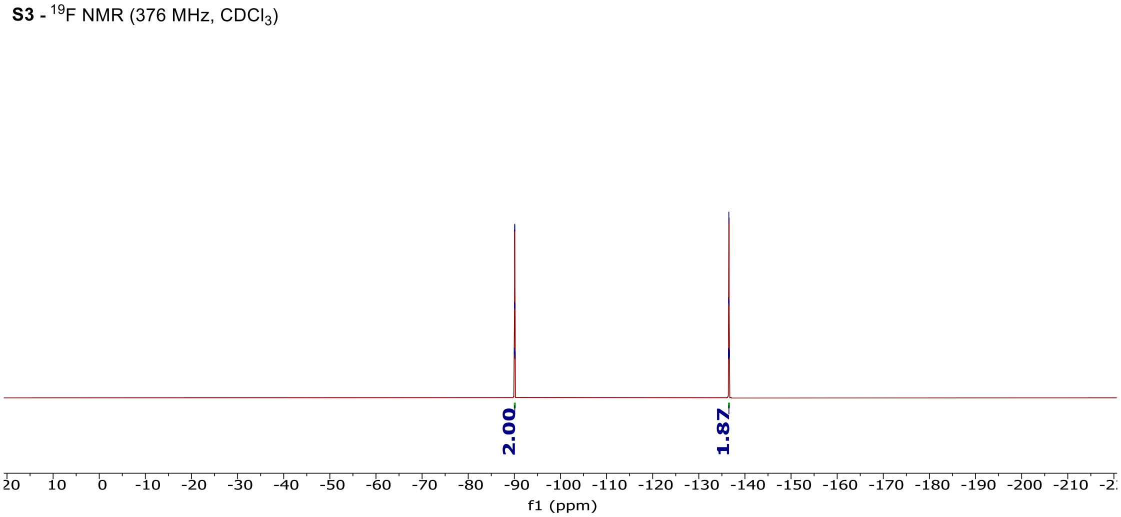

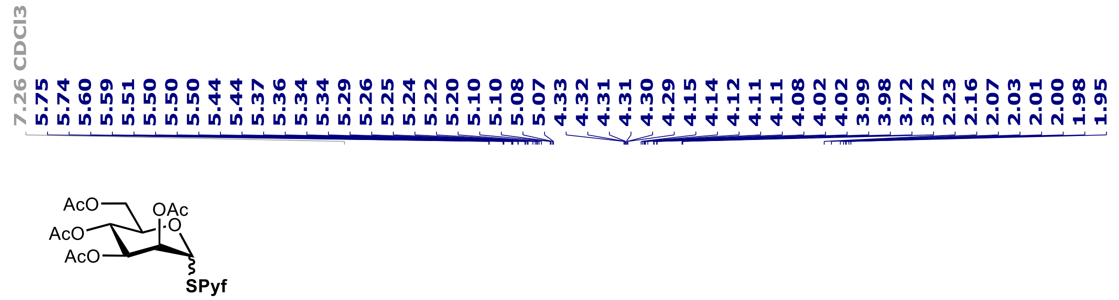

S4 - ^{1}H NMR (400 MHz, $CDCl_3$)

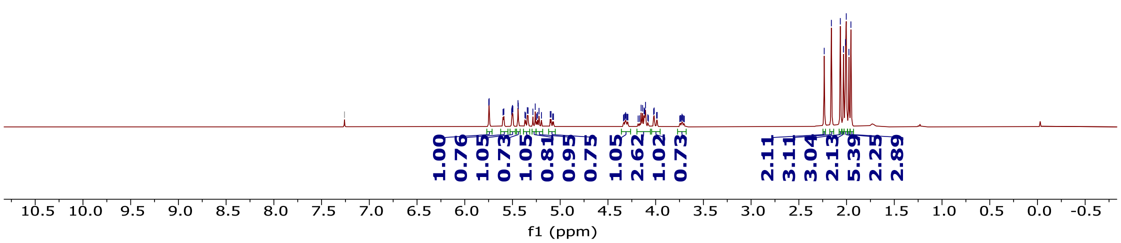

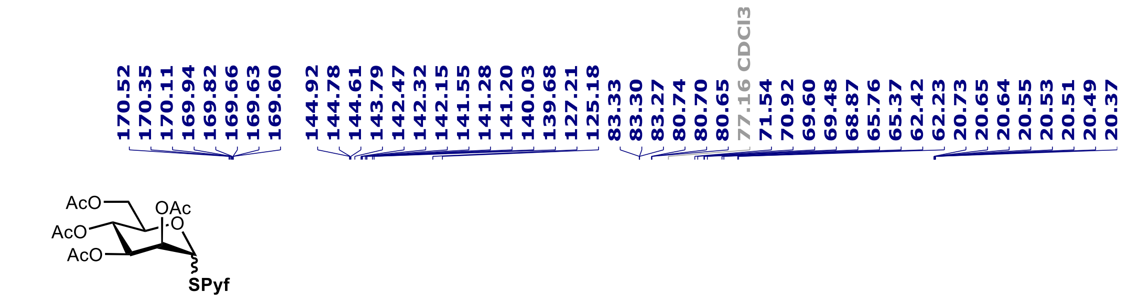

S4 - ^{13}C NMR (100 MHz, $CDCl_3$)

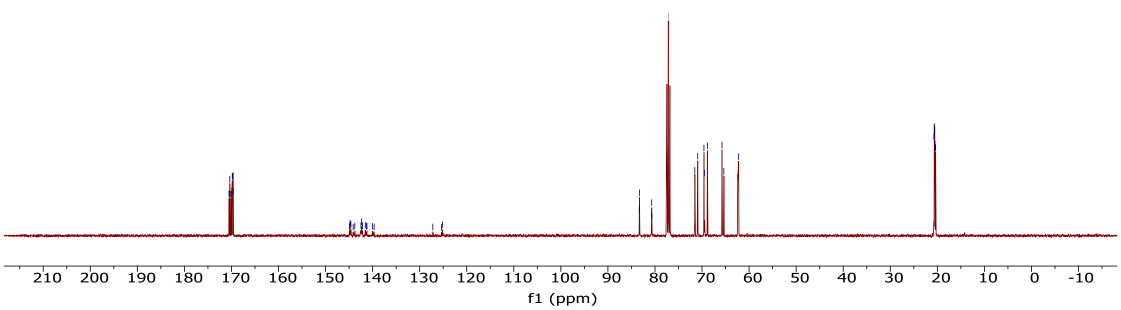

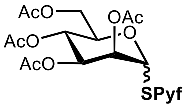

S4 - ^{19}F NMR (376 MHz, $CDCl_3$)

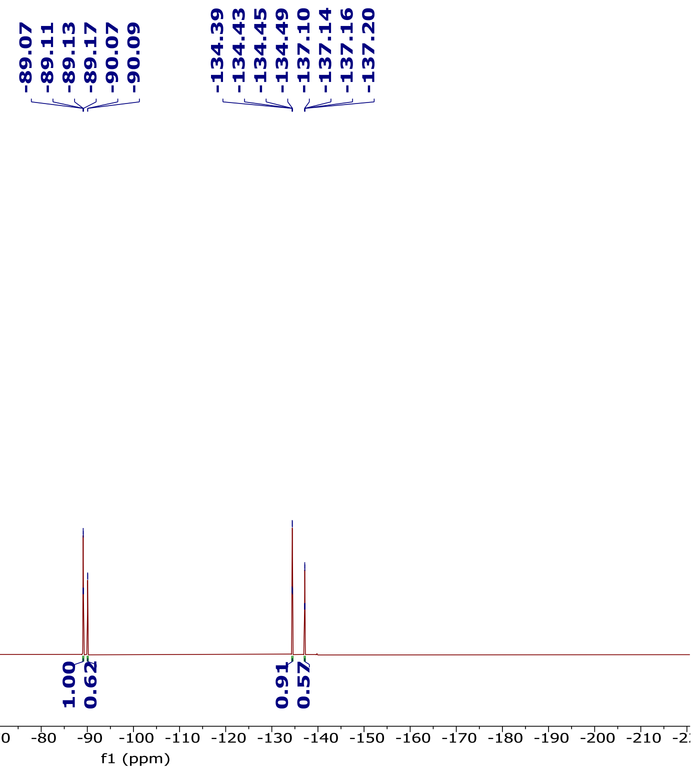

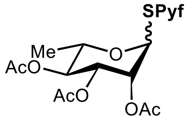

S5 - ^{1}H NMR (400 MHz, $CDCl_3$)

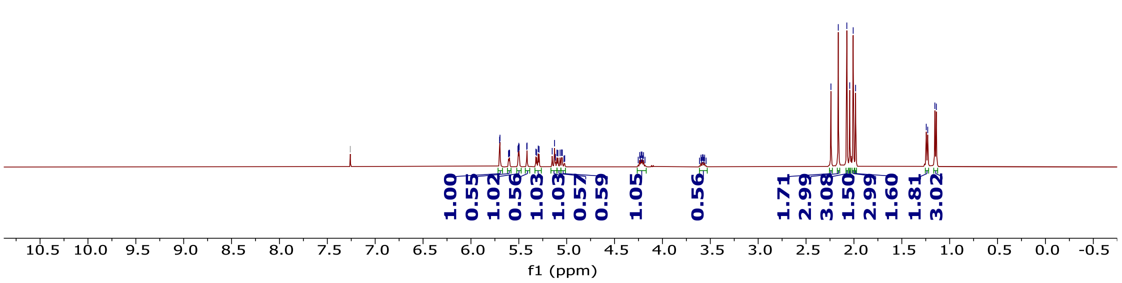

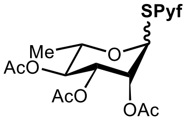

S5 - ^{13}C NMR (100 MHz, $CDCl_3$)

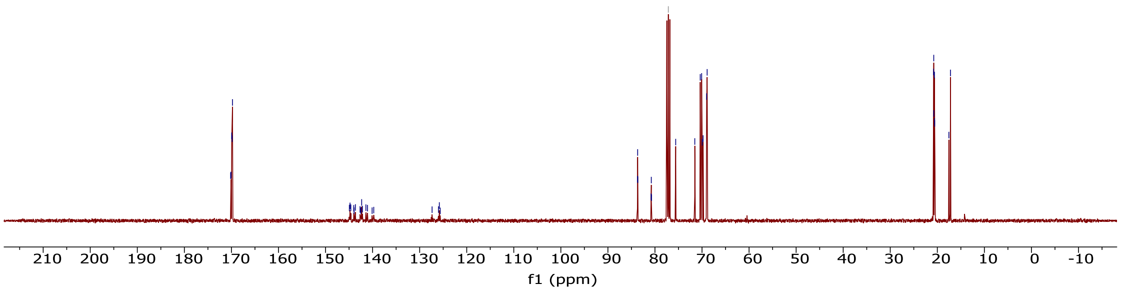

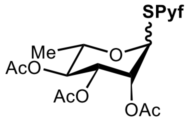

S5 - ^{19}F NMR (376 MHz, $CDCl_3$)

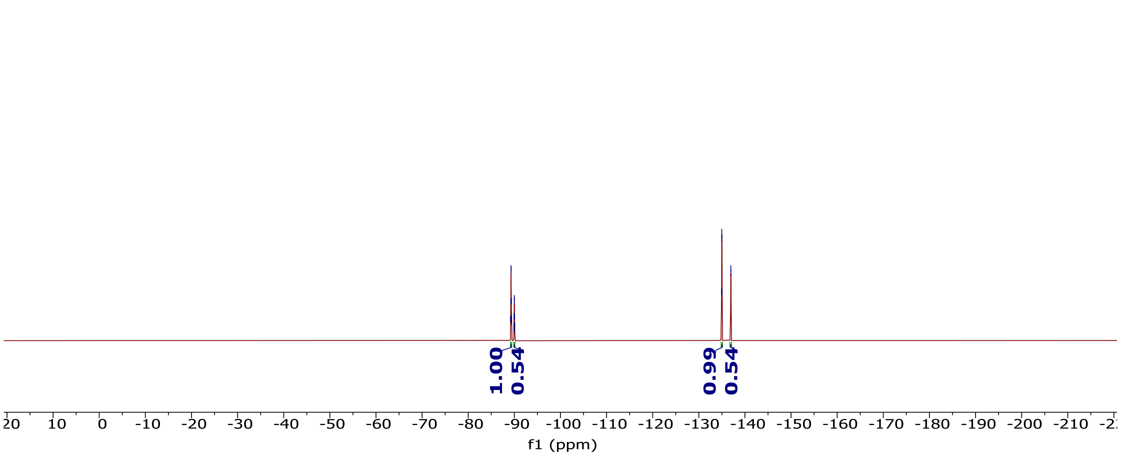

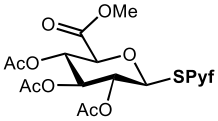

S6 - ^{1}H NMR (400 MHz, $CDCl_3$)

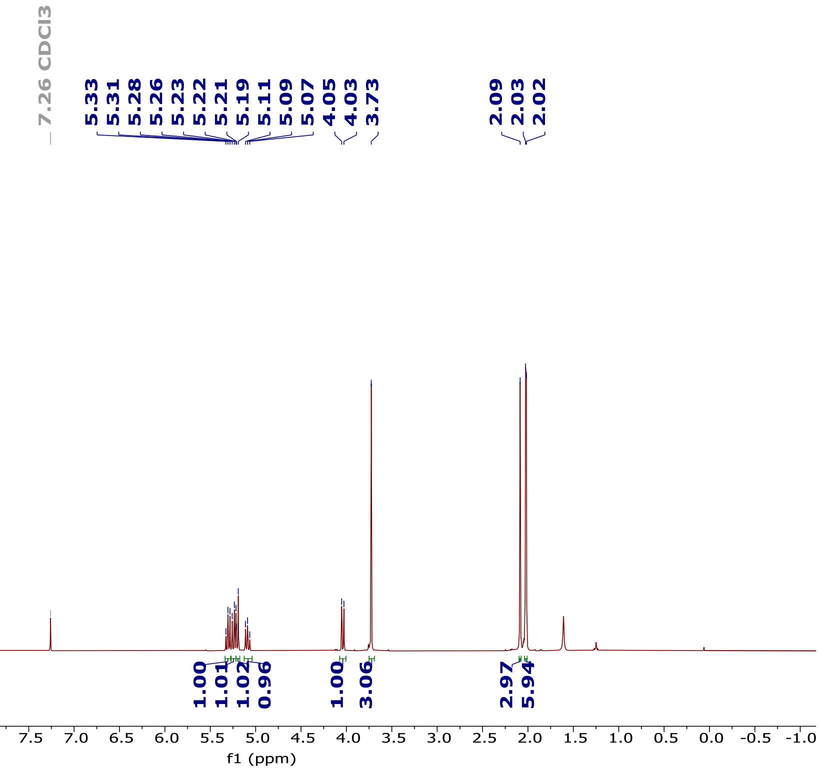

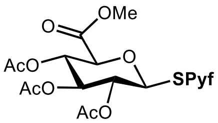

S6 - ^{13}C NMR (100 MHz, $CDCl_3$)

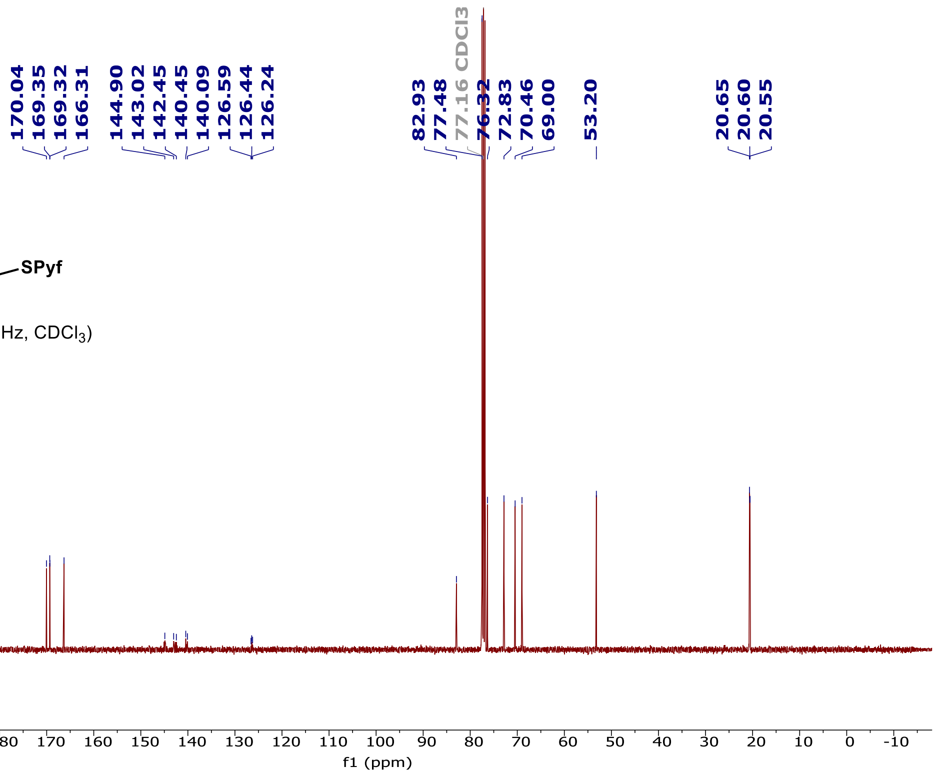

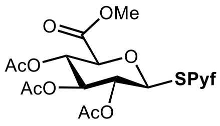

S6 - ^{19}F NMR (376 MHz, $CDCl_3$)

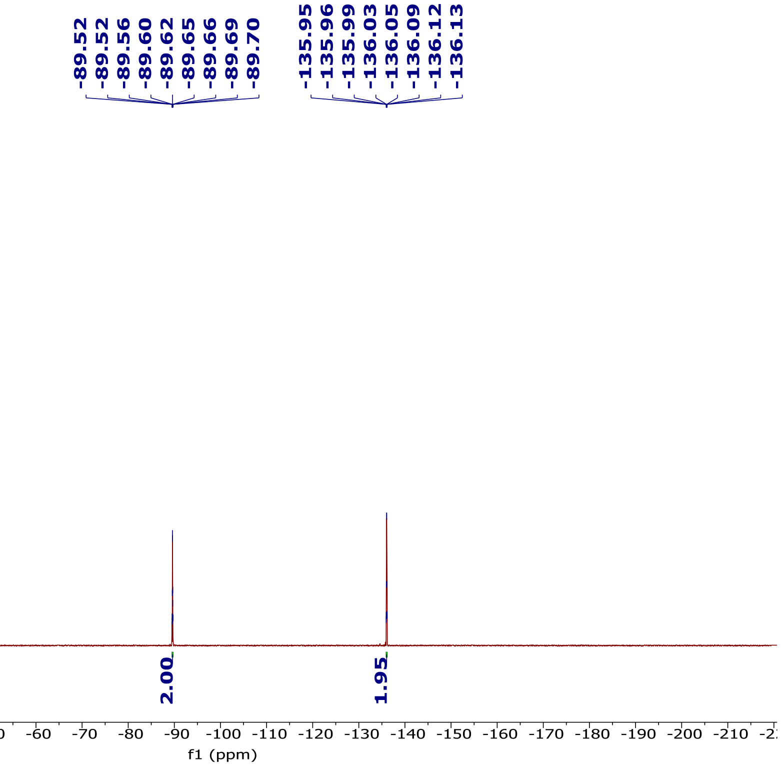

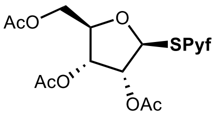

S7 - ^{1}H NMR (400 MHz, $CDCl_3$)

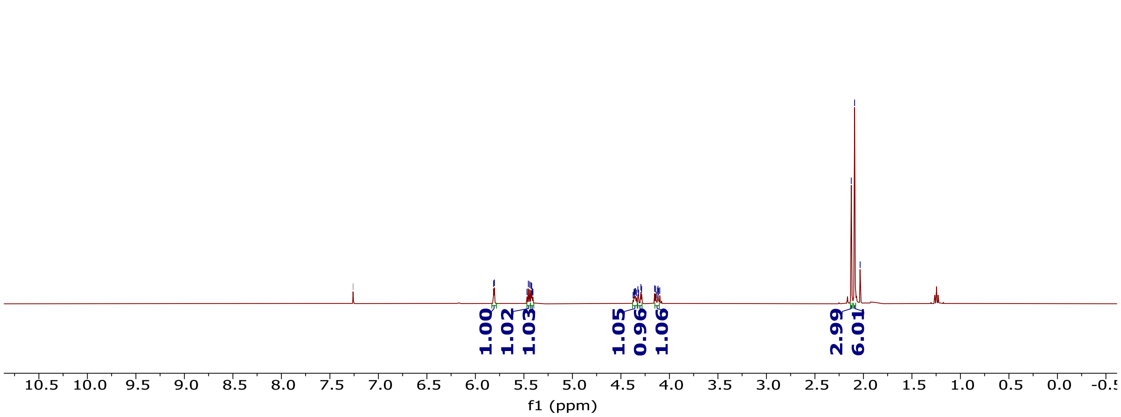

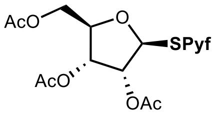

S7 - ^{13}C NMR (100 MHz, $CDCl_3$)

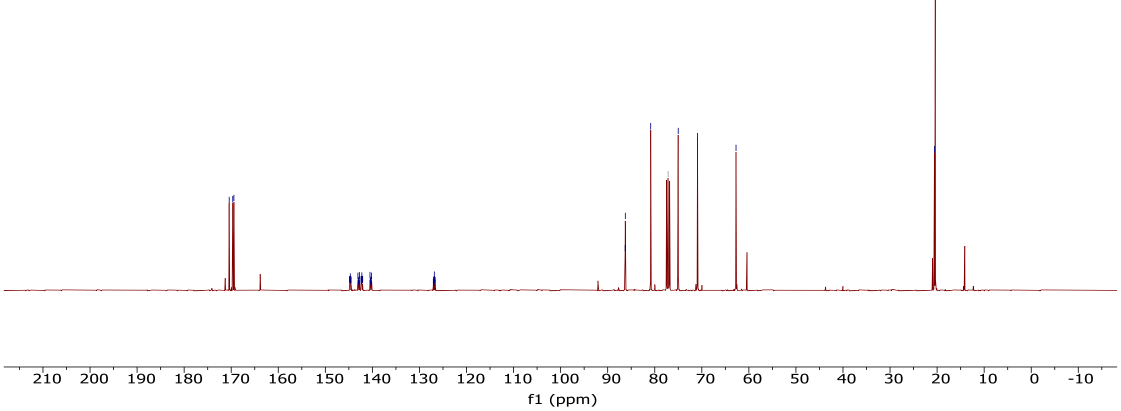

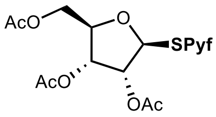

S7 - ^{19}F NMR (376 MHz, $CDCl_3$)

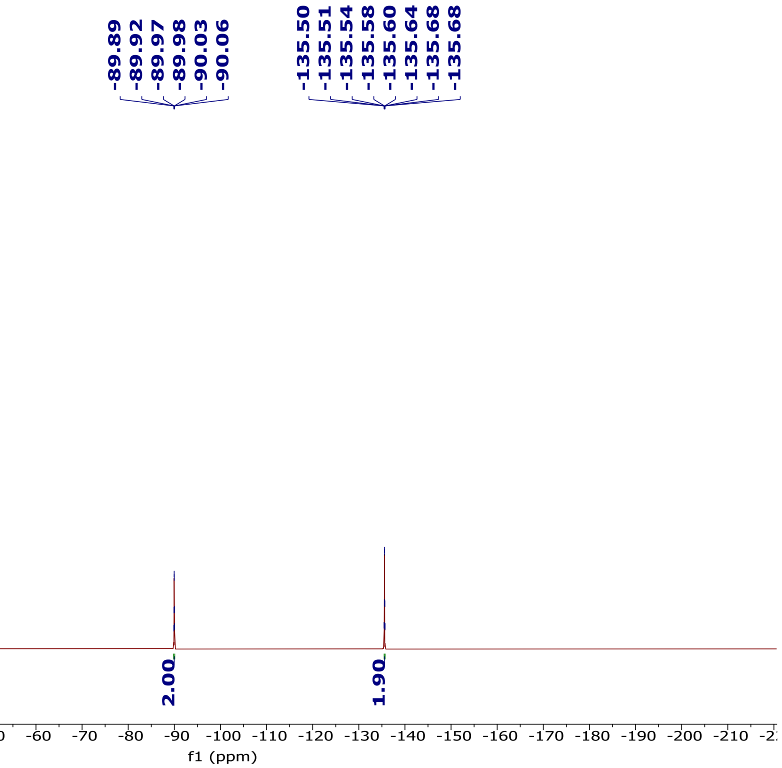

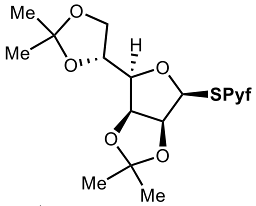

S8 - ^{1}H NMR (400 MHz, $CDCl_3$)

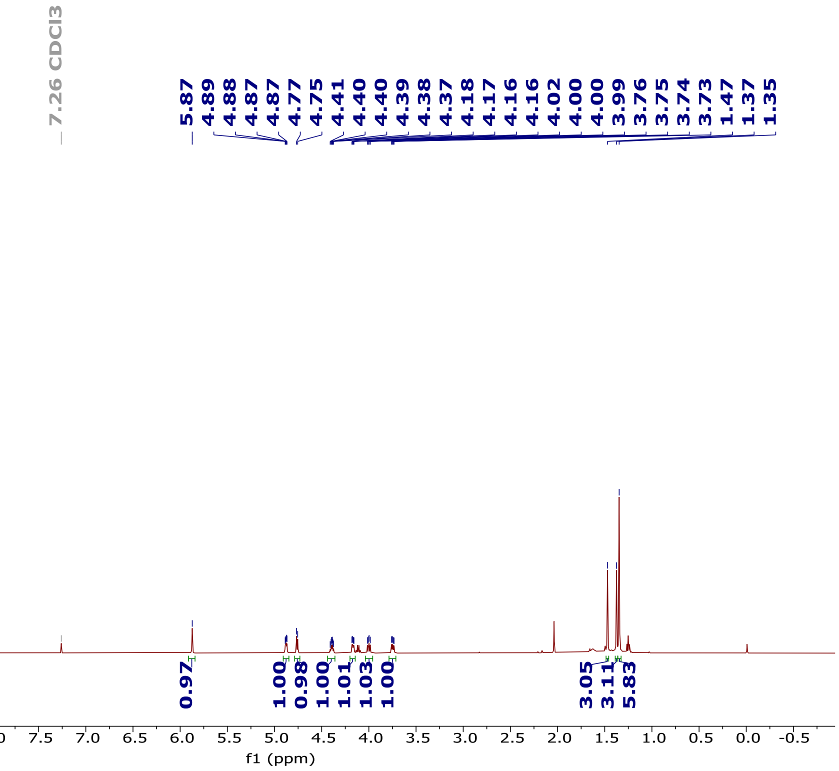

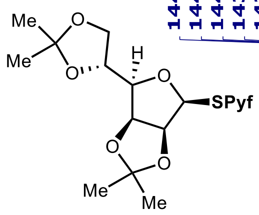

S8 - ^{13}C NMR (100 MHz, $CDCl_3$)

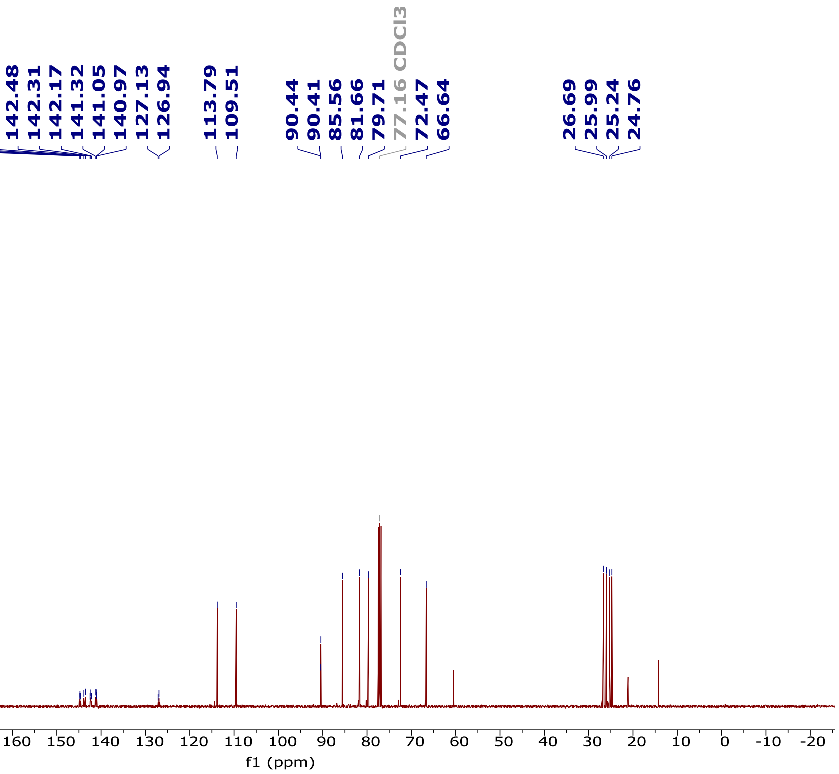

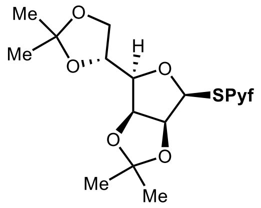

S8 - ^{19}F NMR (376 MHz, $CDCl_3$)

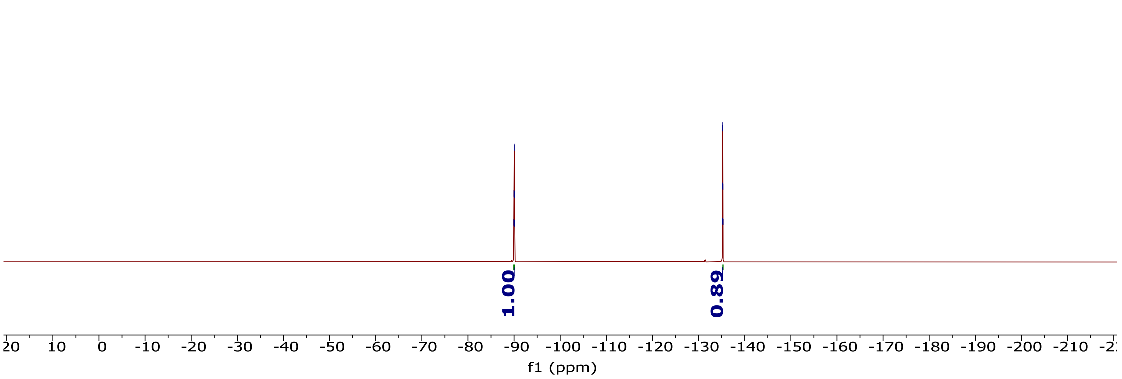

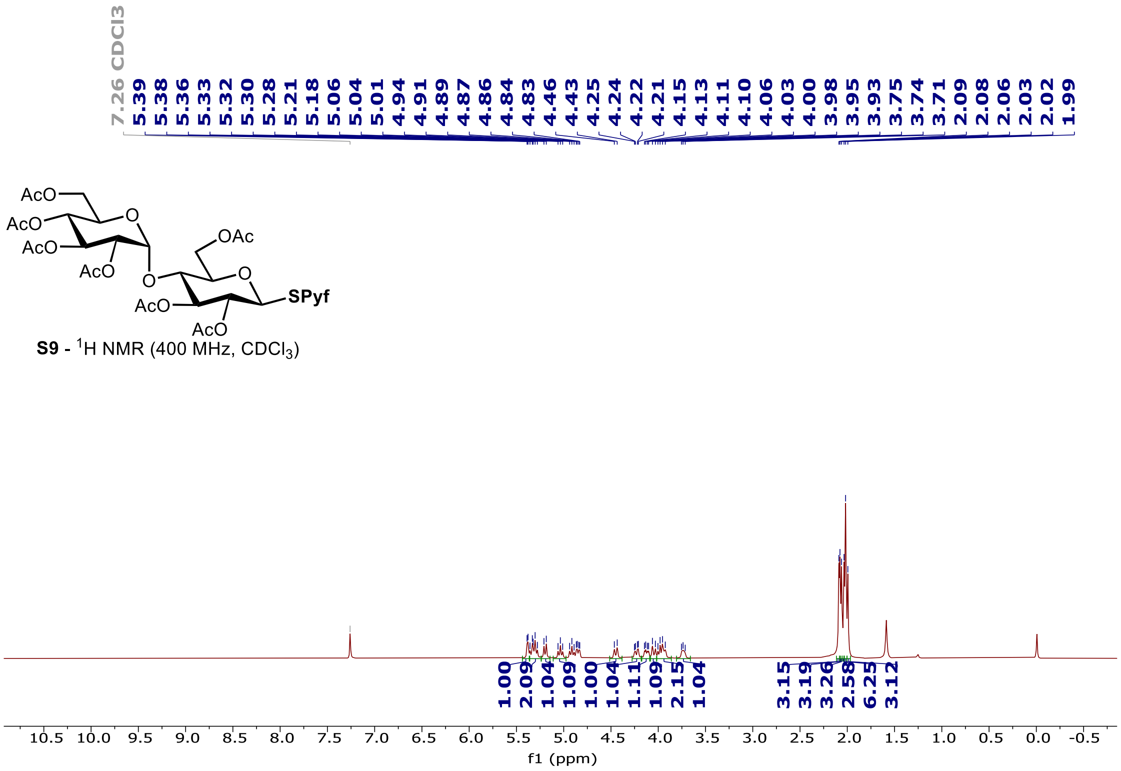

S9 - ^{1}H NMR (400 MHz, $CDCl_3$)

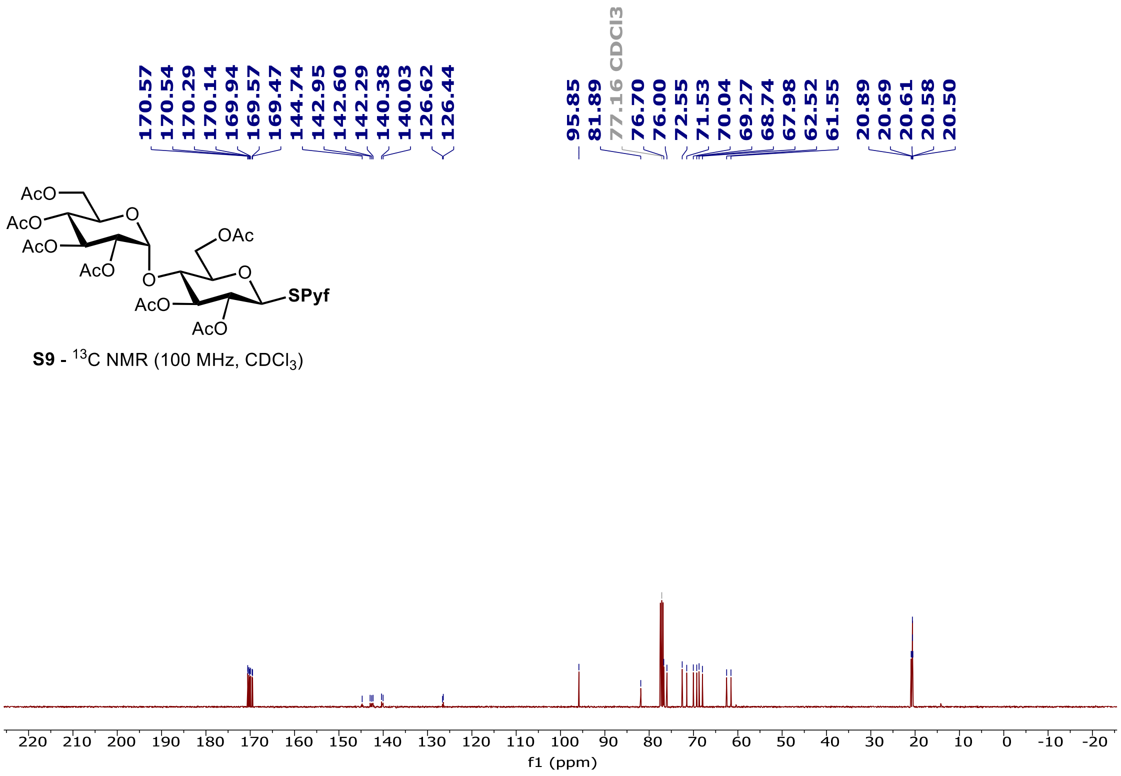

S9 - ^{13}C NMR (100 MHz, $CDCl_3$)

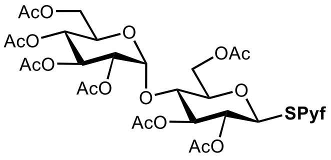

S9 - ^{19}F NMR (376 MHz, $CDCl_3$)

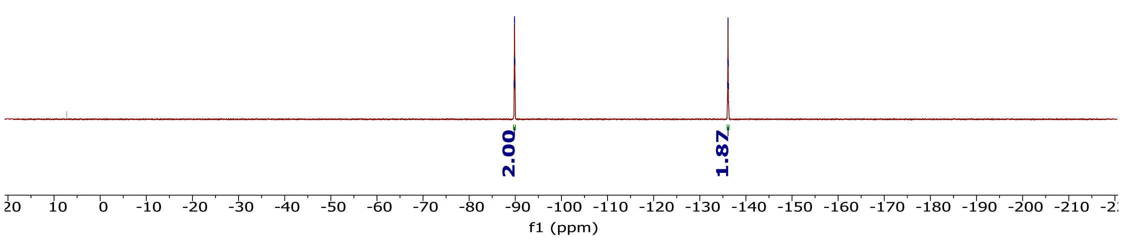

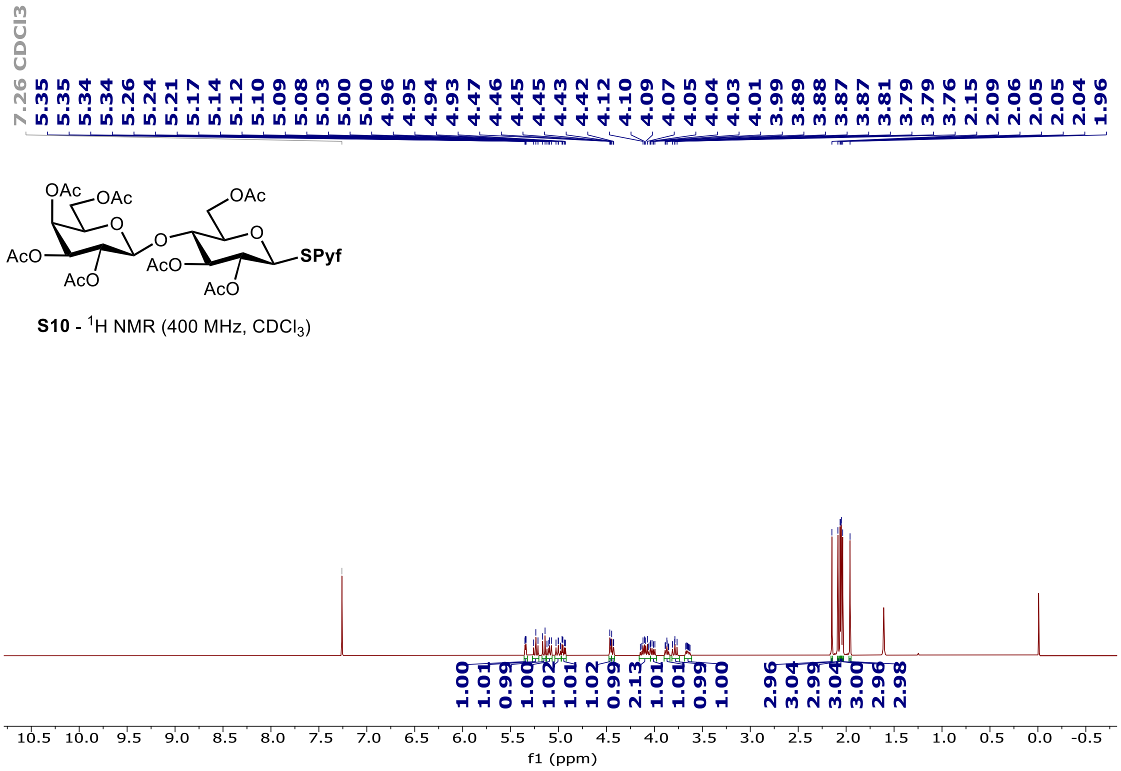

S10 - ^{1}H NMR (400 MHz, $CDCl_3$)

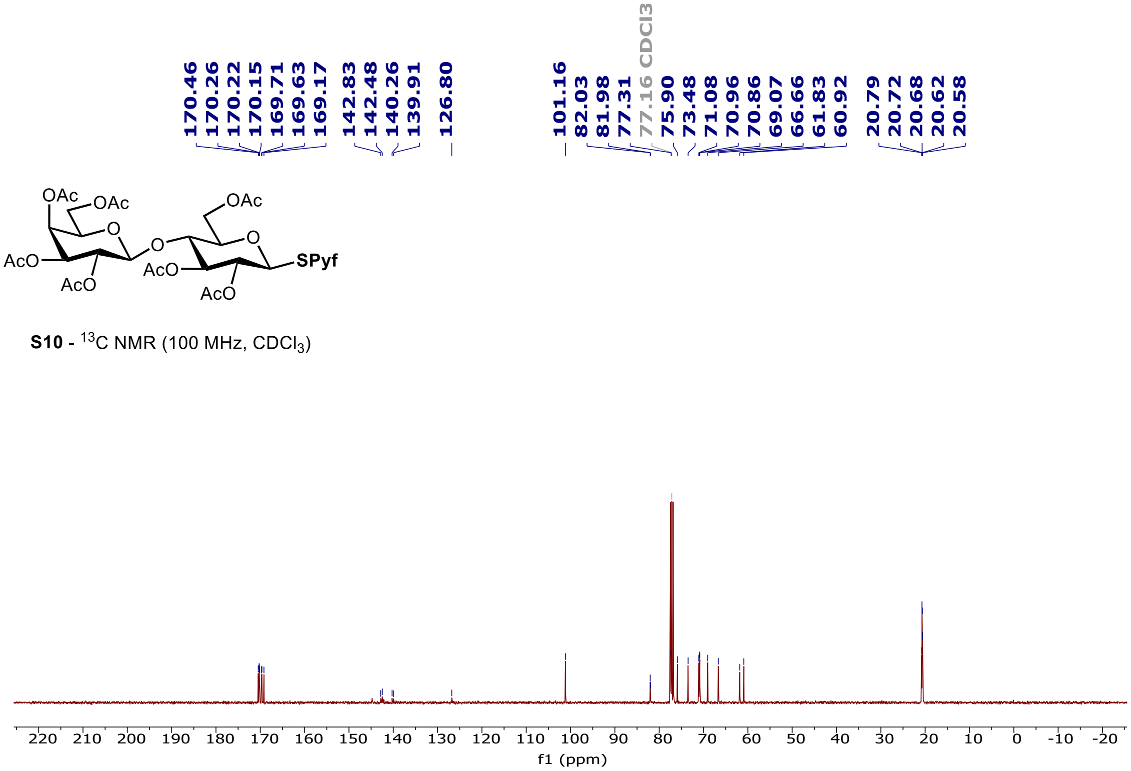

S10 - ^{13}C NMR (100 MHz, $CDCl_3$)

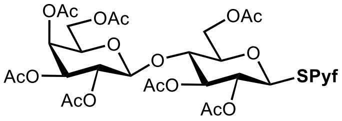

S10 - ^{19}F NMR (376 MHz, $CDCl_3$)

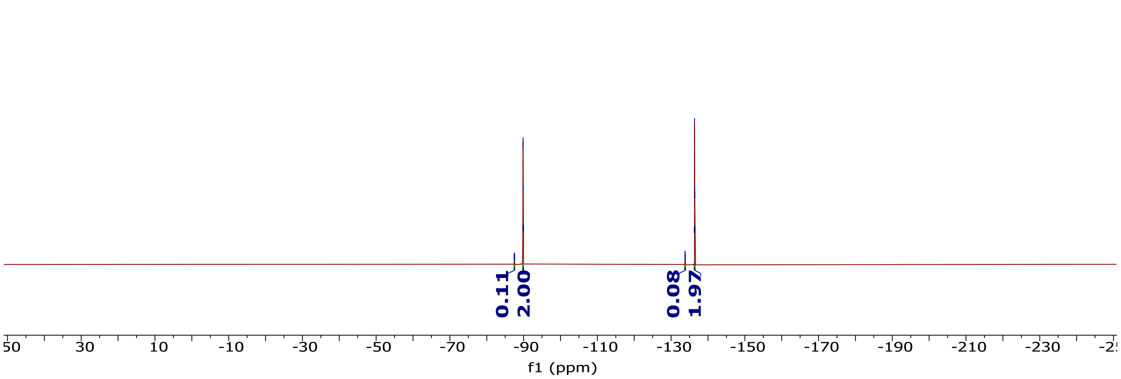

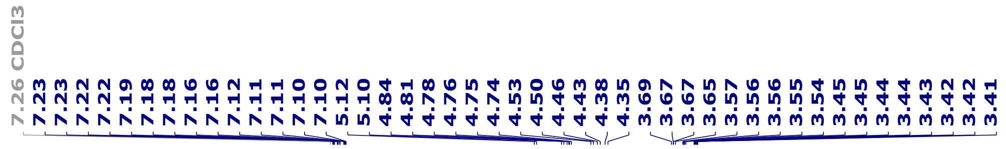

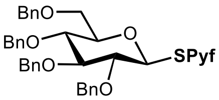

S11- ^{1}H NMR (400 MHz, $CDCl_3$)

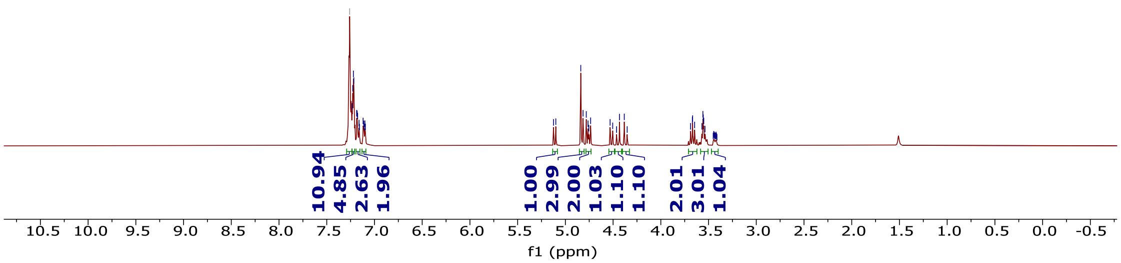

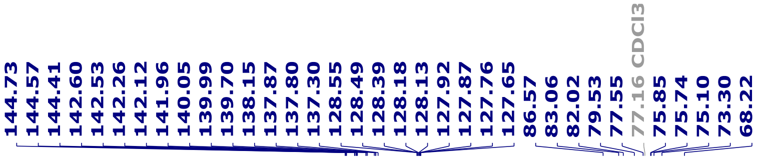

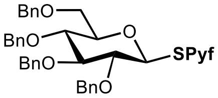

S11 - ^{13}C NMR (100 MHz, $CDCl_3$)

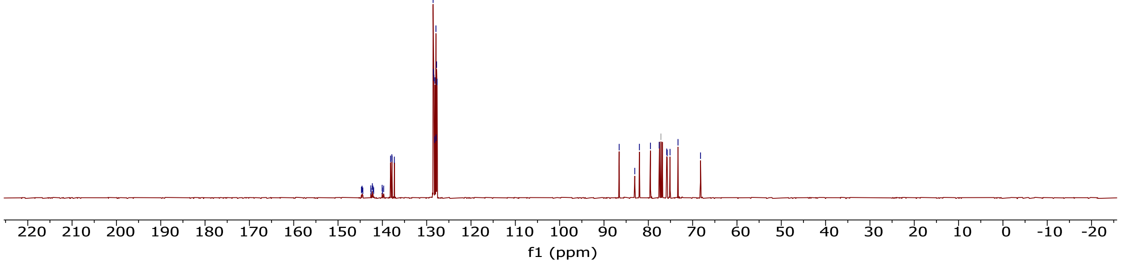

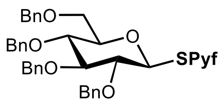

S11 - ^{19}F NMR (376 MHz, $CDCl_3$)

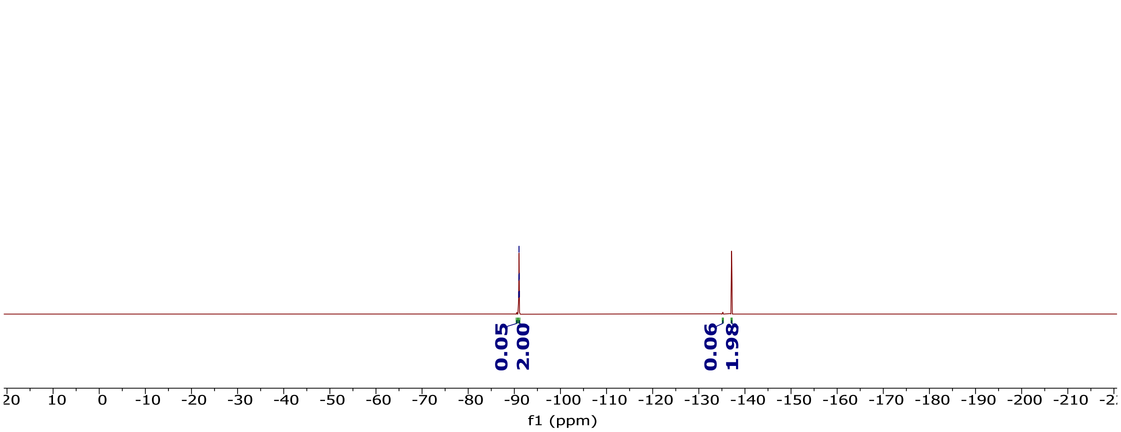

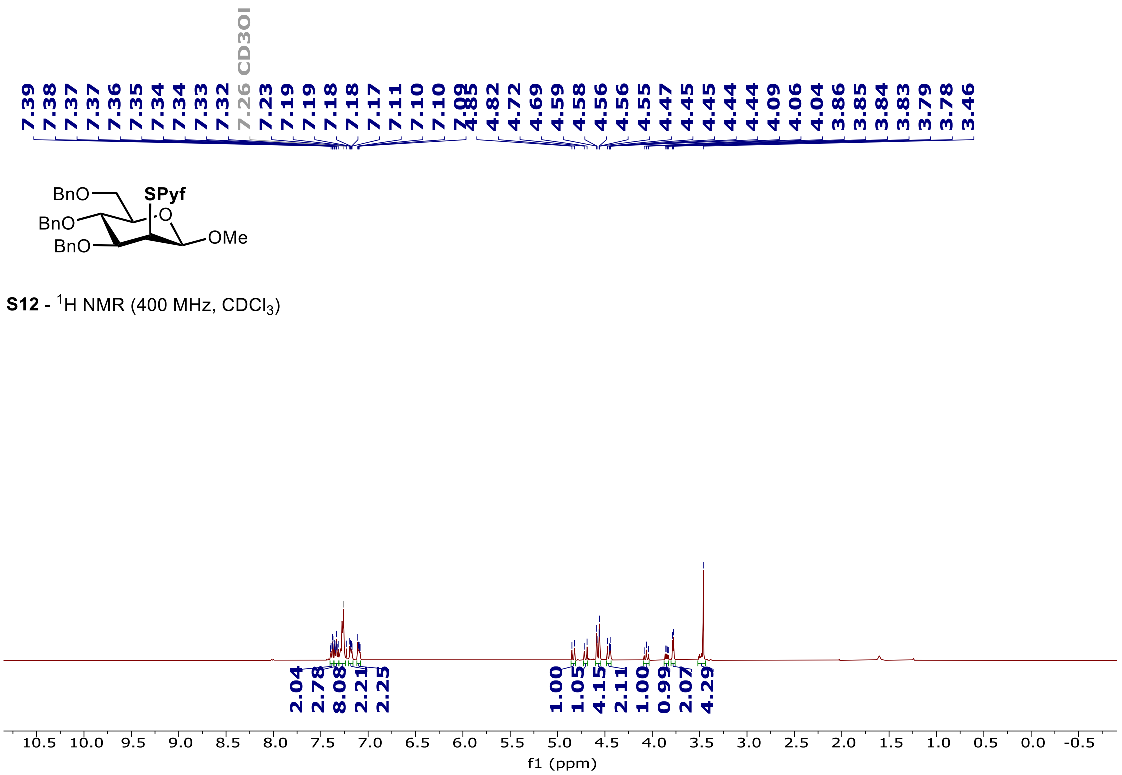

S12 - ^{1}H NMR (400 MHz, $CDCl_3$)

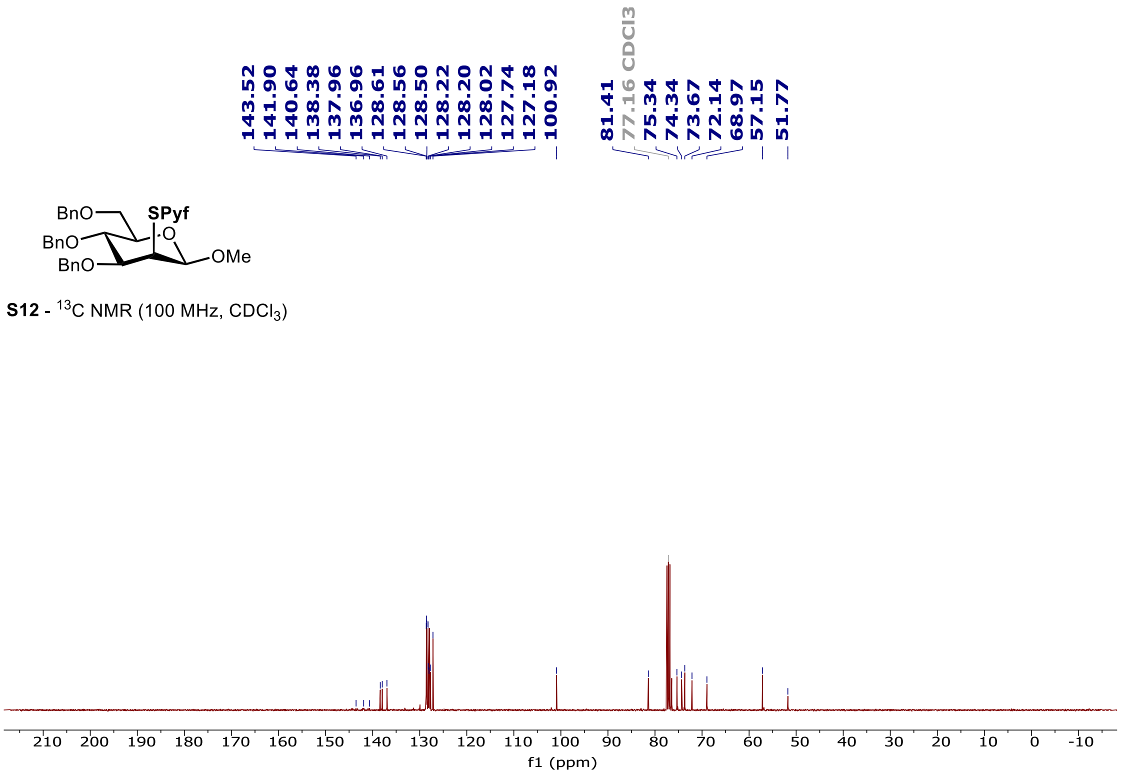

S12 - ^{13}C NMR (100 MHz, $CDCl_3$)

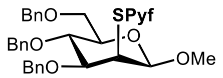

S12 - ^{19}F NMR (376 MHz, $CDCl_3$)

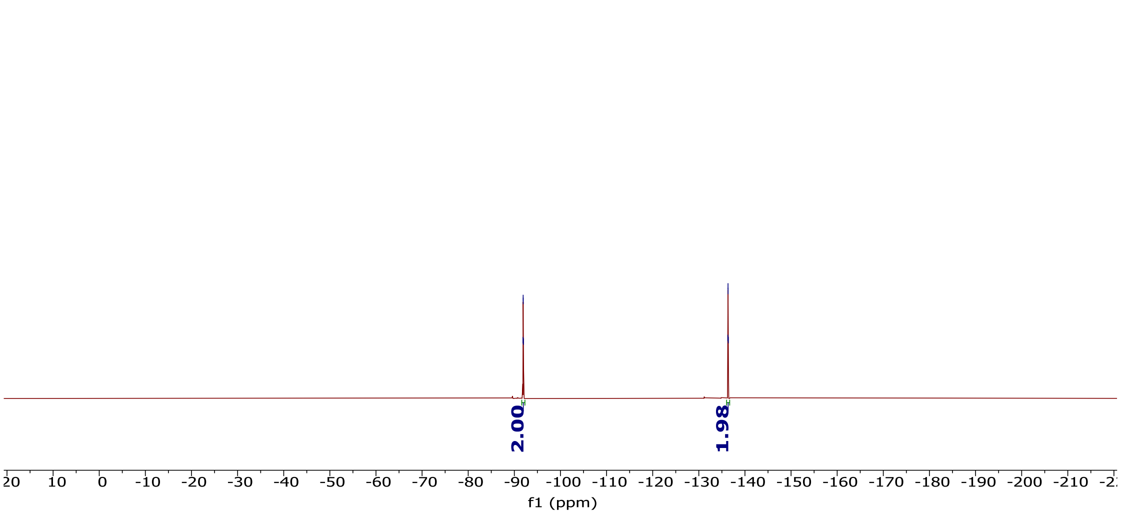

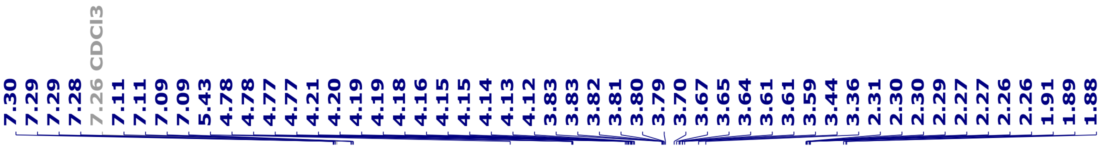

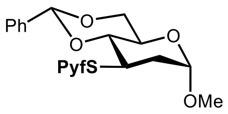

S13 - ^{1}H NMR (400 MHz, $CDCl_3$)

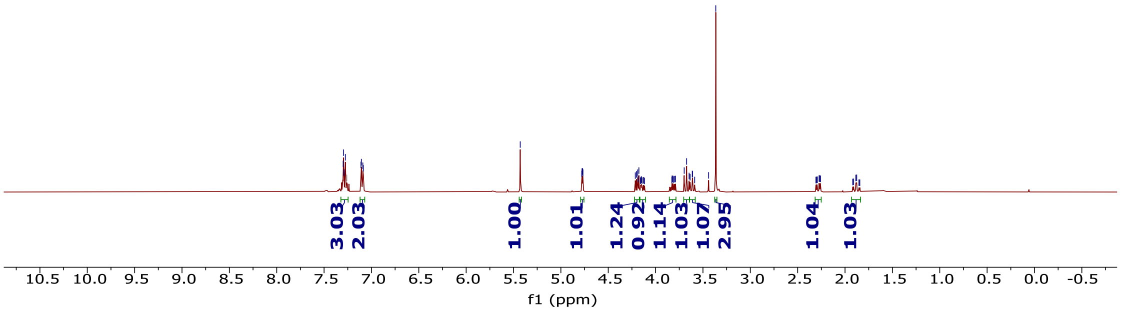

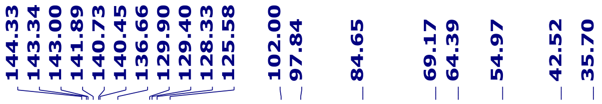

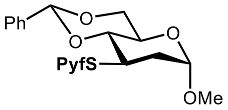

S13 - ^{13}C NMR (100 MHz, $CDCl_3$)

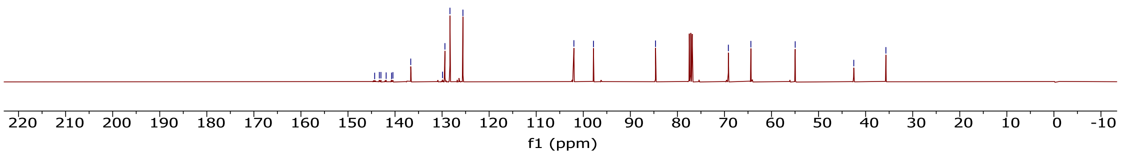

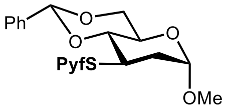

S13 - ^{19}F NMR (376 MHz, $CDCl_3$)

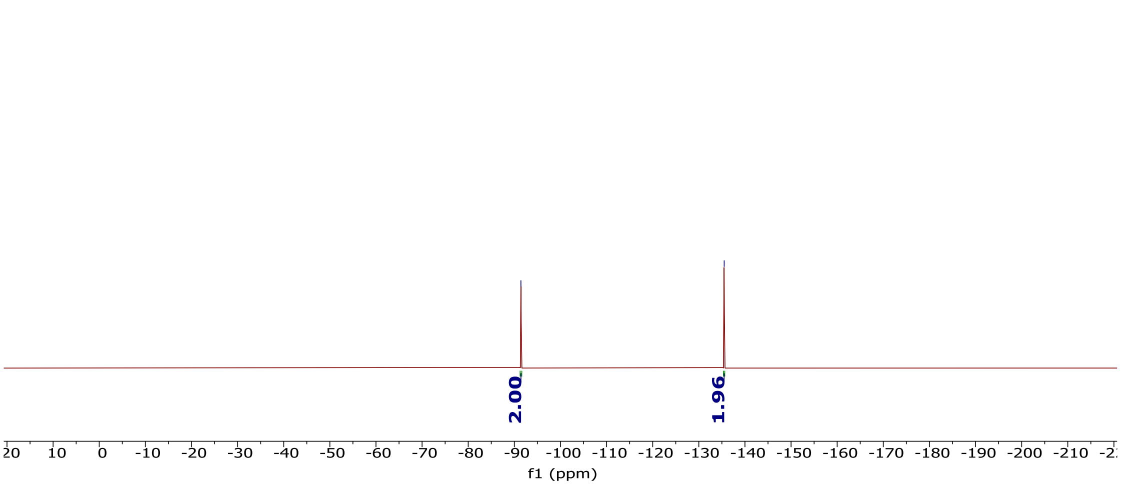

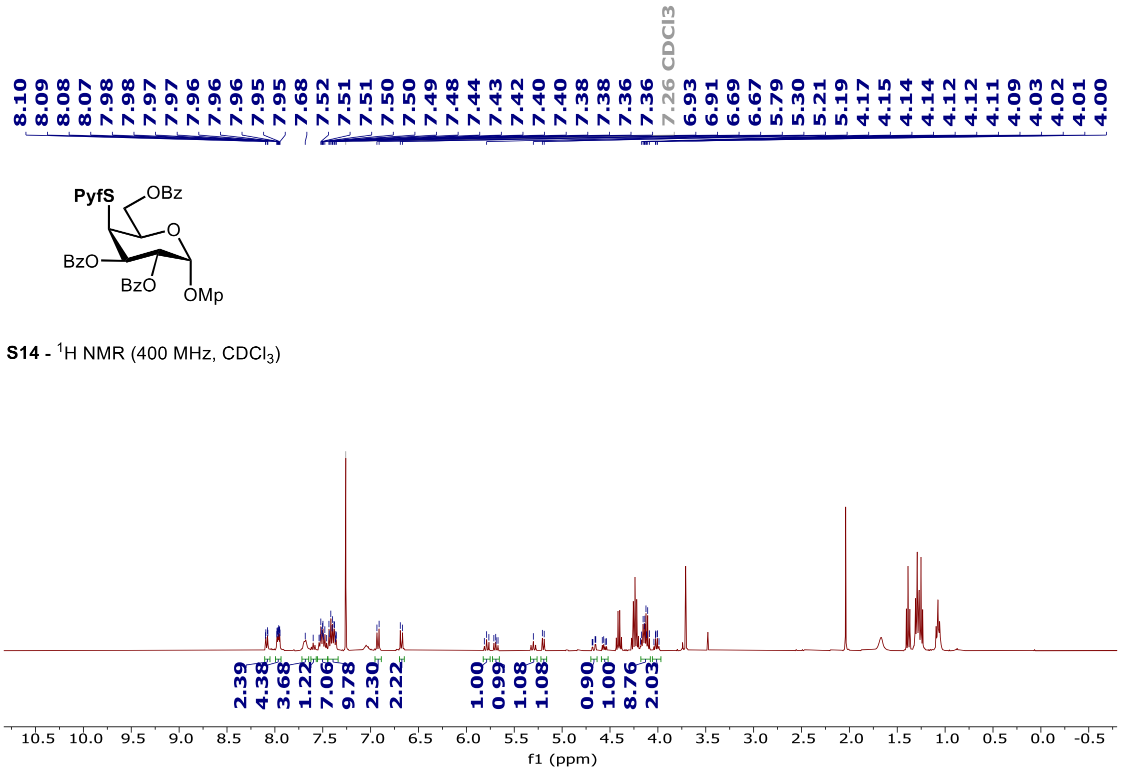

S14 - ^{1}H NMR (400 MHz, $CDCl_3$)

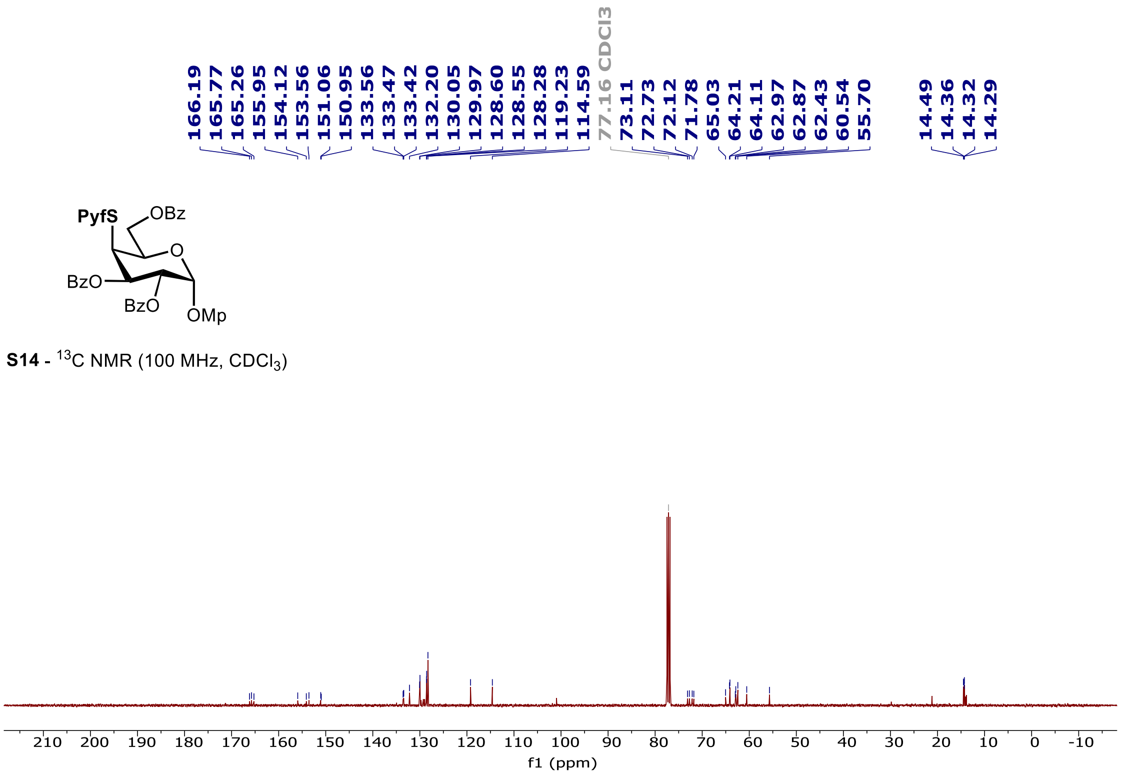

S14 - ^{13}C NMR (100 MHz, $CDCl_3$)

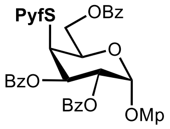

S14 - ^{19}F NMR (376 MHz, $CDCl_3$)

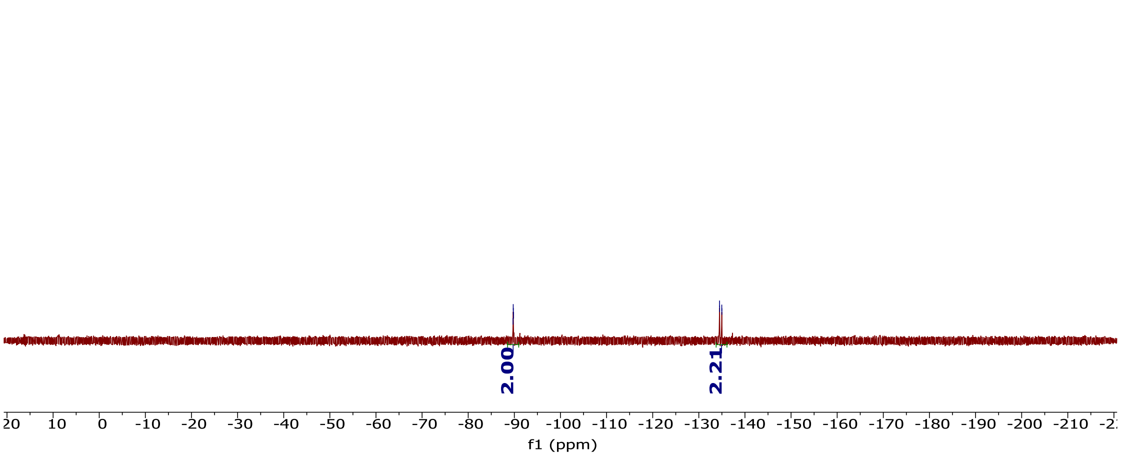

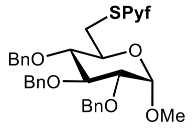

S15 - ^{1}H NMR (400 MHz, $CDCl_3$)

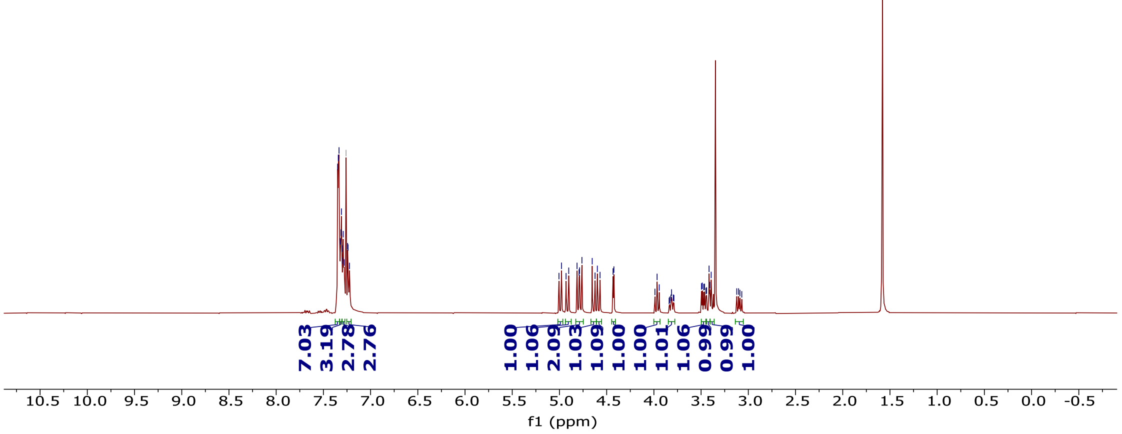

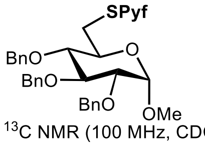

S15 - ^{13}C NMR (100 MHz, $CDCl_3$)

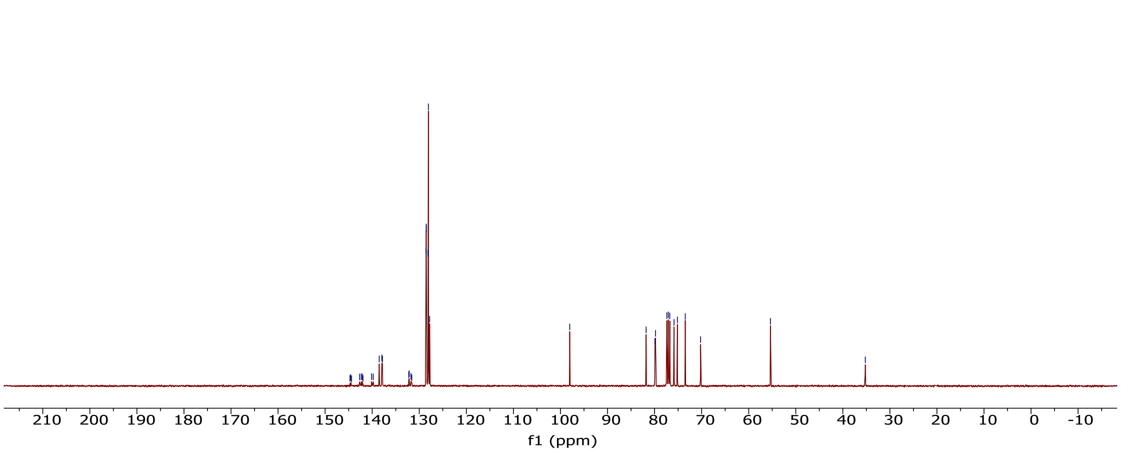

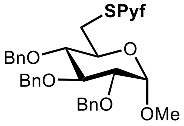

S15 - ^{19}F NMR (376 MHz, $CDCl_3$)

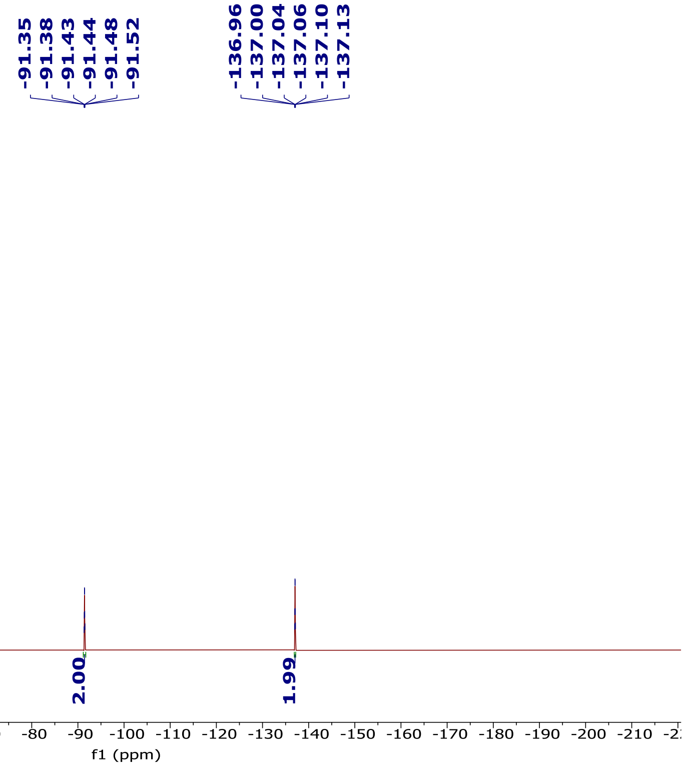

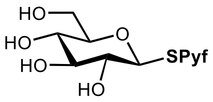

S16 - ^{1}H NMR (400 MHz, CD_3OD)

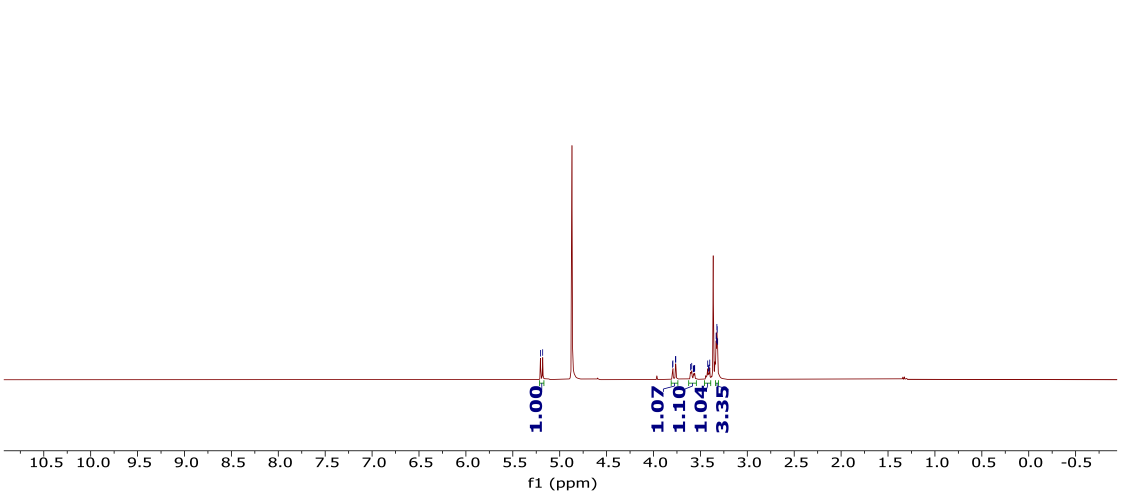

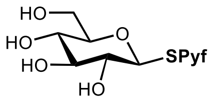

S16 - ^{13}C NMR (100 MHz, CD_3OD)

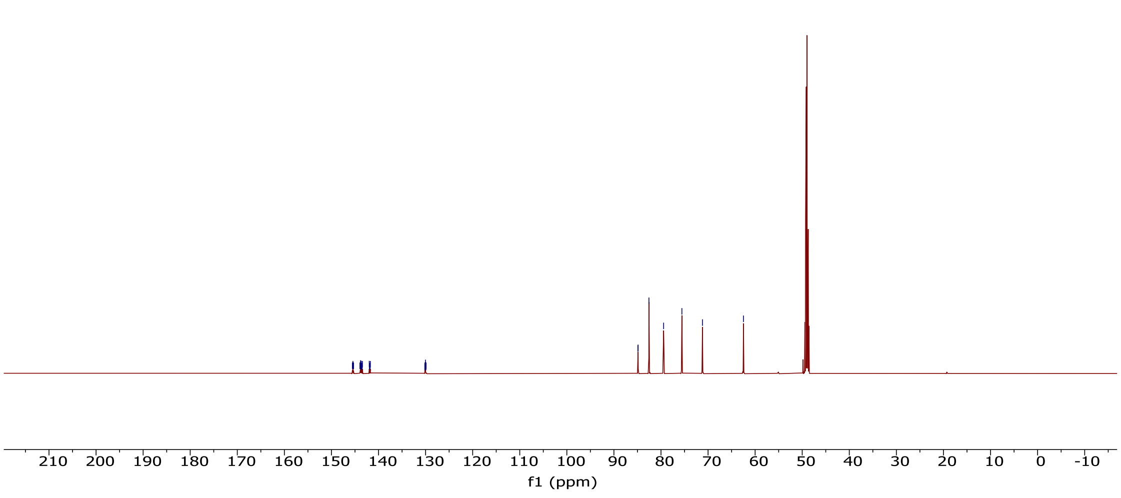

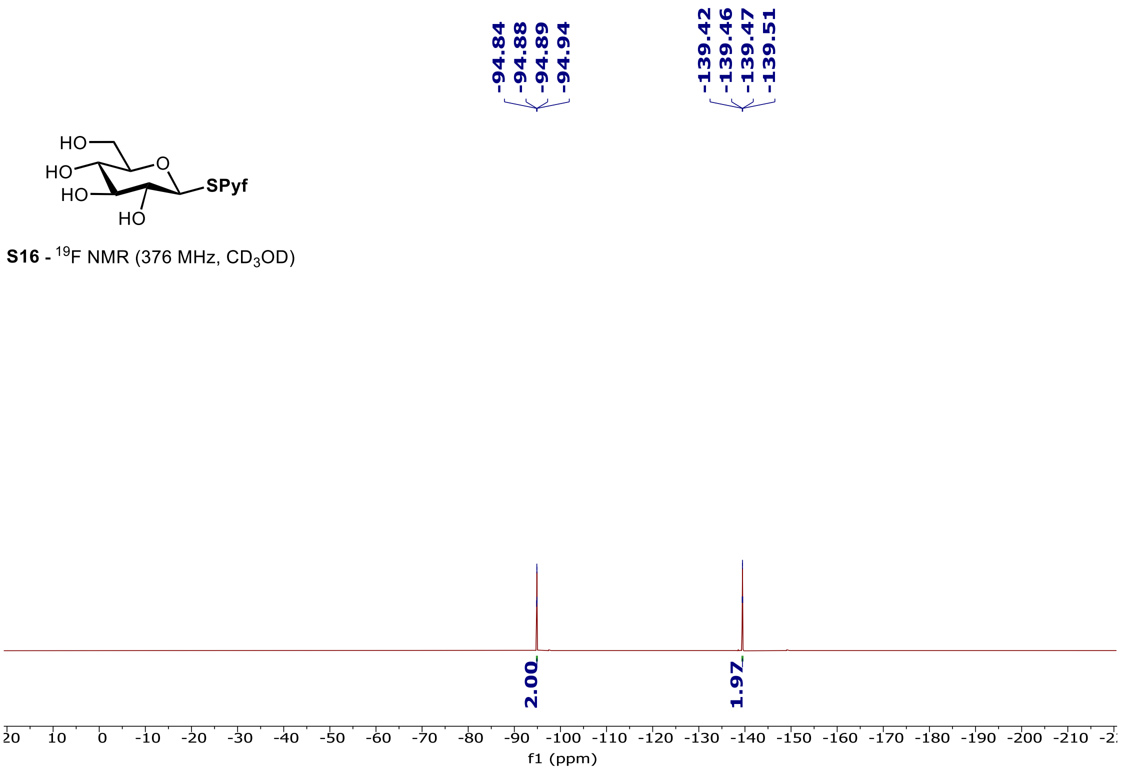

S16 - ^{19}F NMR (376 MHz, CD_3OD)

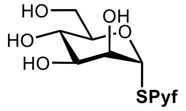

S17 - ^{1}H NMR (400 MHz, CD_3OD)

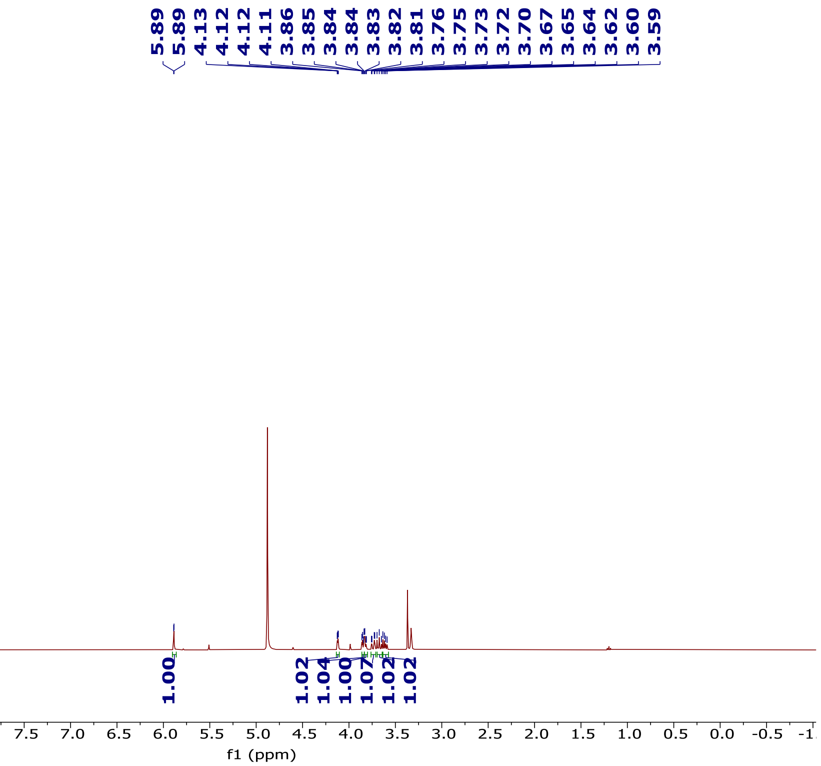

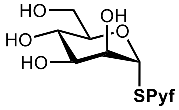

S17 - ^{13}C NMR (100 MHz, CD_3OD)

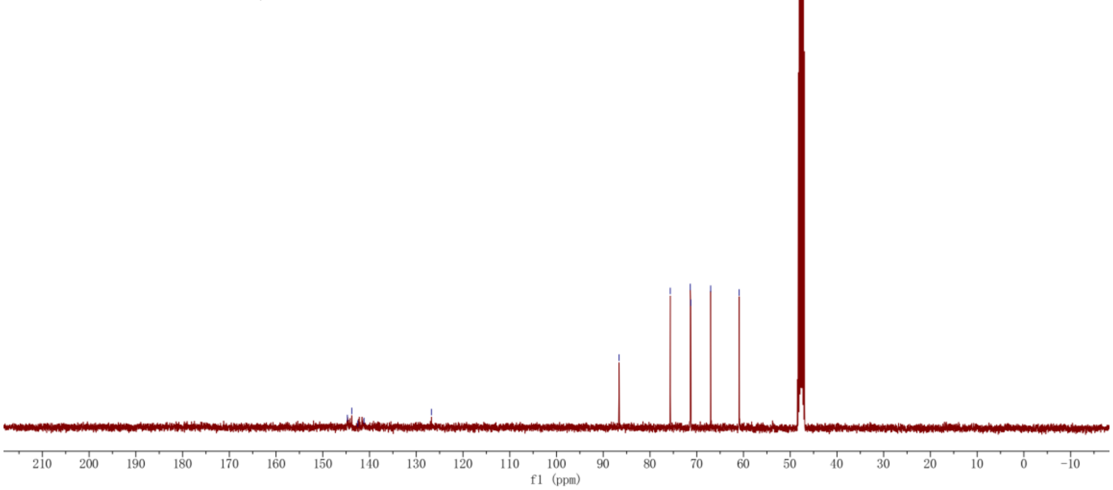

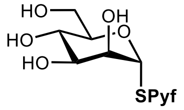

S17 - ^{19}F NMR (376 MHz, CD_3OD)

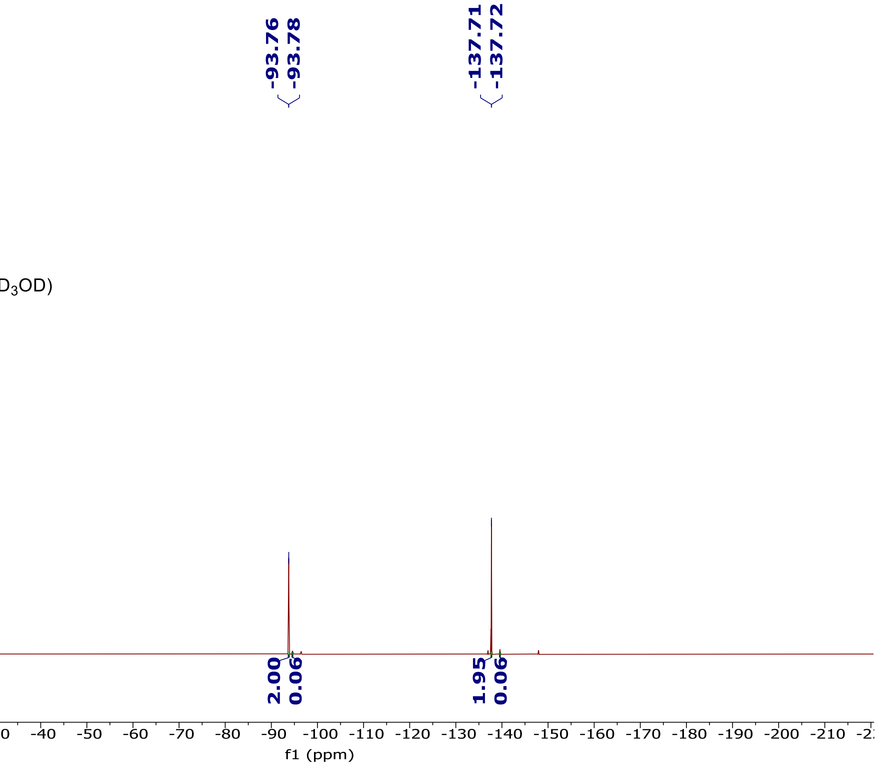

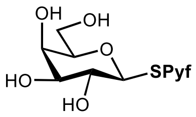

S18 - ^{1}H NMR (400 MHz, CD_3OD)

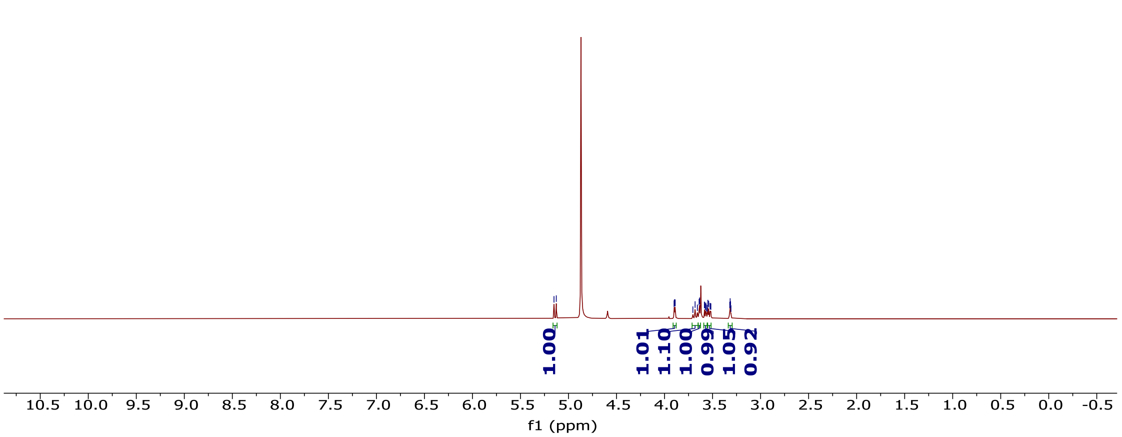

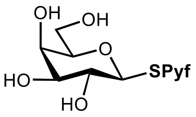

S18 - ^{13}C NMR (100 MHz, CD_3OD)

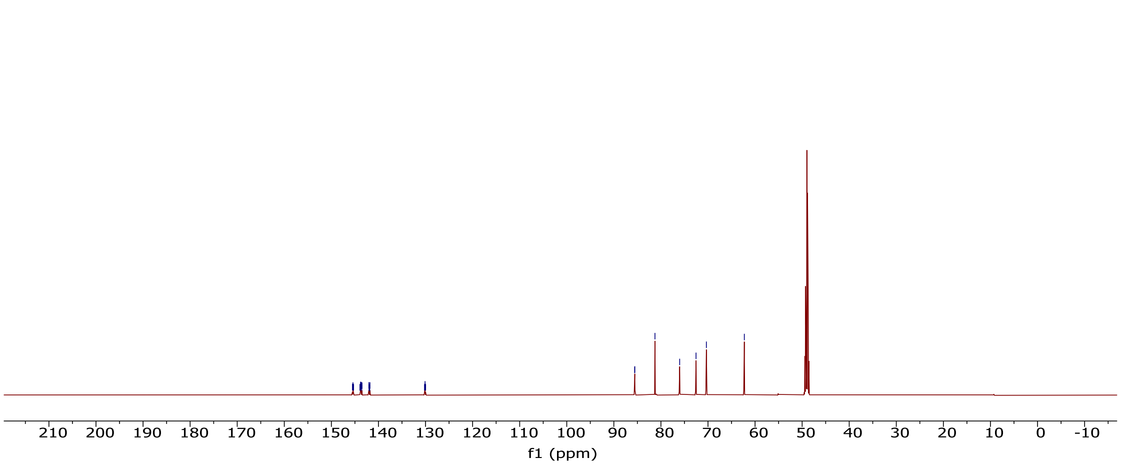

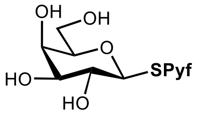

S18 - ^{19}F NMR (376 MHz, CD_3OD)

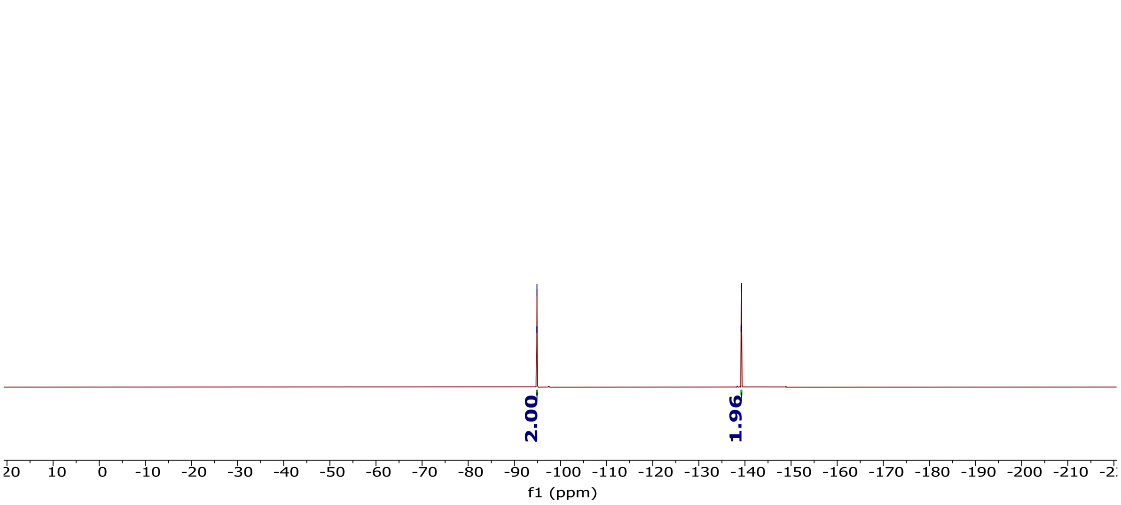

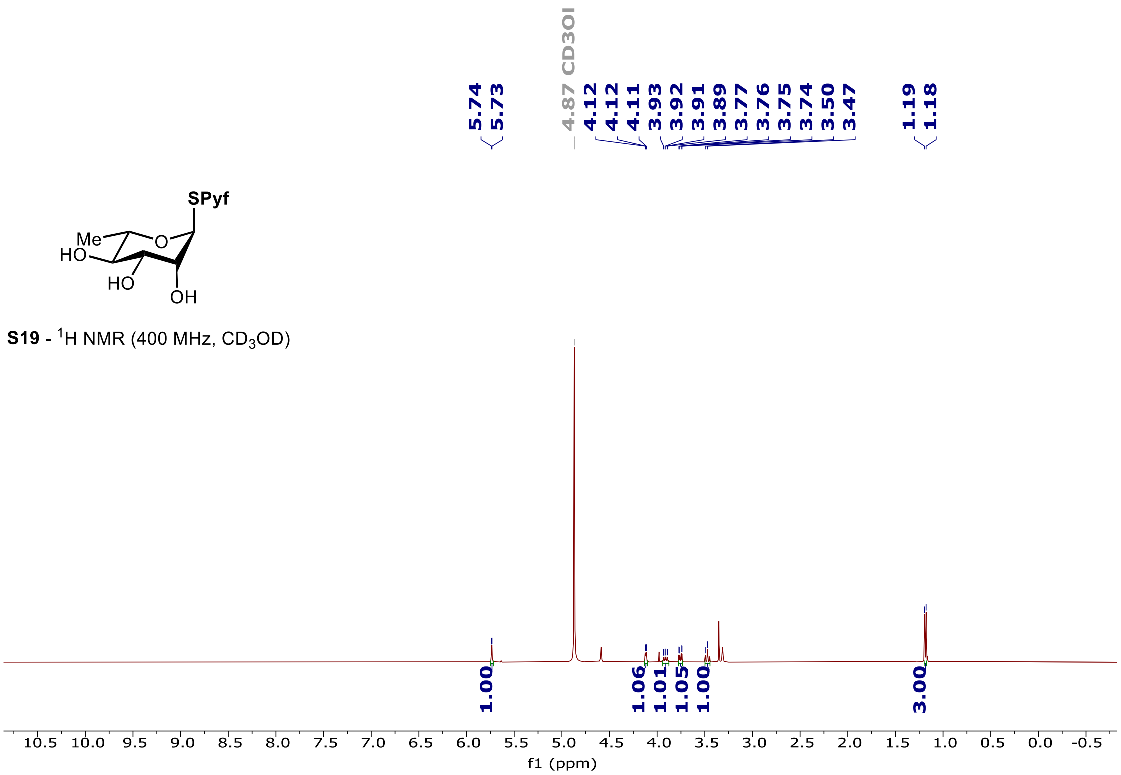

S19 - ^{1}H NMR (400 MHz, CD_3OD)

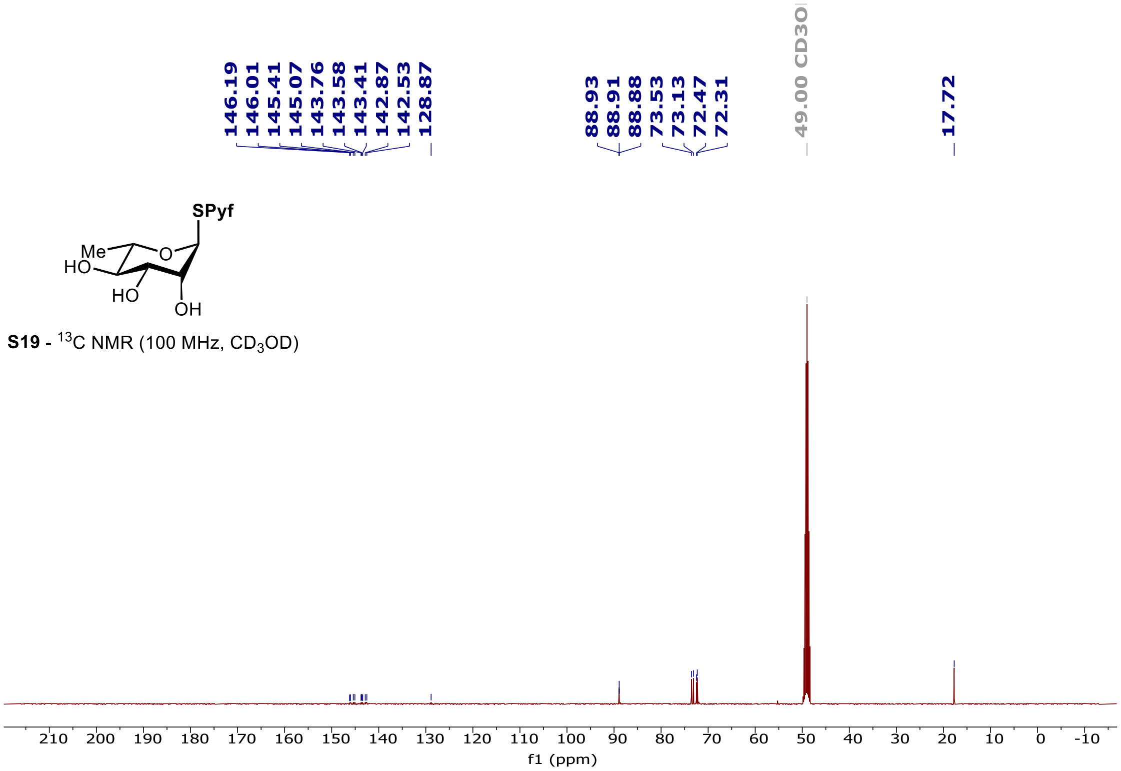

S19 - ^{13}C NMR (100 MHz, CD_3OD)

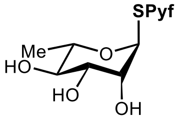

S19 - ^{19}F NMR (376 MHz, CD_3OD)

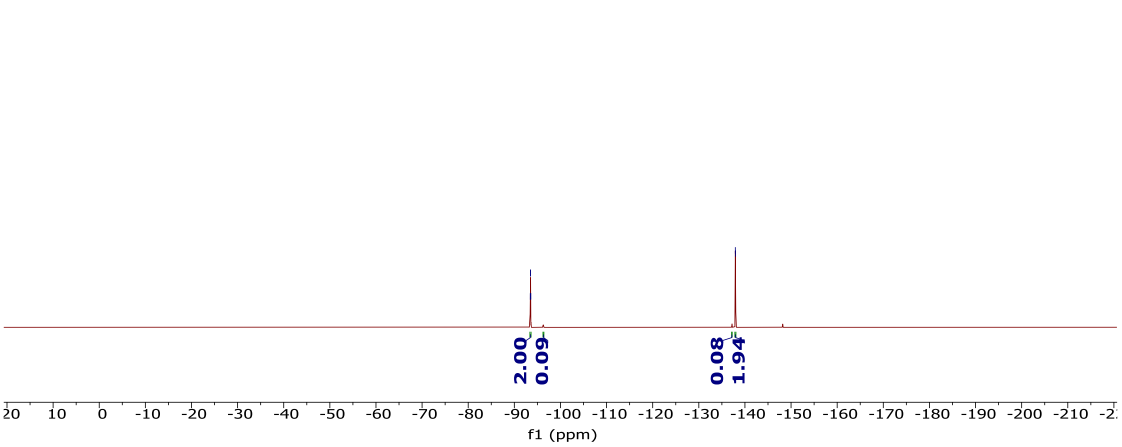

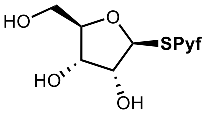

S20 - ^{1}H NMR (400 MHz, CD_3OD)

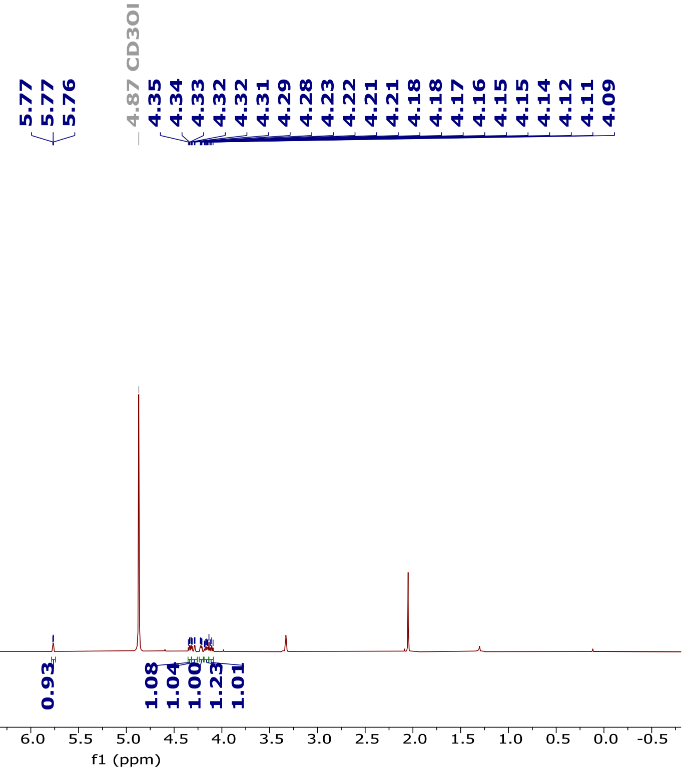

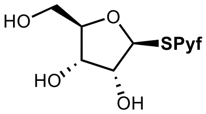

S20 - ^{13}C NMR (100 MHz, CD_3OD)

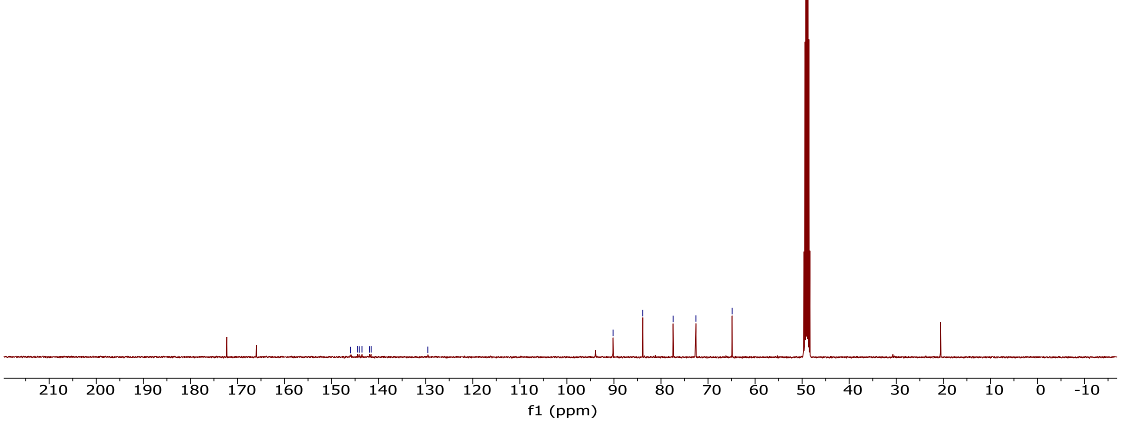

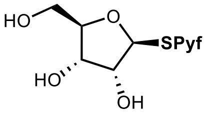

S20 - ^{19}F NMR (376 MHz, CD_3OD)

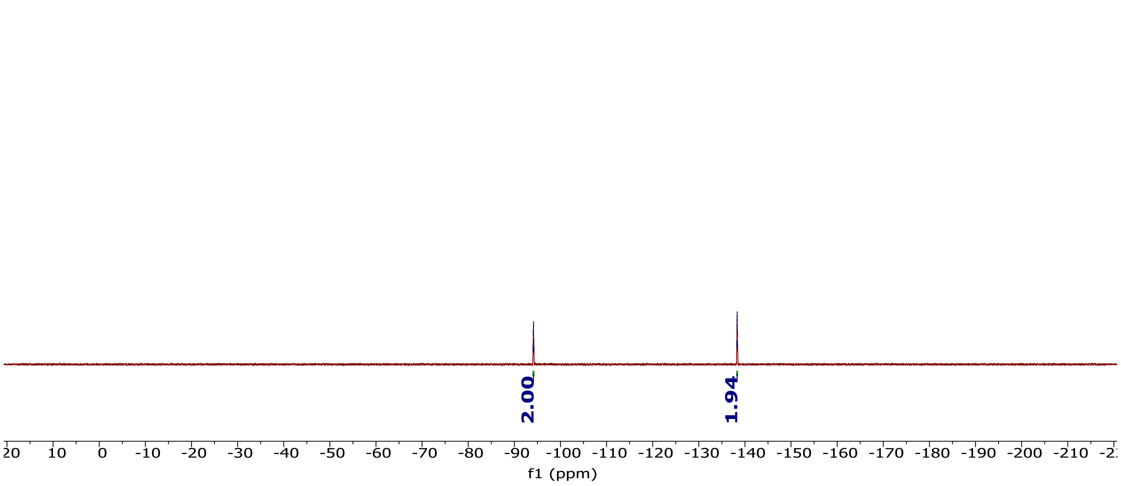

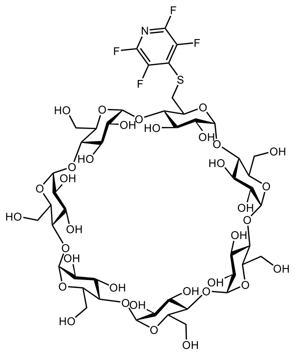

S21 - ^{1}H NMR (400 MHz, D_2O)

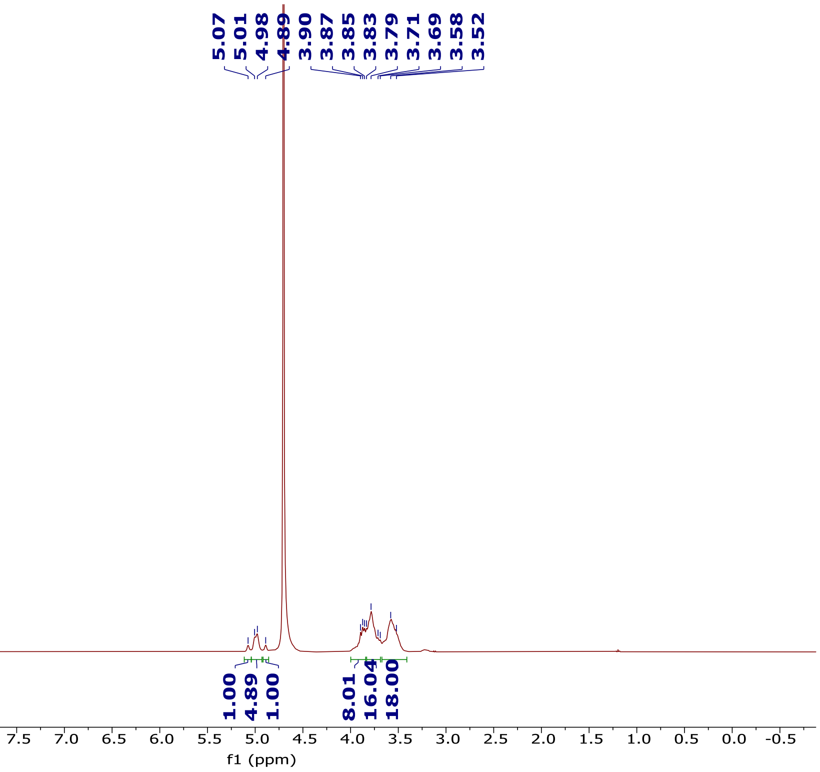

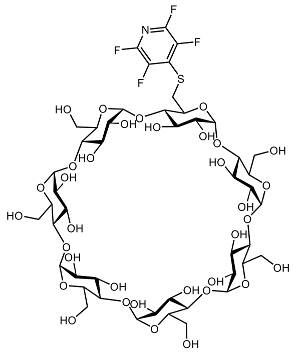

S21 - ^{13}C NMR (100 MHz, D_2O)

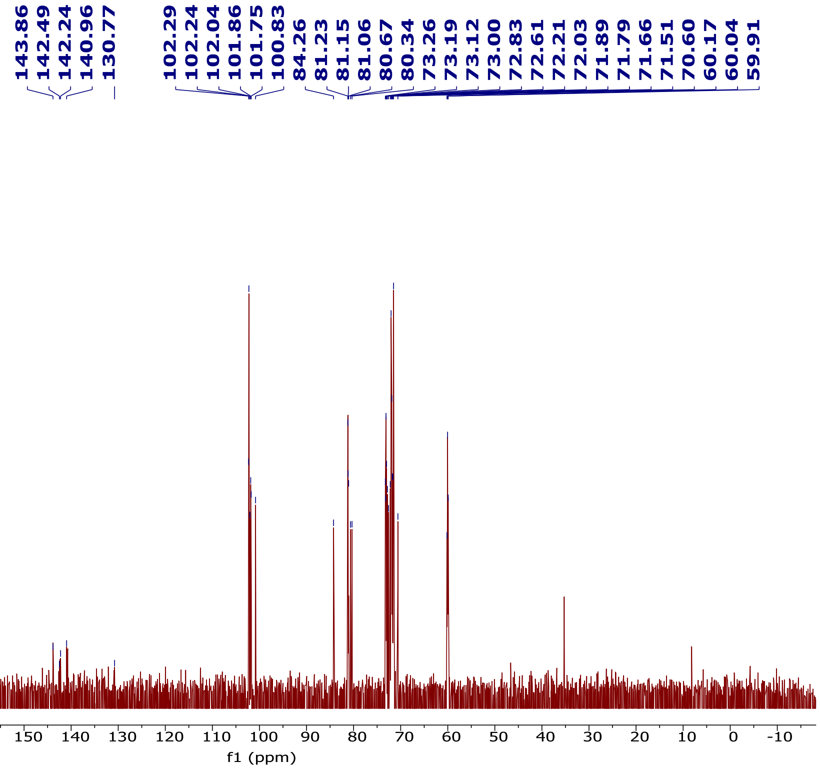

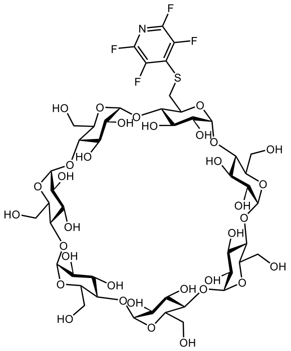

S21 - ^{19}F NMR (376 MHz, D_2O)

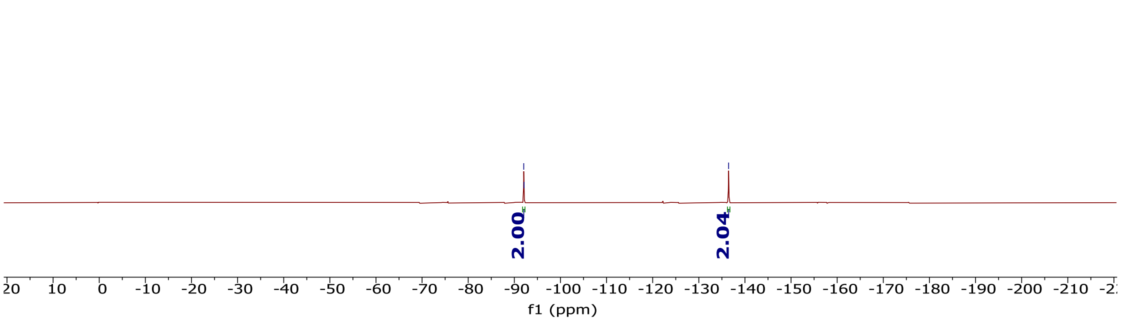

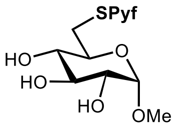

S22 - ^{1}H NMR (400 MHz, CD_3OD)

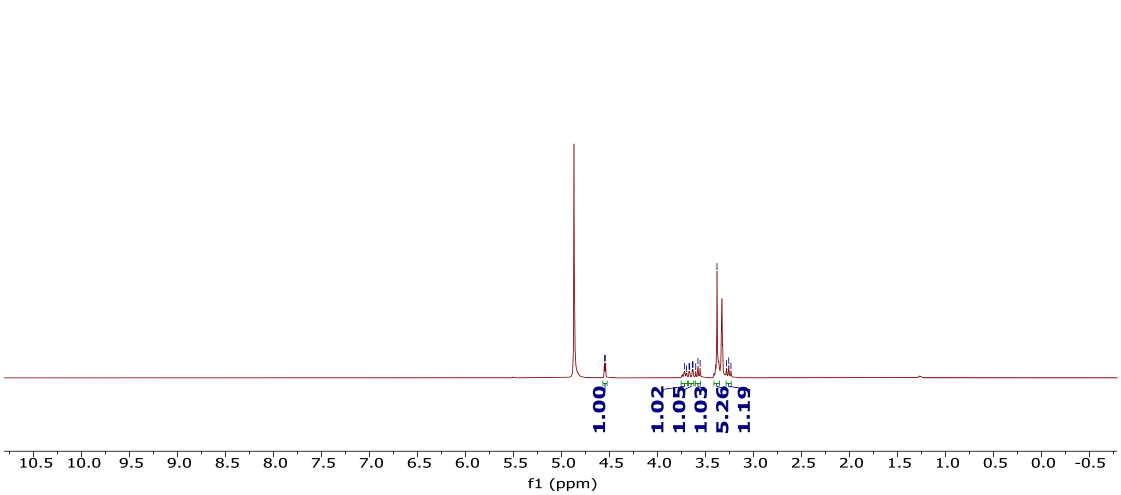

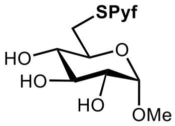

S22 - ^{13}C NMR (100 MHz, CD_3OD)

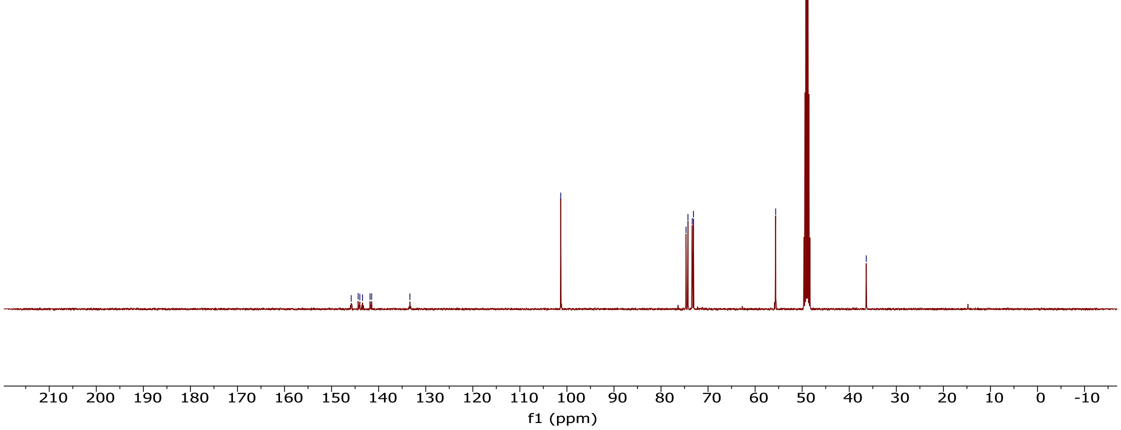

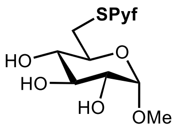

S22 - ^{19}F NMR (376 MHz, CD_3OD)

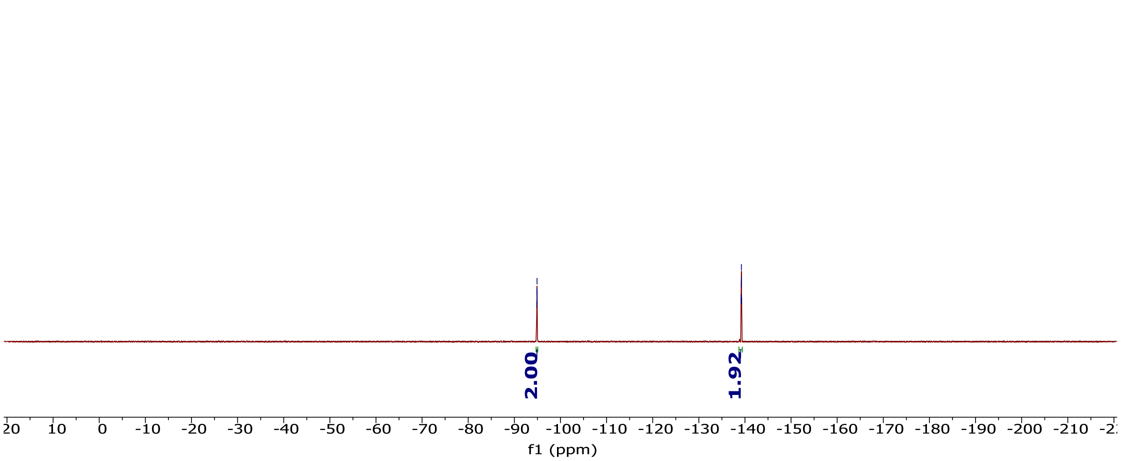

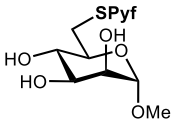

S23 - ^{1}H NMR (400 MHz, CD_3OD)

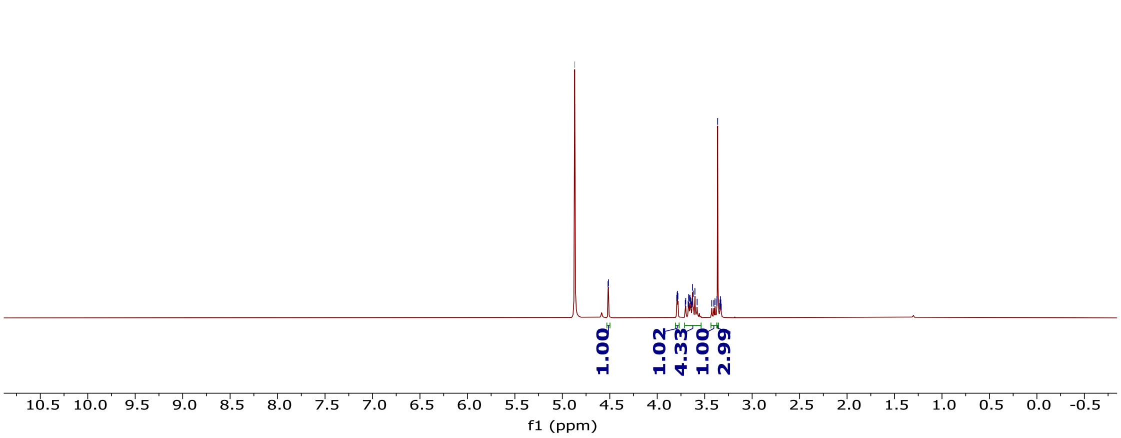

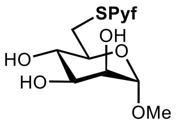

S23 - ^{13}C NMR (100 MHz, CD_3OD)

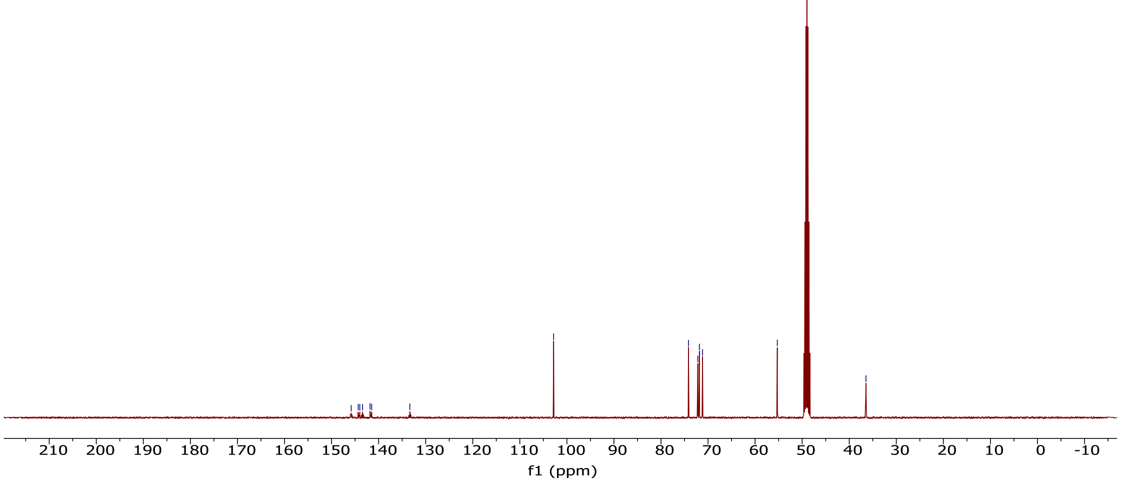

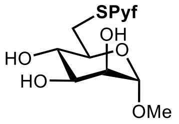

S23 - ^{19}F NMR (376 MHz, CD_3OD)

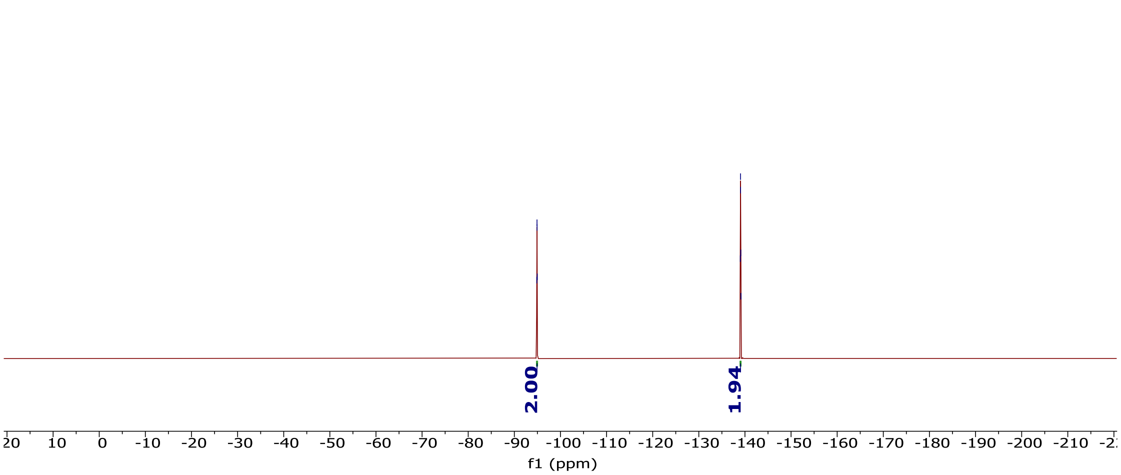

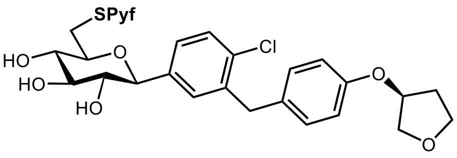

S24 - ^{1}H NMR (400 MHz, CD_3OD)

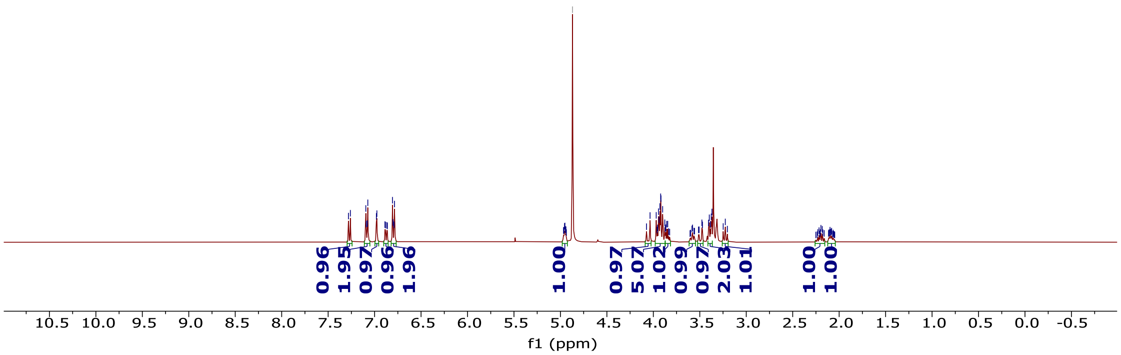

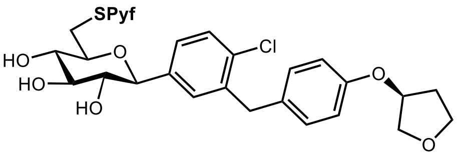

S24 - ^{13}C NMR (100 MHz, CD_3OD)

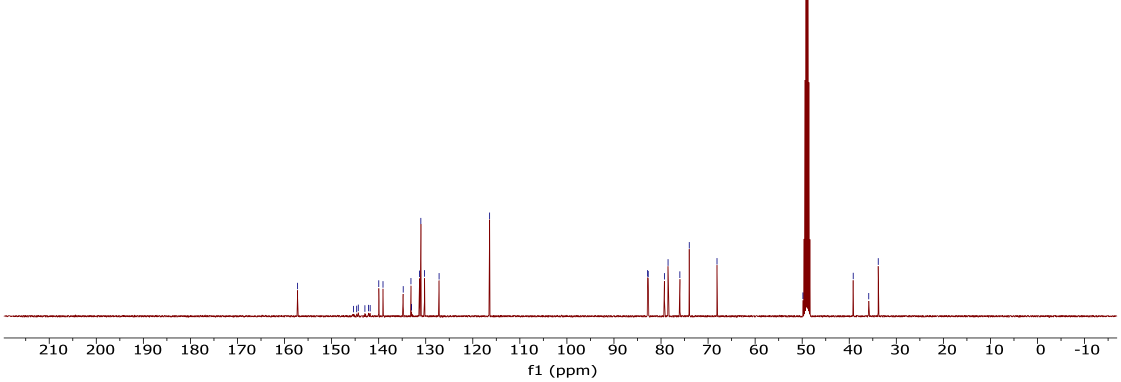

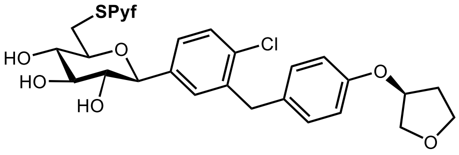

S24 - ^{19}F NMR (376 MHz, CD_3OD)

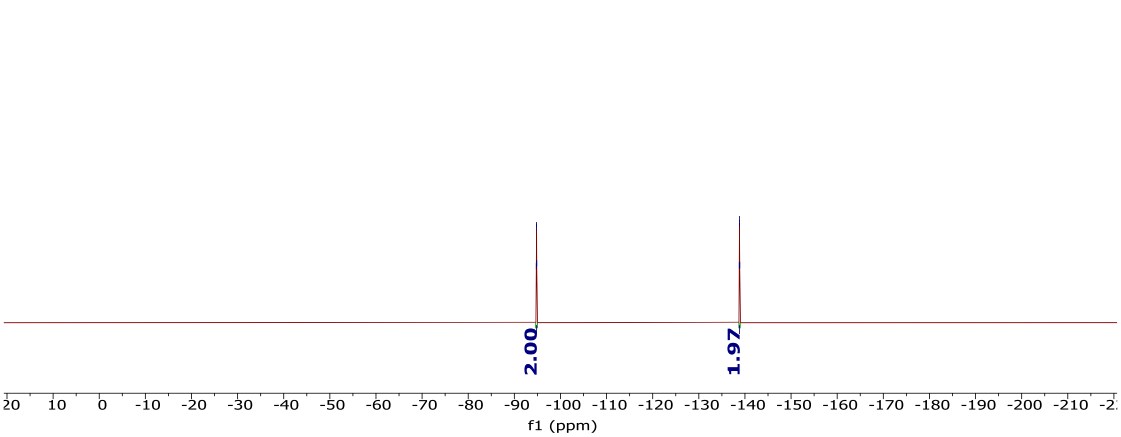

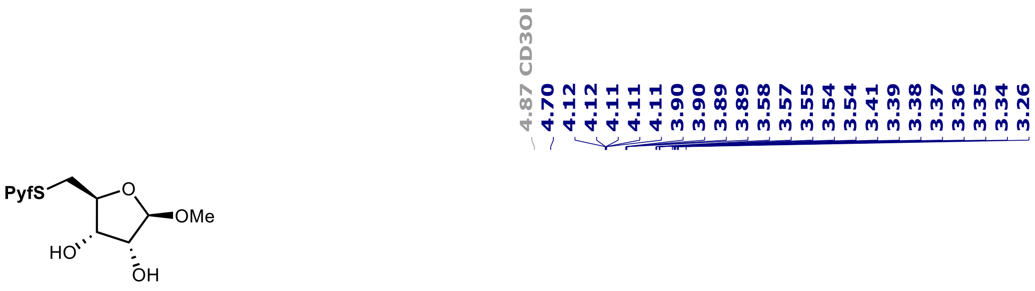

S25 - ^{1}H NMR (400 MHz, CD_3OD)

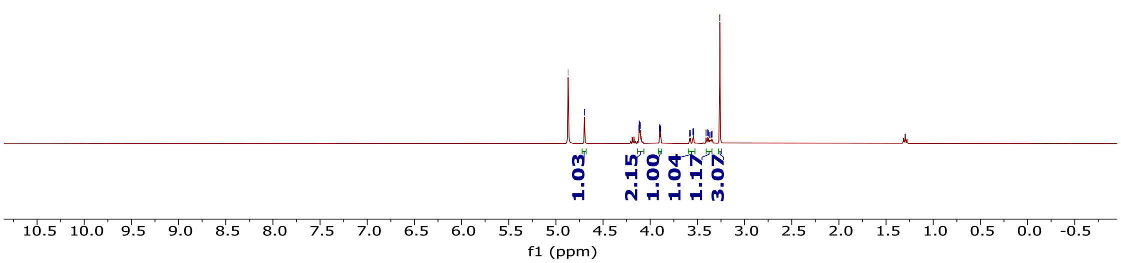

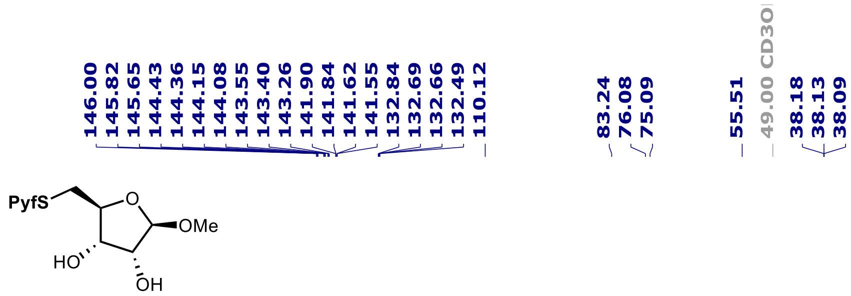

S25 - ^{13}C NMR (100 MHz, CD_3OD)

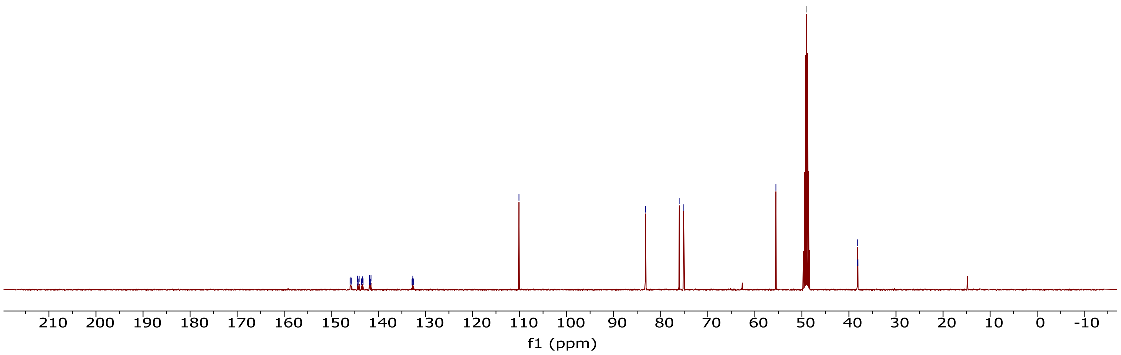

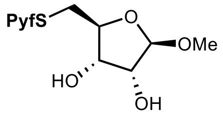

S25 - ^{19}F NMR (376 MHz, CD_3OD)

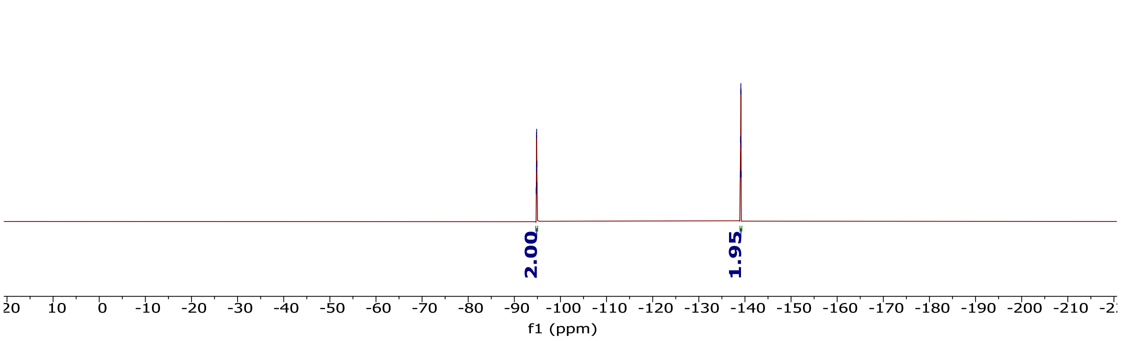

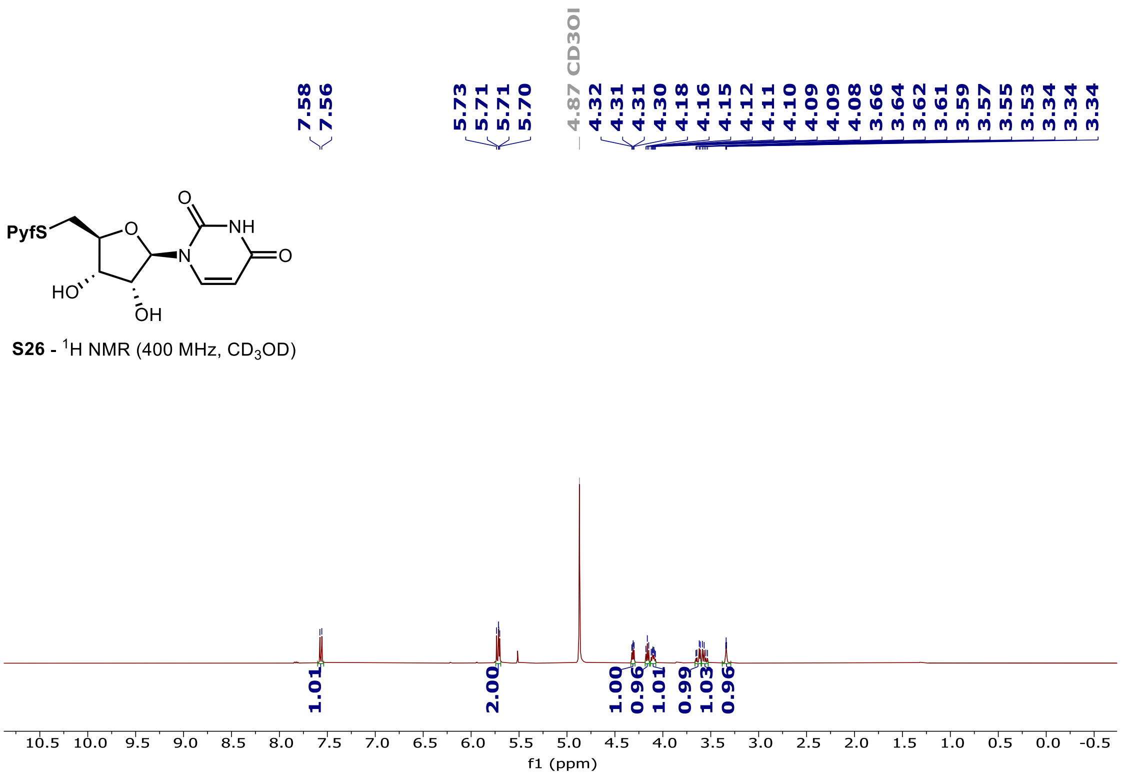

S26 - ^{1}H NMR (400 MHz, CD_3OD)

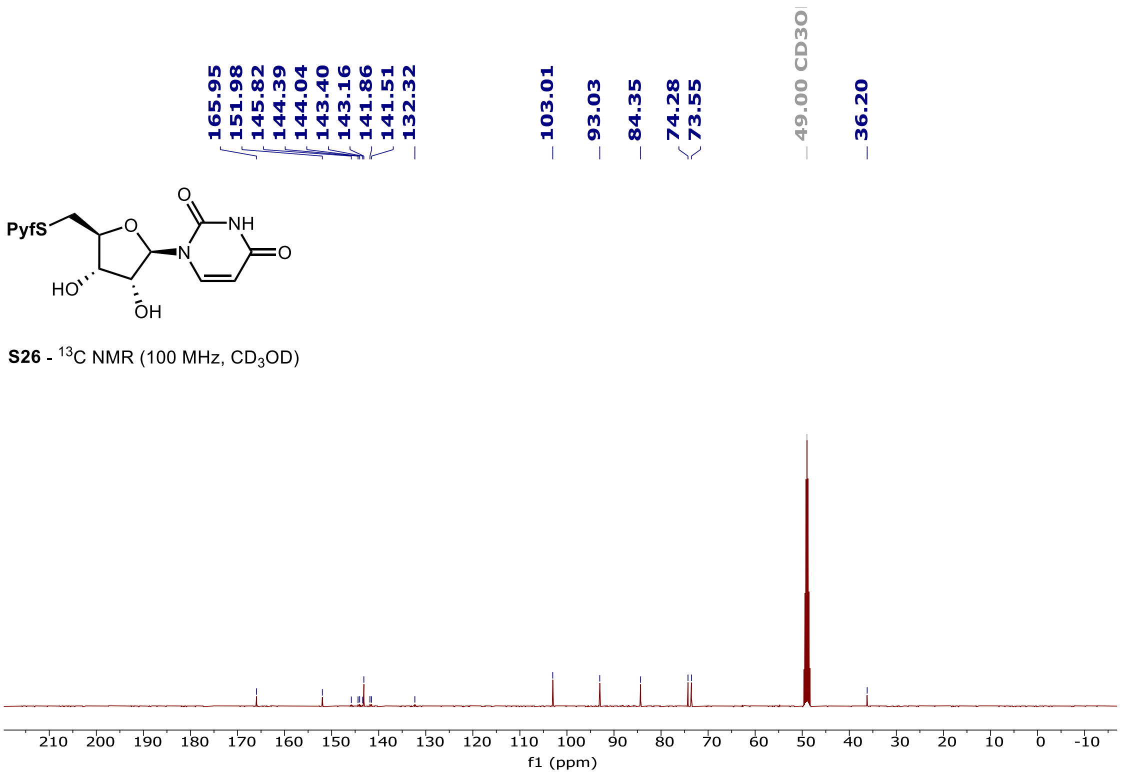

S26 - ^{13}C NMR (100 MHz, CD_3OD)

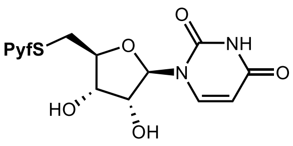

S26 - ^{19}F NMR (376 MHz, CD_3OD)

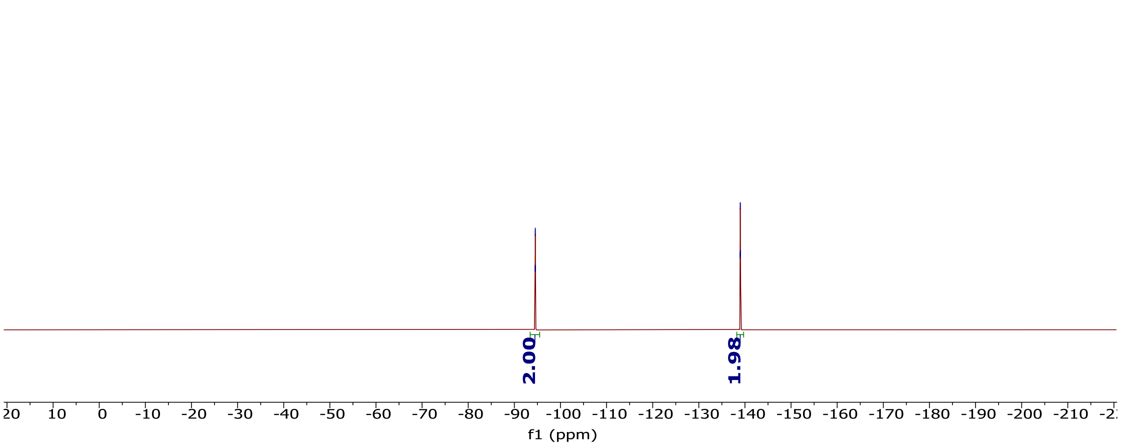

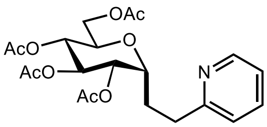

2 - ^{1}H NMR (400 MHz, $CDCl_3$)

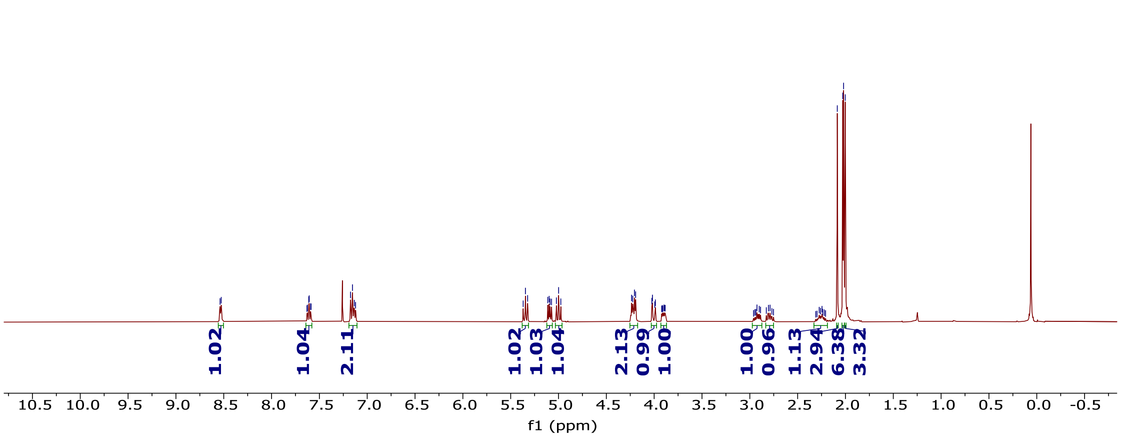

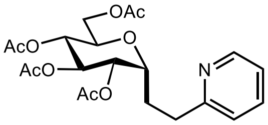

2 - ^{13}C NMR (100 MHz, $CDCl_3$)

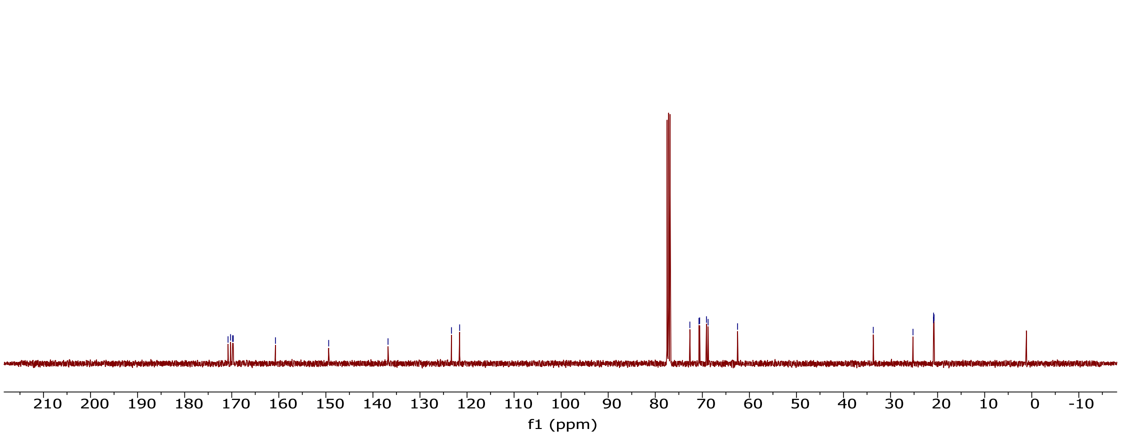

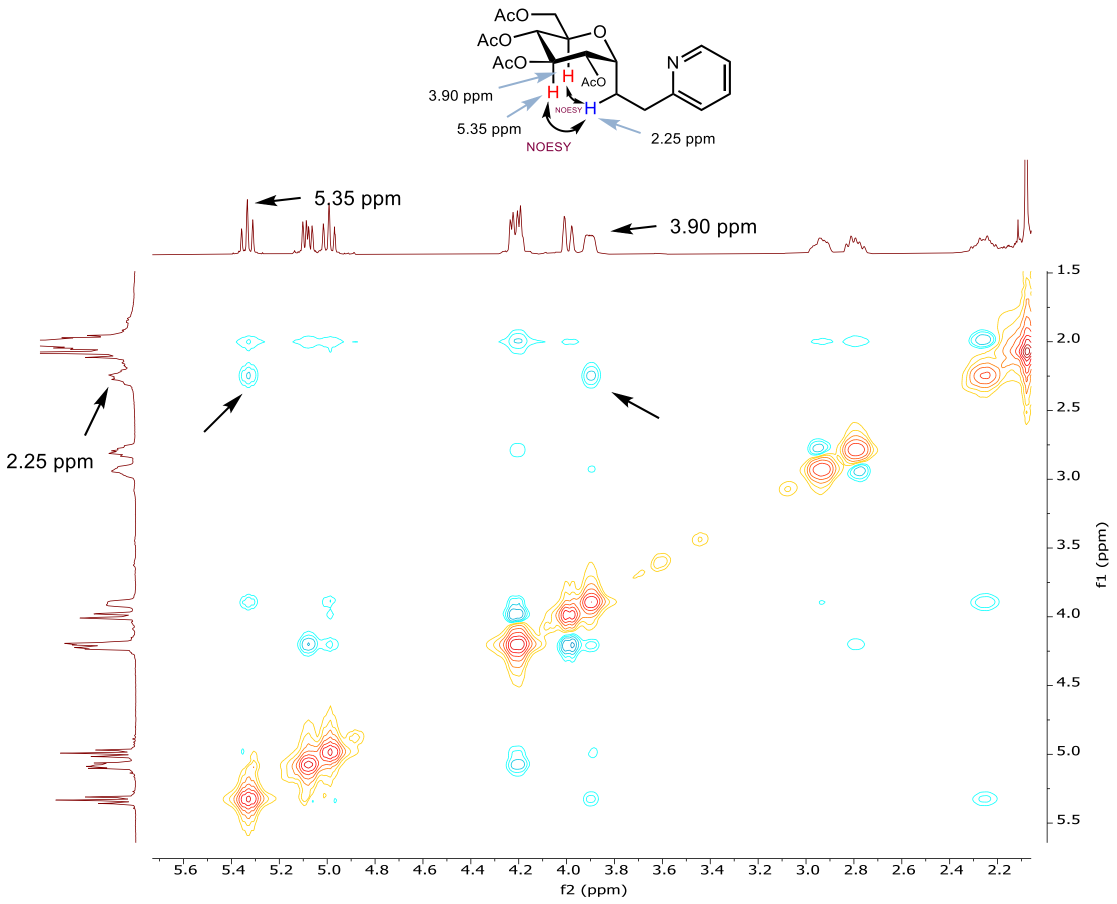
AcO
AcO
AcO
O
AcO
N
H
H
H
3.90 ppm
5.35 ppm
2.25 ppm
NOESY
NOESY
5.35 ppm
3.90 ppm
2.25 ppm
f2 (ppm)
f1 (ppm)

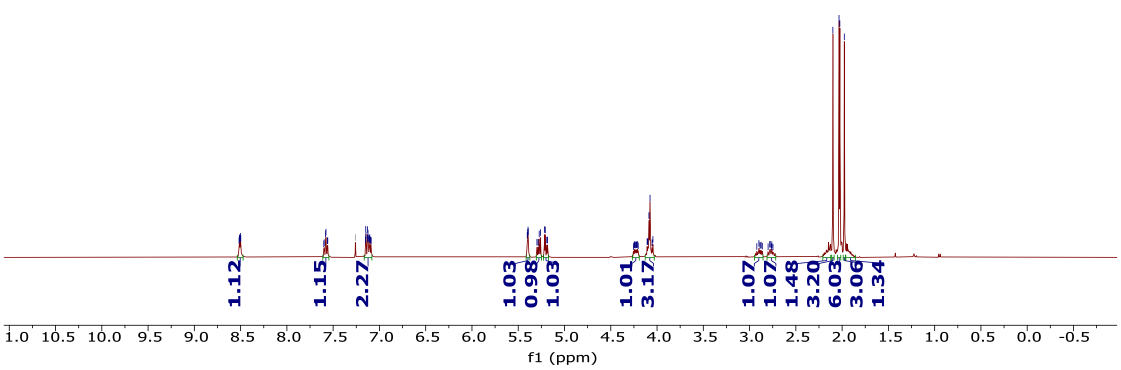

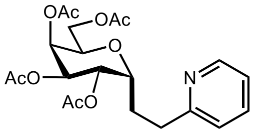

7 - ^{1}H NMR (400 MHz, $CDCl_3$)

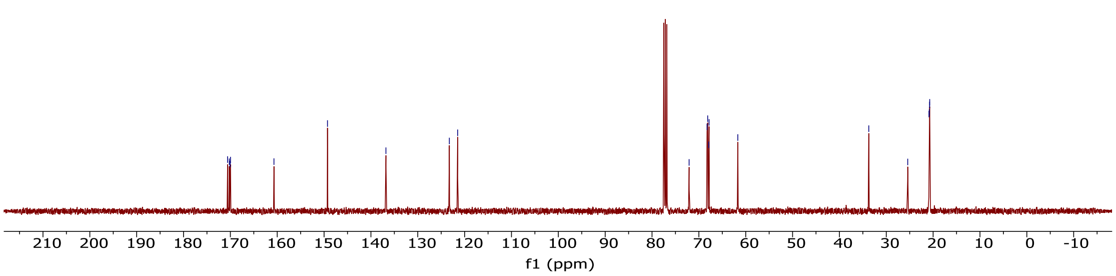

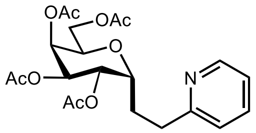

7 - ^{13}C NMR (100 MHz, $CDCl_3$)

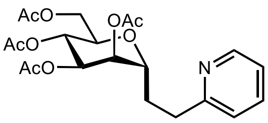

8 - ^{1}H NMR (400 MHz, $CDCl_3$)

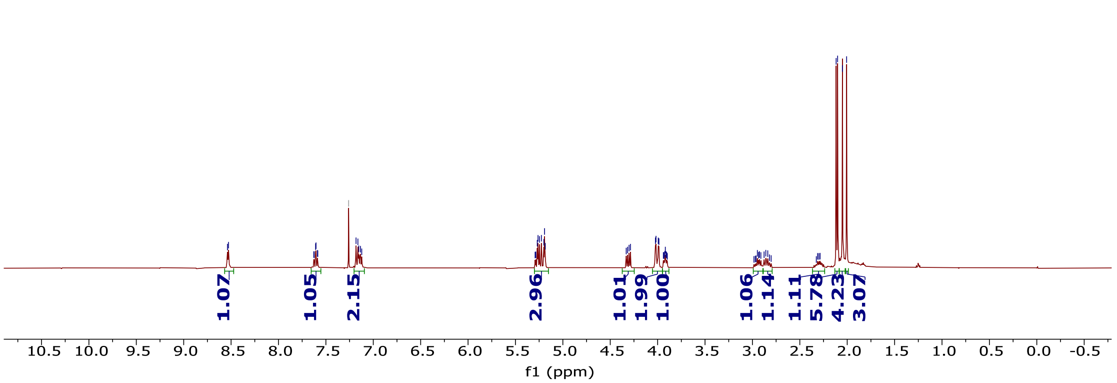

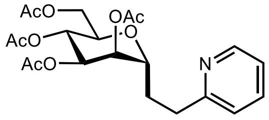

8 - ^{13}C NMR (100 MHz, $CDCl_3$)

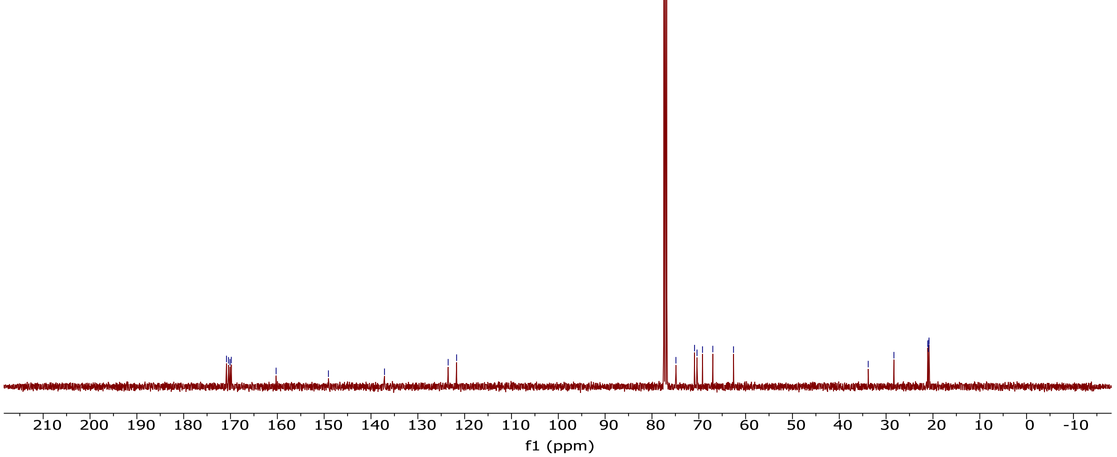

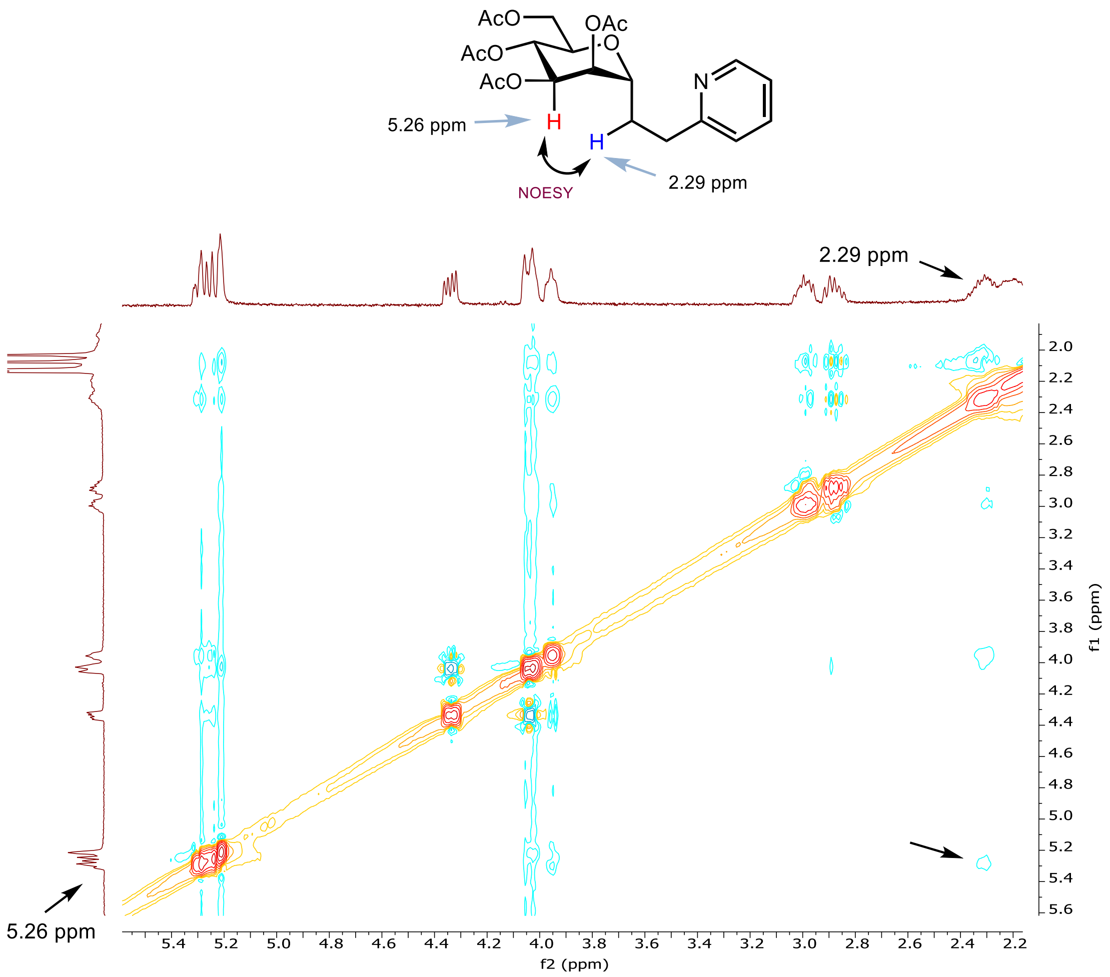
AcO
OAc
O
AcO
AcO
N
5.26 ppm
H
H
NOESY
2.29 ppm
2.29 ppm
5.26 ppm
f1 (ppm)
f2 (ppm)

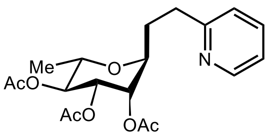

9 - ^{1}H NMR (400 MHz, $CDCl_3$)

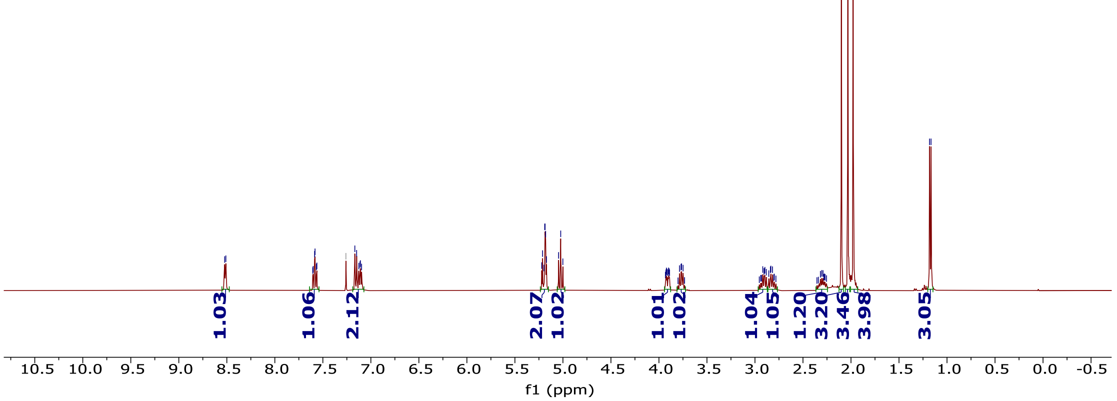

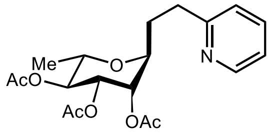

9 - ^{13}C NMR (100 MHz, $CDCl_3$)

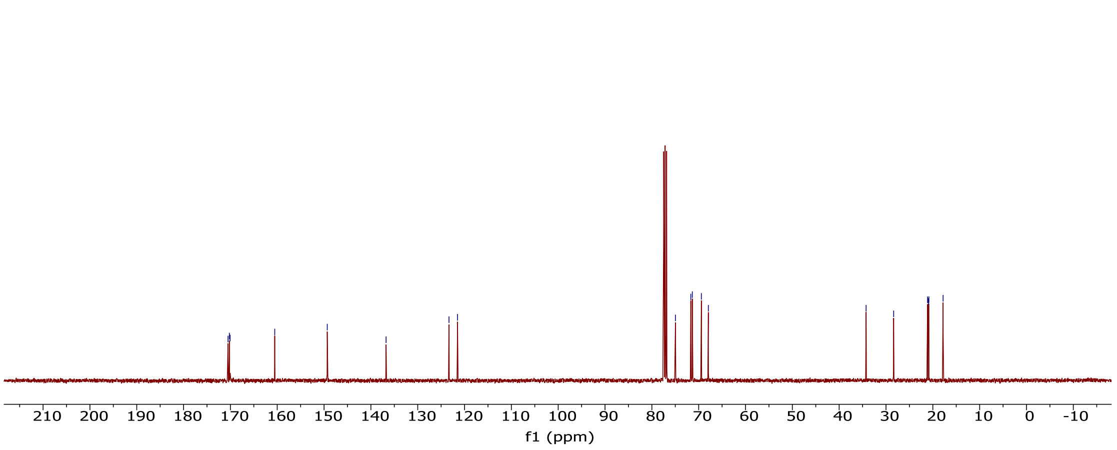

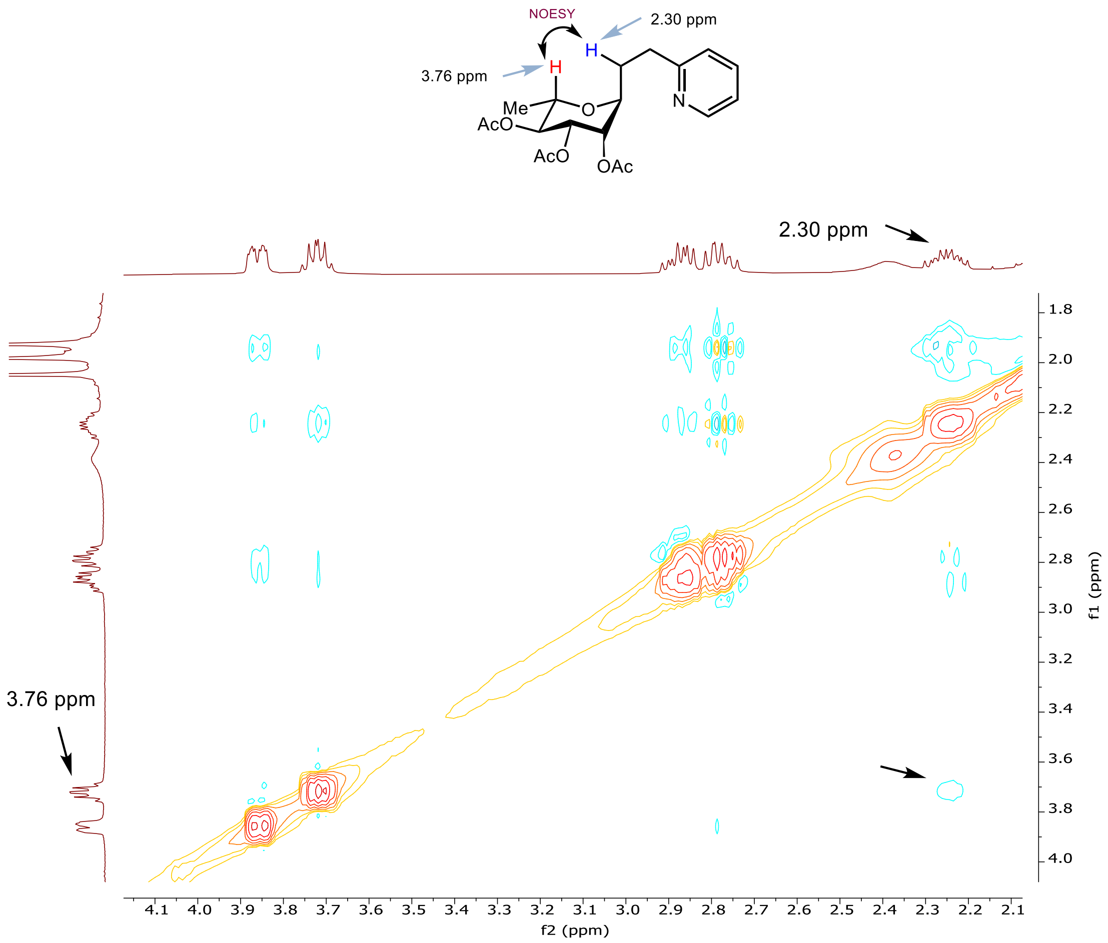
NOESY
2.30 ppm
3.76 ppm
Me
AcO
AcO
OAc
O
N
H
H
2.30 ppm
3.76 ppm
f2 (ppm)
f1 (ppm)

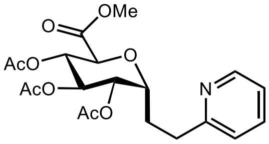

10 - ^{1}H NMR (400 MHz, $CDCl_3$)

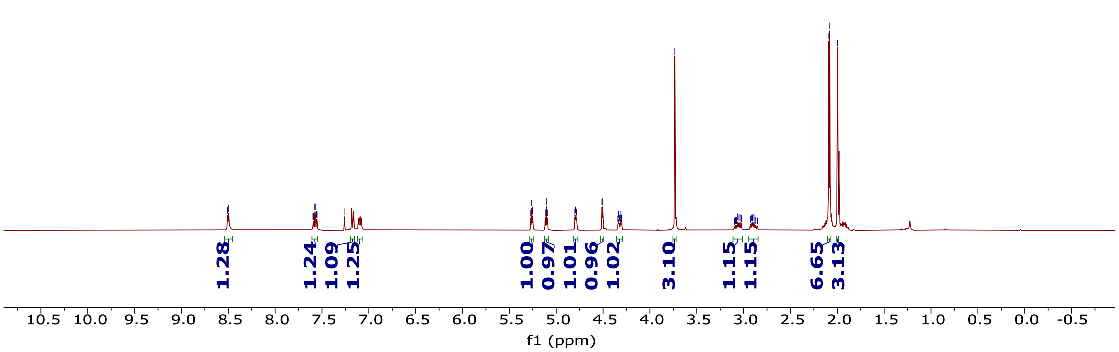

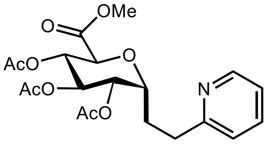

10 - ^{13}C NMR (100 MHz, $CDCl_3$)

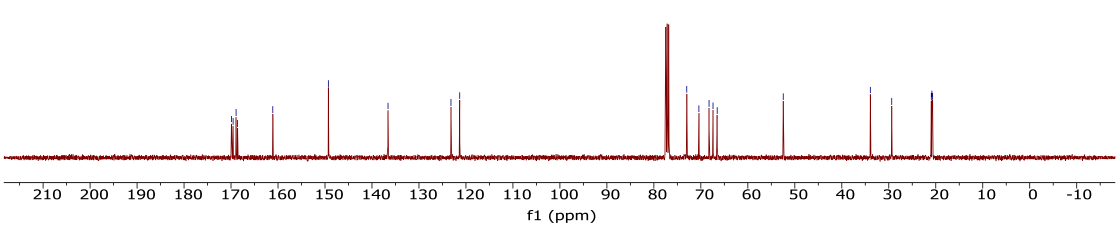

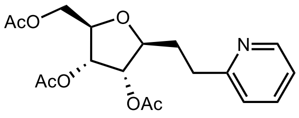

11 - ^{1}H NMR (400 MHz, $CDCl_3$)

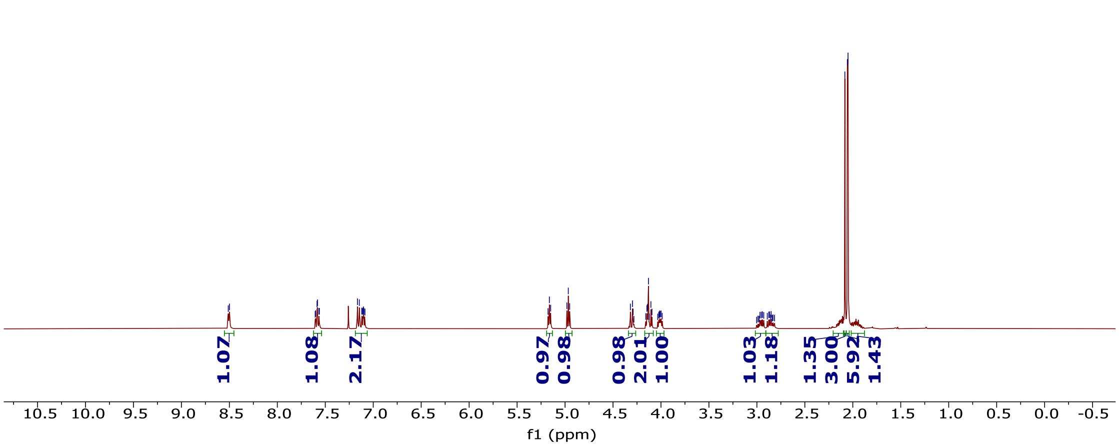

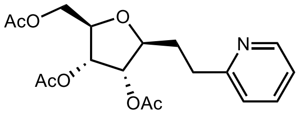

11 - ^{13}C NMR (100 MHz, $CDCl_3$)

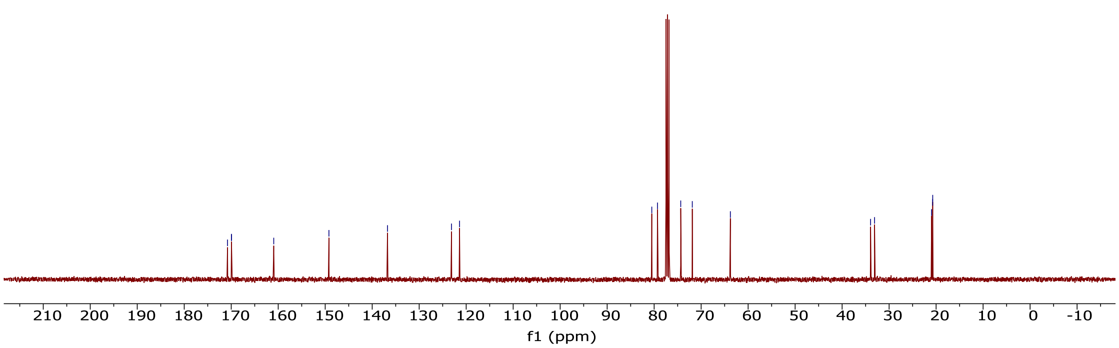

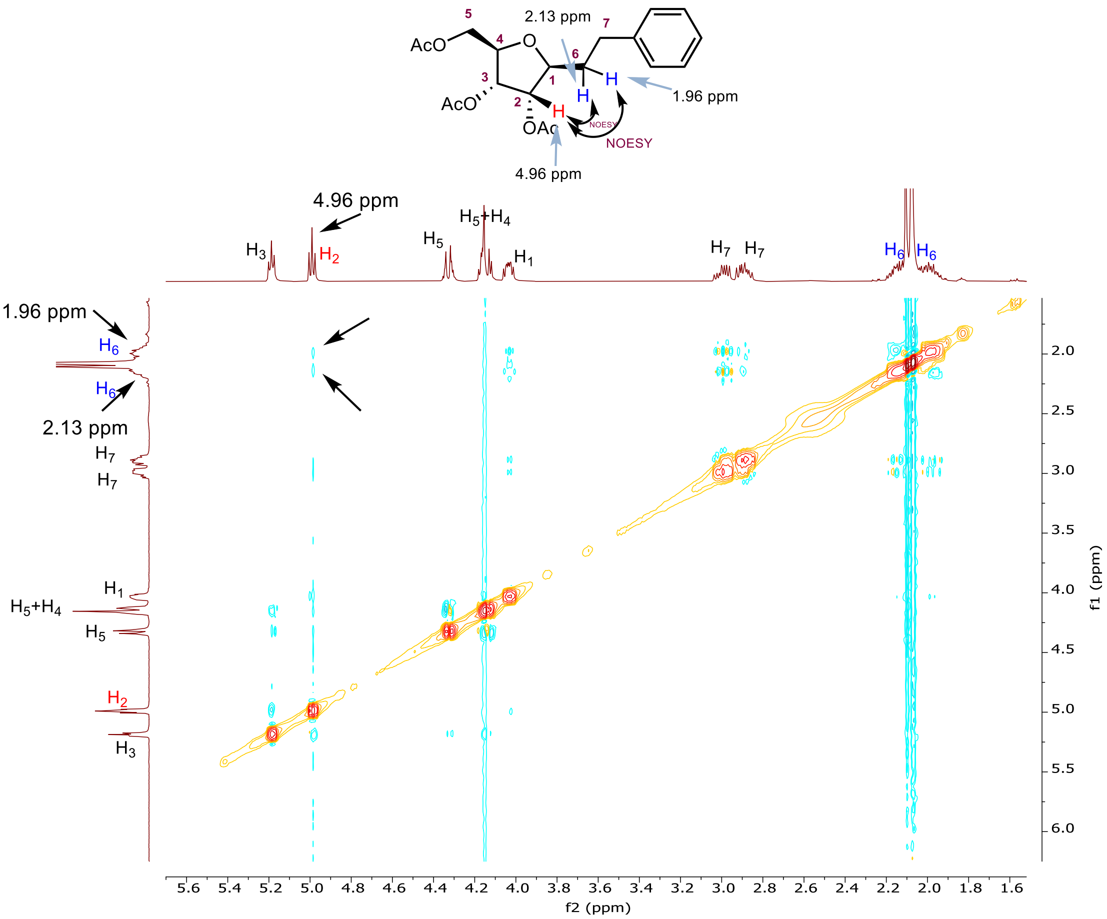

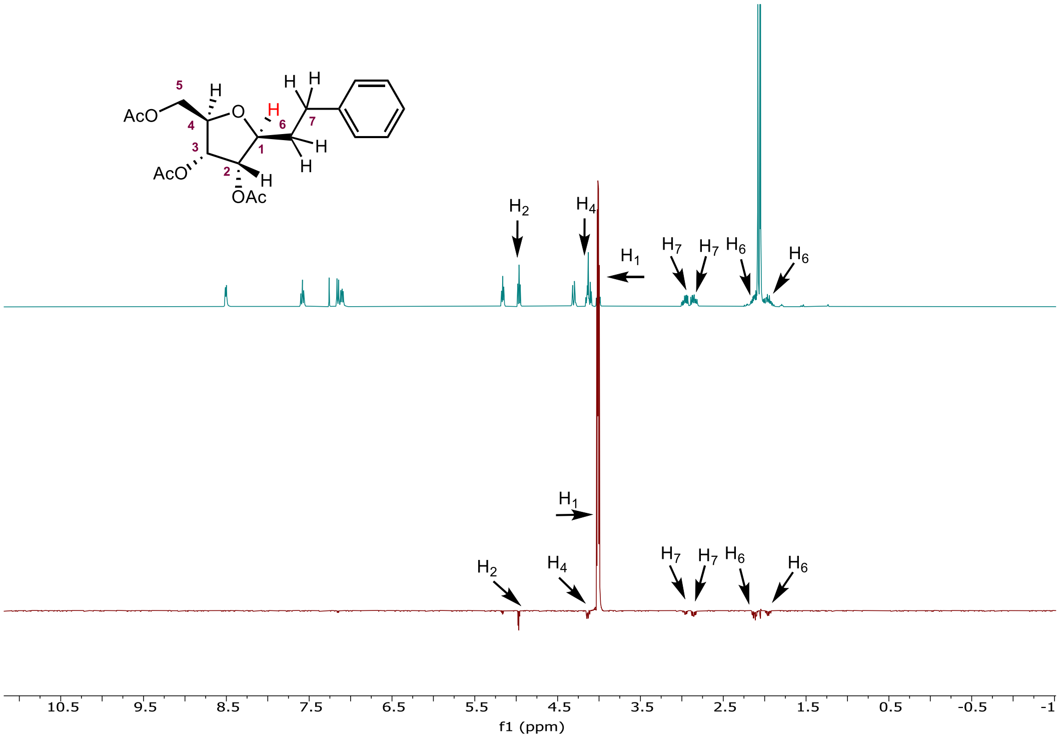

1D NOE NMR spectrum of **11**

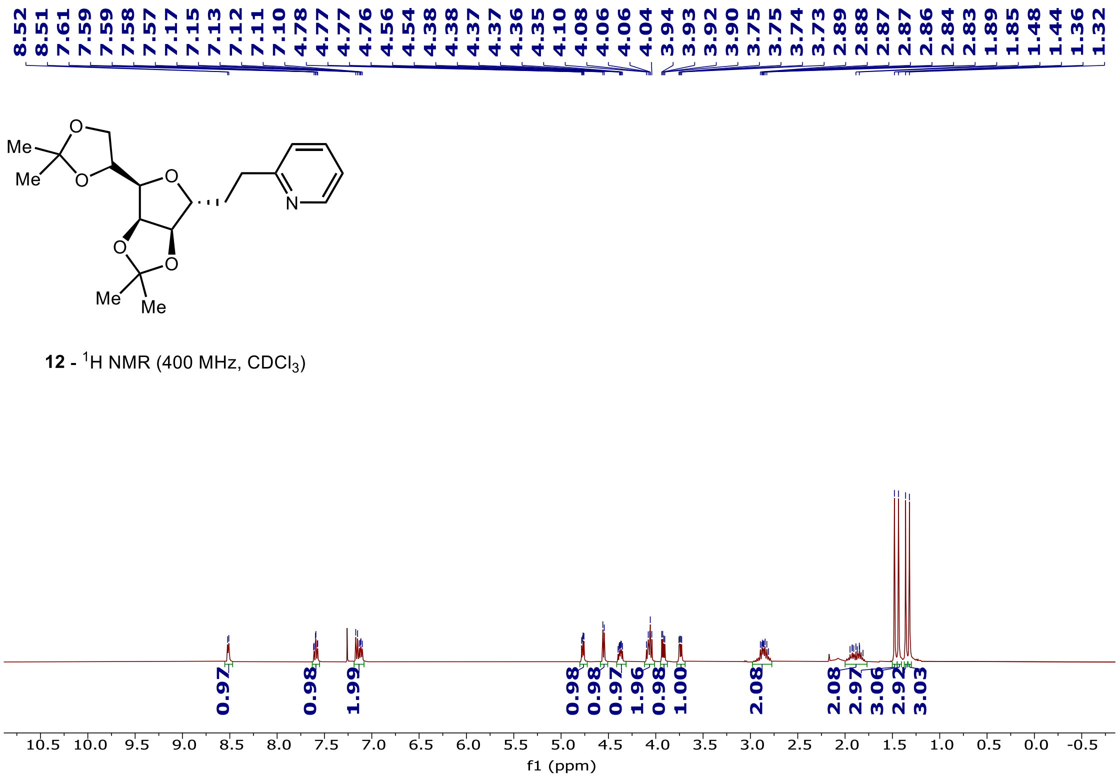

12 - ^{1}H NMR (400 MHz, $CDCl_3$)

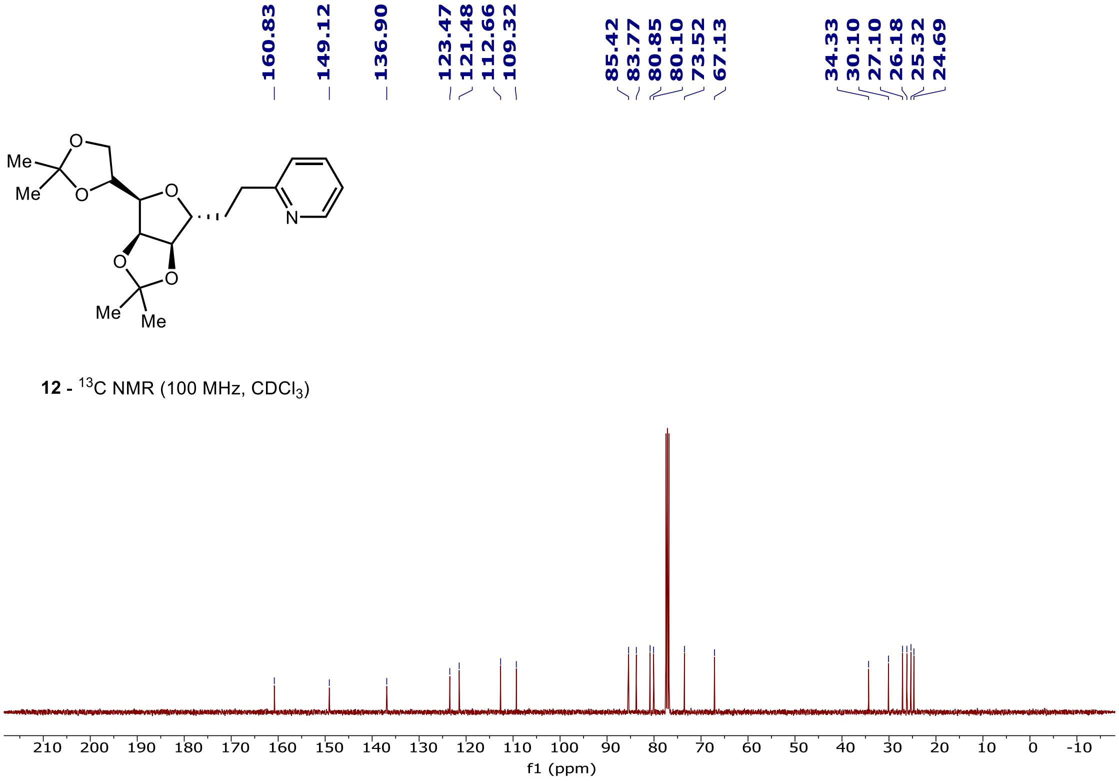

12 - ^{13}C NMR (100 MHz, $CDCl_3$)

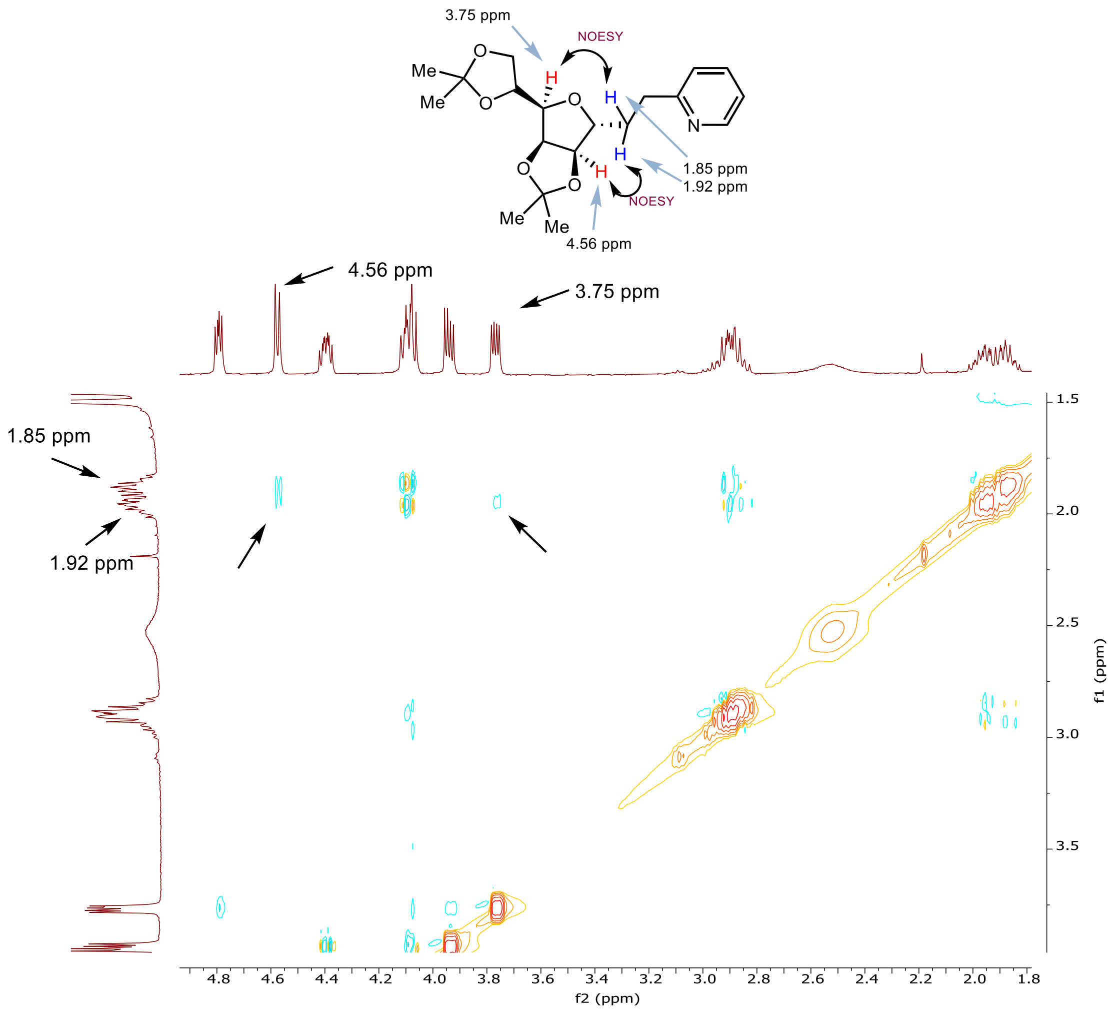
3.75 ppm
NOESY
Me
Me
O
O
O
O
O
Me
Me
H
H
H
H
N
1.85 ppm
1.92 ppm
NOESY
4.56 ppm
4.56 ppm
3.75 ppm
1.85 ppm
1.92 ppm
f1 (ppm)
f2 (ppm)

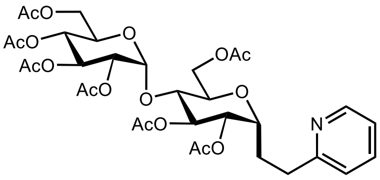

13 - ^{1}H NMR (400 MHz, $CDCl_3$)

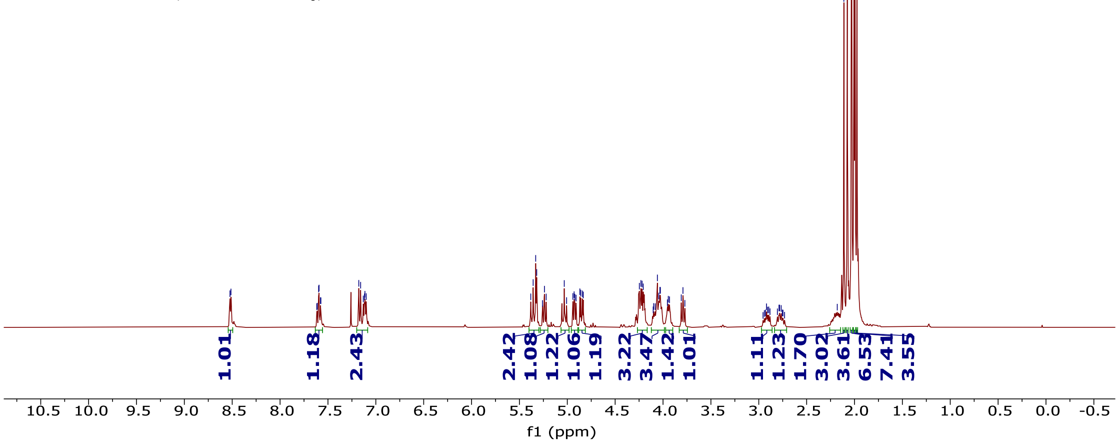

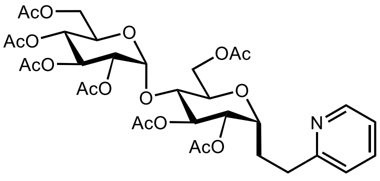

13 - ^{13}C NMR (100 MHz, $CDCl_3$)

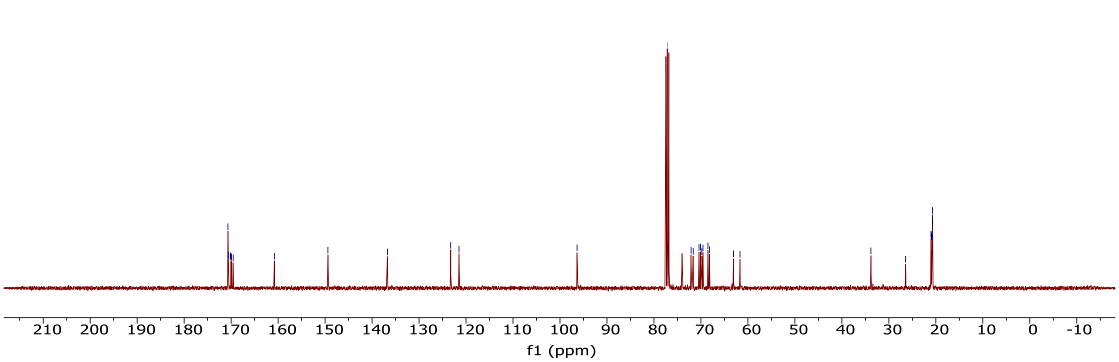

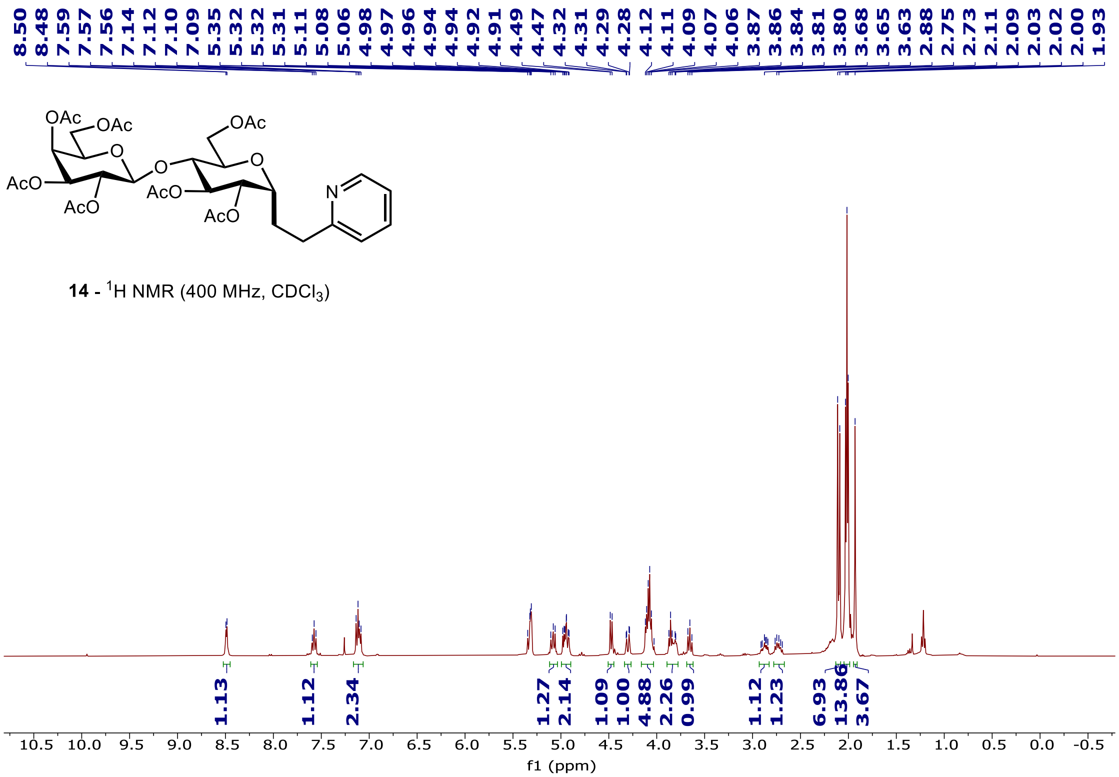

14 - ^{1}H NMR (400 MHz, $CDCl_3$)

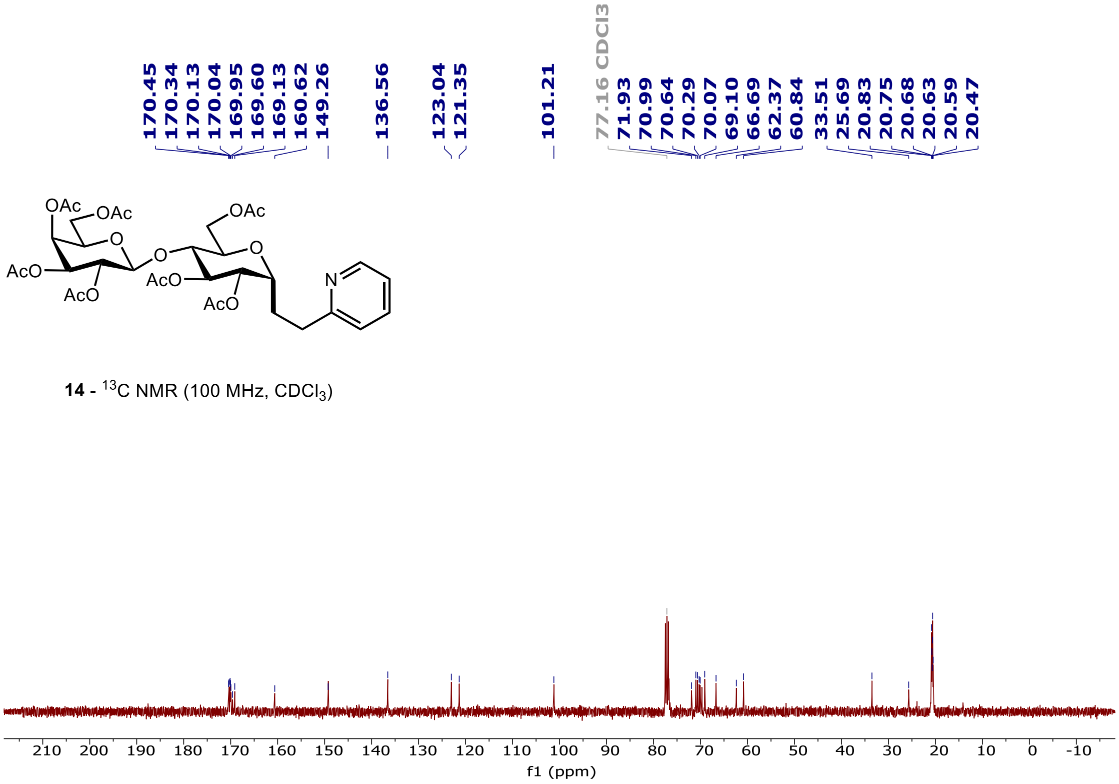

14 - ^{13}C NMR (100 MHz, $CDCl_3$)

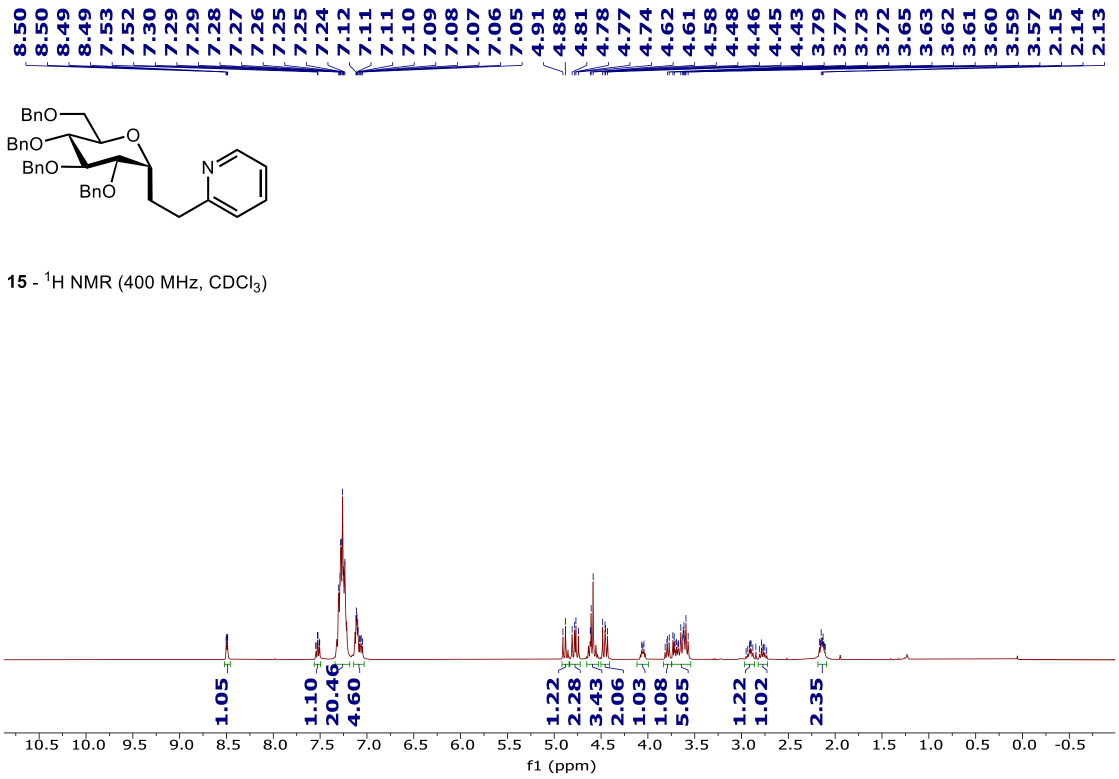

15 - ^{1}H NMR (400 MHz, $CDCl_3$)

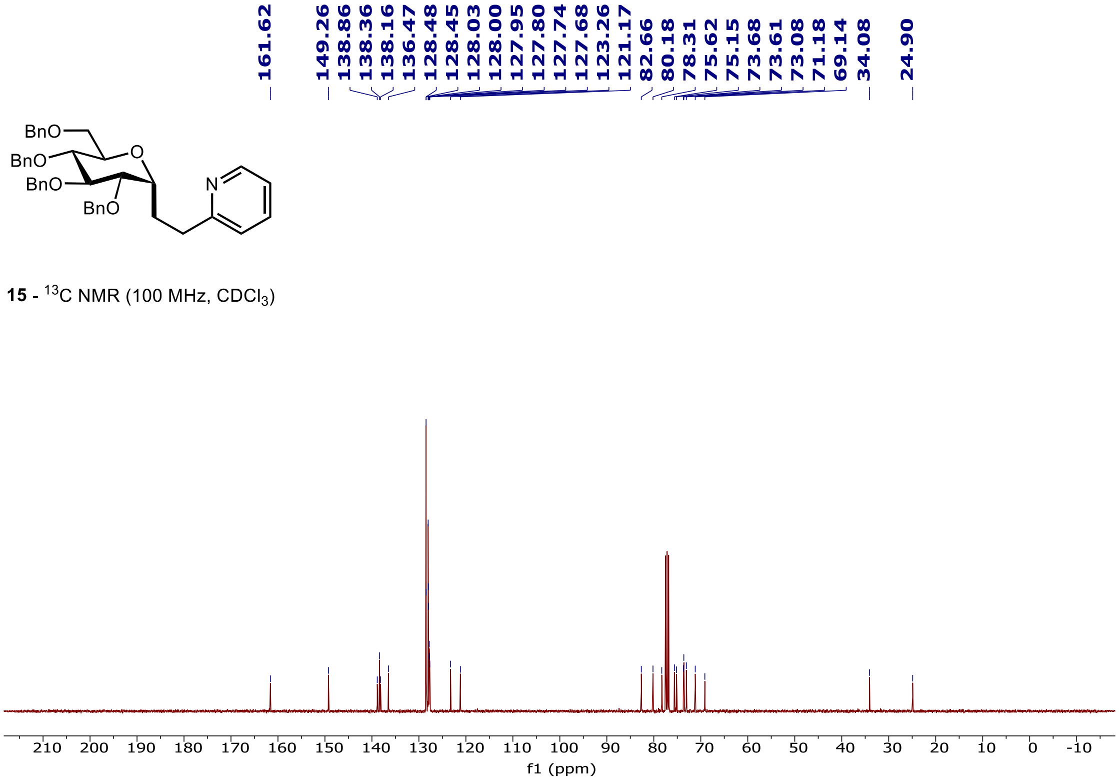

15 - ^{13}C NMR (100 MHz, $CDCl_3$)

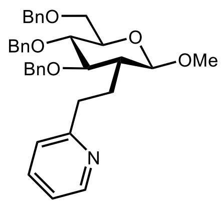

16 - ^{1}H NMR (400 MHz, $CDCl_3$)

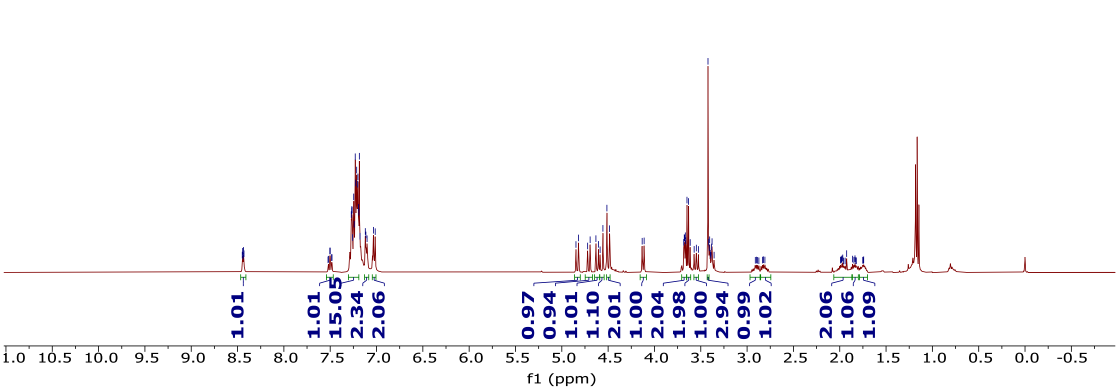

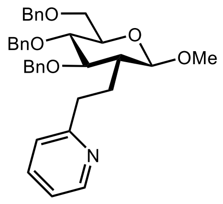

16 - ^{13}C NMR (100 MHz, $CDCl_3$)

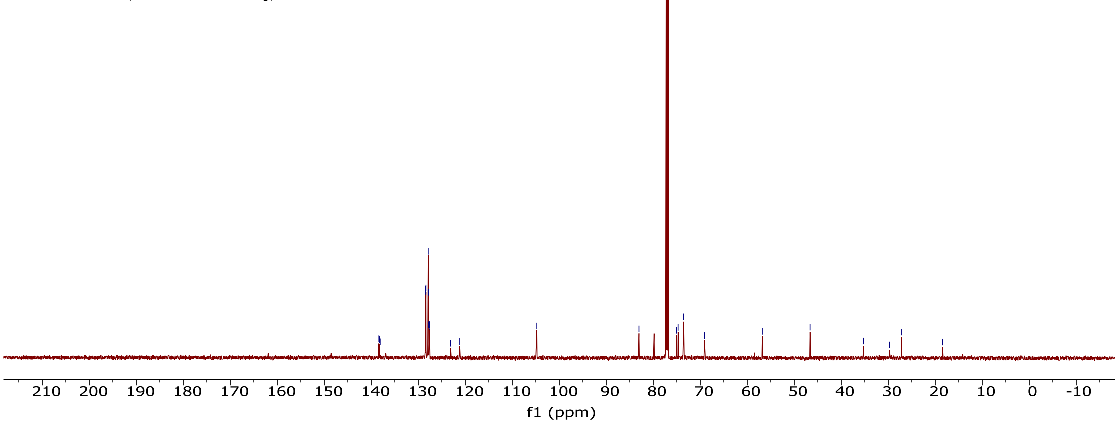

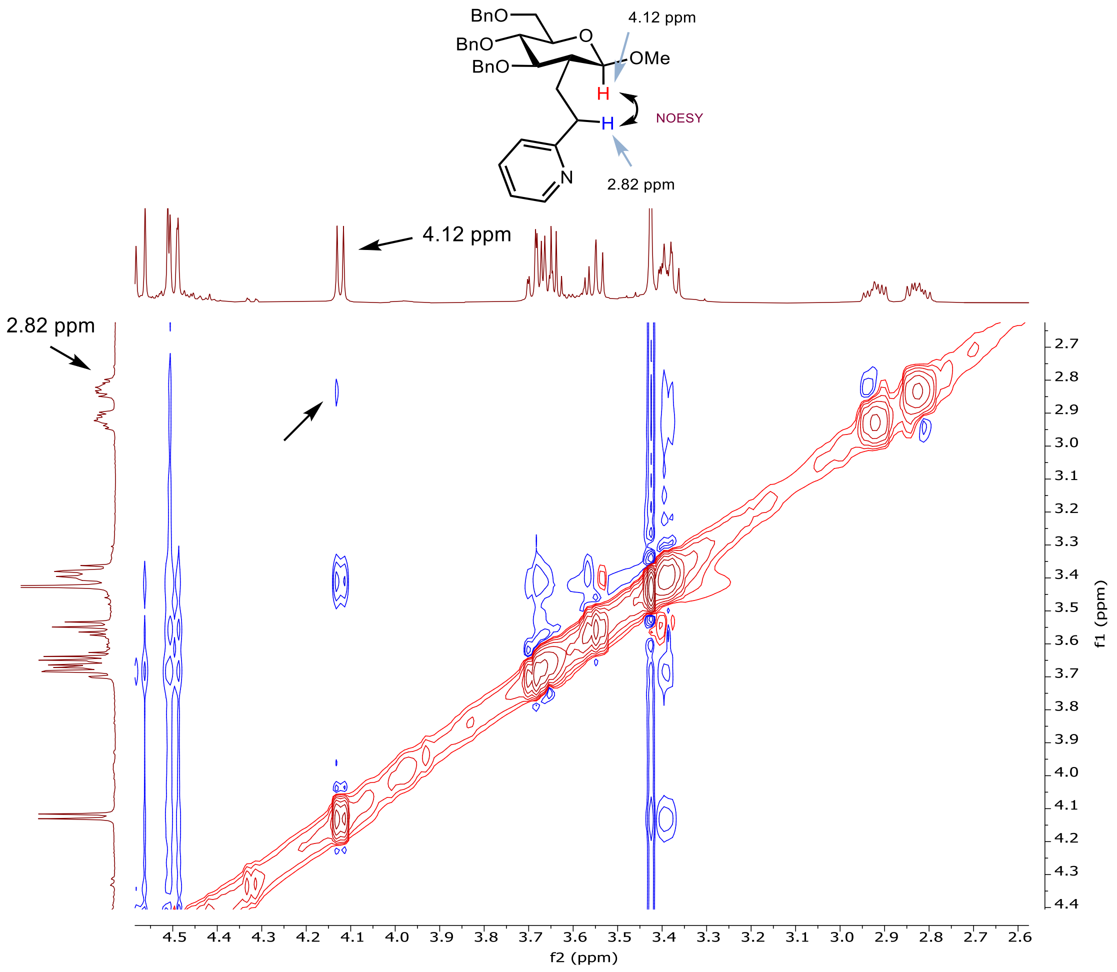
BnO
BnO
BnO
O
OMe
H
H
N
4.12 ppm
NOESY
2.82 ppm
4.12 ppm
2.82 ppm
f1 (ppm)
f2 (ppm)

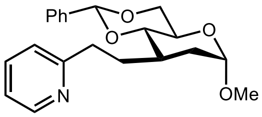

17 - ^{1}H NMR (400 MHz, $CDCl_3$)

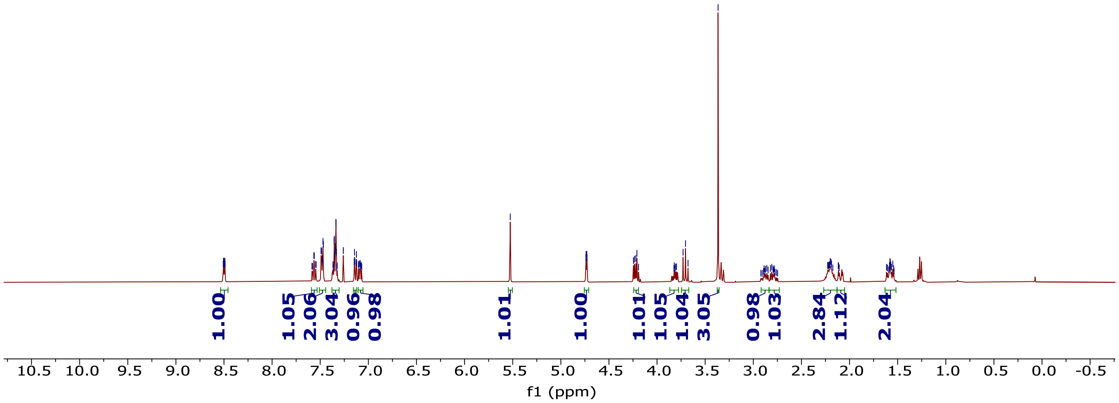

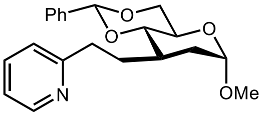

17 - ^{13}C NMR (100 MHz, $CDCl_3$)

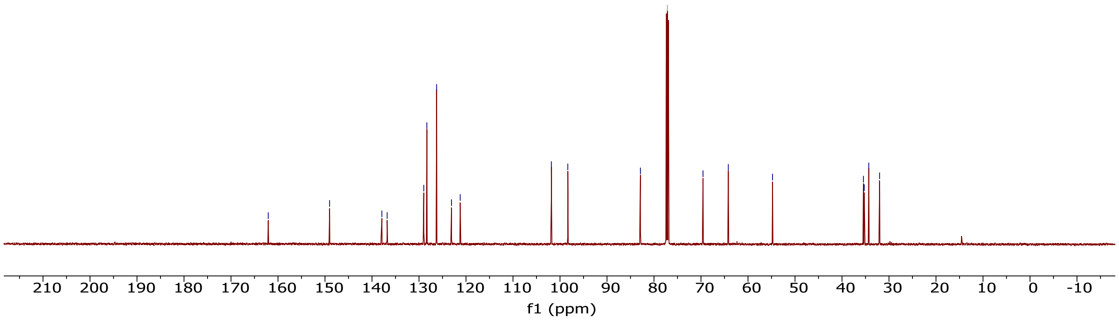

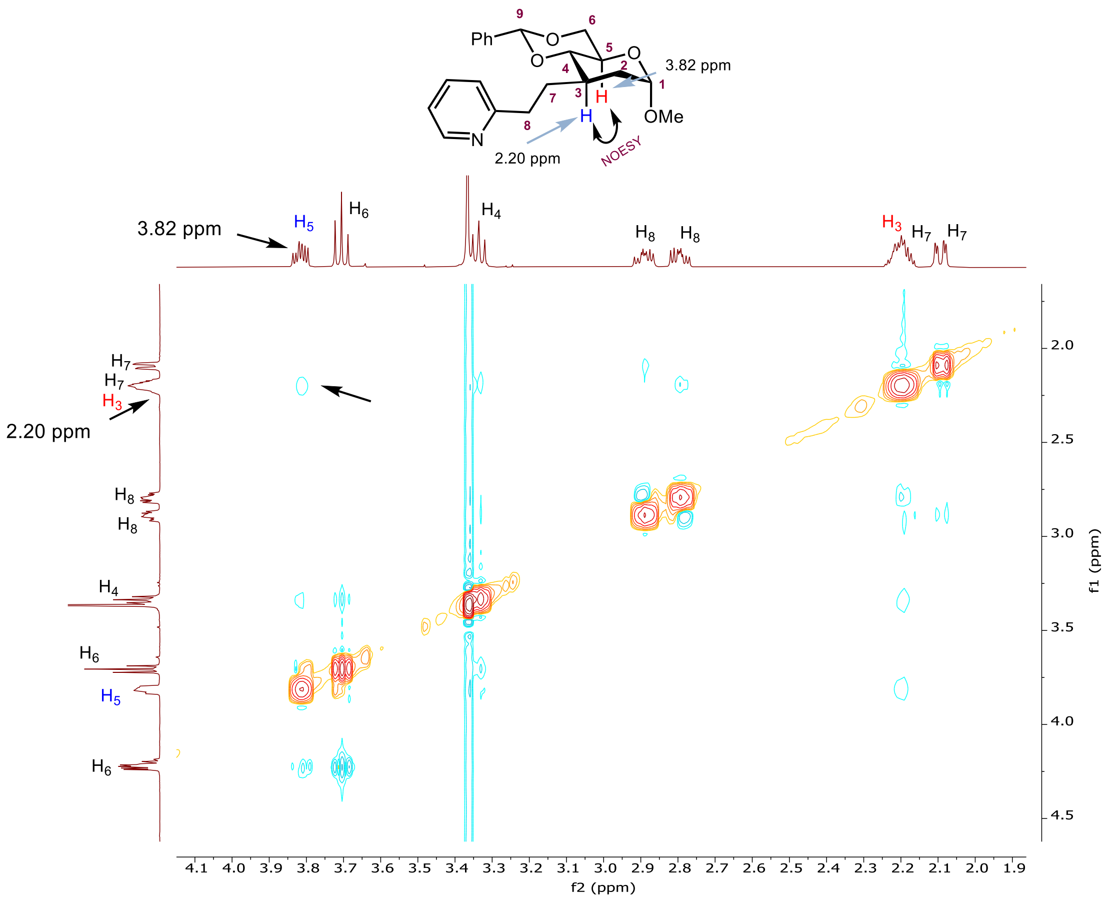

Ph
O
O
O
OMe
N
H
H
9
6
5
4
2
1
3
7
8
3.82 ppm
2.20 ppm
NOESY
H5
H6
H4
H8
H8
H3
H7
H7
H7
H7
H3
H8
H8
H4
H6
H5
H6
f2 (ppm)
f1 (ppm)

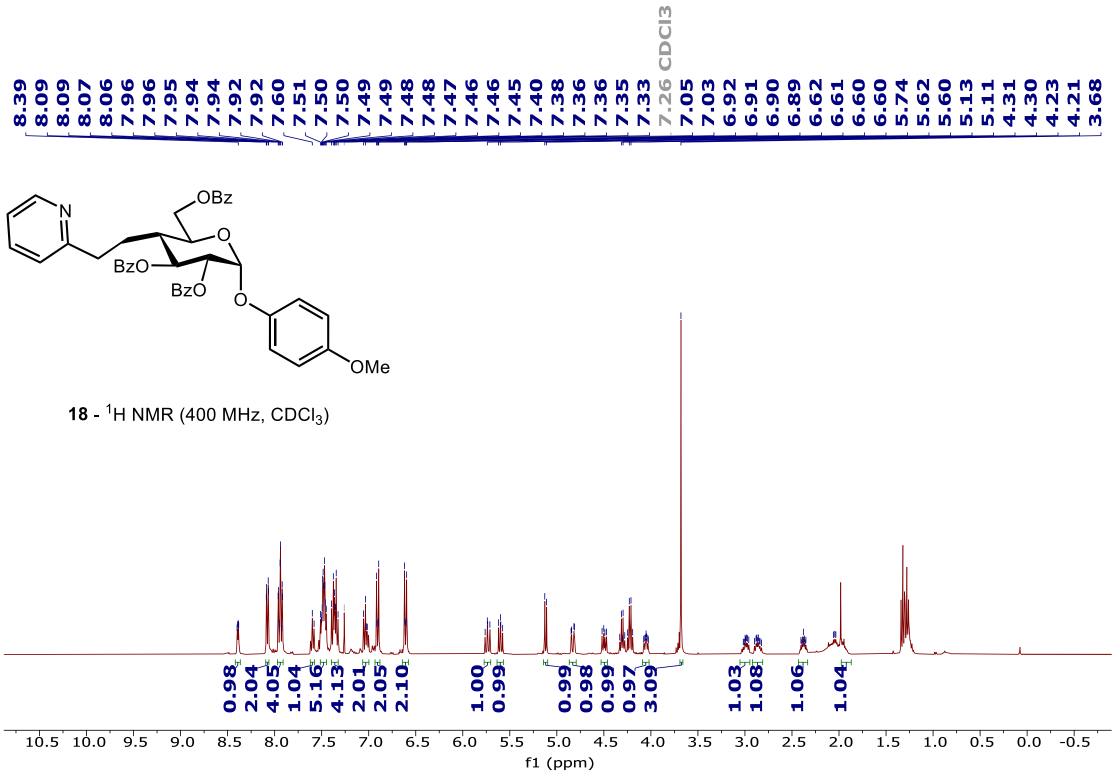

18 - ^{1}H NMR (400 MHz, $CDCl_3$)

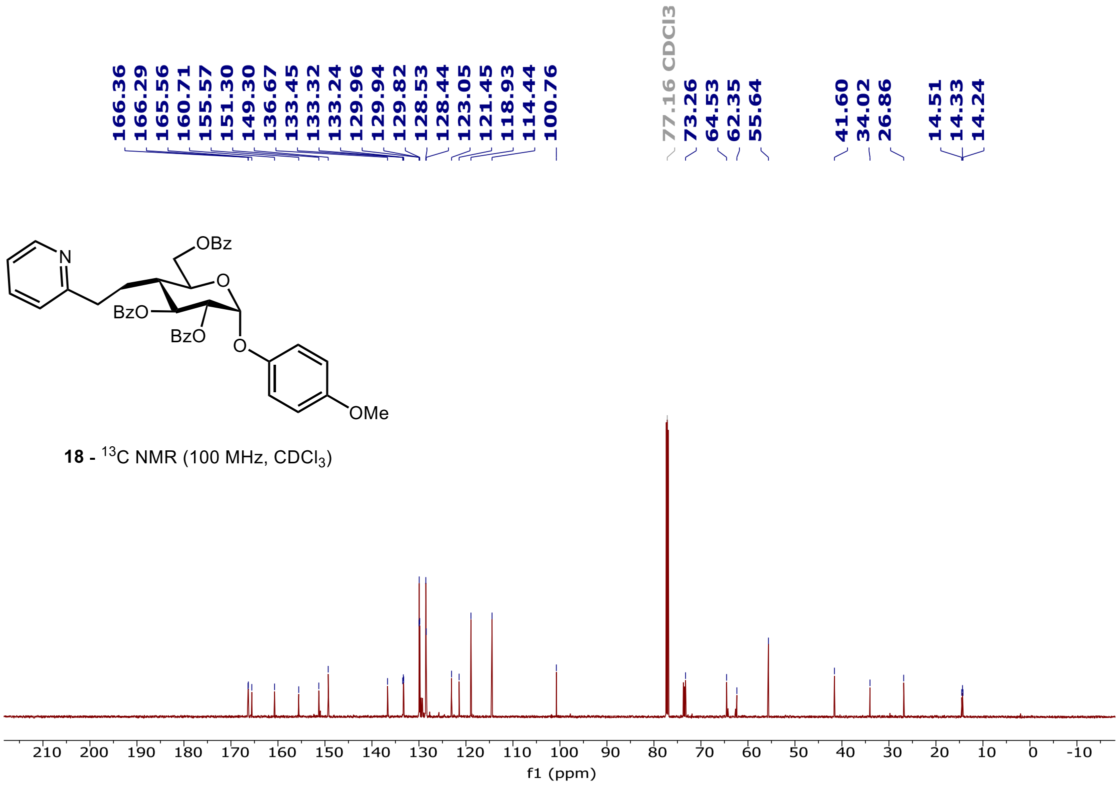

18 - ^{13}C NMR (100 MHz, $CDCl_3$)

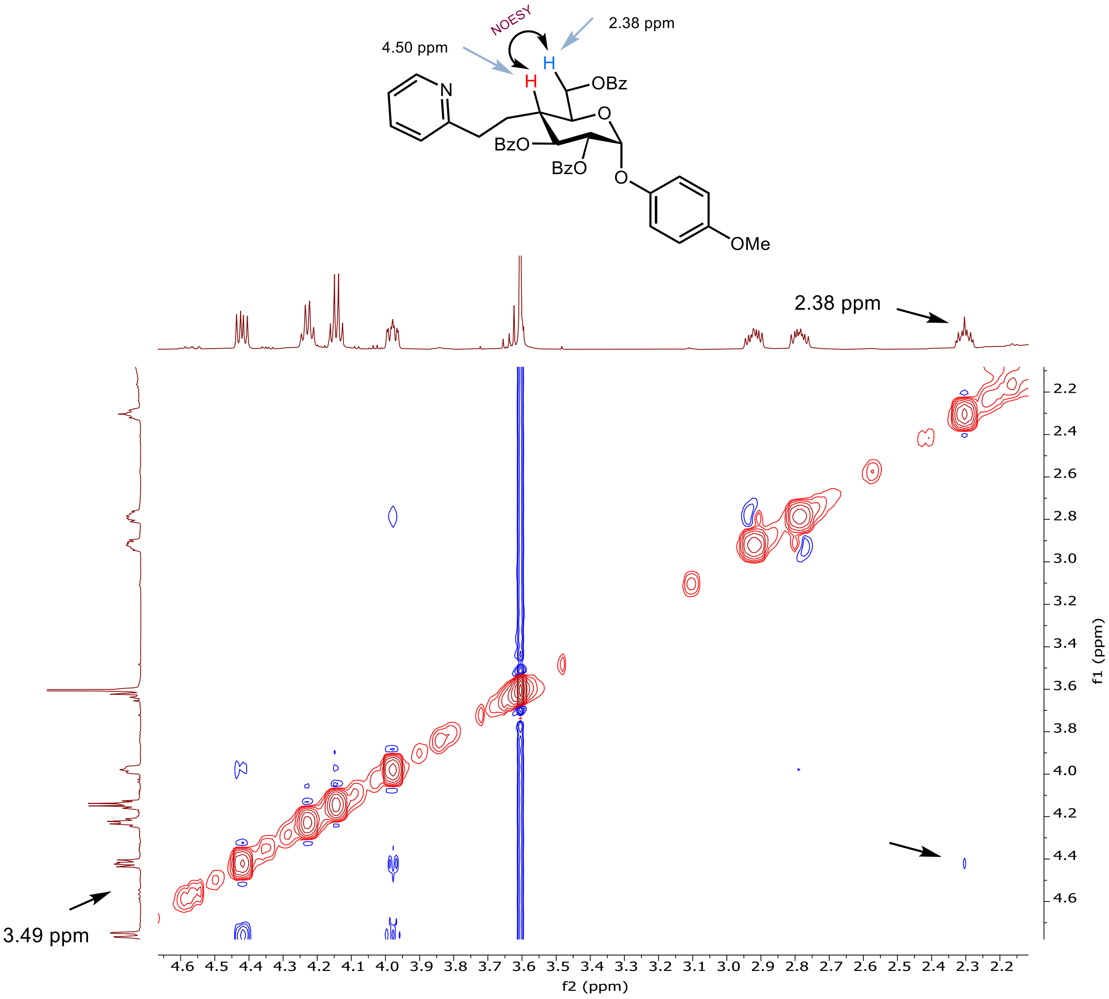
NOESY
4.50 ppm
2.38 ppm
H
H
OBz
N
O
BzO
BzO
O
OMe
2.38 ppm
3.49 ppm
f1 (ppm)
f2 (ppm)

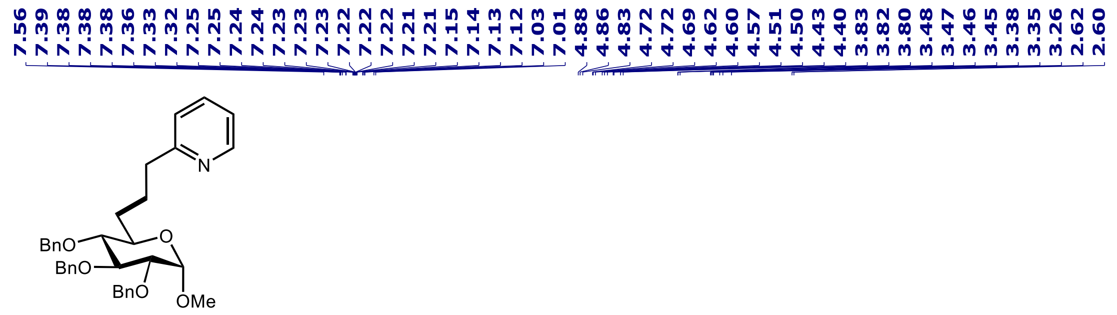

19 - ^{1}H NMR (400 MHz, $CDCl_3$)

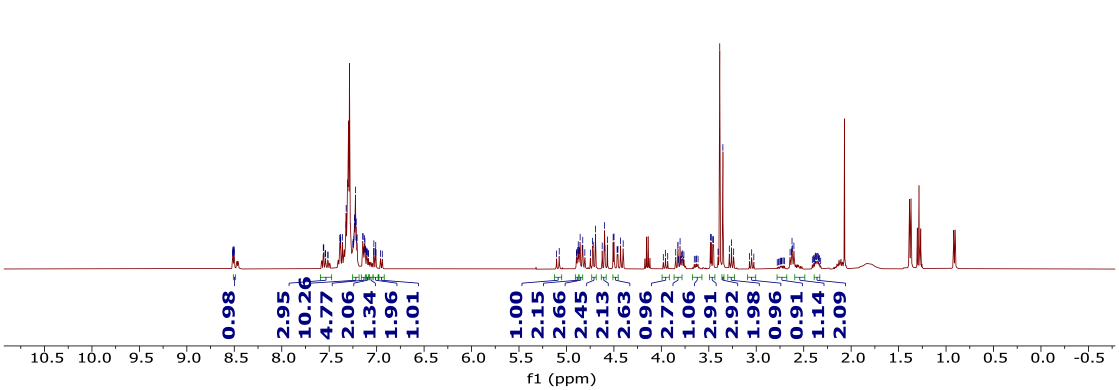

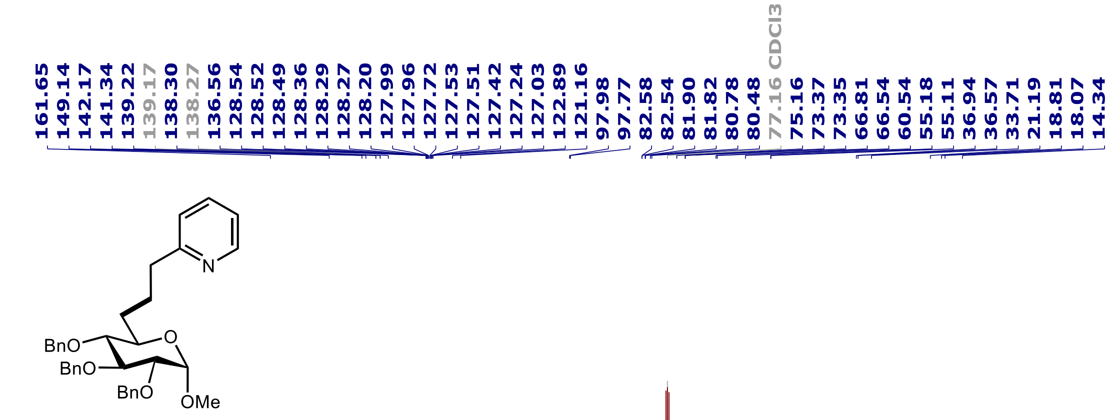

19 - ^{13}C NMR (100 MHz, $CDCl_3$)

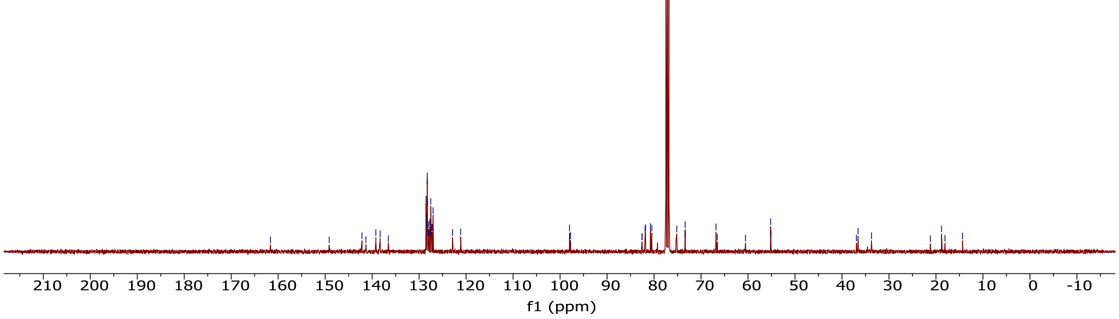

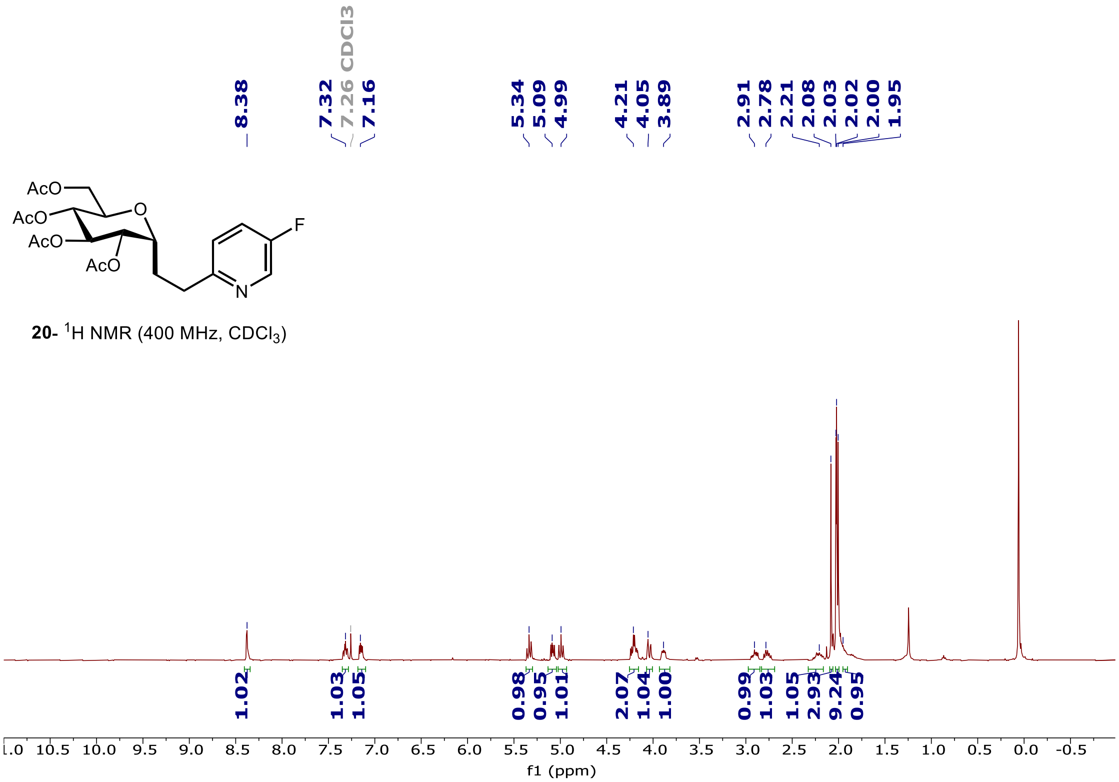

20- ^{1}H NMR (400 MHz, $CDCl_3$)

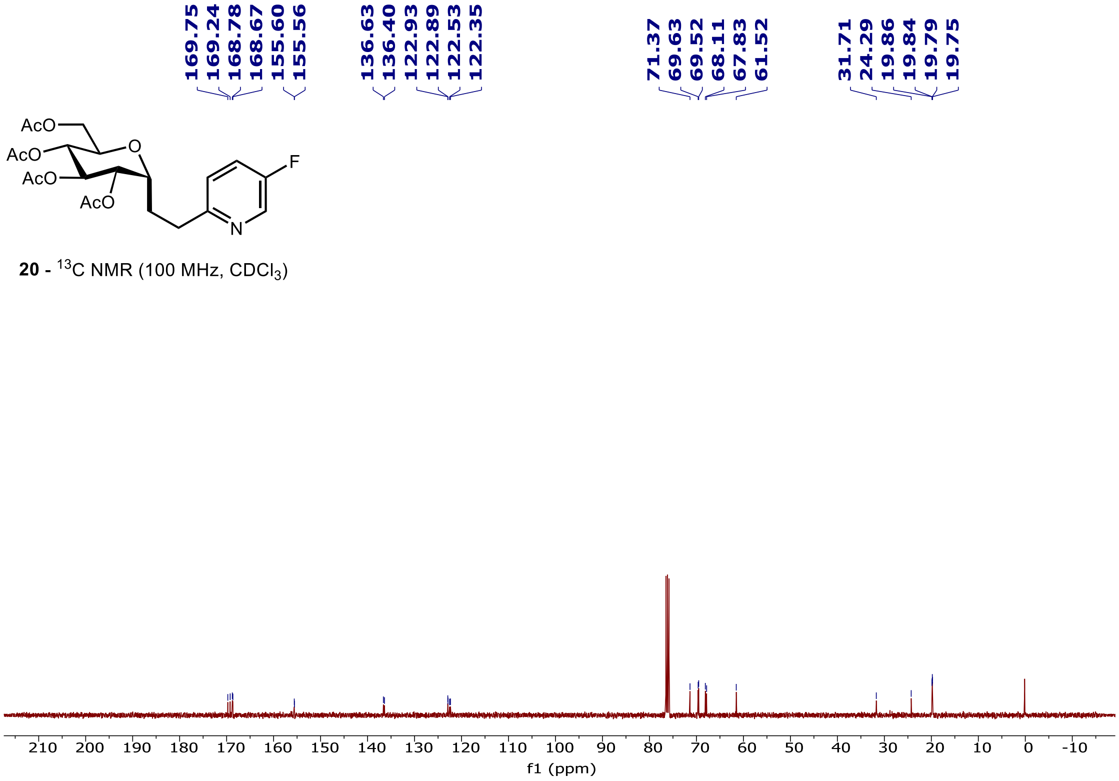

20 - ^{13}C NMR (100 MHz, $CDCl_3$)

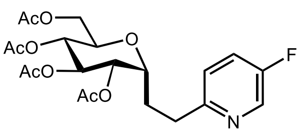

20 - ^{19}F NMR (376 MHz, $CDCl_3$)

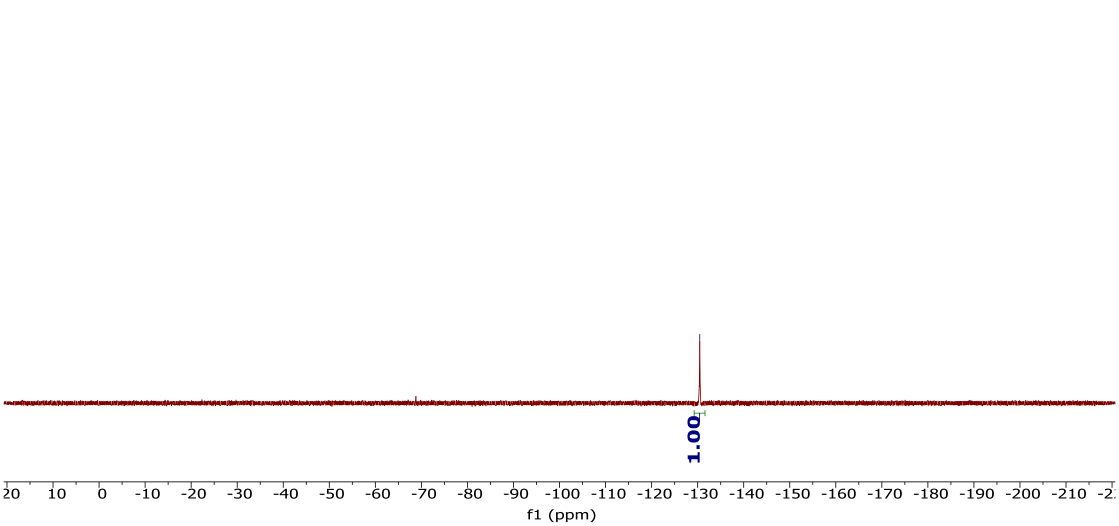

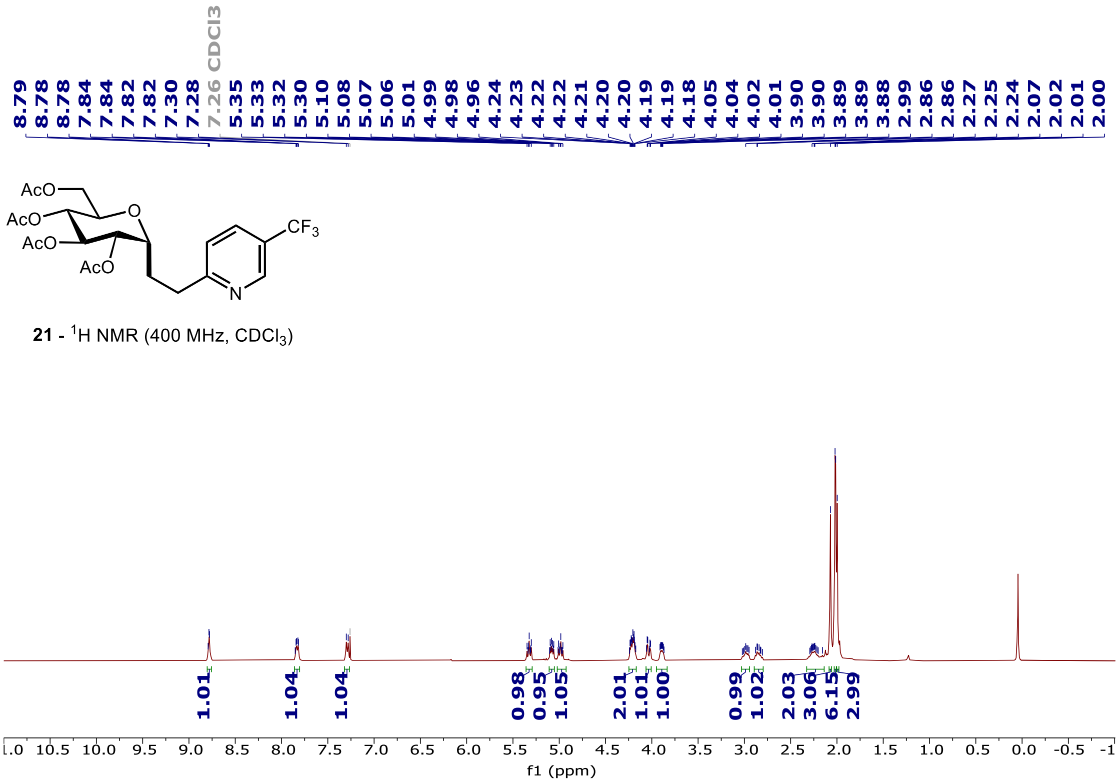

21 - ^{1}H NMR (400 MHz, $CDCl_3$)

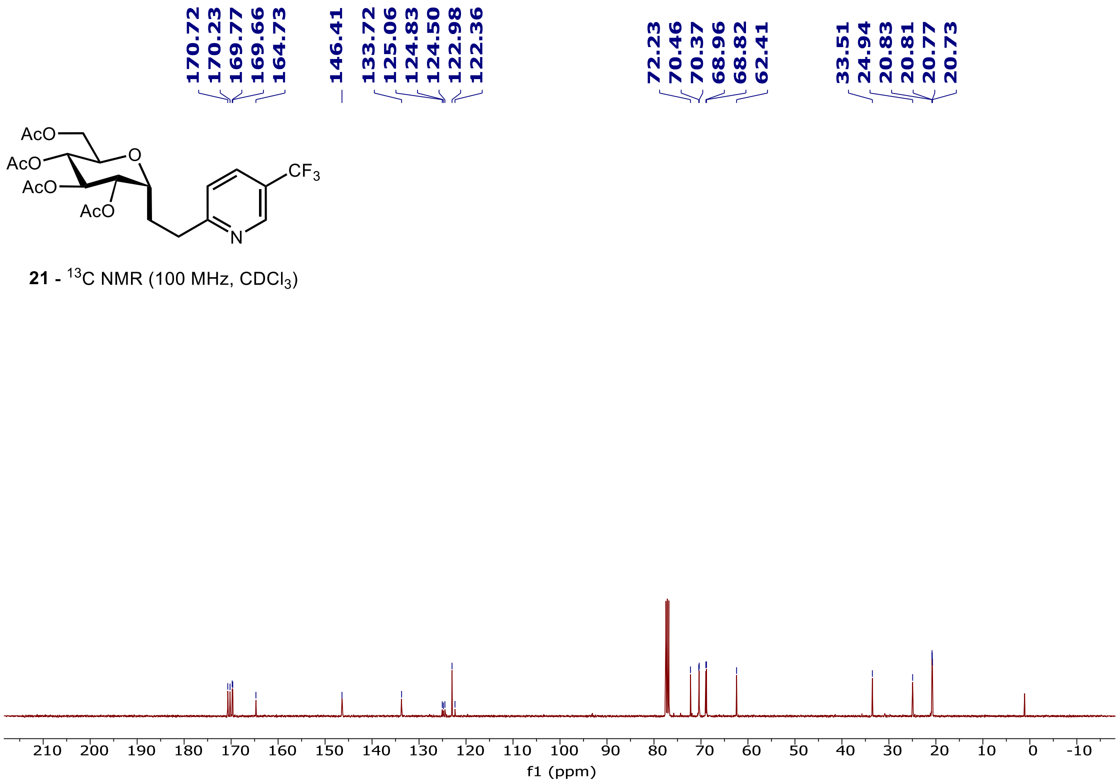

21 - ^{13}C NMR (100 MHz, $CDCl_3$)

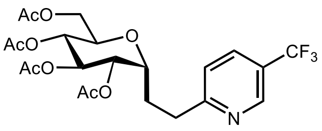

21 - ^{19}F NMR (376 MHz, $CDCl_3$)

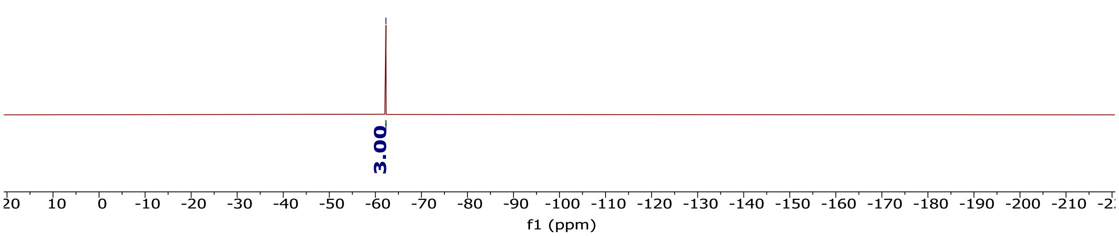

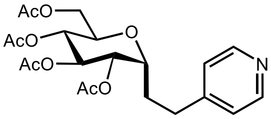

22 - ^{1}H NMR (400 MHz, $CDCl_3$)

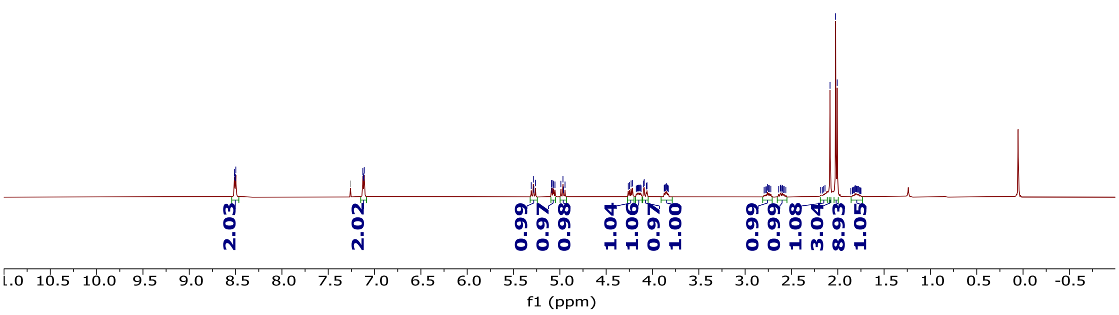

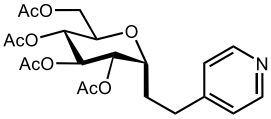

22 - ^{13}C NMR (100 MHz, $CDCl_3$)

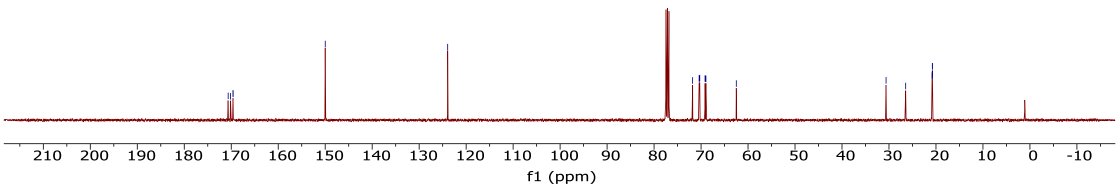

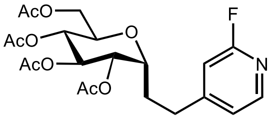

23 - ^{1}H NMR (400 MHz, $CDCl_3$)

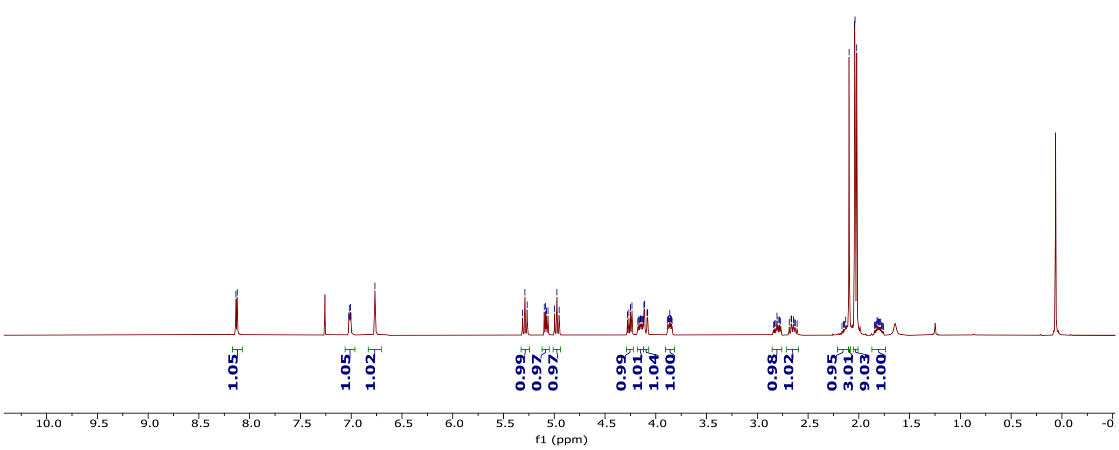

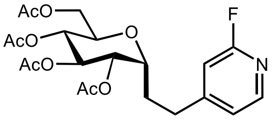

23 - ^{13}C NMR (100 MHz, $CDCl_3$)

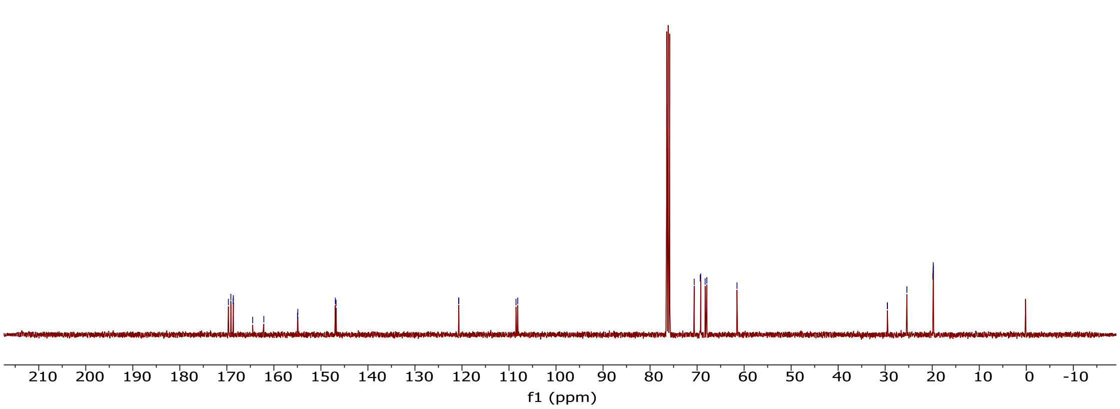

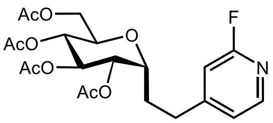

23 - ^{19}F NMR (376 MHz, $CDCl_3$)

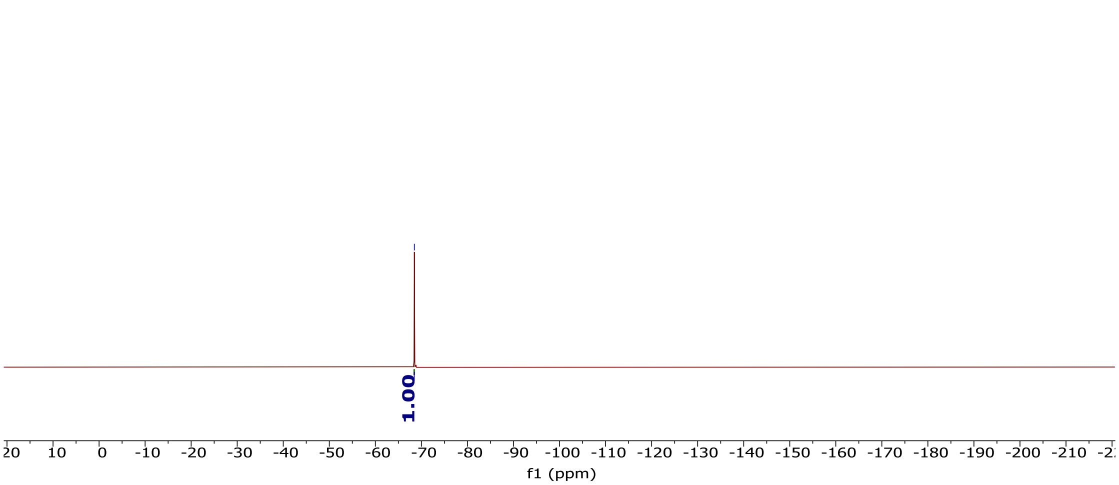

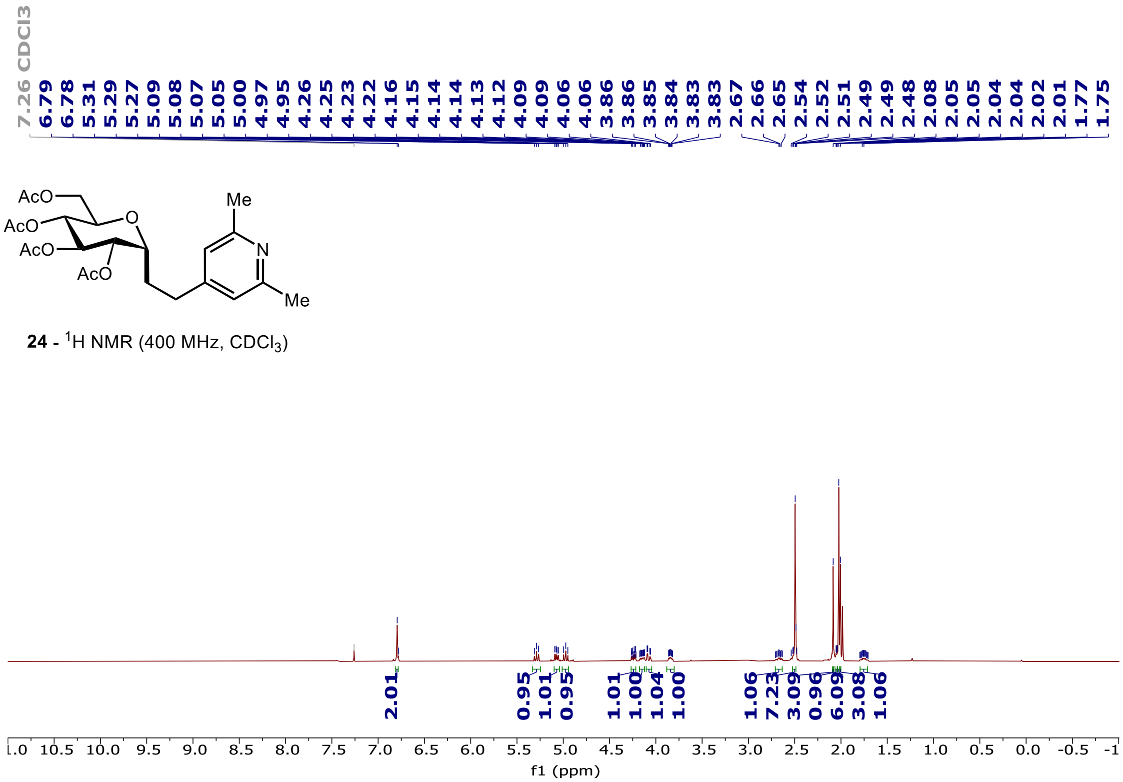

24 - ^{1}H NMR (400 MHz, $CDCl_3$)

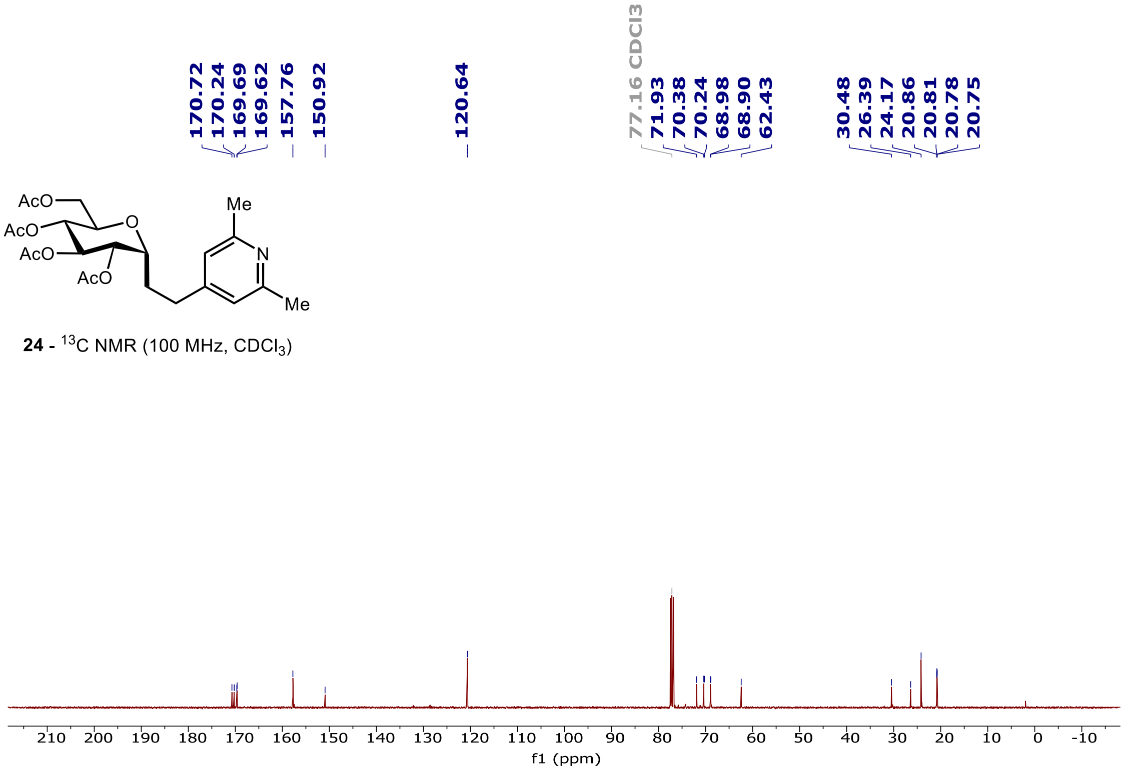

24 - ^{13}C NMR (100 MHz, $CDCl_3$)

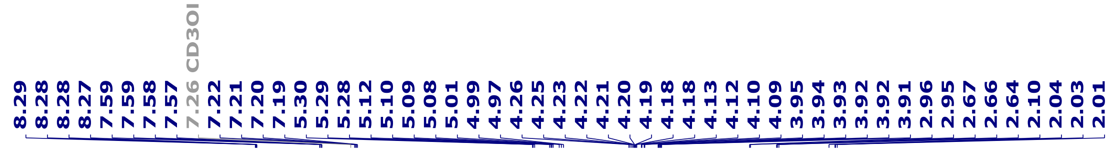

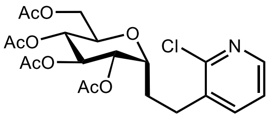

25 - ¹H NMR (400 MHz, $CDCl_3$)

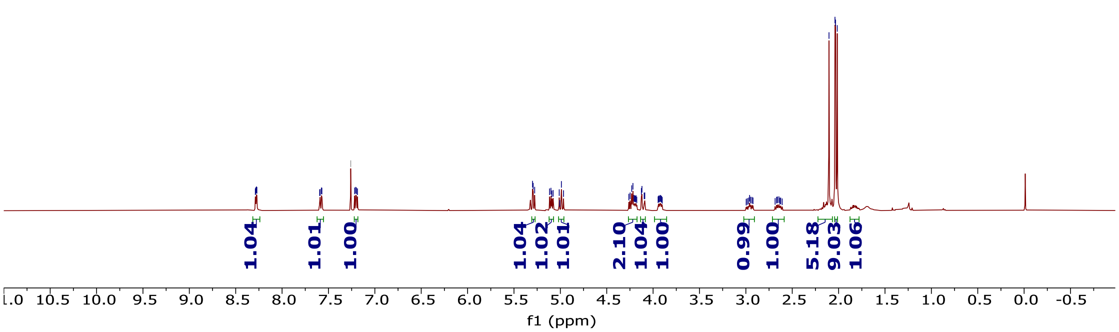

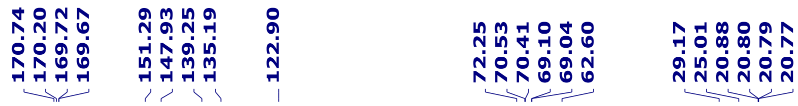

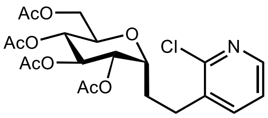

25 - ¹³C NMR (100 MHz, $CDCl_3$)

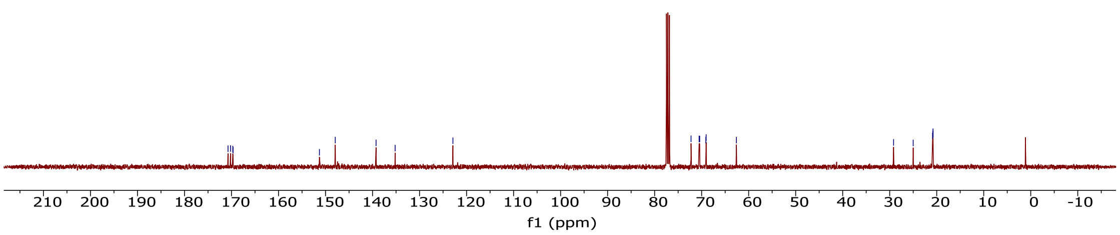

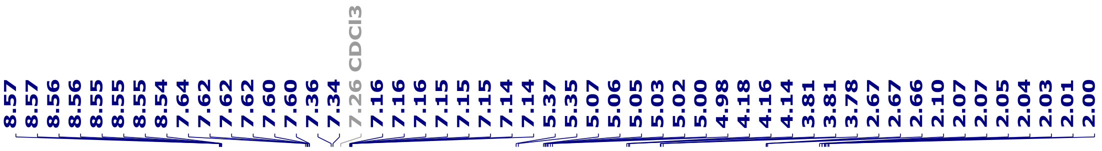

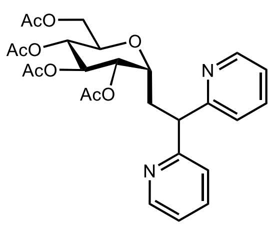

26 - ^{1}H NMR (400 MHz, $CDCl_3$)

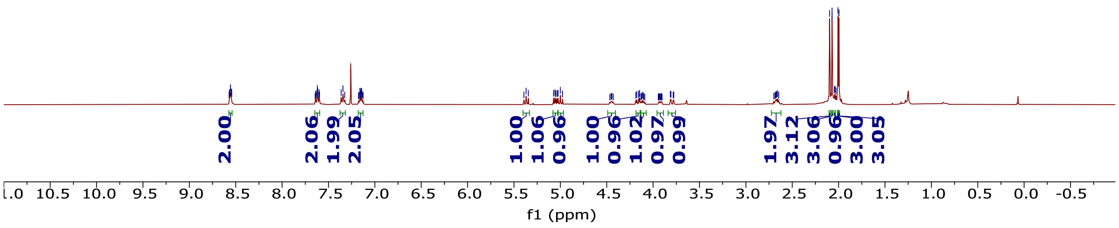

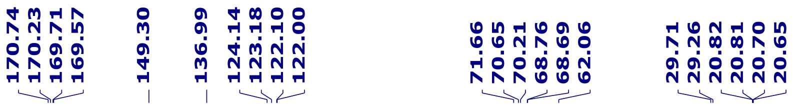

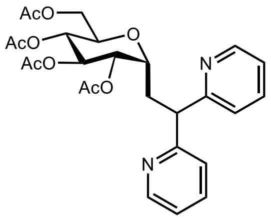

26 - ^{13}C NMR (100 MHz, $CDCl_3$)

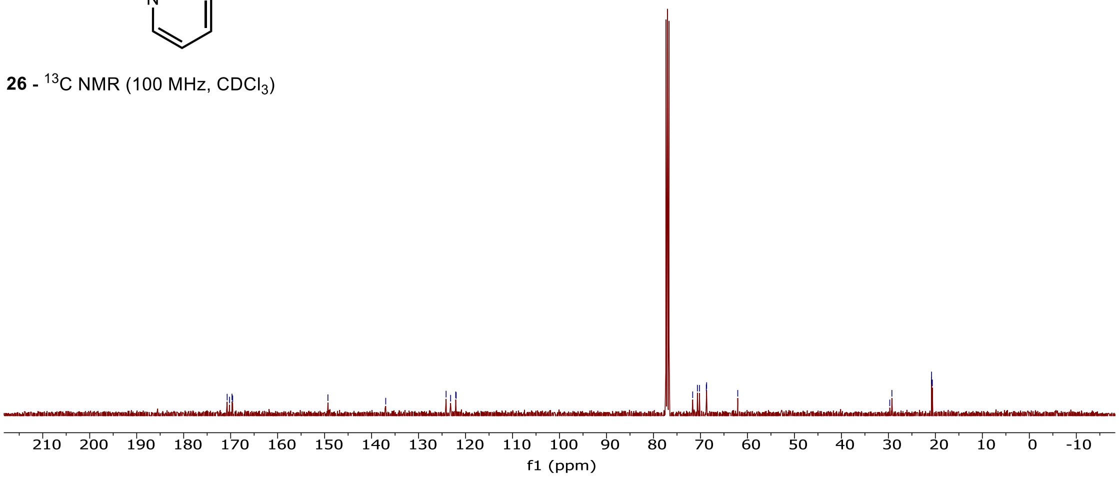

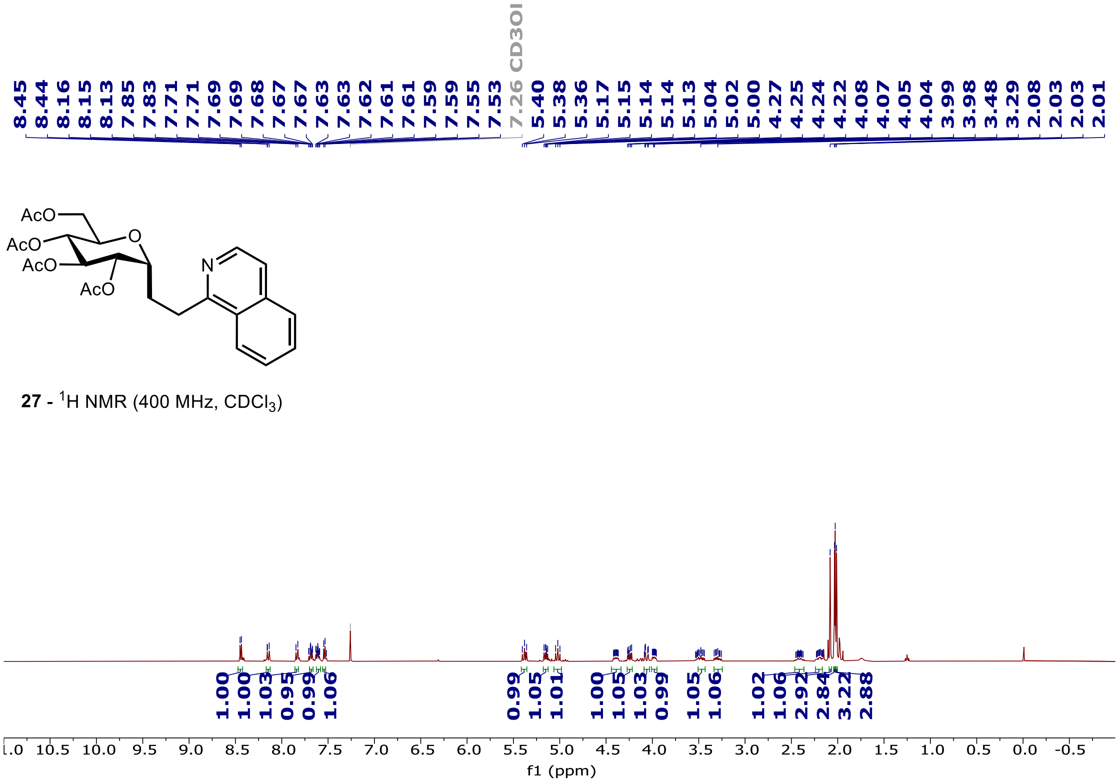

27 - ^{1}H NMR (400 MHz, $CDCl_3$)

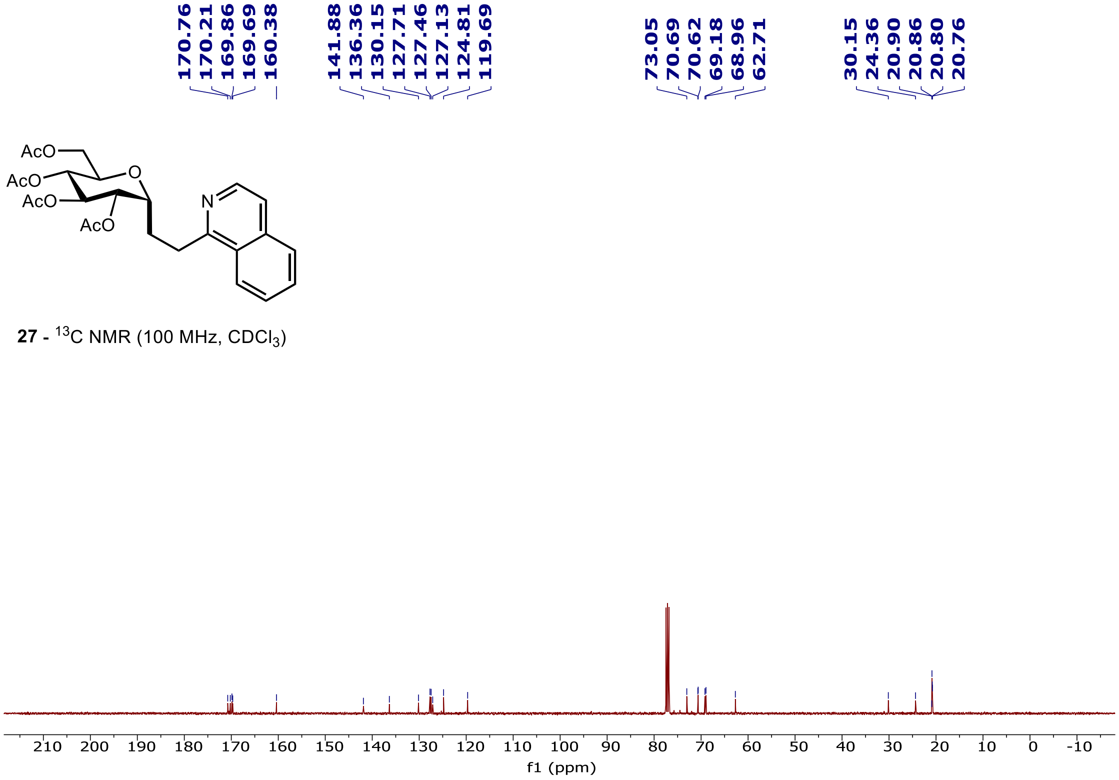

27 - ^{13}C NMR (100 MHz, $CDCl_3$)

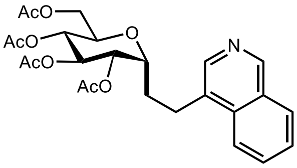

28 - ^{1}H NMR (400 MHz, $CDCl_3$)

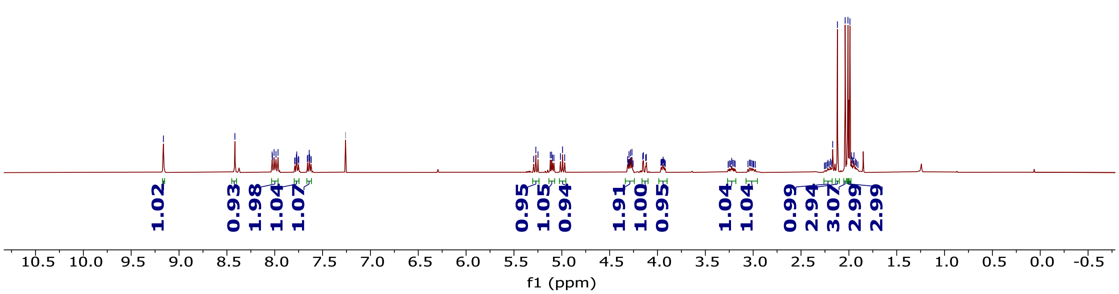

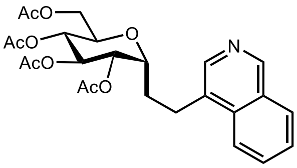

28 - ^{13}C NMR (100 MHz, $CDCl_3$)

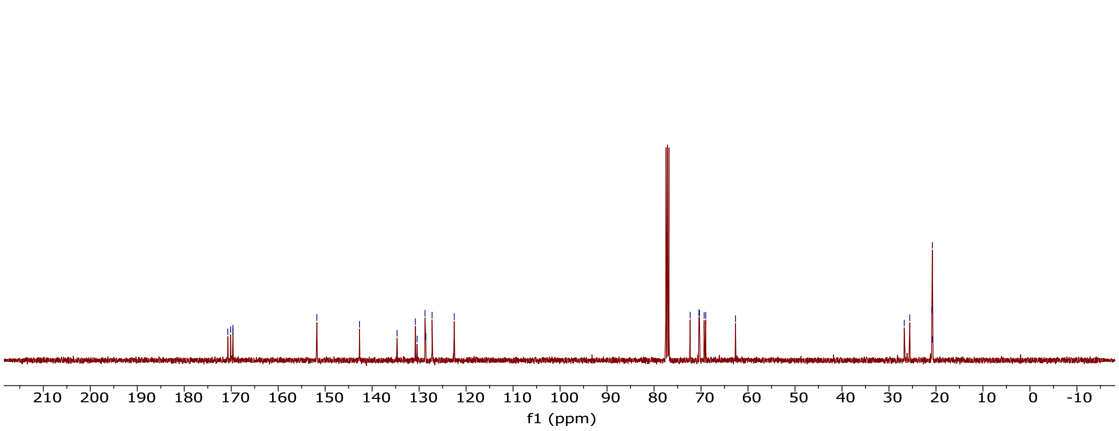

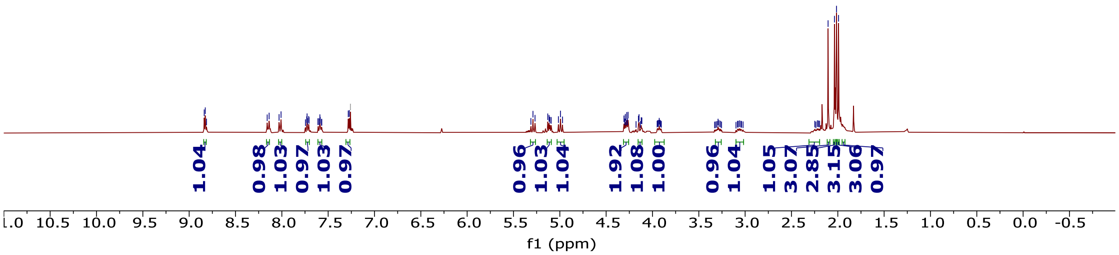

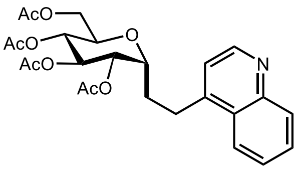

29 - ^{1}H NMR (400 MHz, $CDCl_3$)

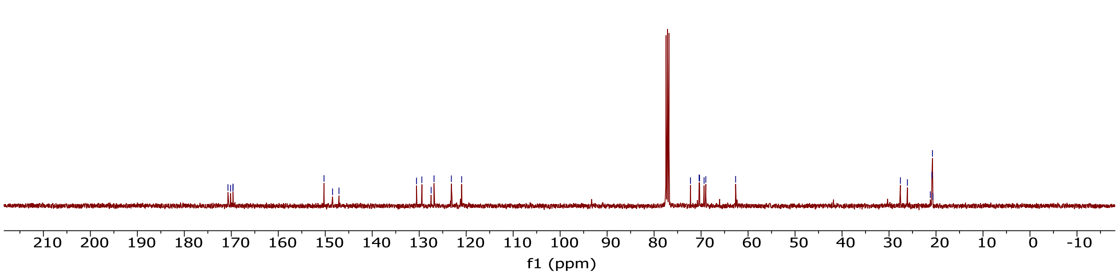

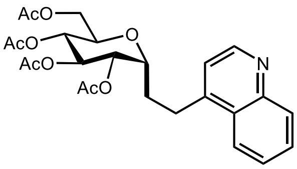

29 - ^{13}C NMR (100 MHz, $CDCl_3$)

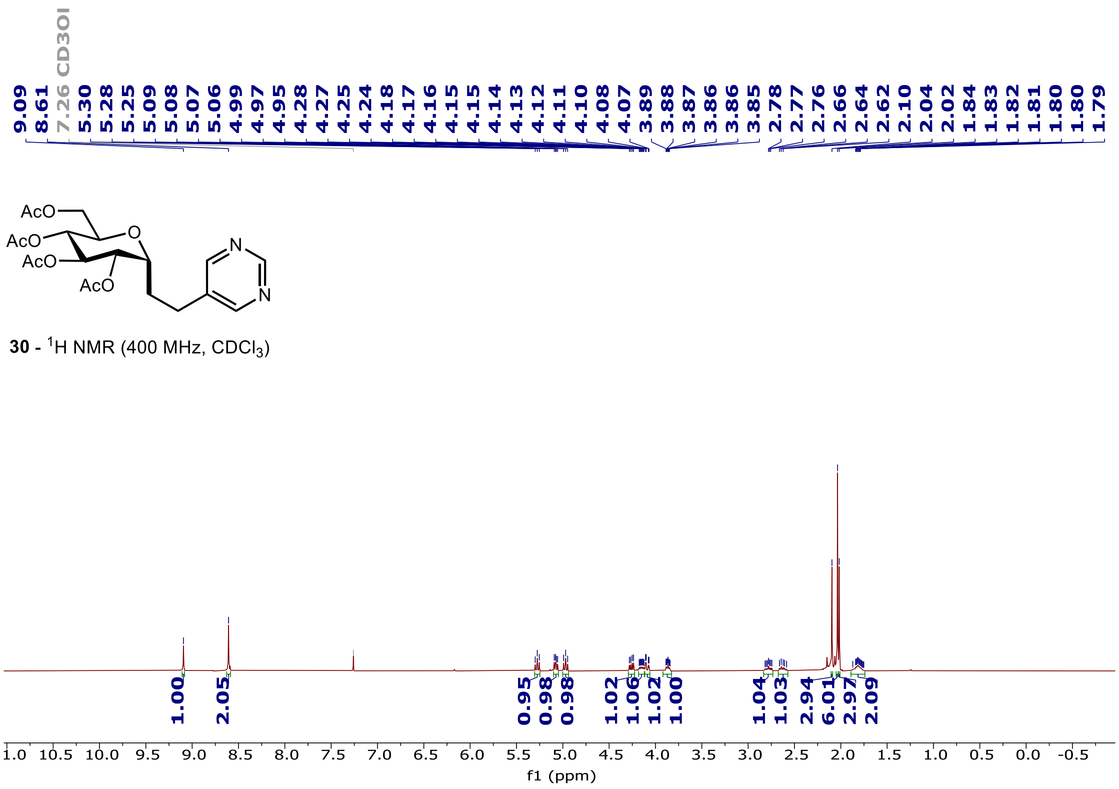

30 - ^{1}H NMR (400 MHz, $CDCl_3$)

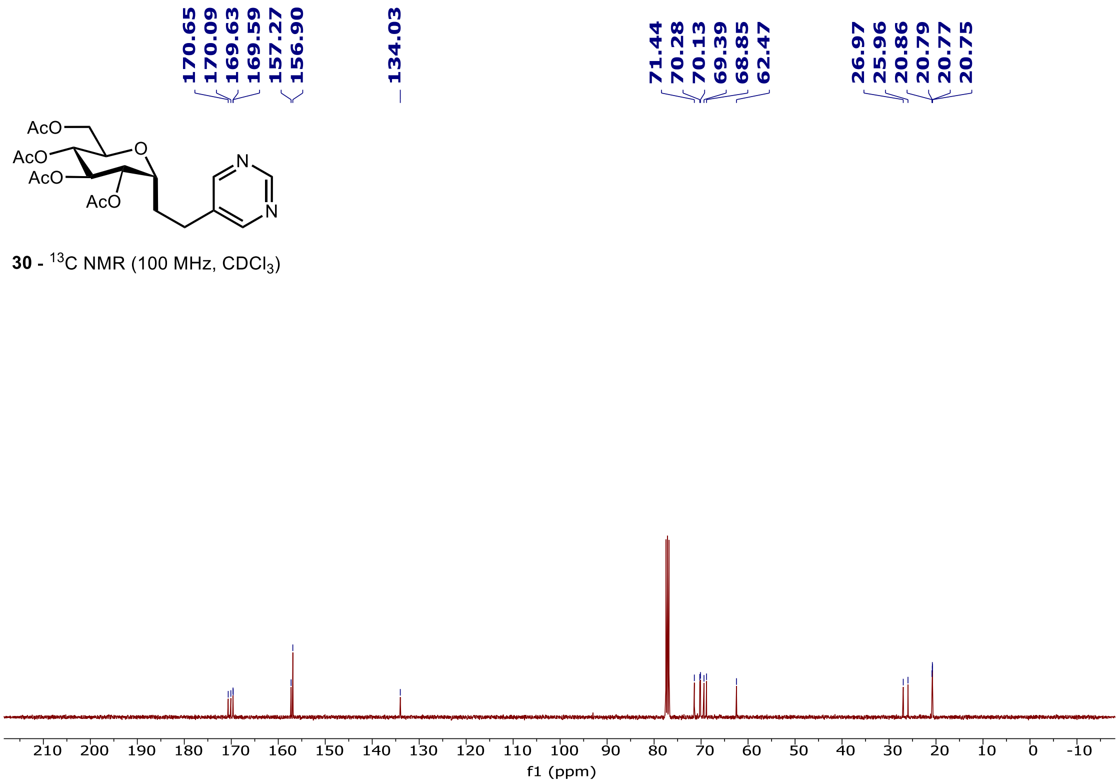

30 - ^{13}C NMR (100 MHz, $CDCl_3$)

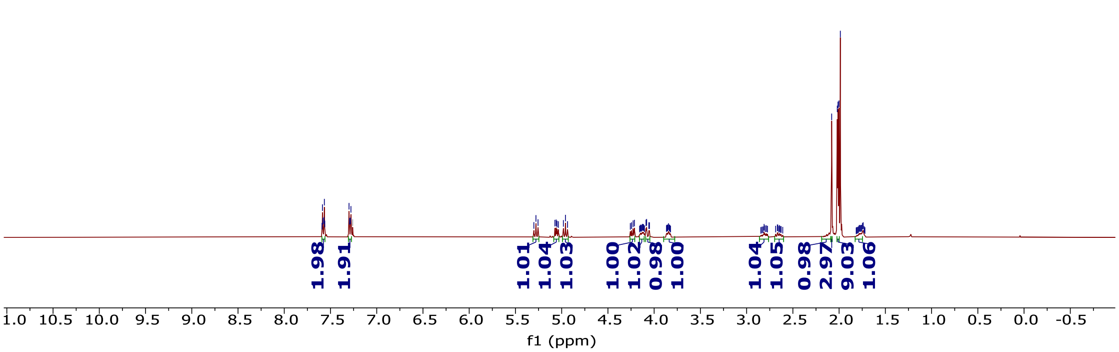

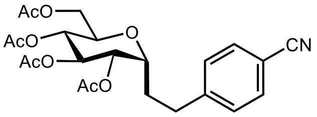

31 - ^{1}H NMR (400 MHz, CDCl$_3$)

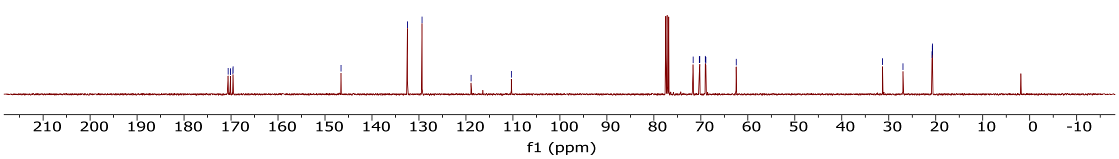

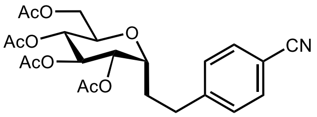

31 - ^{13}C NMR (100 MHz, CDCl$_3$)

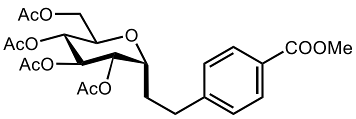

32 - ^{1}H NMR (400 MHz, $CDCl_3$)

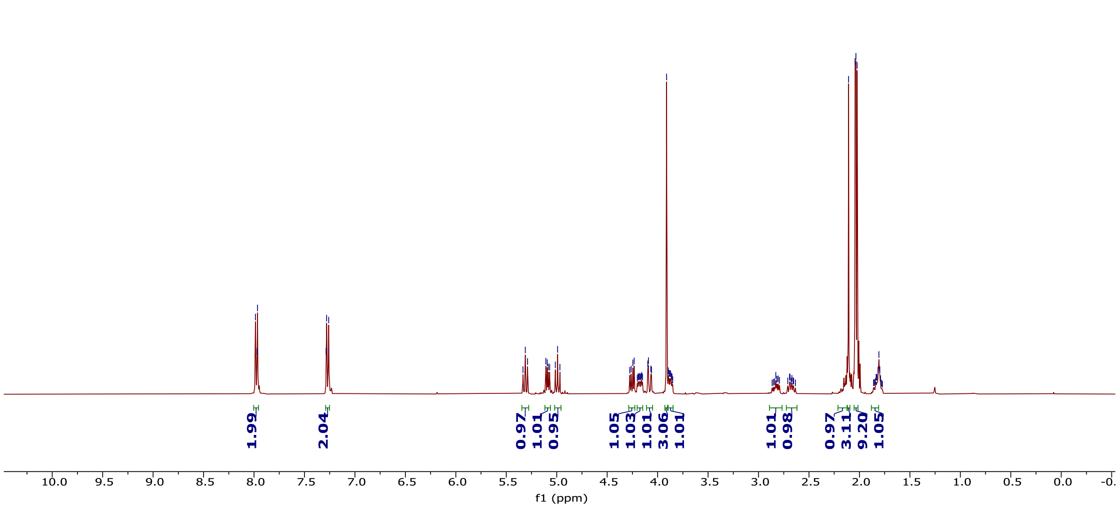

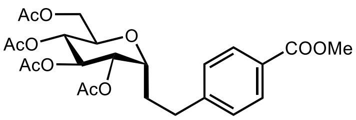

32 - ^{13}C NMR (100 MHz, $CDCl_3$)

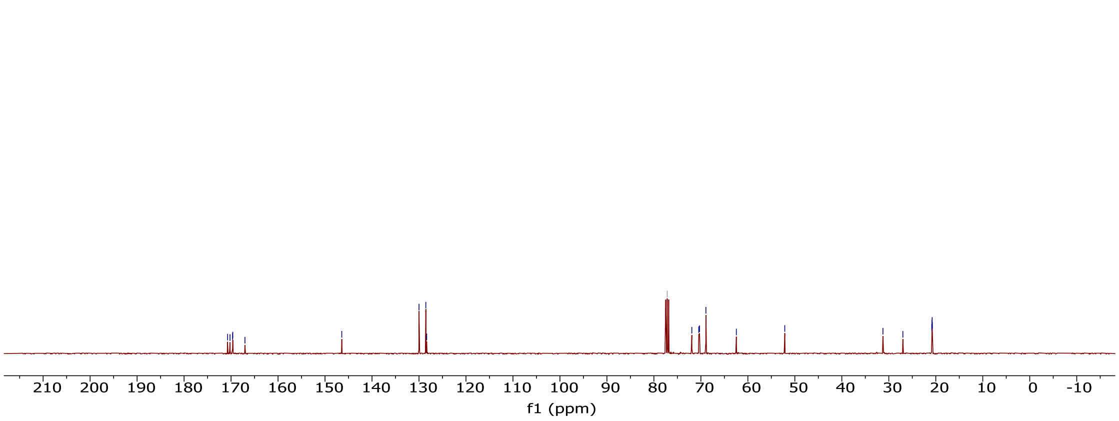

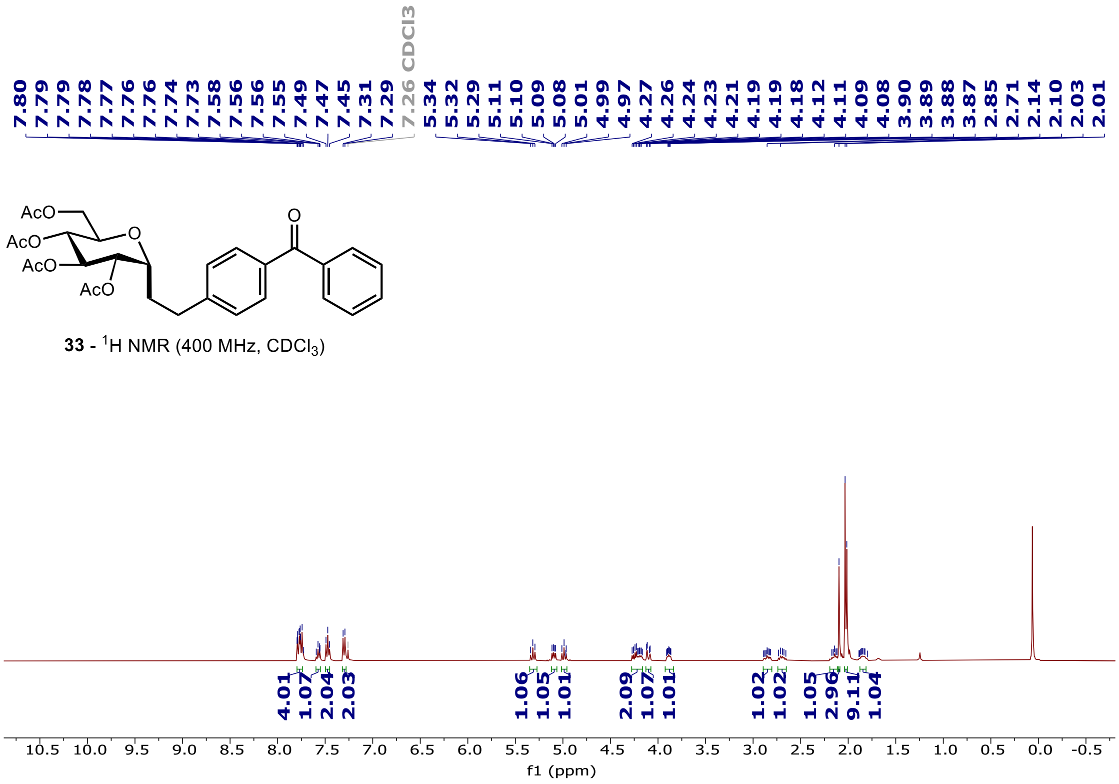

33 - ^{1}H NMR (400 MHz, $CDCl_3$)

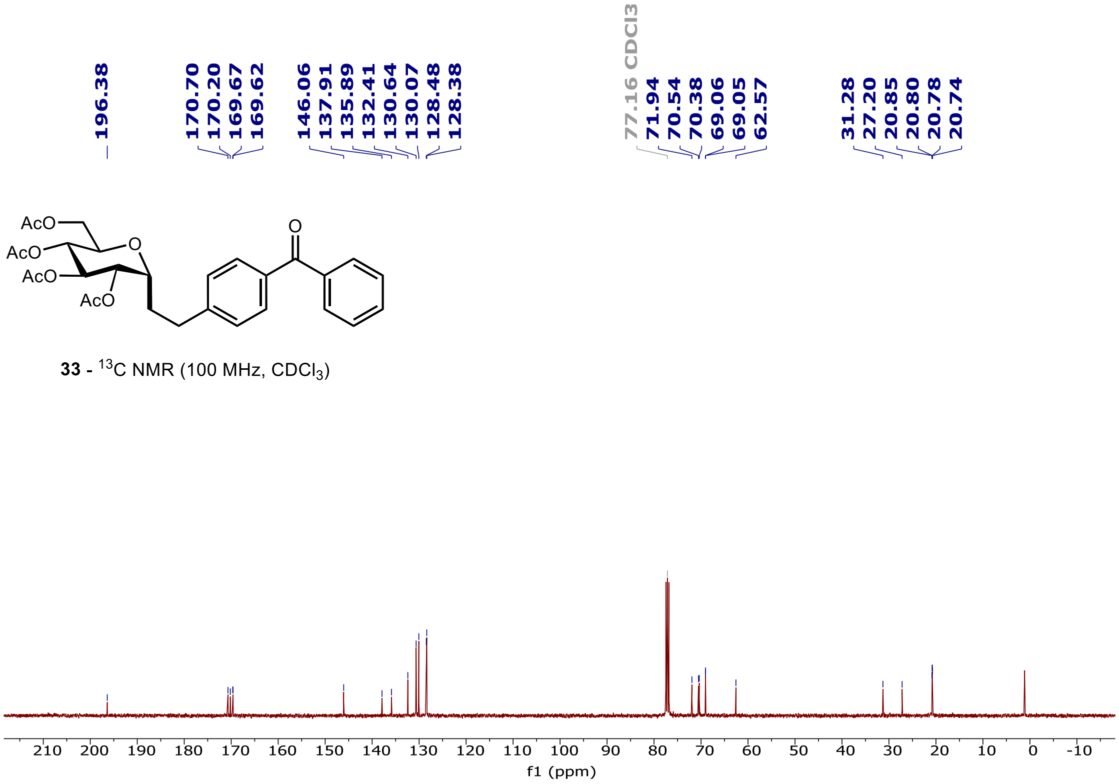

33 - ^{13}C NMR (100 MHz, $CDCl_3$)

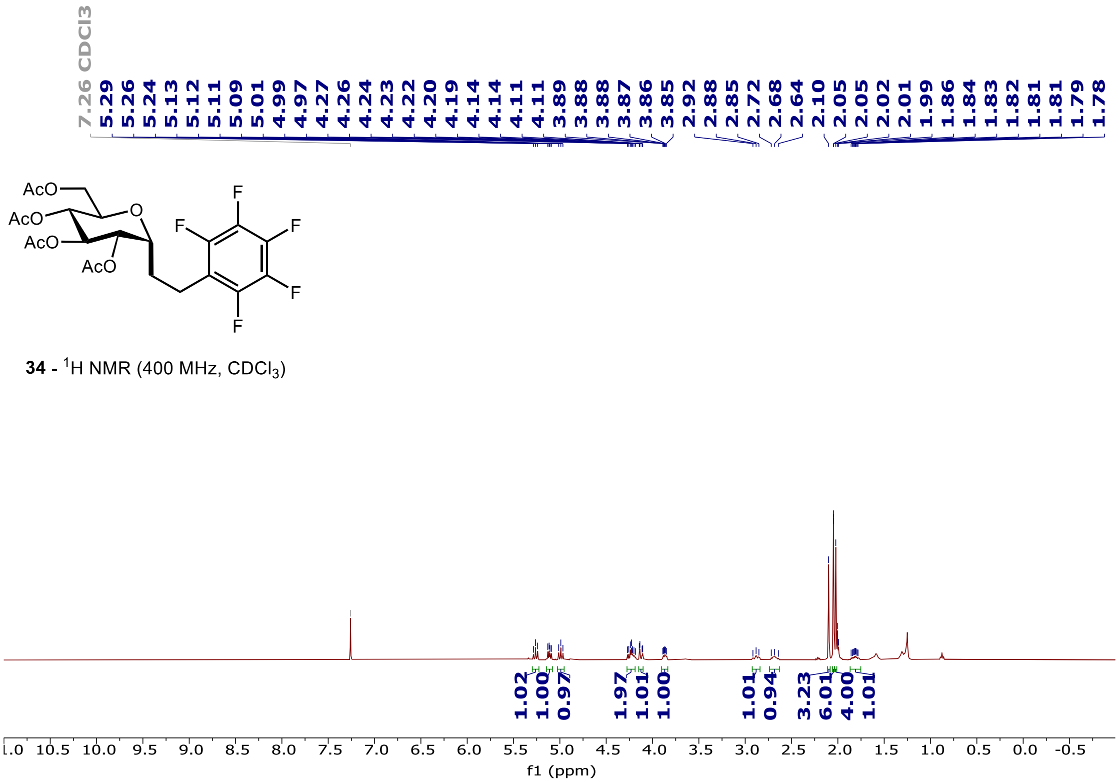

34 - ^{1}H NMR (400 MHz, $CDCl_3$)

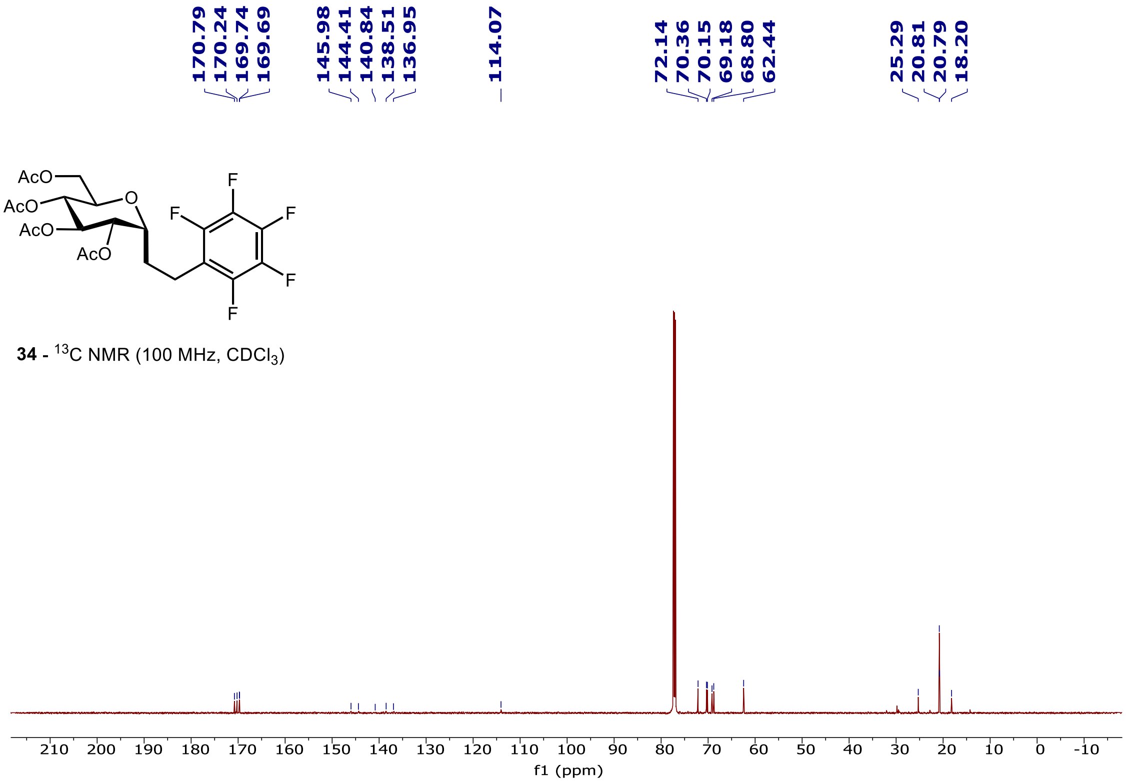

34 - ^{13}C NMR (100 MHz, $CDCl_3$)

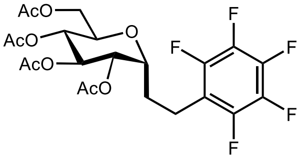

34 - ^{19}F NMR (376 MHz, $CDCl_3$)

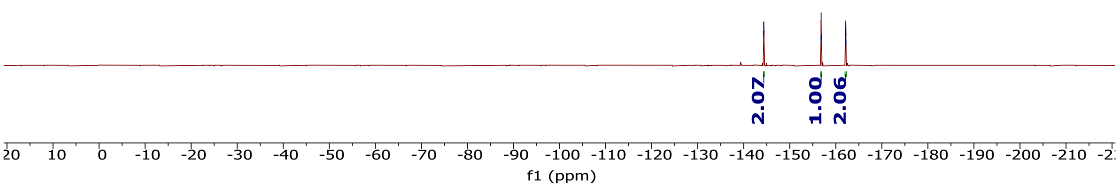

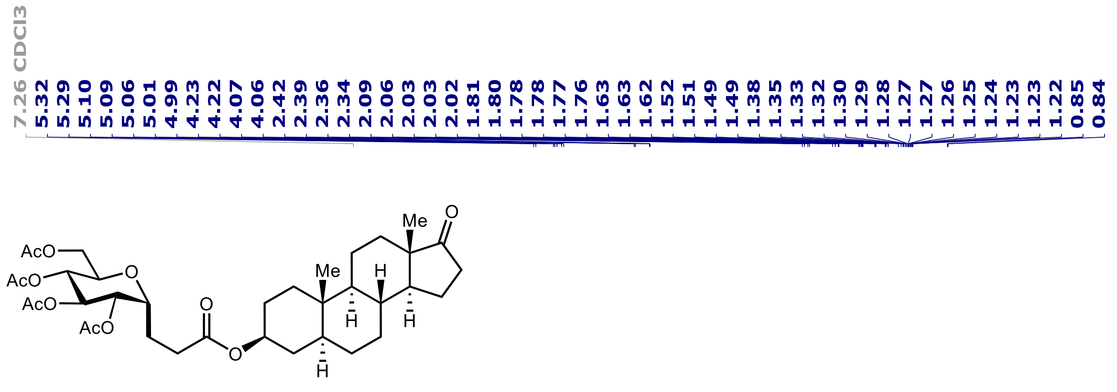

35 - ^{1}H NMR (400 MHz, $CDCl_3$)

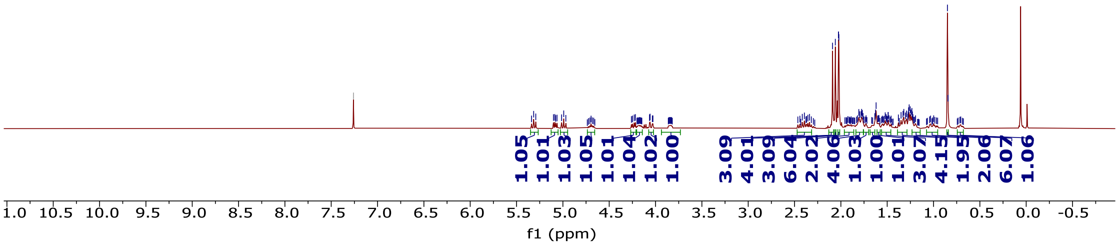

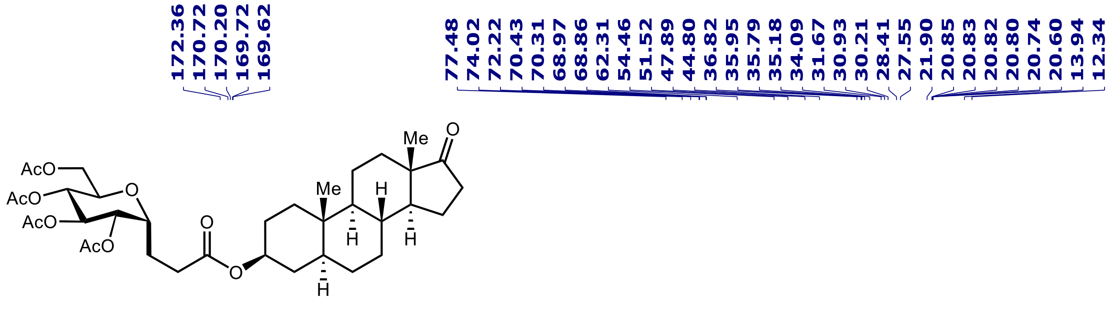

35 - ^{13}C NMR (100 MHz, $CDCl_3$)

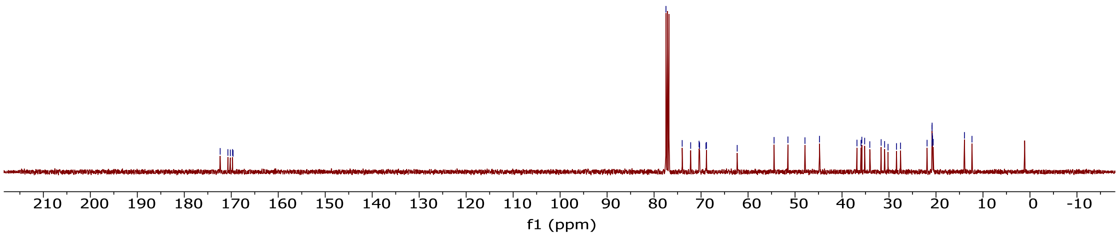

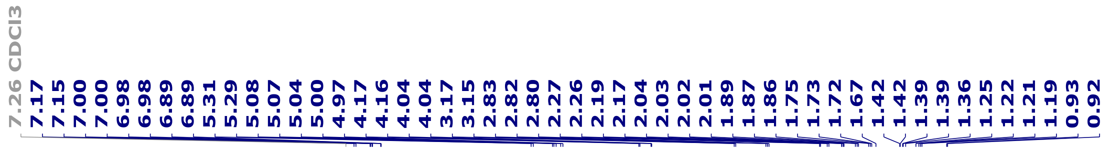

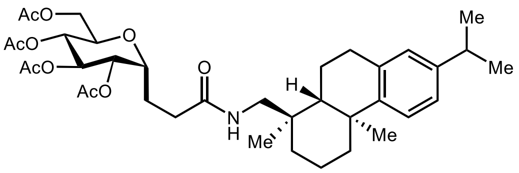

36 - ^{1}H NMR (400 MHz, $CDCl_3$)

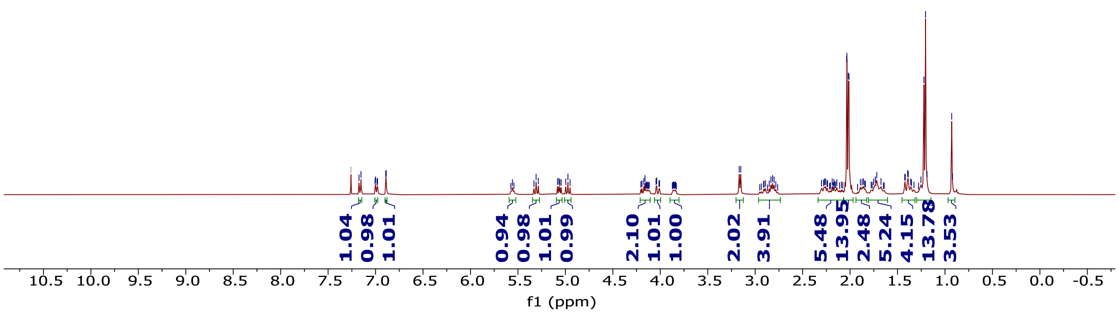

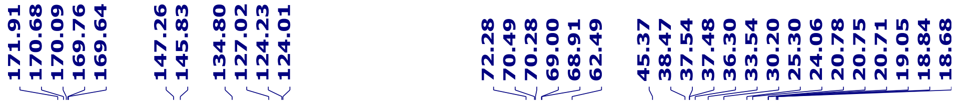

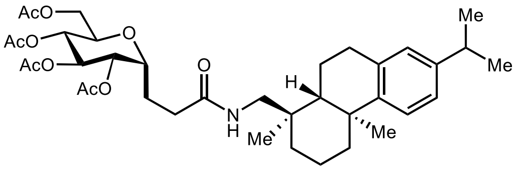

36 - ^{13}C NMR (100 MHz, $CDCl_3$)

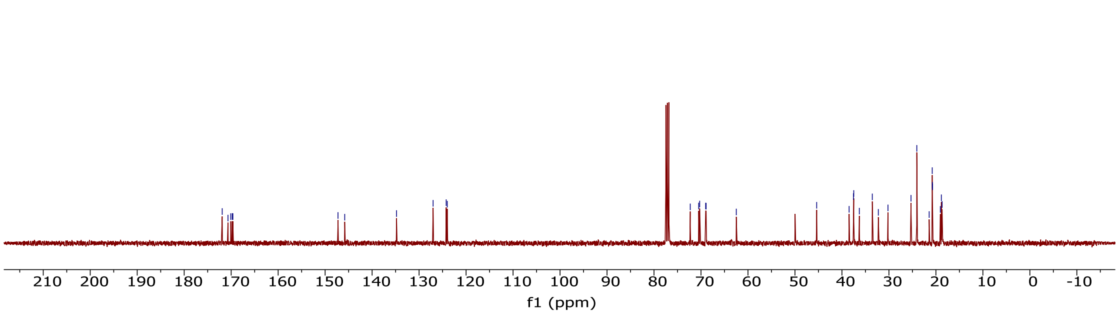

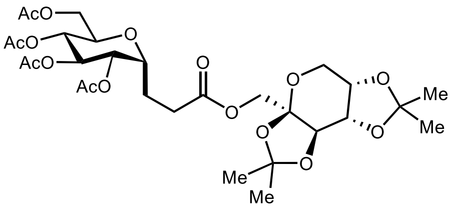

37 - ^{1}H NMR (400 MHz, $CDCl_3$)

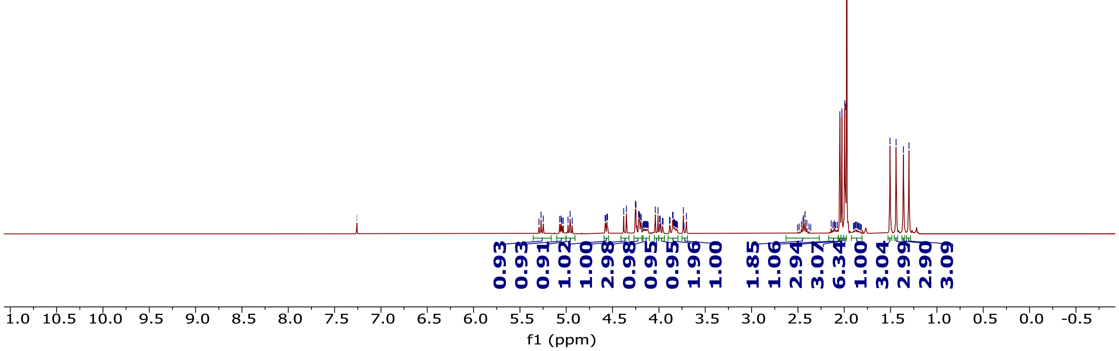

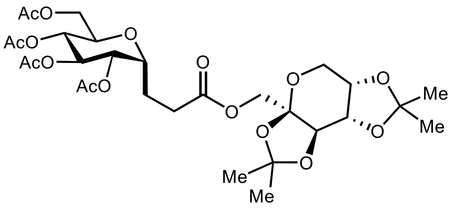

37 - ^{13}C NMR (100 MHz, $CDCl_3$)

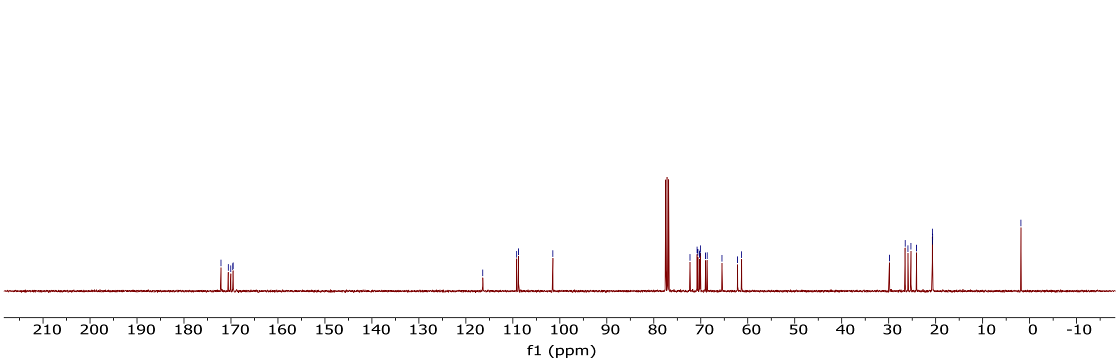

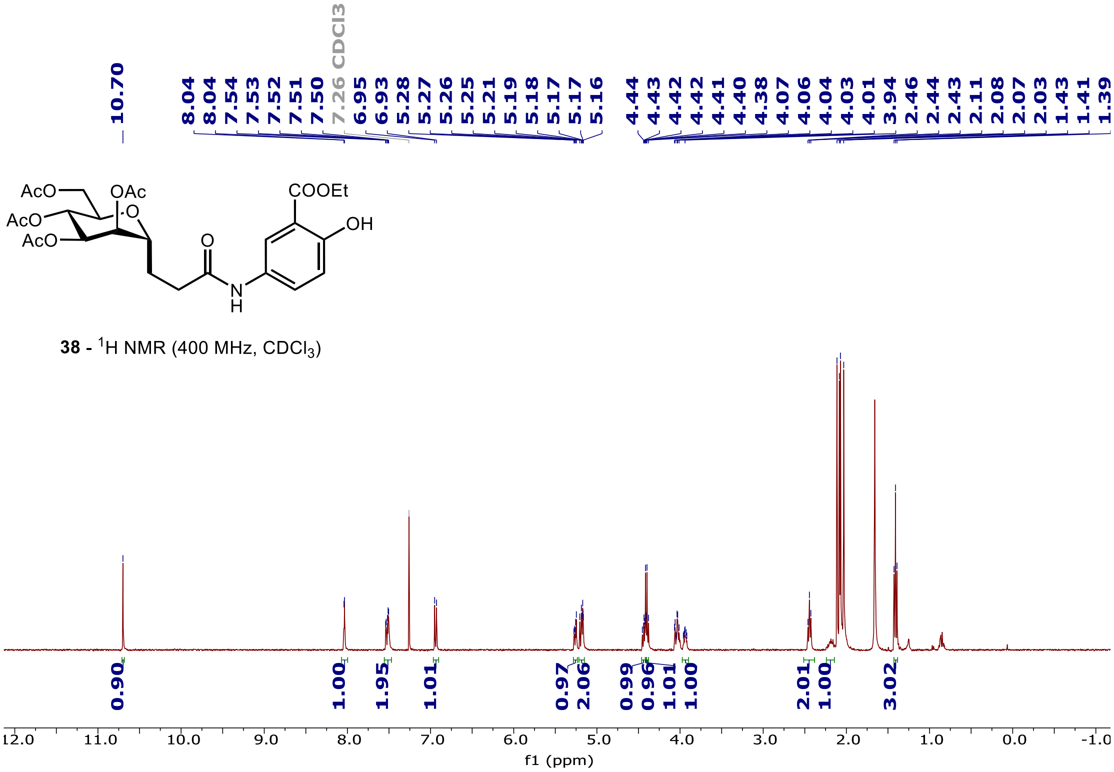

38 - ^{1}H NMR (400 MHz, $CDCl_3$)

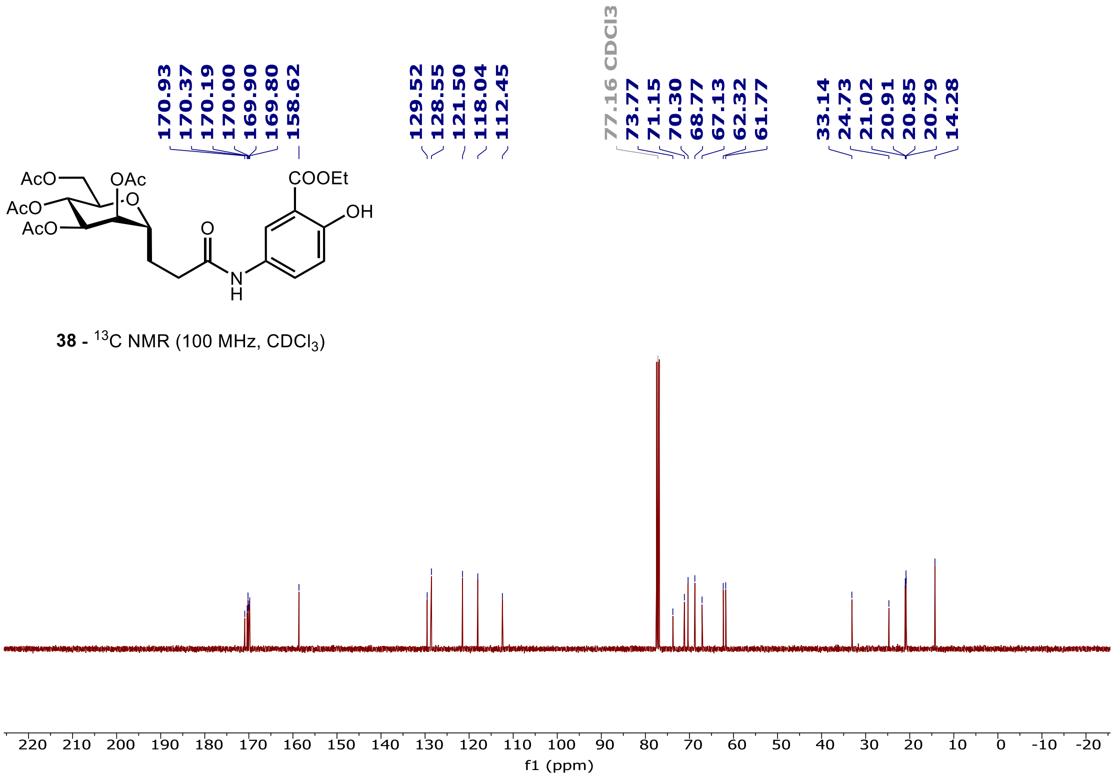

38 - ^{13}C NMR (100 MHz, $CDCl_3$)

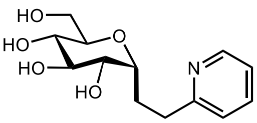

39 - ^{1}H NMR (400 MHz, CD_3OD)

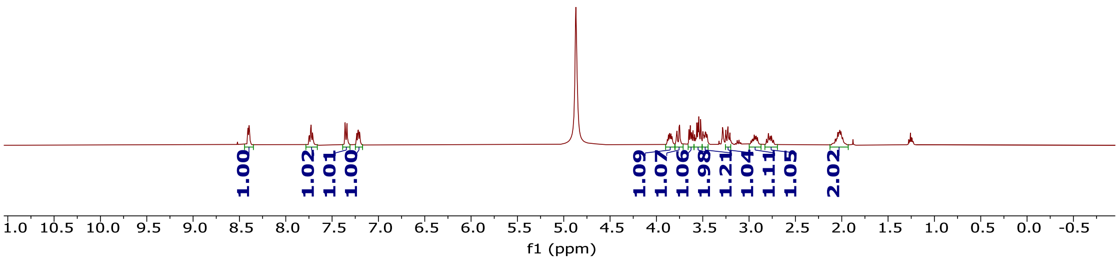

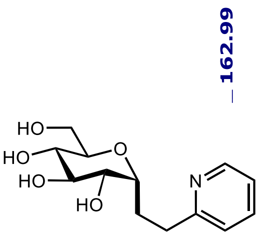

39 - ^{13}C NMR (100 MHz, CD_3OD)

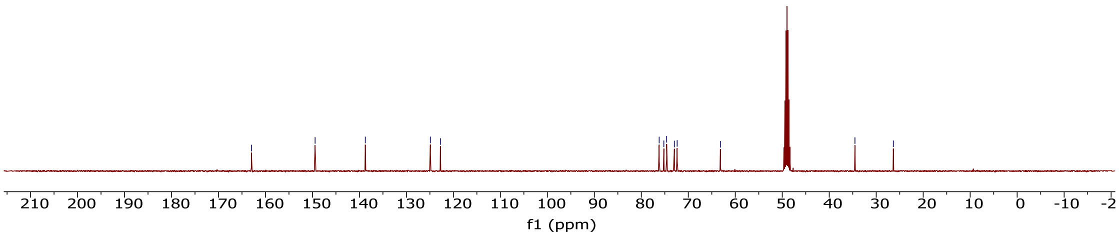

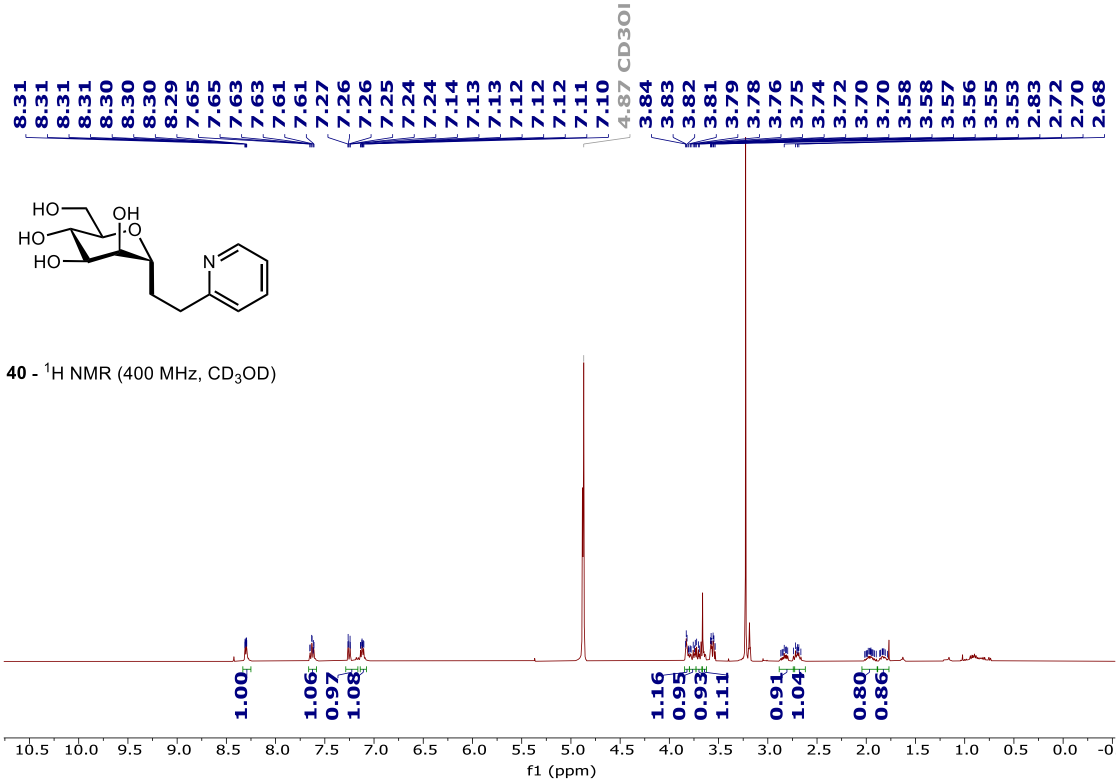

40 - ^{1}H NMR (400 MHz, CD_3OD)

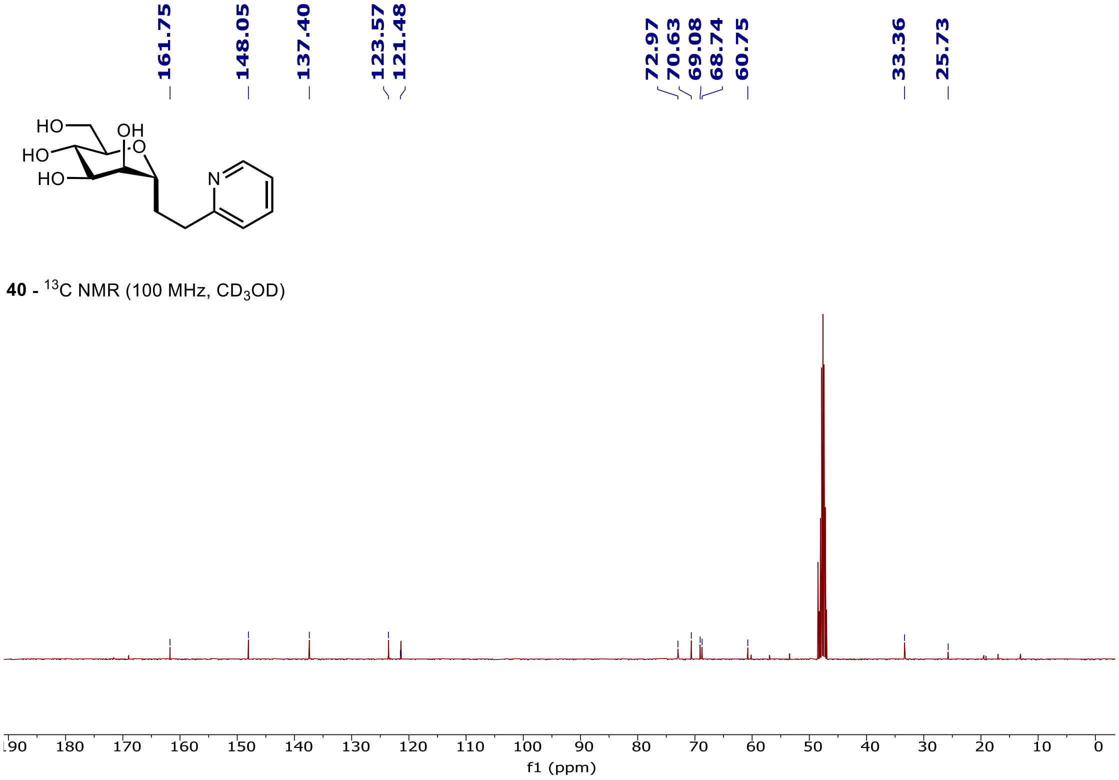

40 - ^{13}C NMR (100 MHz, CD_3OD)

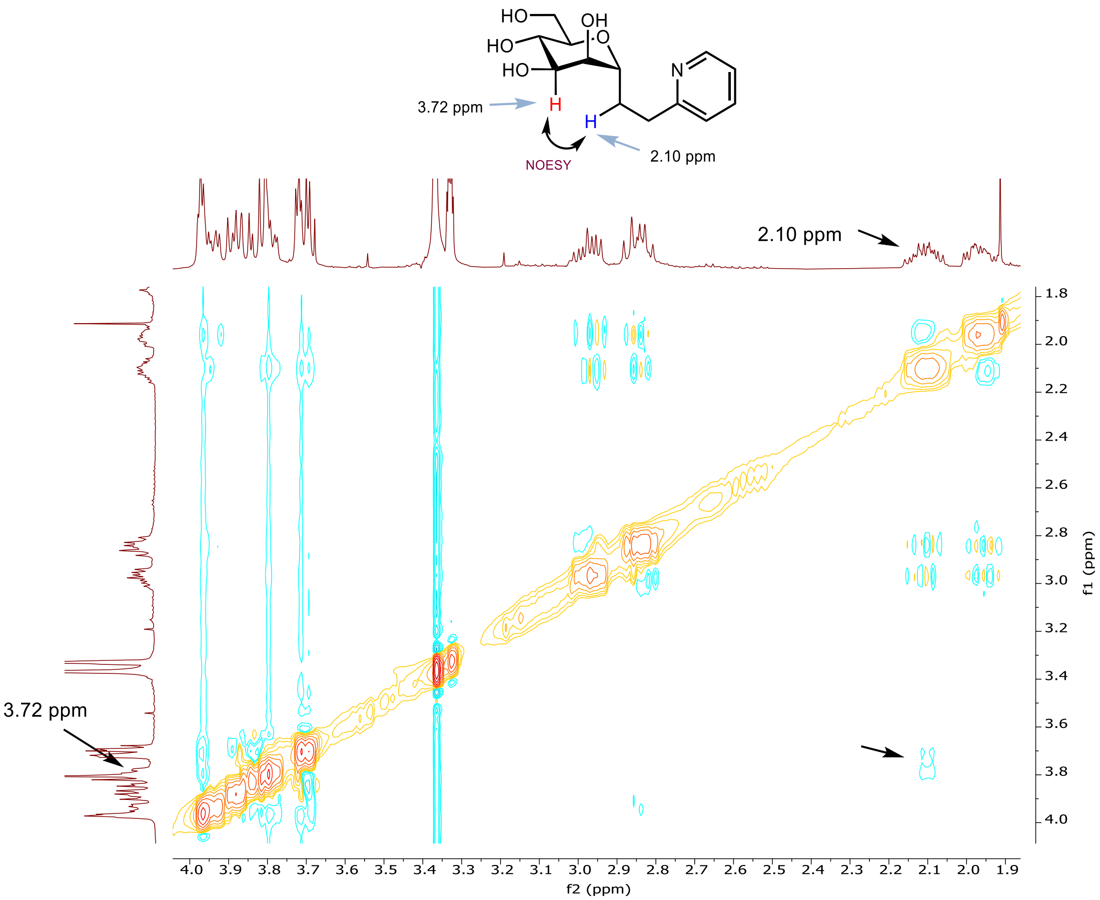
HO
OH
O
HO
HO
N
3.72 ppm
H
H
NOESY
2.10 ppm
2.10 ppm
3.72 ppm
f2 (ppm)
f1 (ppm)

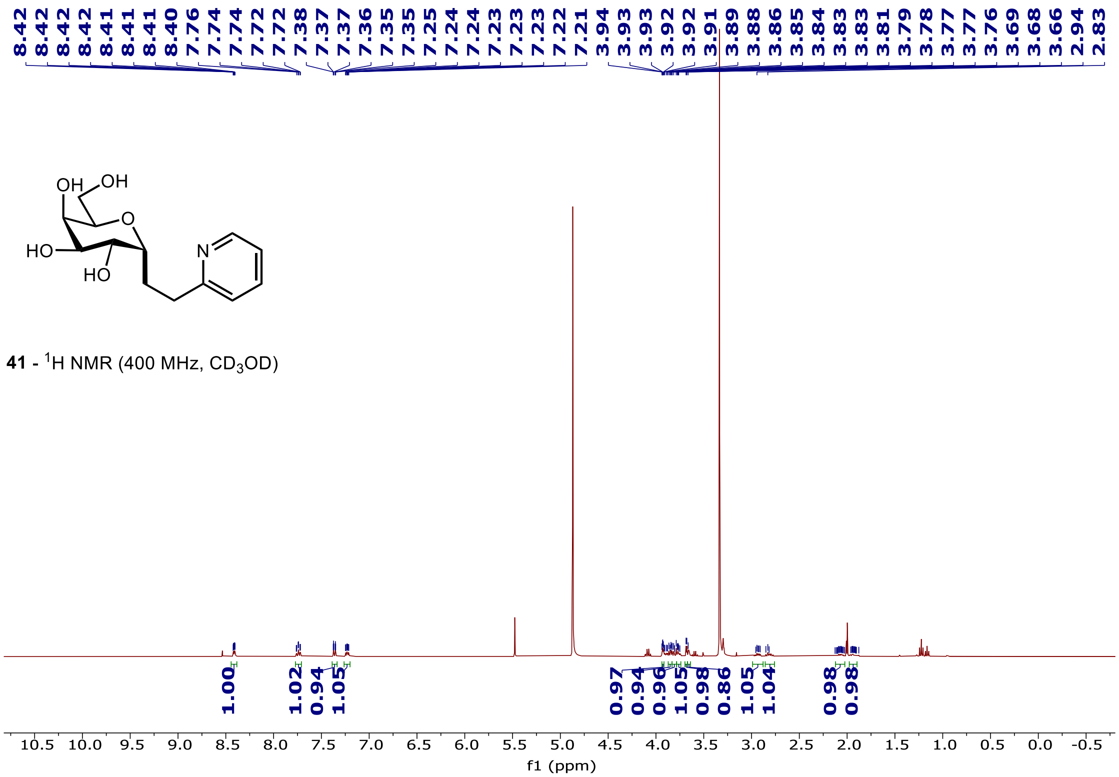

41 - ^{1}H NMR (400 MHz, CD_3OD)

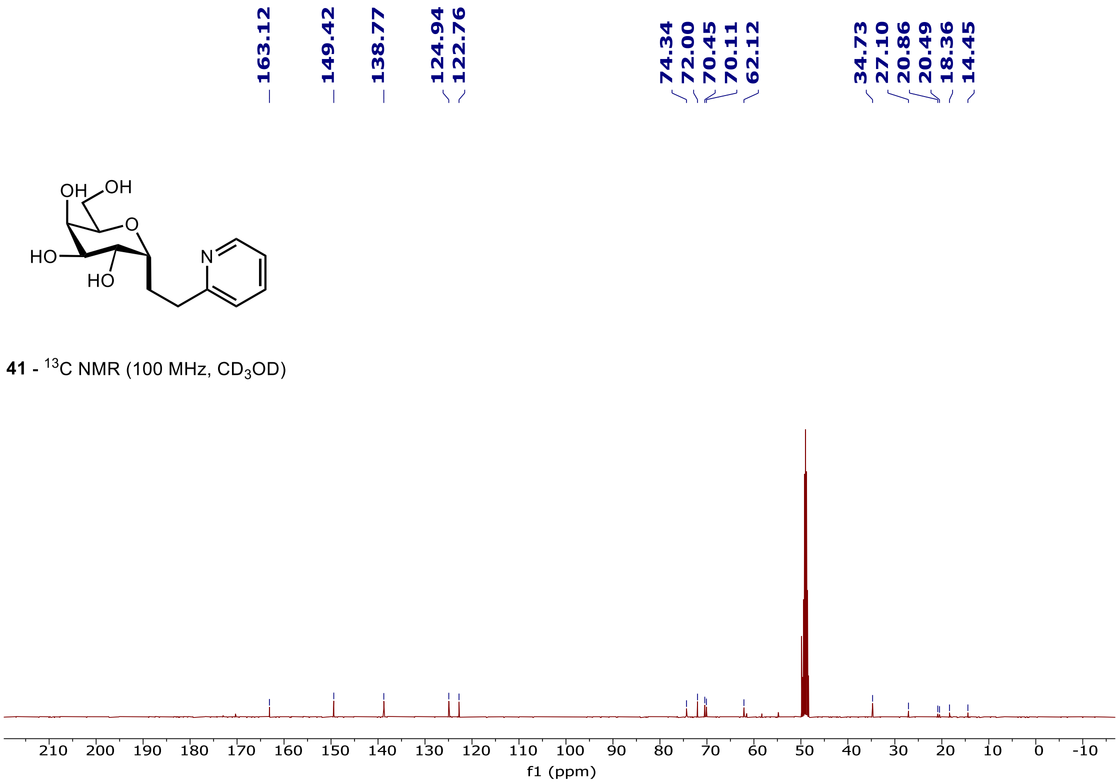

41 - ^{13}C NMR (100 MHz, CD_3OD)

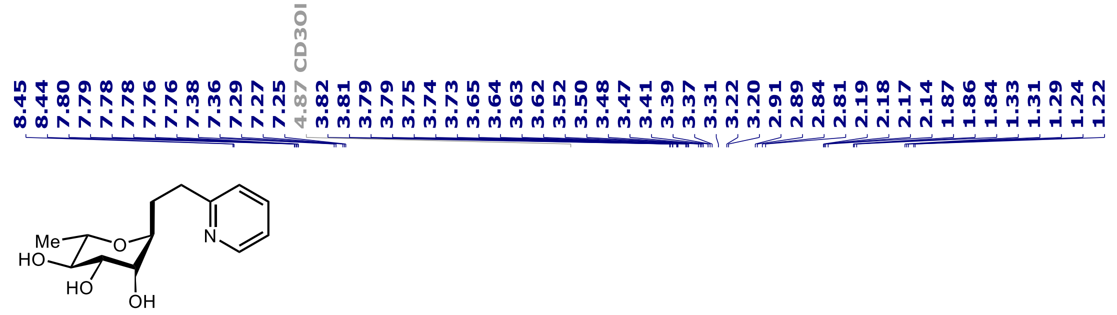

42 - ^{1}H NMR (400 MHz, CD_3OD)

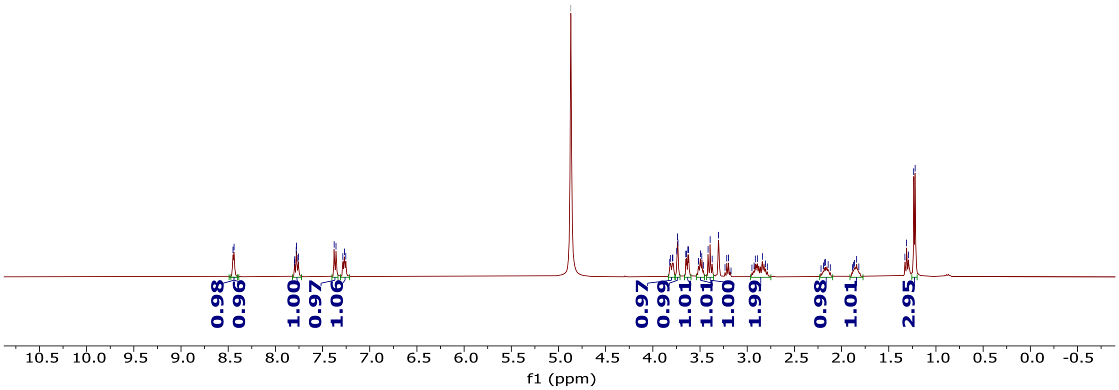

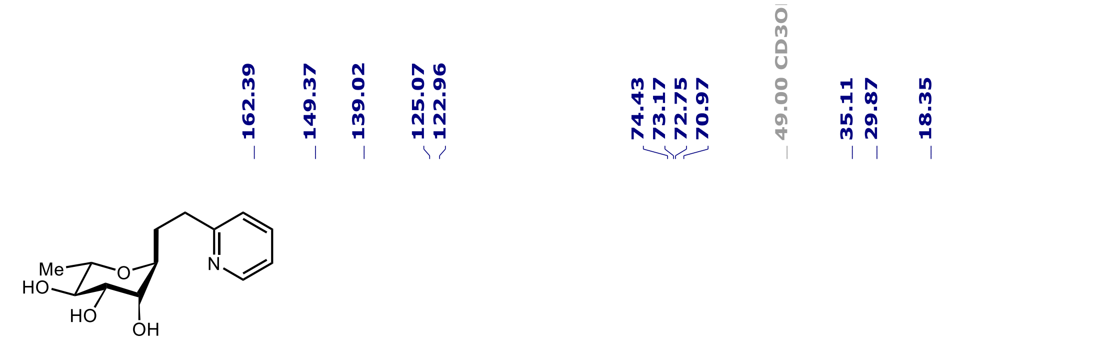

42 - ^{13}C NMR (100 MHz, CD_3OD)

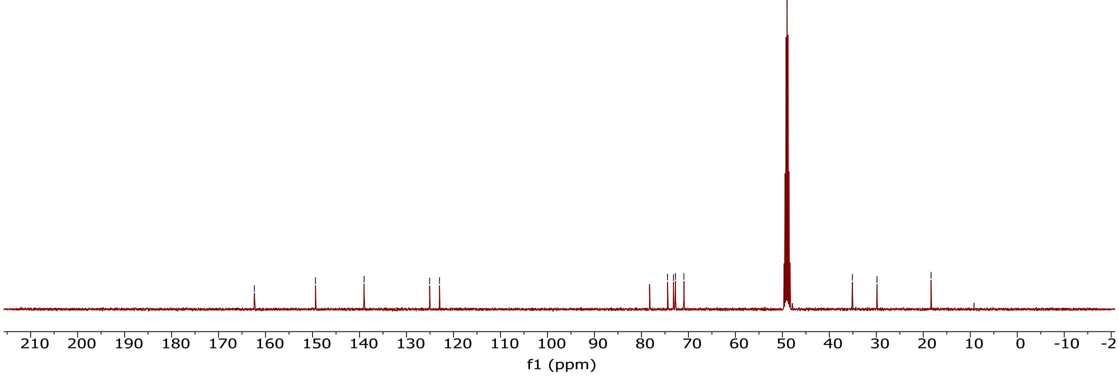

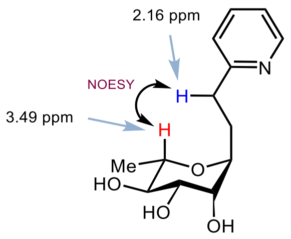
2.16 ppm
NOESY
3.49 ppm
H
H
Me
O
N
HO
HO
OH

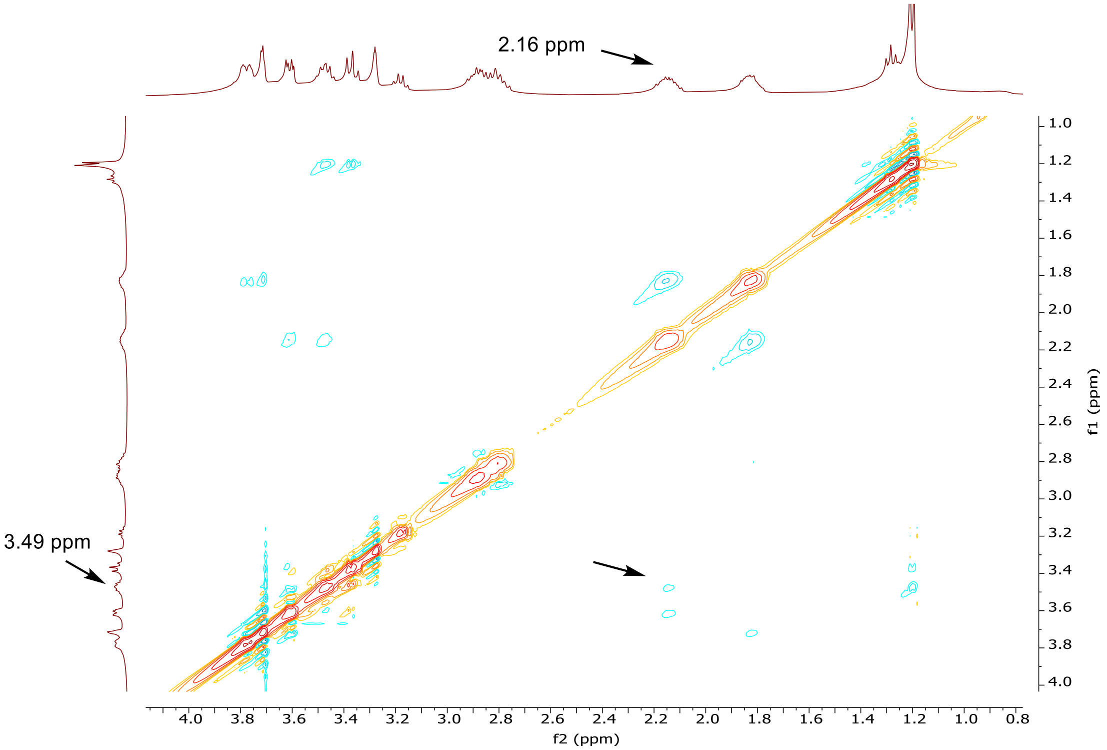
2.16 ppm
3.49 ppm
f2 (ppm)
f1 (ppm)

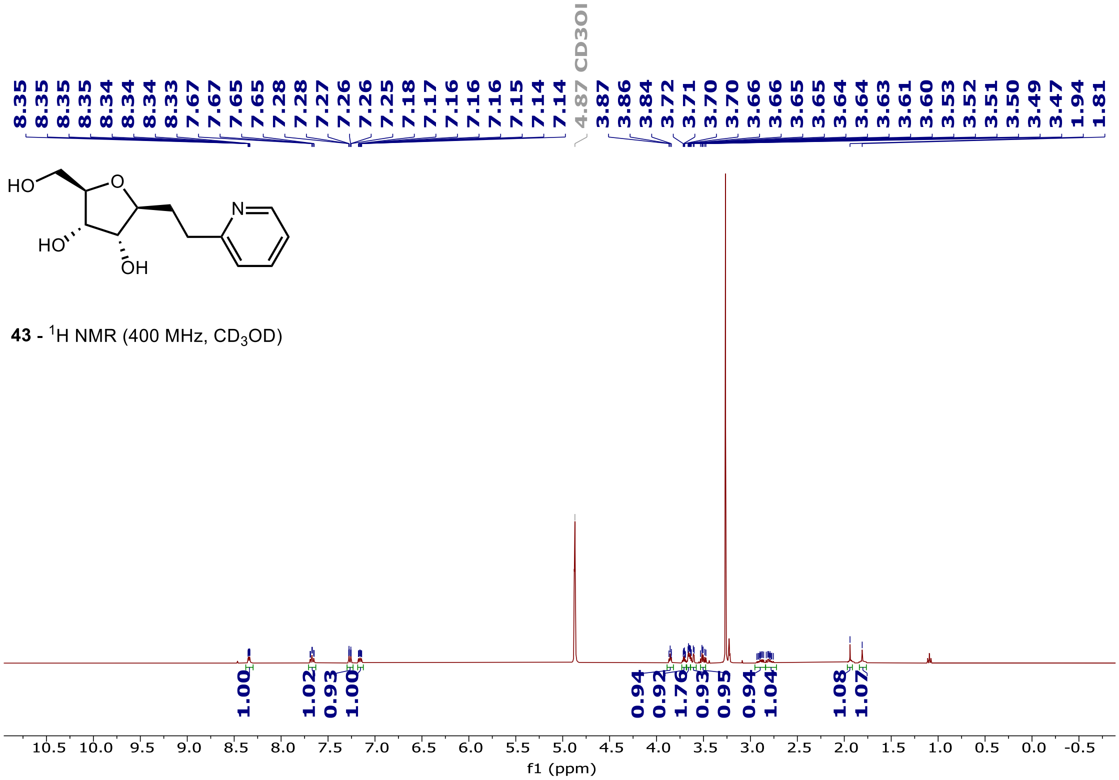

43 - ^{1}H NMR (400 MHz, CD_3OD)

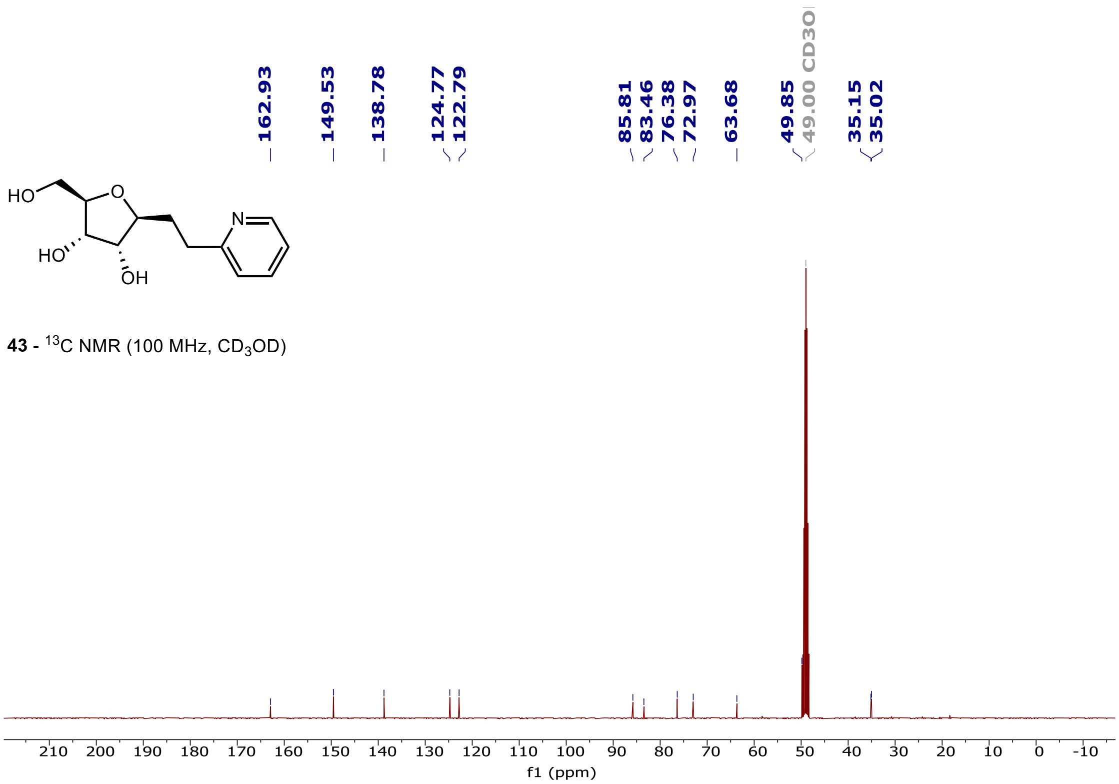

43 - ^{13}C NMR (100 MHz, CD_3OD)

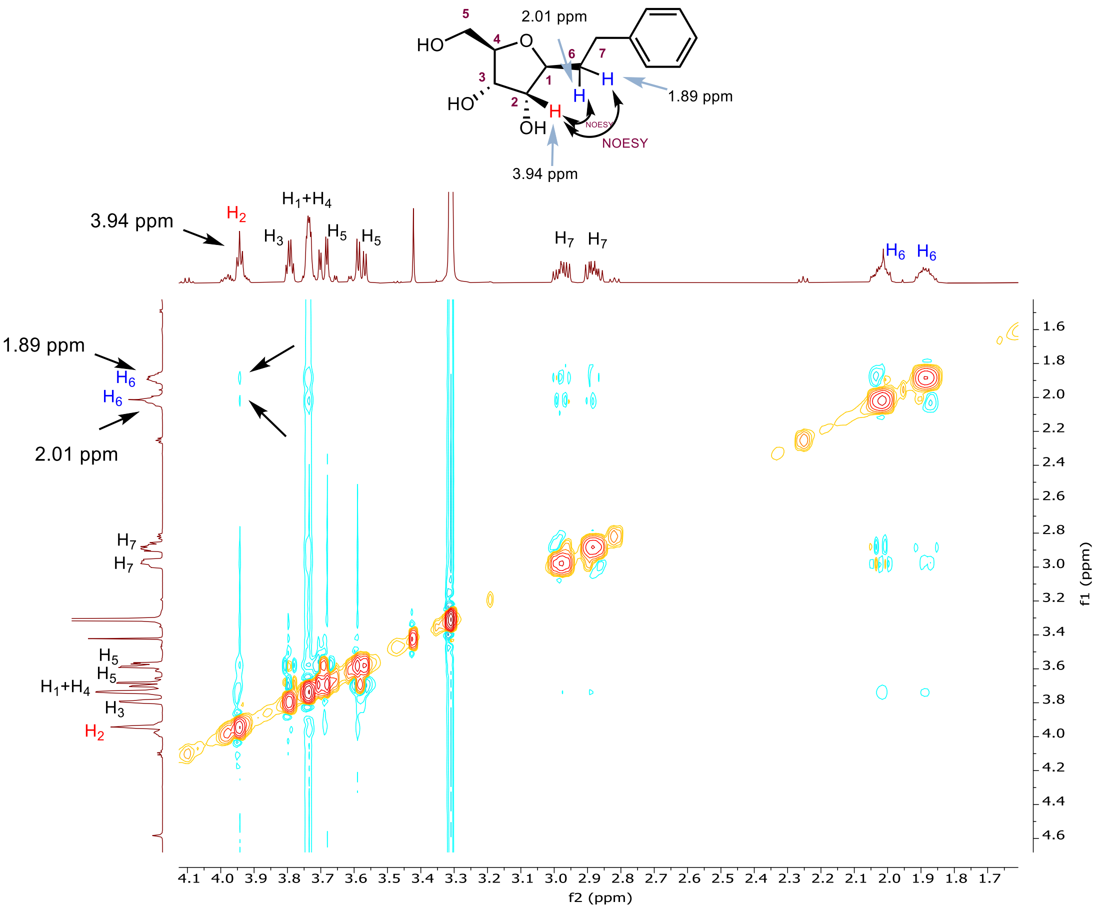
2.01 ppm
1.89 ppm
3.94 ppm
NOESY
3.94 ppm
H2
H1+H4
H3
H5
H5
H7
H7
H6
H6
1.89 ppm
H6
H6
2.01 ppm
H7
H7
H5
H5
H1+H4
H3
H2
f2 (ppm)
f1 (ppm)

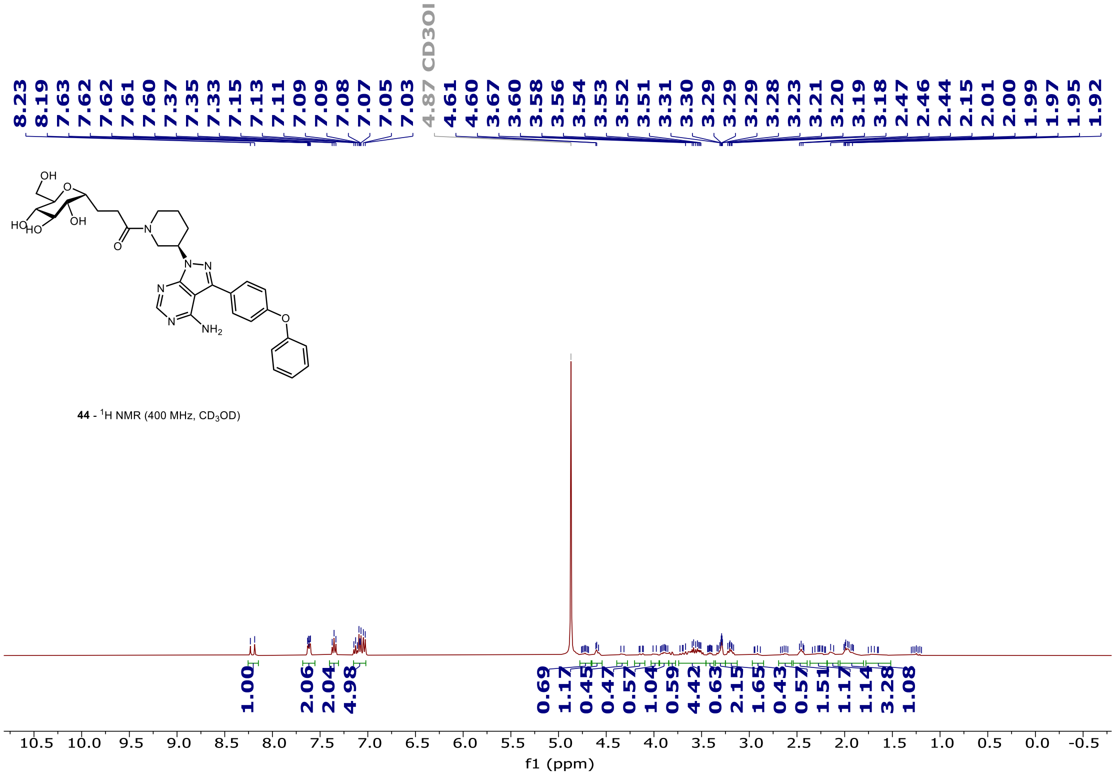

44 - ^{1}H NMR (400 MHz, CD_3OD)

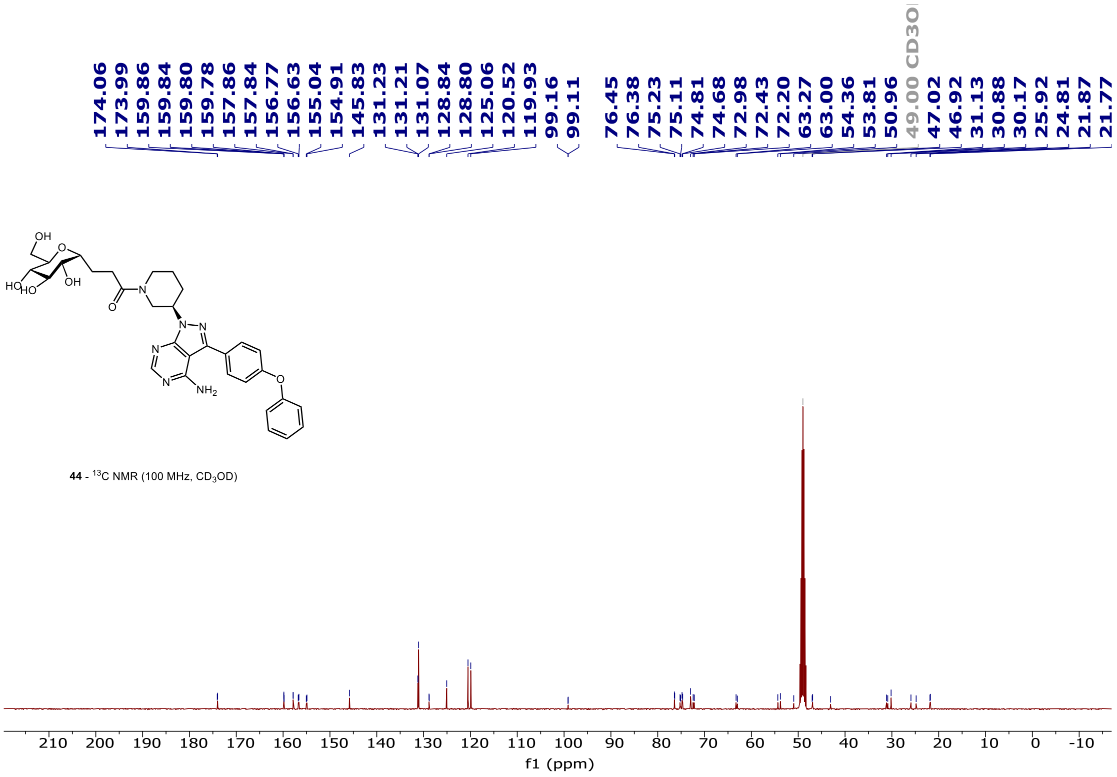

44 - ^{13}C NMR (100 MHz, CD_3OD)

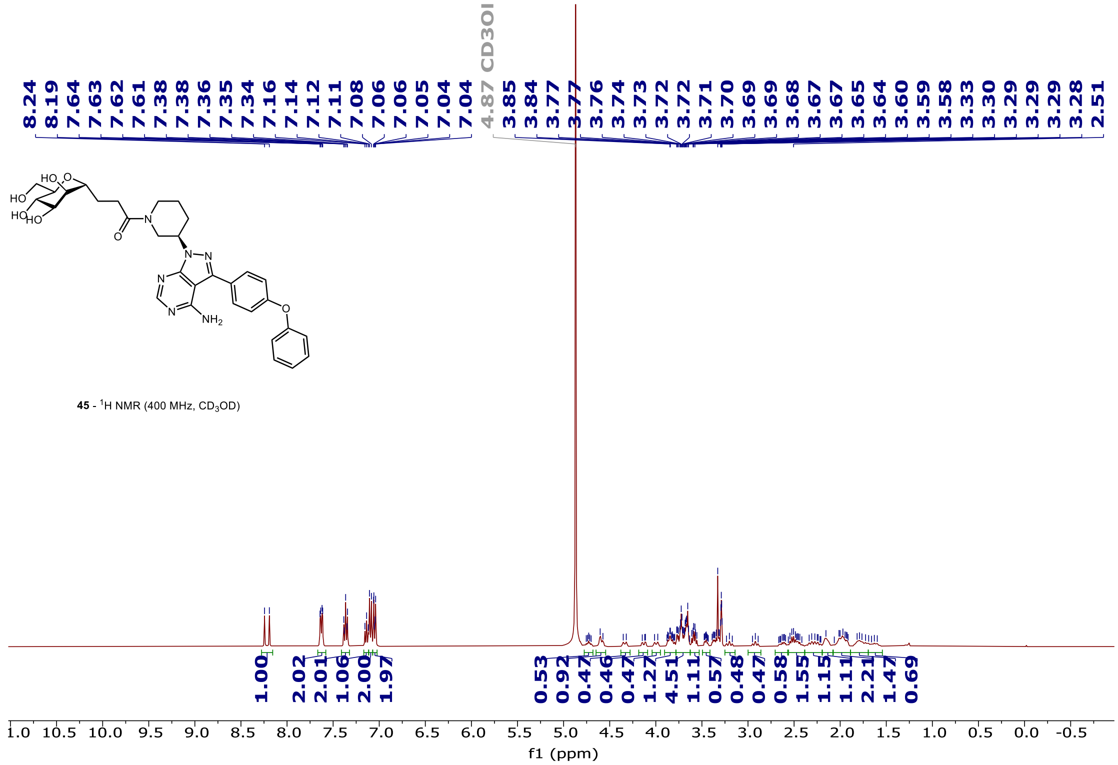

45 - ^{1}H NMR (400 MHz, CD_3OD)

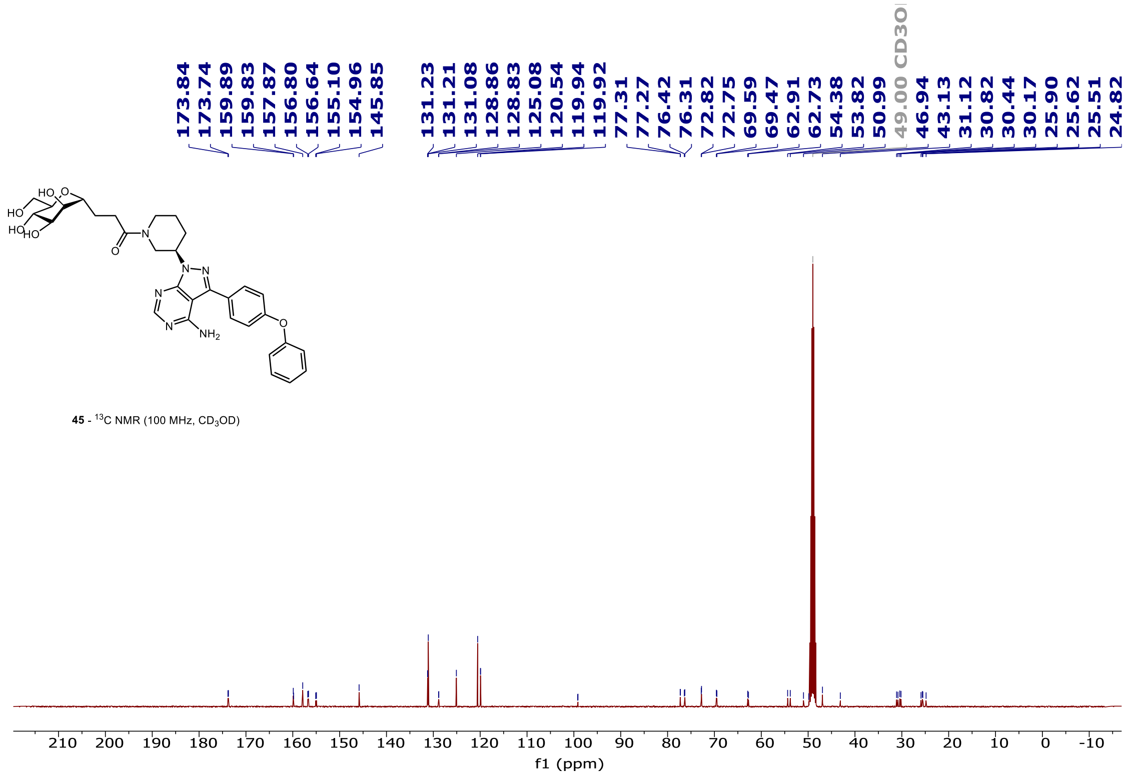

45 - ^{13}C NMR (100 MHz, CD_3OD)

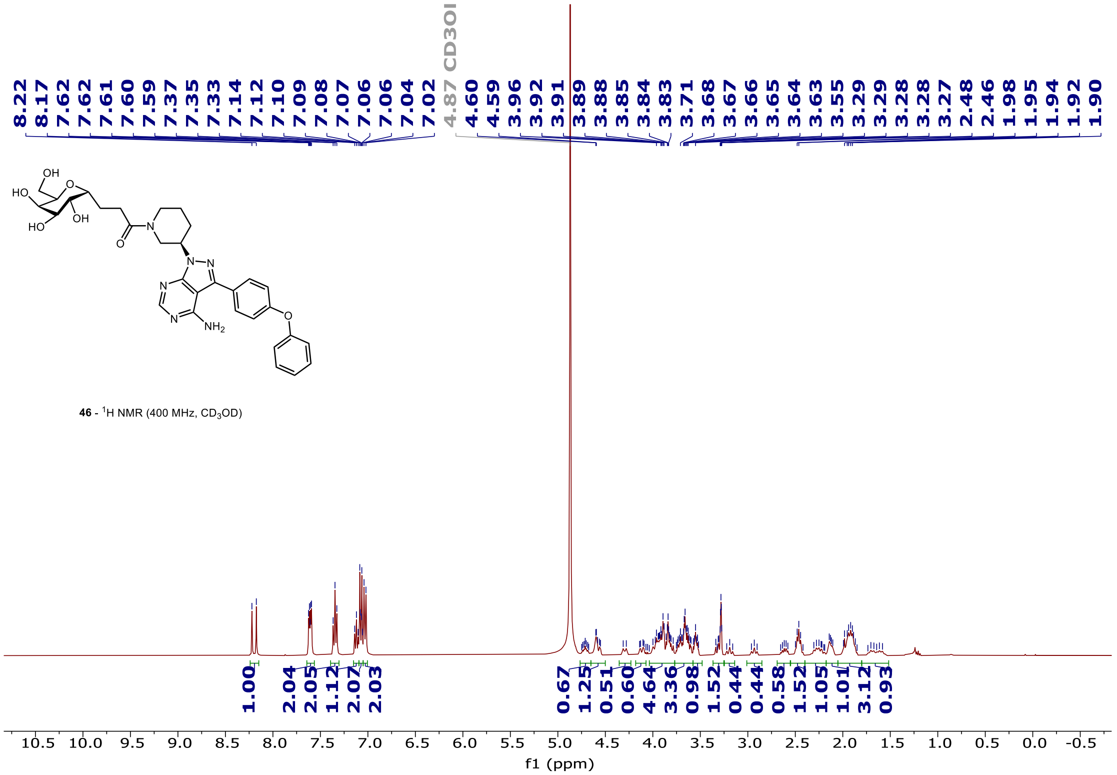

46 - ^{1}H NMR (400 MHz, CD_3OD)

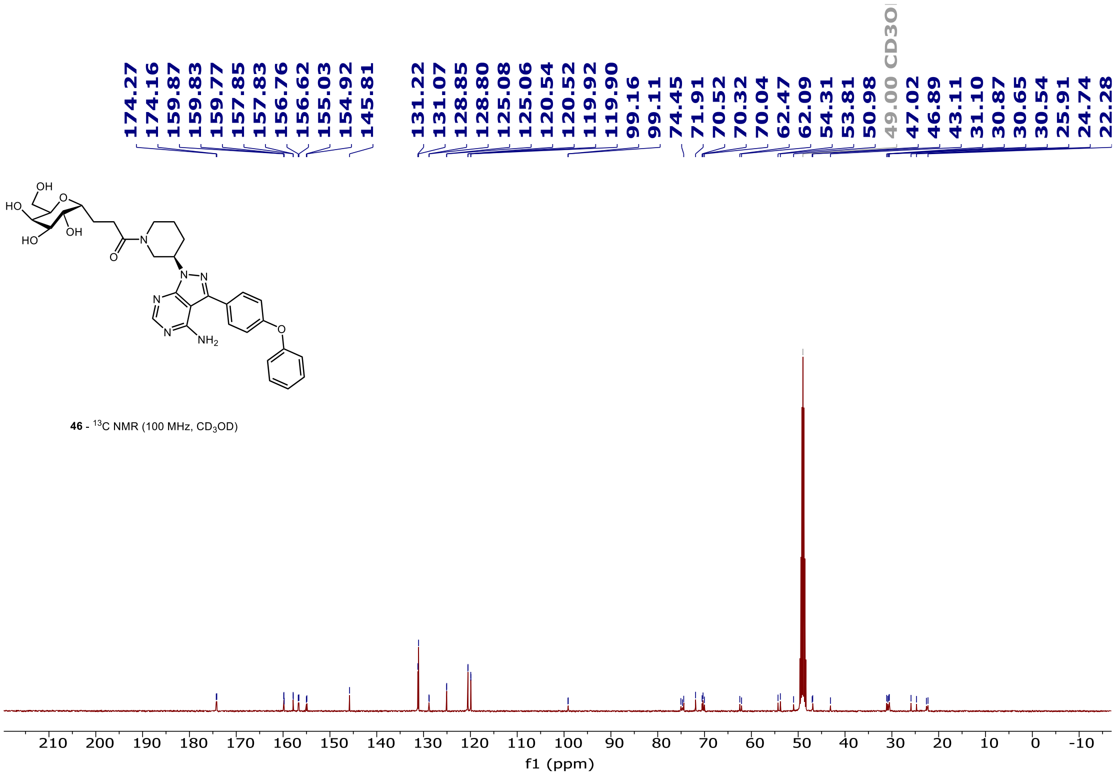

46 - ^{13}C NMR (100 MHz, CD_3OD)

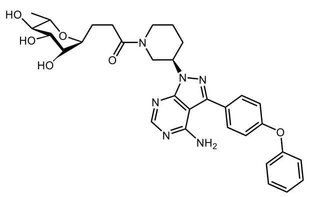

47 - ^{1}H NMR (400 MHz, CD_3OD)

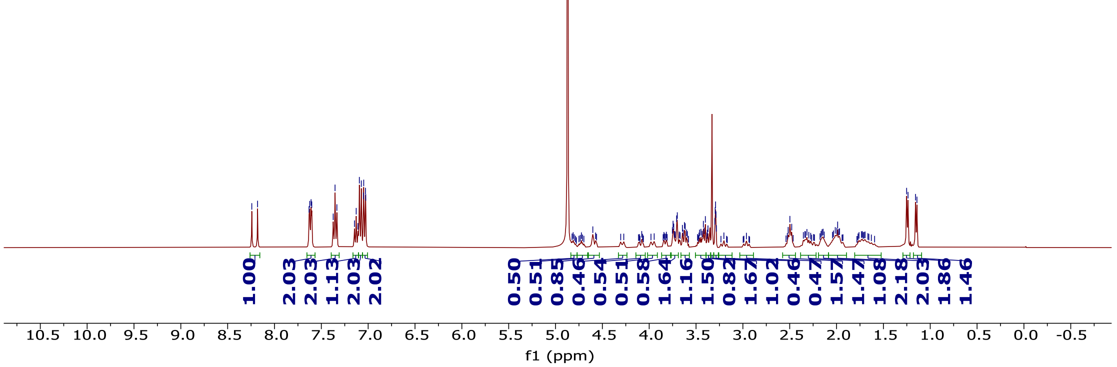

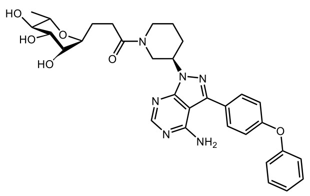

47 - ^{13}C NMR (100 MHz, CD_3OD)

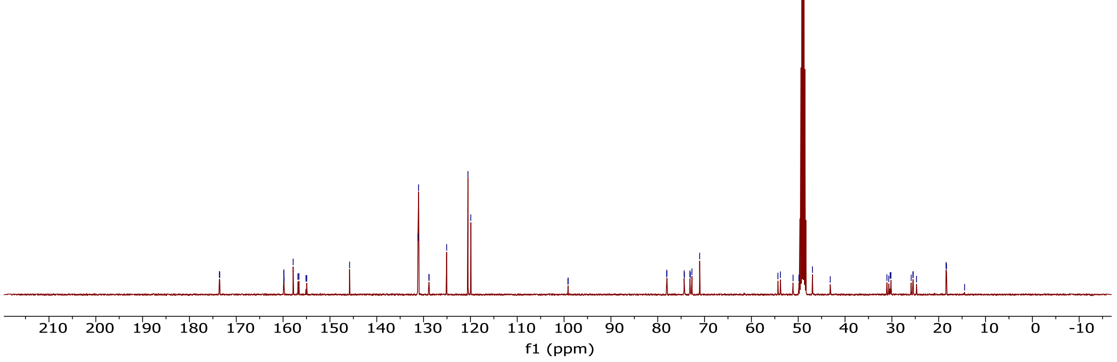

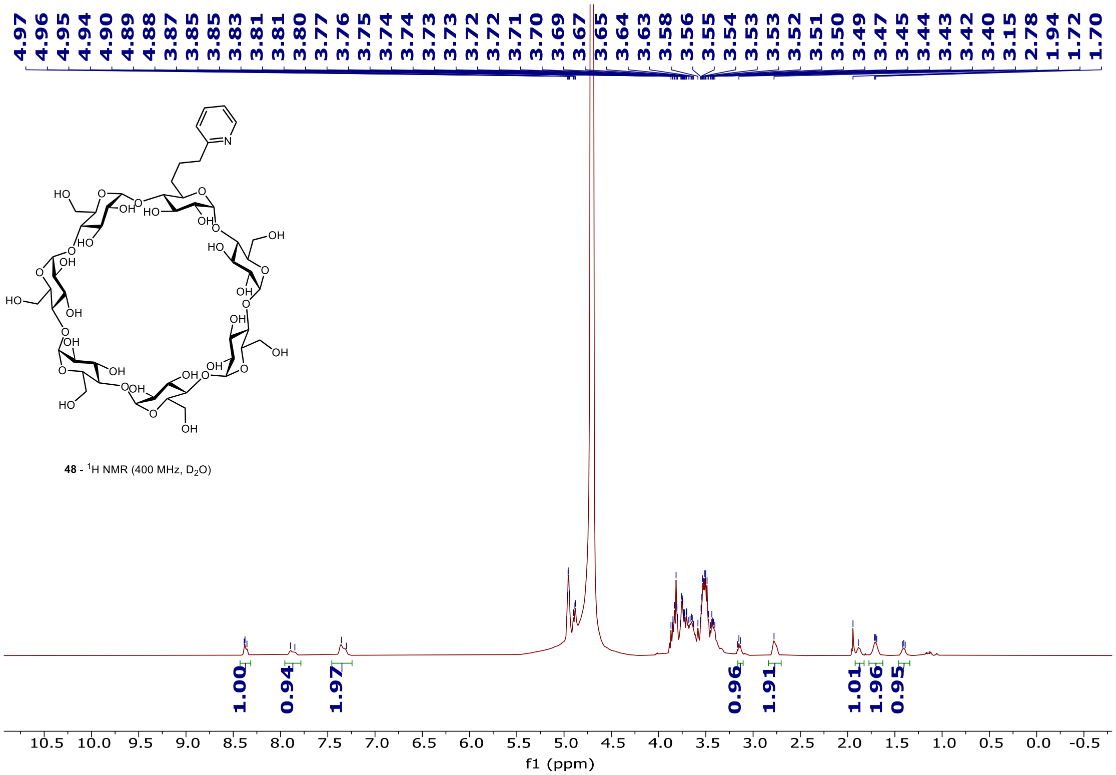

48 - ^{1}H NMR (400 MHz, D_2O)

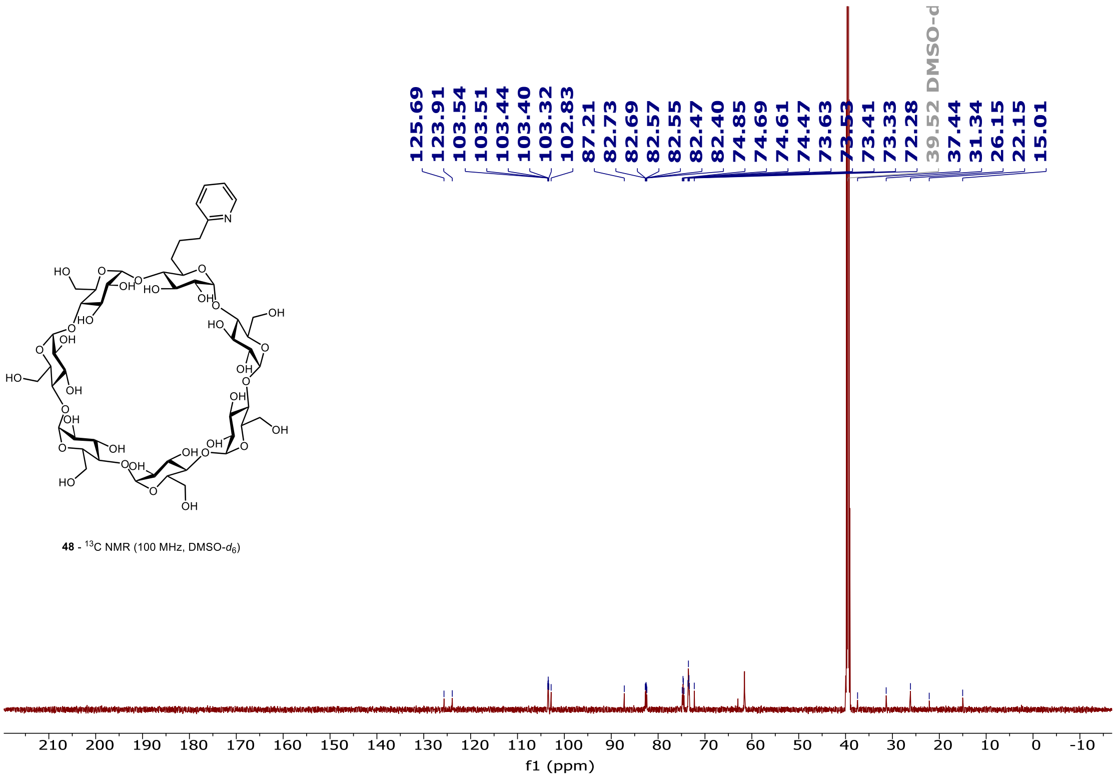

48 - ^{13}C NMR (100 MHz, DMSO-d_6)

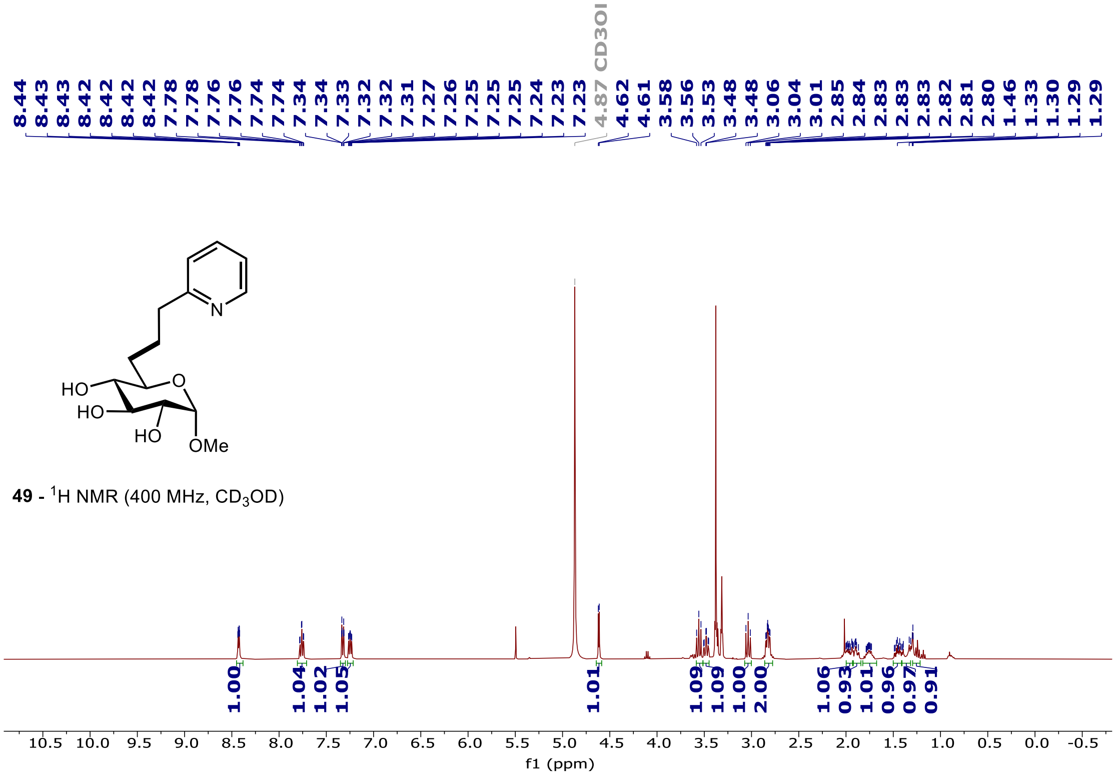

49 - ¹H NMR (400 MHz, CD_3OD)

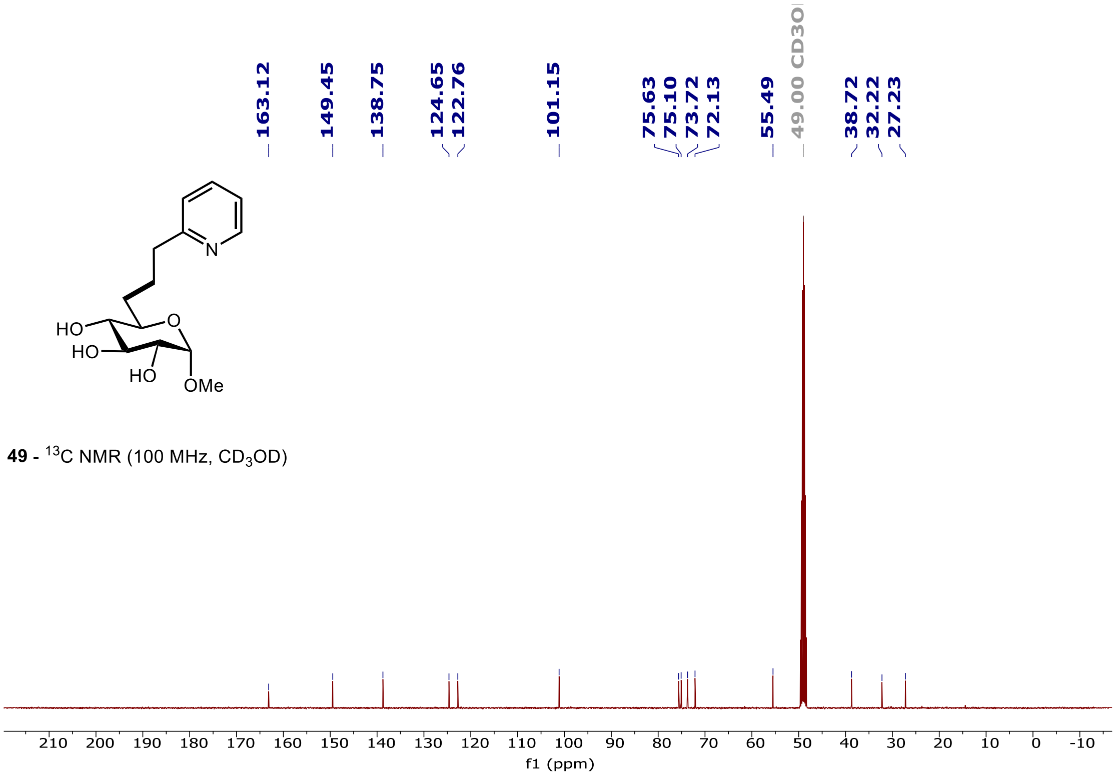

49 - ¹³C NMR (100 MHz, CD_3OD)

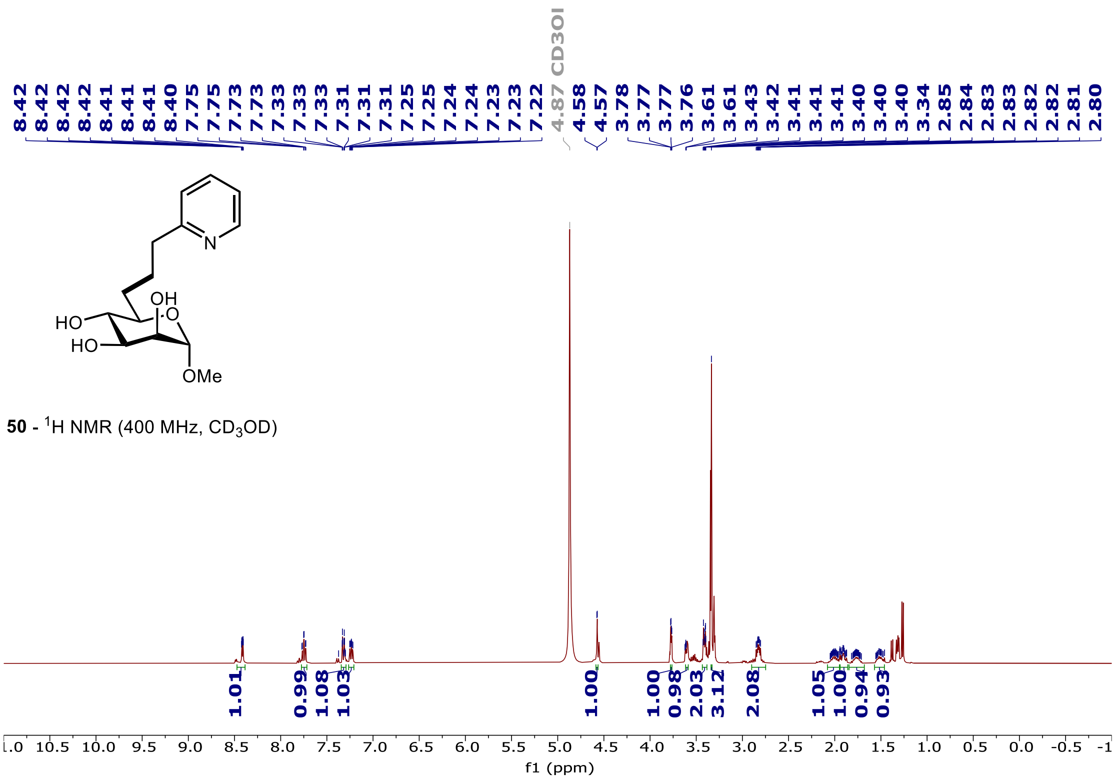

50 - ^{1}H NMR (400 MHz, CD_3OD)

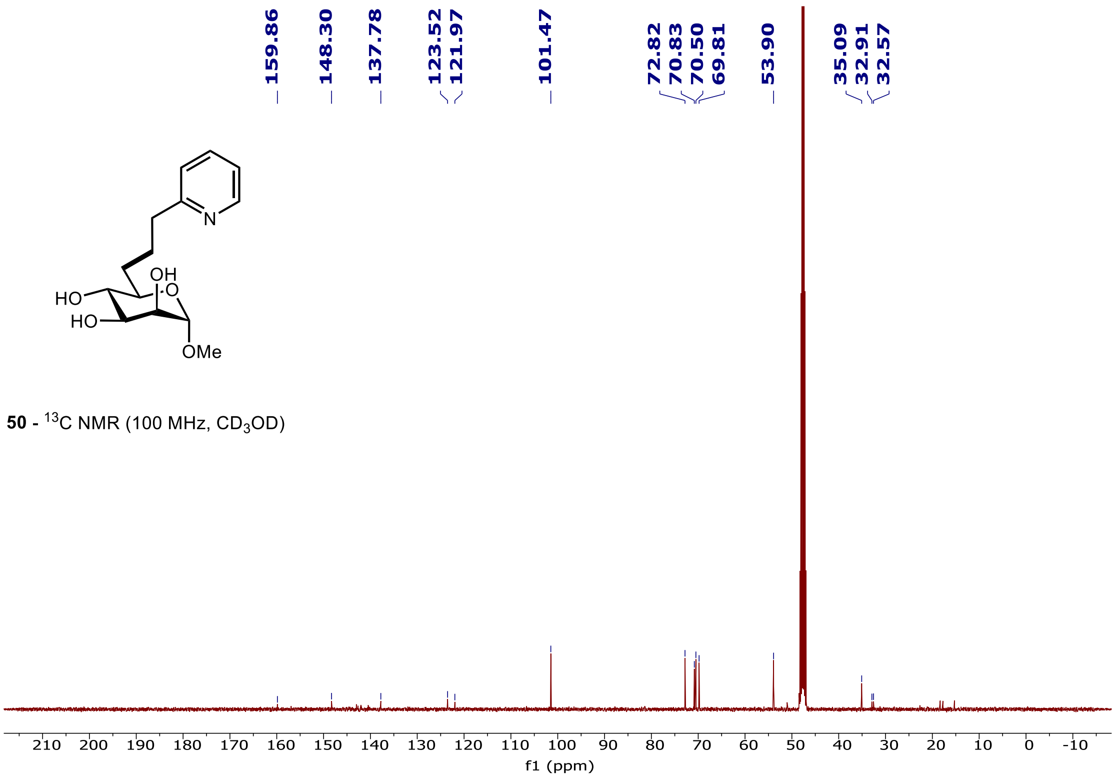

50 - ^{13}C NMR (100 MHz, CD_3OD)

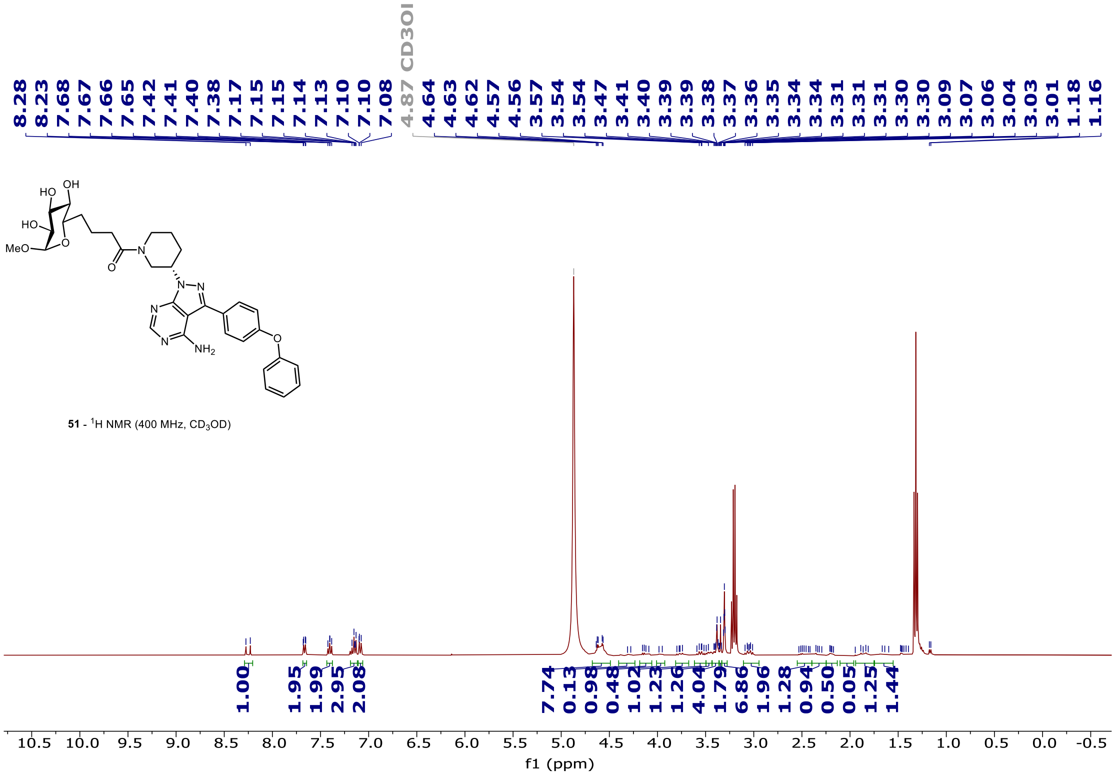

51 - ^{1}H NMR (400 MHz, CD_3OD)

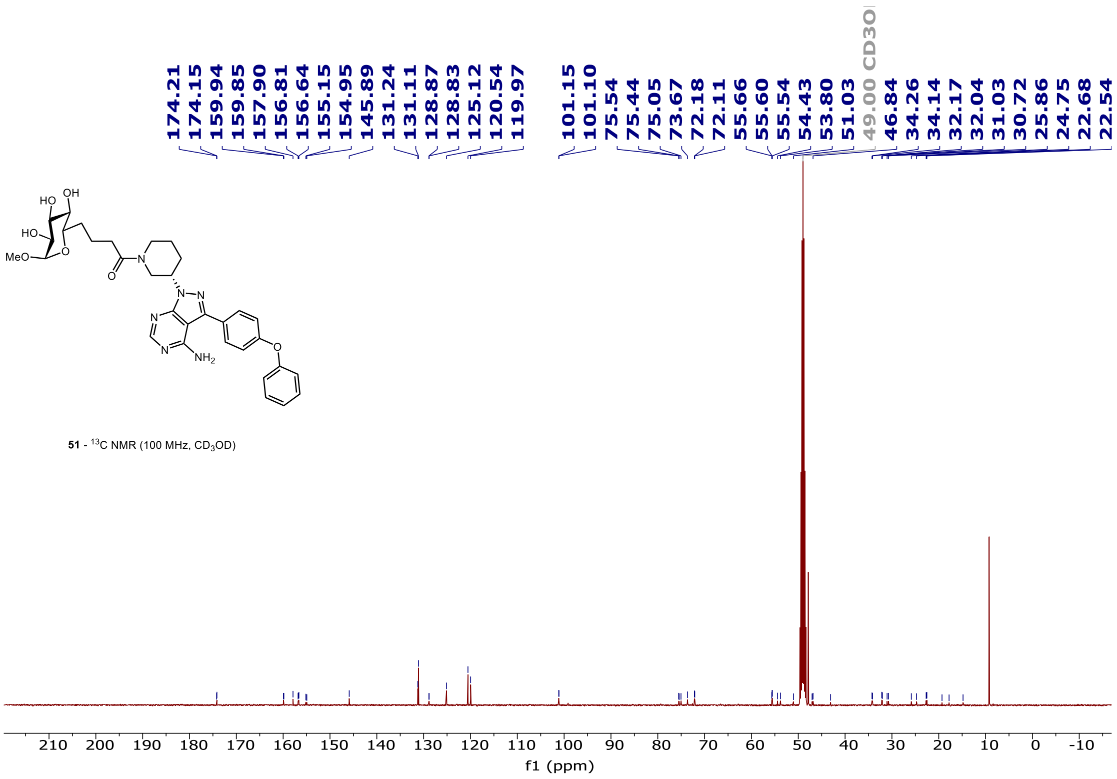

51 - ^{13}C NMR (100 MHz, CD_3OD)

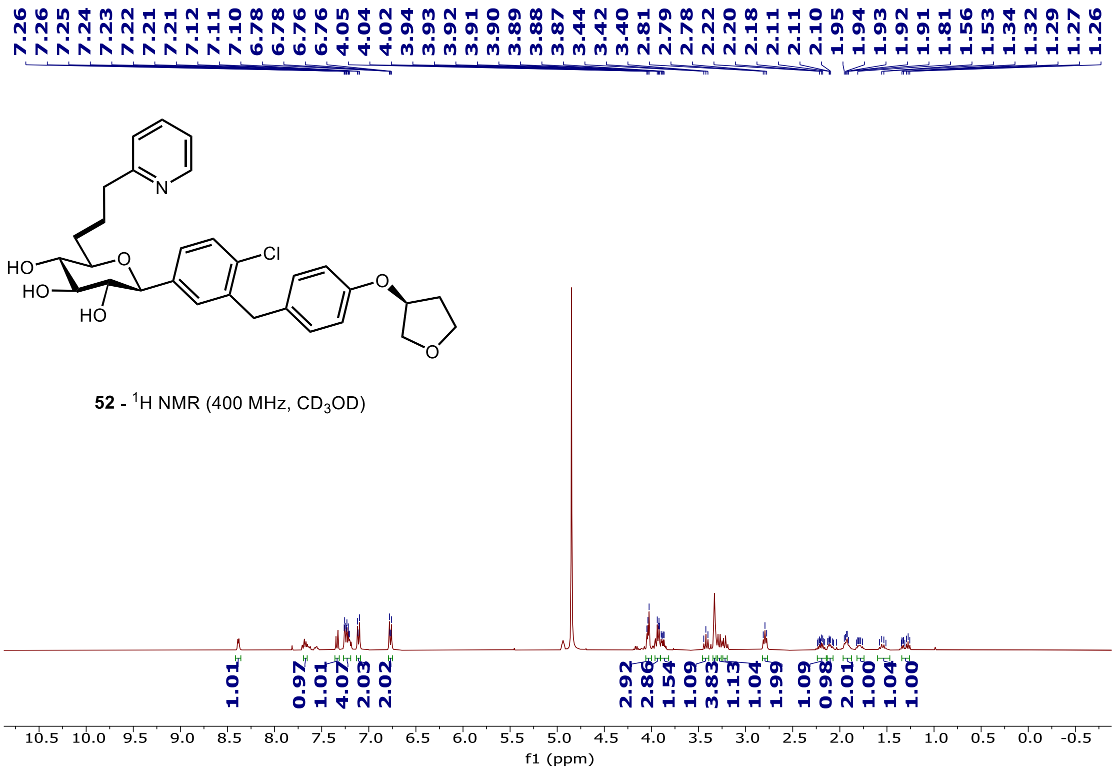

52 - ^{1}H NMR (400 MHz, CD_3OD)

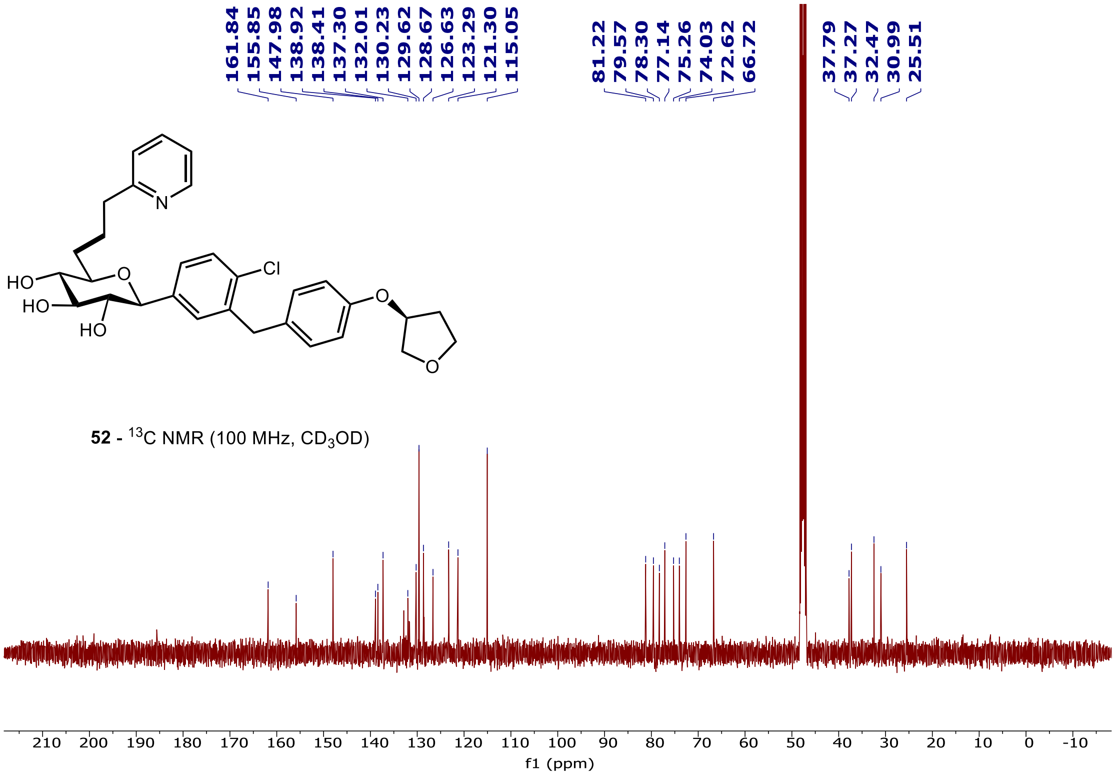

52 - ^{13}C NMR (100 MHz, CD_3OD)

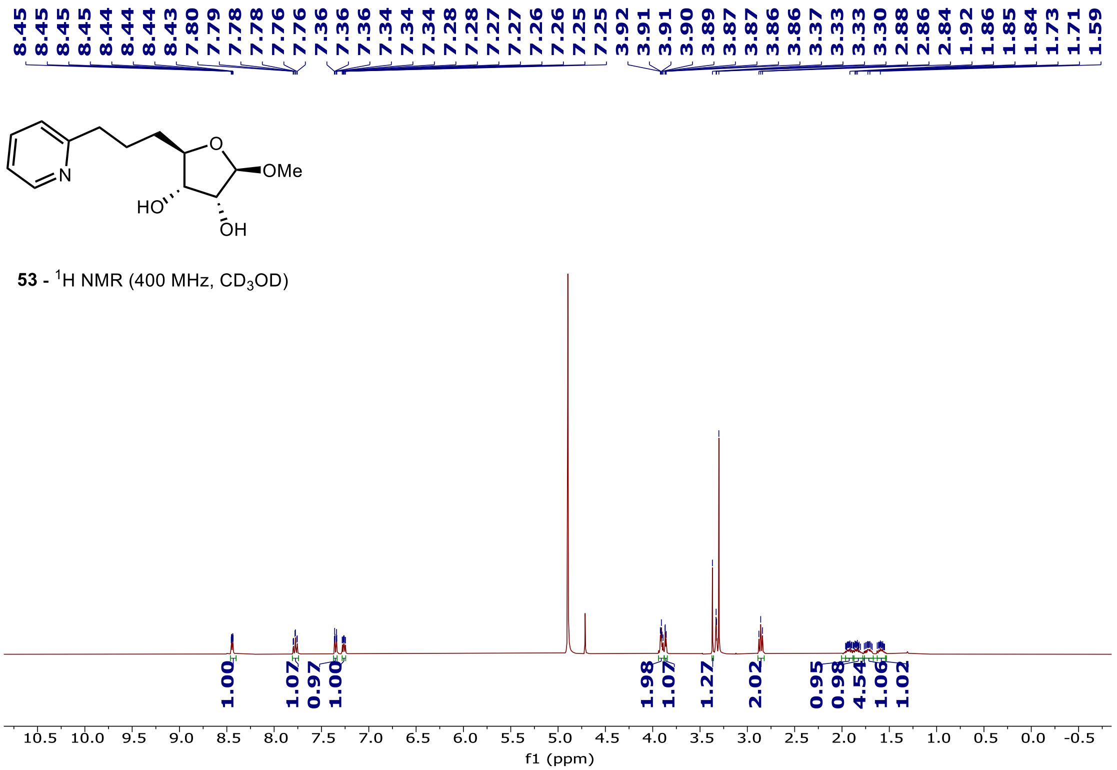

53 - ^{1}H NMR (400 MHz, CD_3OD)

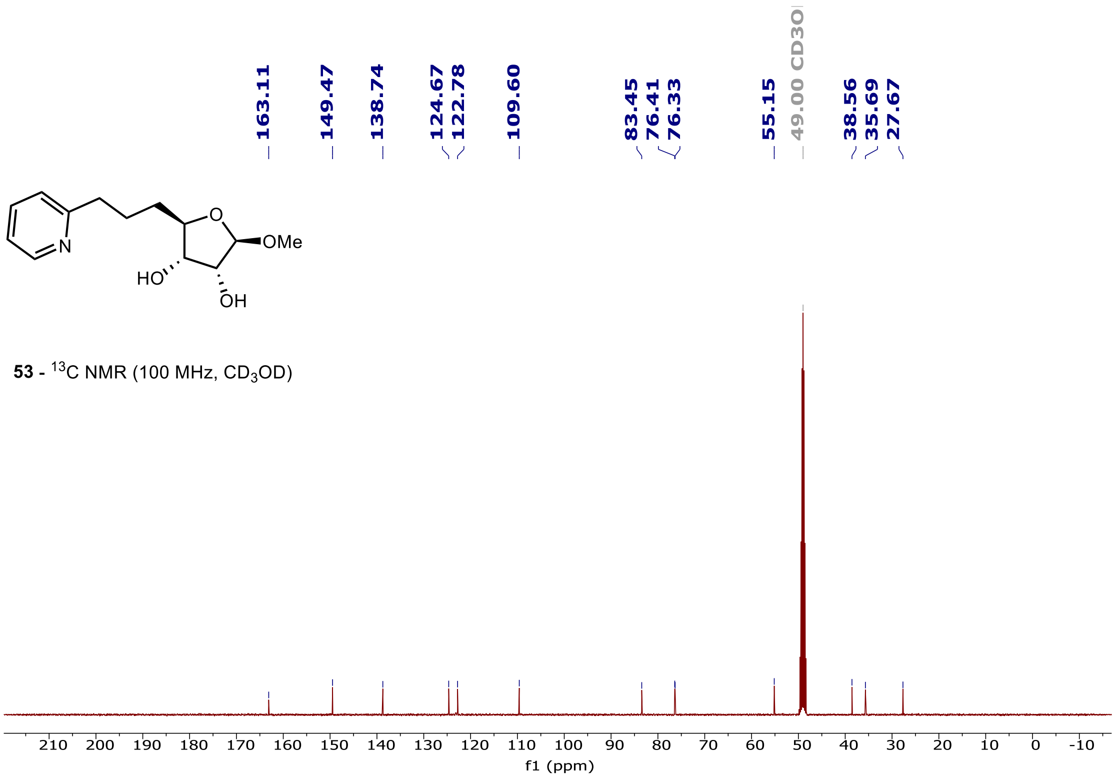

53 - ^{13}C NMR (100 MHz, CD_3OD)

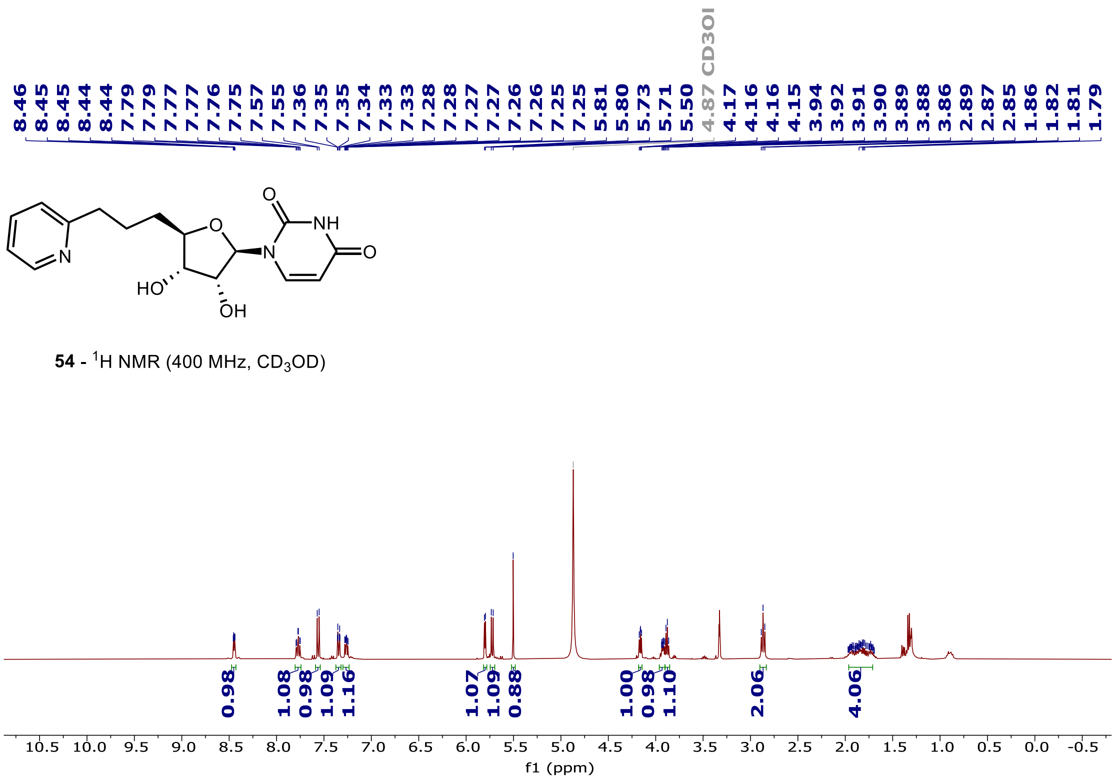

54 - ^{1}H NMR (400 MHz, CD_3OD)

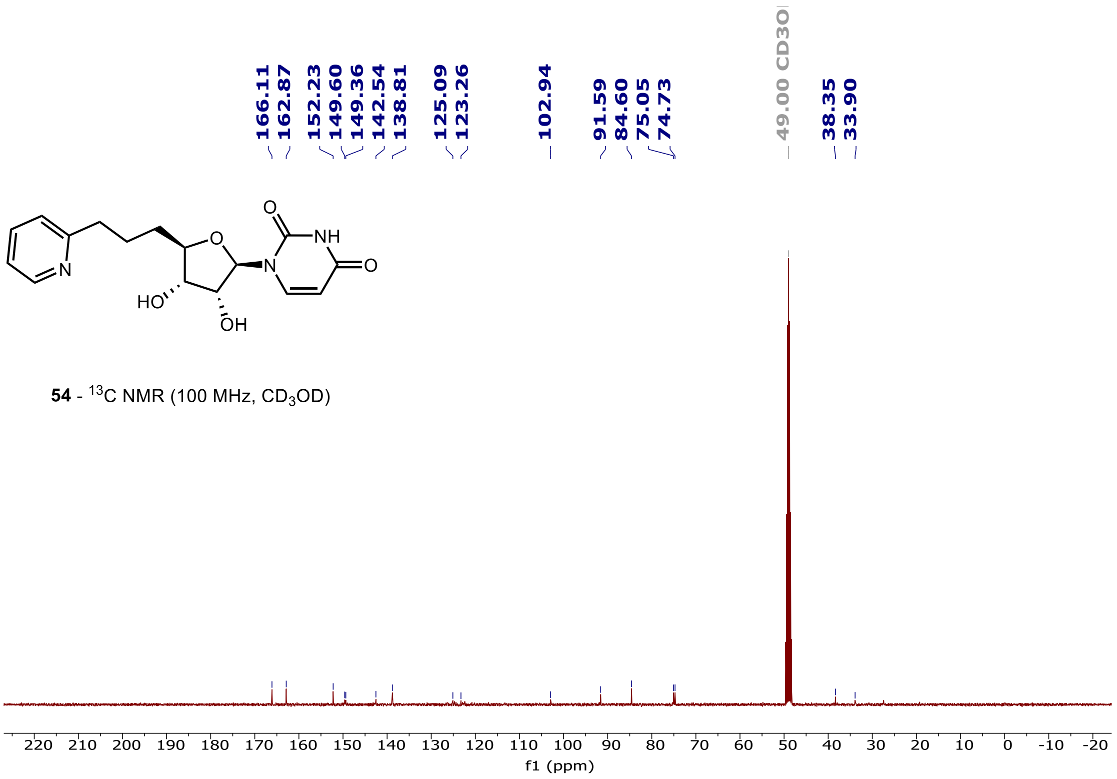

54 - ^{13}C NMR (100 MHz, CD_3OD)

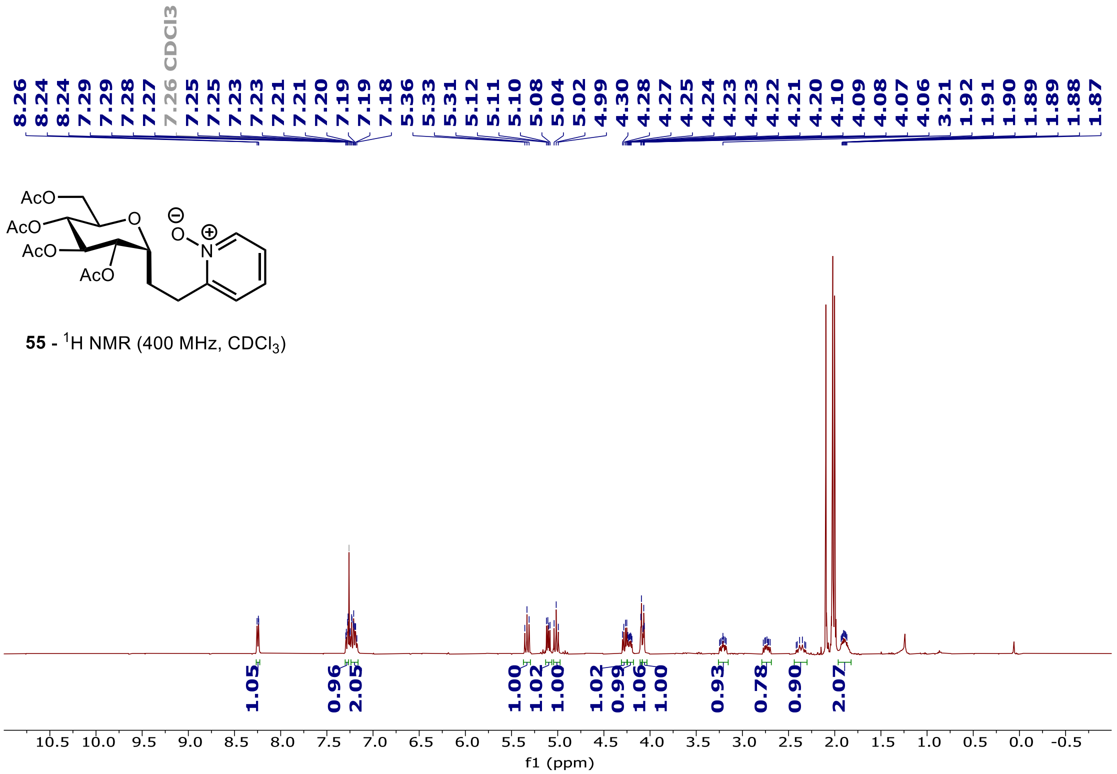

55 - ^{1}H NMR (400 MHz, $CDCl_3$)

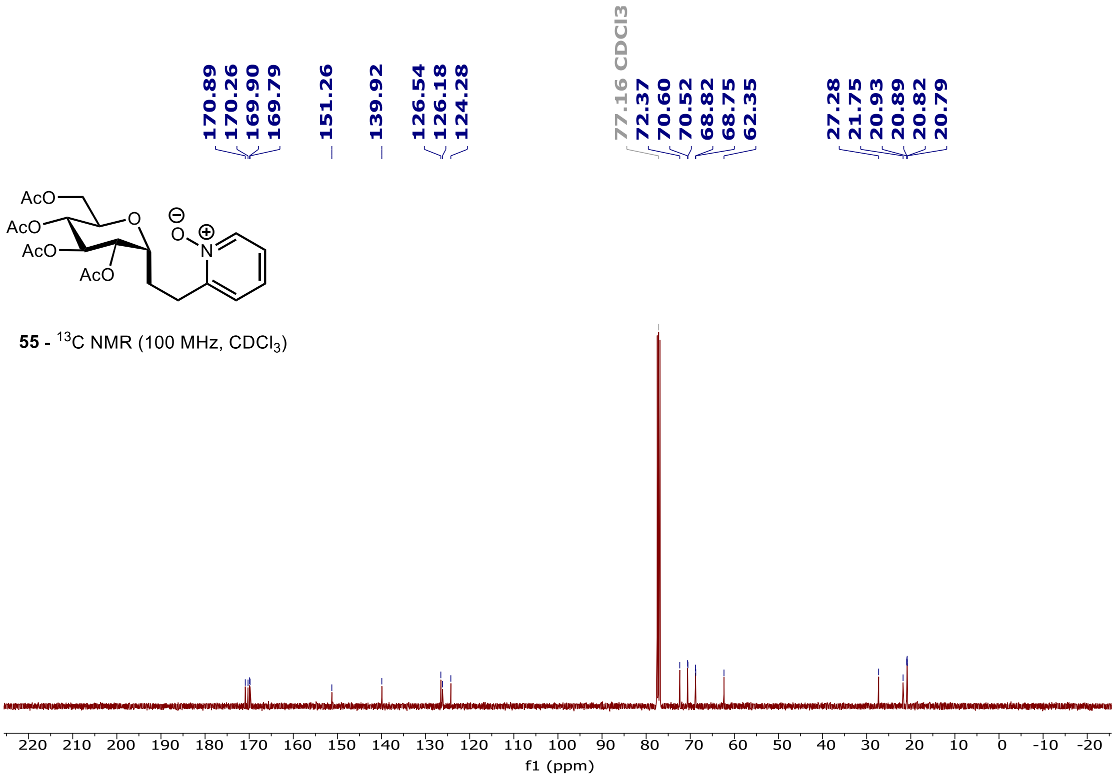

55 - ^{13}C NMR (100 MHz, $CDCl_3$)

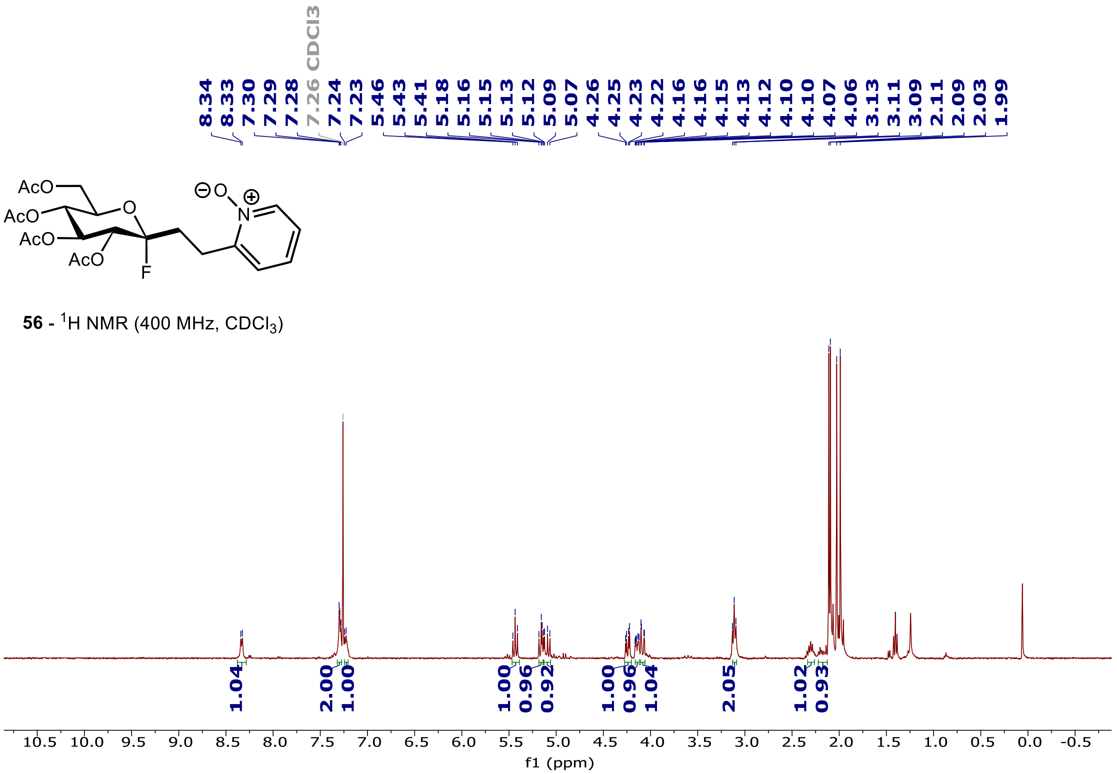

56 - ^{1}H NMR (400 MHz, $CDCl_3$)

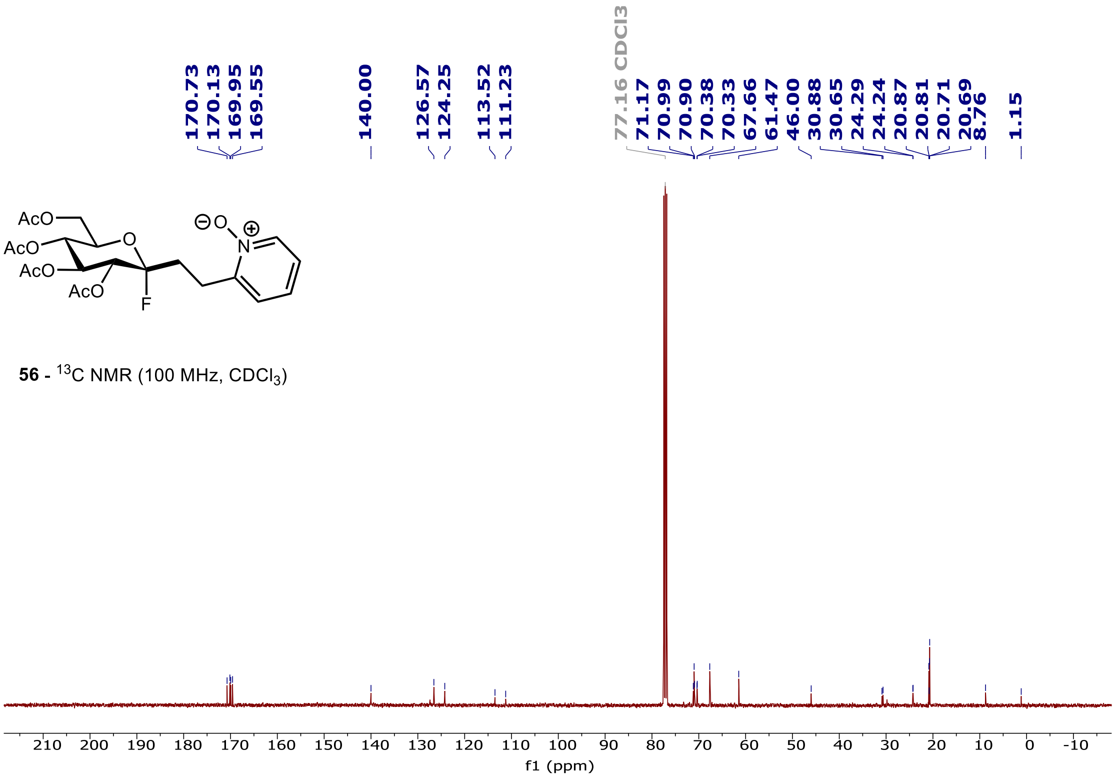

56 - ^{13}C NMR (100 MHz, $CDCl_3$)

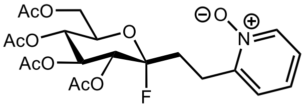

56 - ^{19}F NMR (376 MHz, $CDCl_3$)

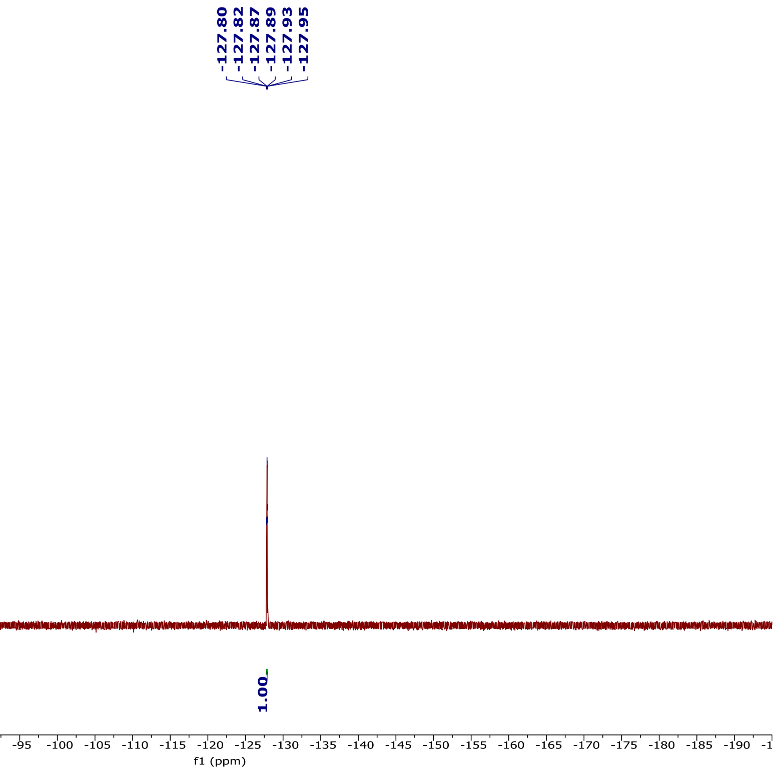

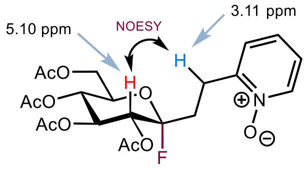
5.10 ppm
NOESY
3.11 ppm
AcO
AcO
AcO
AcO
H
H
O
N
O
F

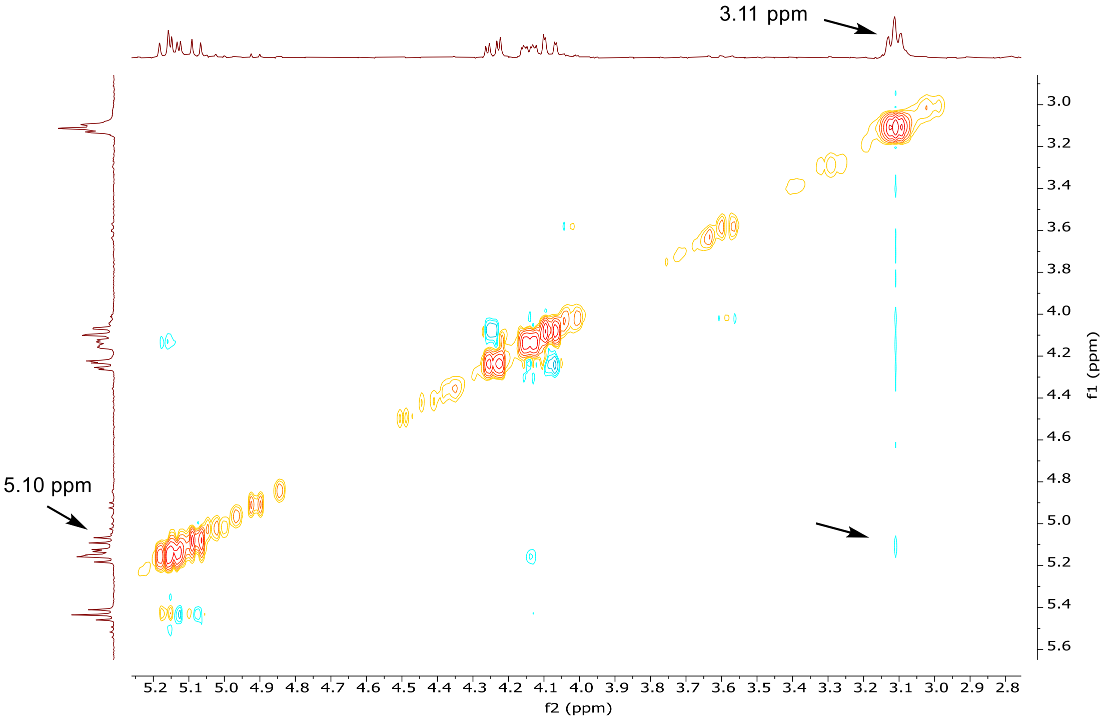
3.11 ppm
5.10 ppm
5.2 5.1 5.0 4.9 4.8 4.7 4.6 4.5 4.4 4.3 4.2 4.1 4.0 3.9 3.8 3.7 3.6 3.5 3.4 3.3 3.2 3.1 3.0 2.9 2.8
f2 (ppm)
3.0 3.2 3.4 3.6 3.8 4.0 4.2 4.4 4.6 4.8 5.0 5.2 5.4 5.6
f1 (ppm)

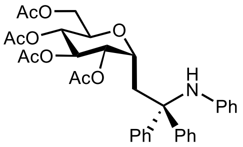

59 - ^{1}H NMR (400 MHz, $CDCl_3$)

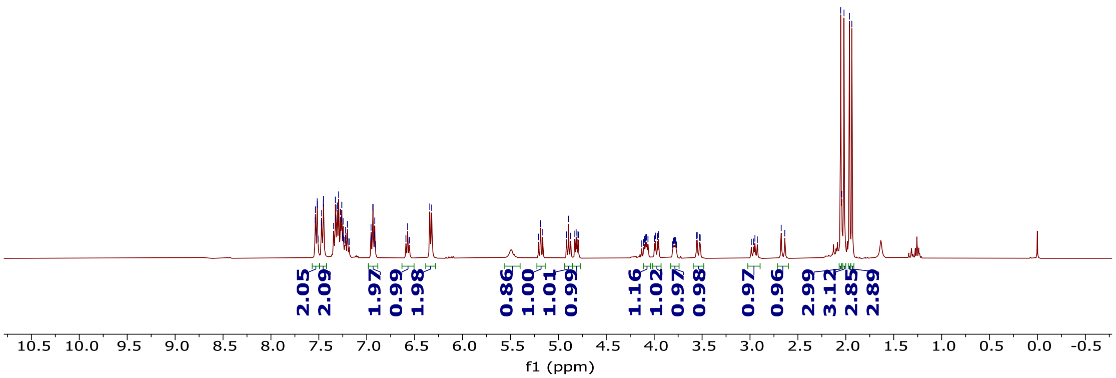

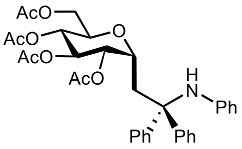

59 - ^{13}C NMR (100 MHz, $CDCl_3$)

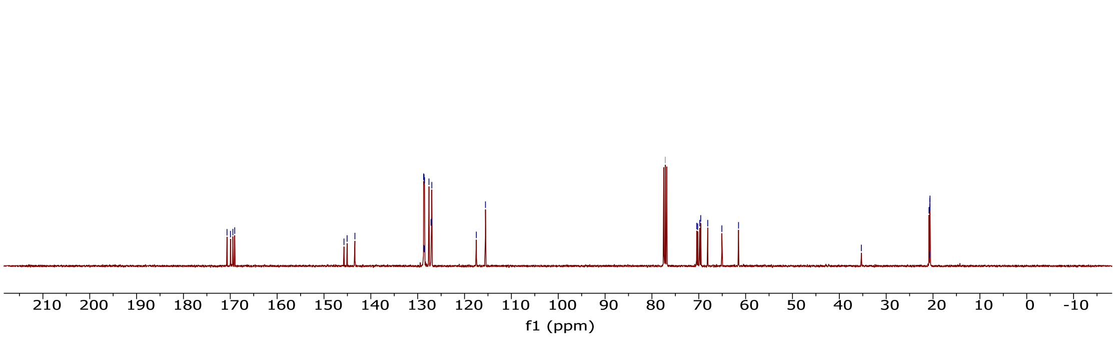

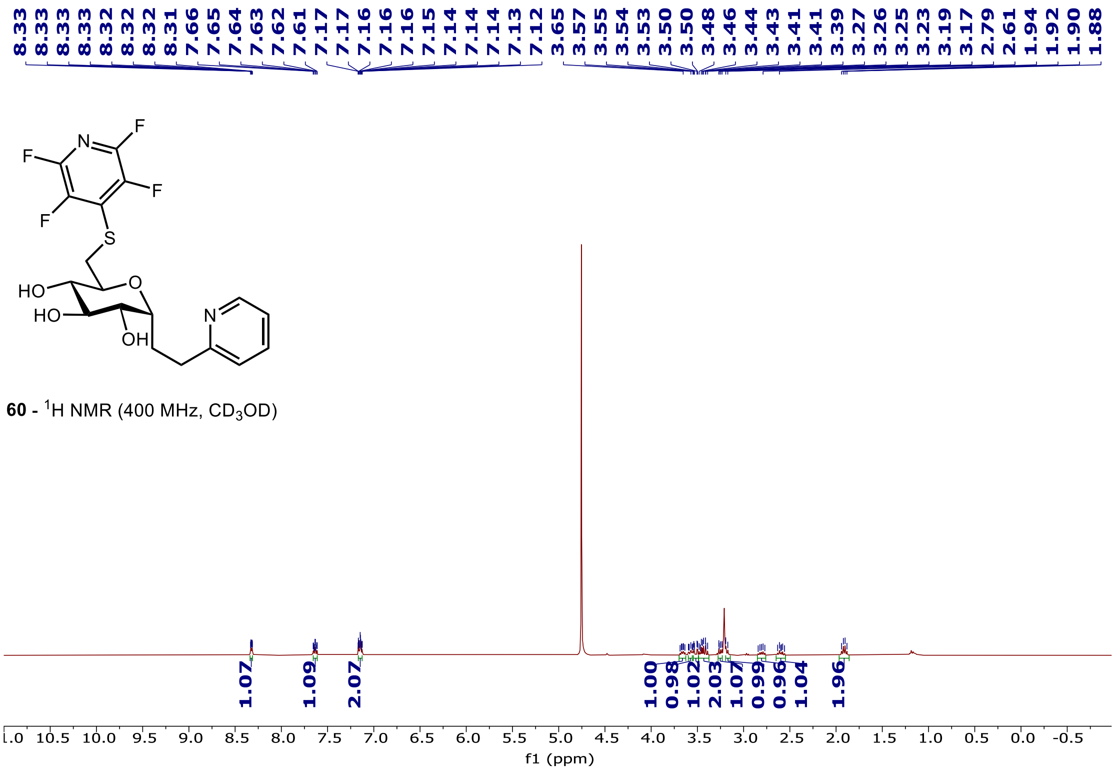

60 - ^{1}H NMR (400 MHz, CD_3OD)

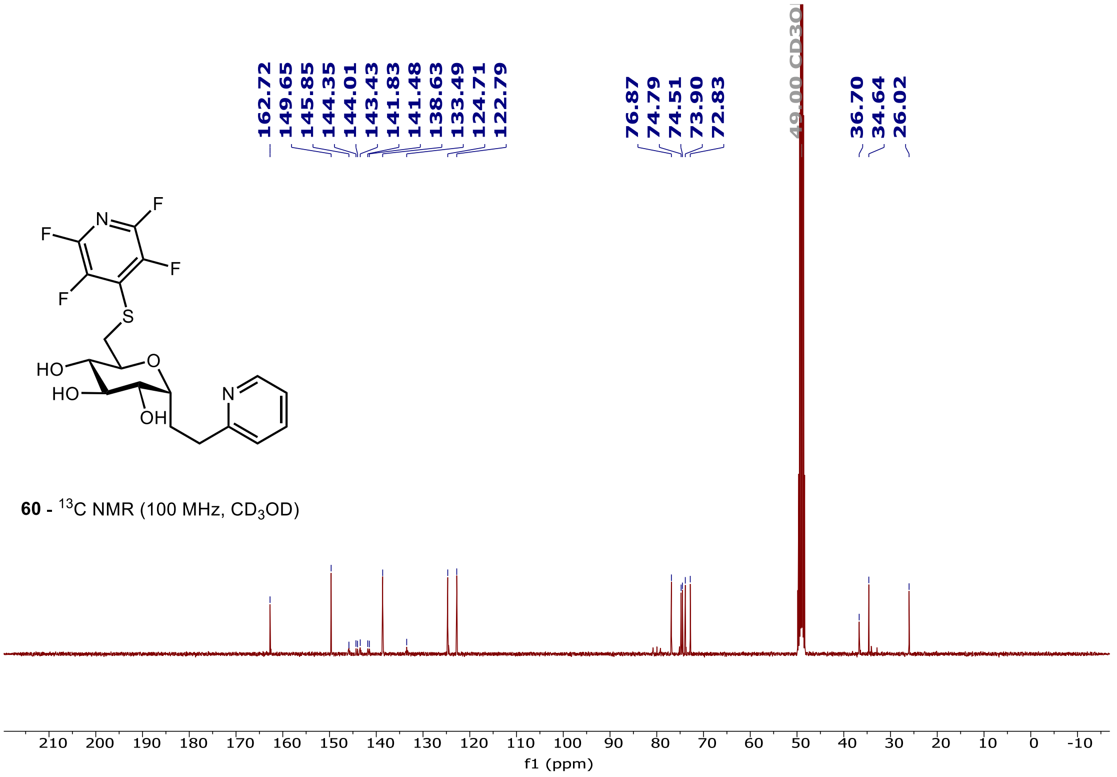

60 - ^{13}C NMR (100 MHz, CD_3OD)

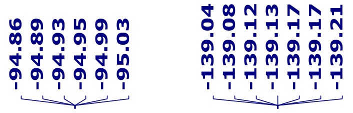

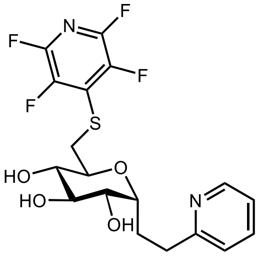

60 - ^{19}F NMR (376 MHz, CD_3OD)

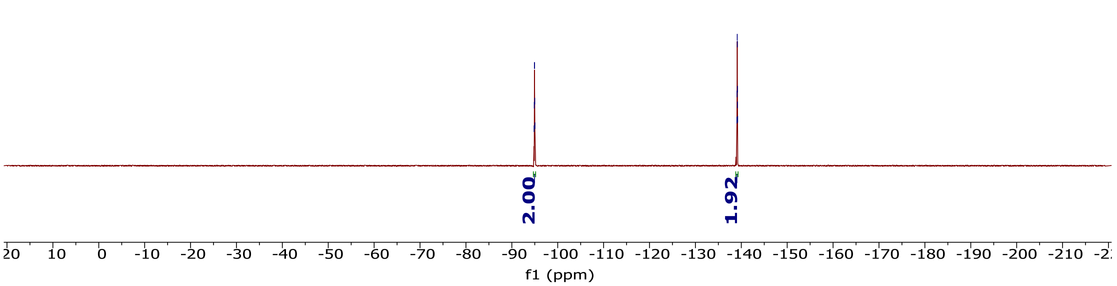

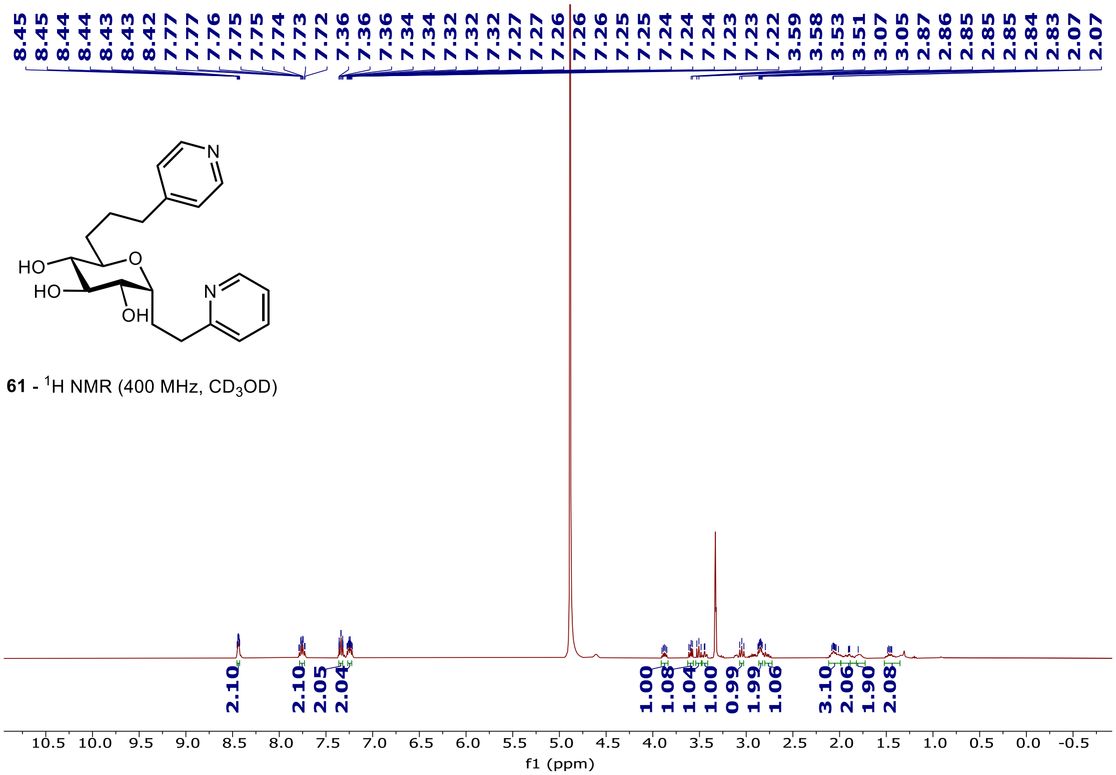

61 - ^{1}H NMR (400 MHz, CD_3OD)

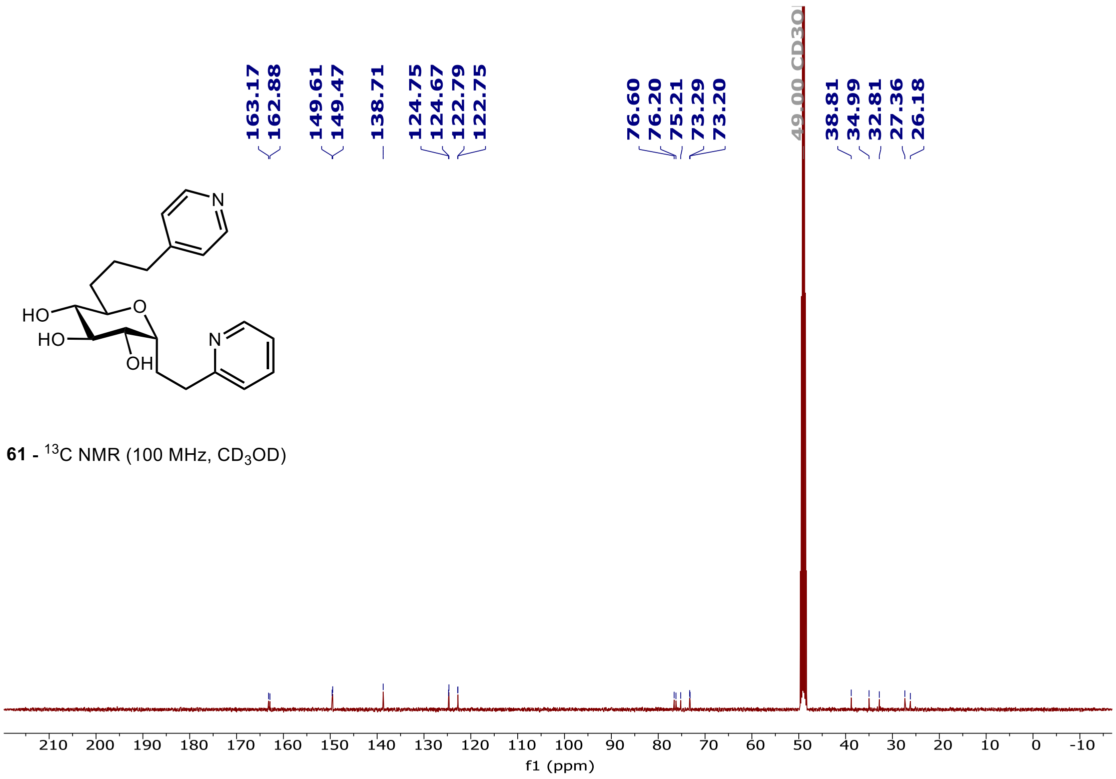

61 - ^{13}C NMR (100 MHz, CD_3OD)
